# Peptide Boronic Acids by Late‐Stage Hydroboration on the Solid Phase

**DOI:** 10.1002/advs.202400640

**Published:** 2024-05-29

**Authors:** Marius Werner, Julian Brinkhofer, Leon Hammermüller, Thomas Heim, Truc Lam Pham, Jonas Huber, Christian Klein, Franziska Thomas

**Affiliations:** ^1^ Institute of Organic Chemistry Heidelberg University Im Neuenheimer Feld 270 69120 Heidelberg Germany; ^2^ Medicinal Chemistry Institute of Pharmacy and Molecular Biotechnology (IPMB) Heidelberg University Im Neuenheimer Feld 364 69120 Heidelberg Germany

**Keywords:** hydroboration, late‐stage functionalization, nonproteinogenic amino acids, peptides, solid‐phase synthesis

## Abstract

Organoboron compounds have a wide range of applications in numerous research fields, and methods to incorporate them in biomolecules are much sought after. Here, on‐resin chemical syntheses of aliphatic and vinylogous peptide boronic acids are presented by transition metal‐catalyzed late‐stage hydroboration of alkene and alkyne groups in peptides and peptoids, for example on allyl‐ and propargylglycine residues, using readily available chemicals. These methods yield peptide boronic acids with much shorter linkers than previously reported on‐resin methods. Furthermore, the methods are regio‐ and stereoselective, compatible with all canonical amino acid residues and can be applied to short, long, and in part even “difficult” peptide sequences. In a feasibility study, the protected peptide vinylboronic acids are further derivatized by the Petasis reaction using salicylaldehyde derivatives. The ability of the obtained peptide boronic acids to reversibly bind to carbohydrates is demonstrated in a catch‐release model experiment using a fluorescently labeled peptide boronic acid on cross‐linked dextran beads. In summary, this highlights the potential of the target compounds for drug discovery, glycan‐specific target recognition, controlled release, and diagnostics.

## Graphical abstract

Transition metal‐catalyzed hydroboration of peptide alkenes and alkynes with readily available reactants selectively yields aliphatic‐ and vinylboronic acids in a straightforward, on‐resin, late‐stage functionalization approach. Reversible binding of the obtained peptide boronic acids to carbohydrates and further functionalization by Petasis reaction is also demonstrated.

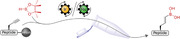

## 1 Introduction

The incorporation of boronic acids into peptides and peptidomimetics has recently attracted increasing interest in therapeutic development, drug delivery and chemical biology. Small peptides such as bortezomib or ixazomib with a C‐terminal α‐amino boronic acid moiety have been shown to display protease‐inhibitory activity.^[^ ^1^ ^]^ The unique property of boronic acids to form labile boronate esters with alcohols, especially 1,2‐diols such as carbohydrates and catechols, opens new possibilities for drug delivery, diagnostics and sensor development.^[^ ^2^ ^]^ Applications of boronic acids in chemical biology range from stapling and labeling via Petasis reaction,^[^ ^3^ ^]^ reversible stapling,^[^ ^4^ ^]^ N‐terminal modification of peptides,^[^ ^5^ ^]^ histidine‐directed modification of the N‐backbone in proteins,^[^ ^6^ ^]^ as well as the use in labeling reactions such as the Carboni‐Lindsey reaction.^[^ ^7^ ^]^ We therefore notice a great potential for efficient strategies to make peptide boronic acids readily available by chemical means.

**Figure 1:**
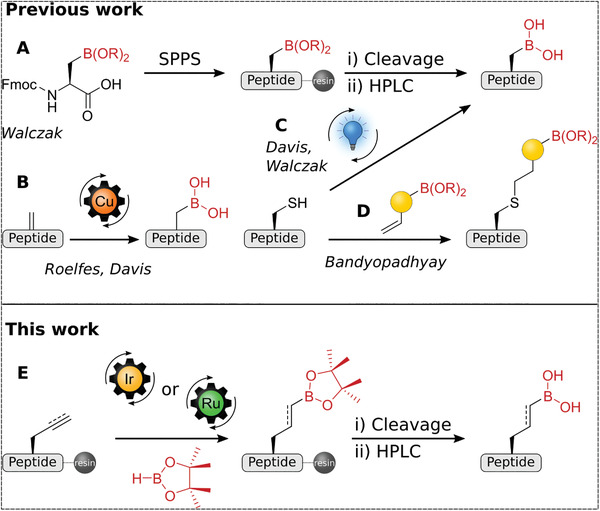
Strategies for the incorporation of boronic acids in peptide side chains. A–D) Previous work: A) Direct incorporation of boronoalanine in SPPS.^[^ ^9^ ^]^ B) Copper‐catalyzed borylation of dehydroalanine.^[^ ^11^ ^]^ C) Photocatalyzed desulfurative borylation.^[^ ^12^ ^]^ D) Thiol‐ene coupling of cysteine with boronic acid alkene compounds.^[^ ^10a^ ^]^ E) This work: Late‐stage alkene and alkyne hydroboration.

Due to the protease‐inhibiting effect of C‐terminal peptide boronic acids, synthesis efforts initially focused on α‐aminoboronic acids and their incorporation into peptides.^[^ ^8^ ^]^ In contrast, few methods are available to introduce boronic acids into peptide side chains (**Figure** **1**). For example, solid‐phase peptide synthesis (SPPS) provides direct access by incorporating boronoalanine as amino acid building block,^[^ ^9^ ^]^ or by reacting boronic acid‐containing compounds—usually aryl boronic acids—with cysteine or lysine side chains (Figure 1A,D).^[^ ^10^ ^]^ However, the first method demands the elaborate synthesis of the protected amino acid building block, while the latter results in long linkers that contain hetero atoms and therefore restricts the stability and biological utility of the obtained products. Roelfes et al. and Davis et al. presented a method for the post‐translational Cu(II)‐catalyzed borylation of dehydroalanine in peptides and proteins (Figure 1B),^[^ ^11^ ^]^ which unfortunately results in a diastereomeric product mixture. To overcome this problem, selective photochemical strategies have been developed to generate boronoalanine from cysteine residues in unprotected peptides and proteins (Figure 1C).^[^ ^12^ ^]^

Although post‐translational borylation methods are elegant and allow the borylation of, e.g., recombinant proteins, the unprotected nature of the peptides/proteins limits the possibilities of subsequent chemical transformations of the boronic acids. Furthermore, these methods are so far limited to the conversion of cysteine to boronoalanine. We therefore aimed to develop an on‐resin borylation approach at the late synthesis stage that allows a flexible choice of the borylation site and provides a protected peptide boronate that is susceptible to further chemical transformations. On‐resin late‐stage functionalization strategies circumvent the need to synthesize customized, protected amino acids by incorporating easily accessible, ideally commercially available amino acid building blocks into the peptide sequence of interest to serve as precursors for site‐selective modifications. This is not only cost‐efficient, but also provides access to modifications that are not stable under the conditions of SPPS and allows peptide diversification in high throughput while allowing for a larger spectrum of chemical reactions compared to late‐stage modification of unprotected peptides in solution.^[^ ^13^ ^]^

In this article, we report a transition metal‐catalyzed late‐stage hydroboration of peptide alkenes and alkynes to furnish alkyl‐ and vinyl peptide boronic acids, respectively. Inspired by Miyaura's method for iridium‐catalyzed hydroboration of alkenes with pinacolborane,^[^ ^14^ ^]^ we developed an iridium‐catalyzed hydroboration on the solid phase that gives the aliphatic boronic acids in excellent yields and regioselectivity, either on allylglycine residues or on allylated peptoid units. (*E*)‐selective synthesis of vinylboronic acids is achieved by ruthenium‐catalyzed on‐resin hydroboration with pinacolborane, e.g., at propargylglycine sites. These on‐resin hydroboration procedures are applicable to short and long peptides such as a 58 amino acid SH3 domain as well as to difficult peptide sequences and accept phosphorylated and in part sulfur‐containing amino acids. In a feasibility study, we show that the protected peptide vinylboronic acids can be further derivatized using the Petasis reaction. The ability of the chemically synthesized peptide boronic acids to bind to 1,2‐diols can be exploited for catch‐release applications. We have demonstrated this in a model experiment using a fluorescently labeled peptide boronic acid, which was selectively immobilized on cross‐linked dextran beads and released after hydrolysis of the boronate esters.

## 2 Results and Discussion

### 2.1 Late‐Stage Hydroboration of Peptide Alkenes

Hydroboration, pioneered by Brown, provides a straightforward route to boronic acids from alkenes or alkynes.^[^ ^15^ ^]^ However, the harsh conditions can lead to side reactions if this chemistry is applied to biomolecules such as peptides. The use of pinacolborane and transition‐metal catalysts in hydroboration allows stereoselective reaction under milder conditions,^[^ ^15^, ^16^ ^]^ paving the way for the development of a late‐stage hydroboration approach on resin‐bound peptides.

The late‐stage hydroboration was developed using the allylglycine containing peptide **P1═** (═ double bond) attached to a Rink amide MBHA polystyrene resin (**Table** **1**). **P1═** was synthesized by microwave‐assisted solid‐phase peptide synthesis. The peptide resin was then reacted with pinacolborane in the presence of an iridium catalyst at room temperature and overnight. Next, the peptide was cleaved from the resin under acidic conditions and analyzed by HPLC and MALDI‐TOF mass spectrometry (MS). Please note that the resulting pinacol boronates were still observed after acidic cleavage with TFA (trifluoroacetic acid), but hydrolyze during HPLC purification, resembling reports on similar pinacol protected boronate esters.^[^ ^17^ ^]^ We tested several iridium‐catalysts (Table 1, entries 1–4, Figures S1–S3, Supporting Information) and found that [Ir(COD)Cl]_2_/2dppm, which selectively catalyzes the anti‐Markovnikov addition of pinacolborane,^[^ ^14^ ^]^ gave the highest yield of the hydroboration product. As a by‐product, however, the norvaline‐containing peptide **P1─** (**─** single bond) was formed by hydrogenation, a common side‐reaction in hydroboration.^[^ ^18^ ^]^ The addition of the base *N*,*N*‐diisopropylethylamine (DIPEA) led to an improvement of the hydroboration yield from 78% to 90% and a reduction of the formation of the norvaline by‐product (entry 5). Performing the reaction with [Ir(COD)Cl]_2_/4PCy_3_ as catalyst was similarly successful (entry 6). During HPLC purification, the pinacolboronate was fully converted to the boronic acid **P1─**B(OH)_2_. The anti‐Markovnikov selectivity was verified by ^1^H‐NMR and ^1^H,^1^H‐COSY NMR experiments of the purified **P1─**B(OH)_2_ peptide, which was subjected to backbone hydrogen‐deuterium exchange with deuterium oxide prior to the experiments to facilitate analysis (Figure S4, Supporting Information). It should be noted that the catalyst and reagents were used at concentrations similar to those used in conventional solution‐phase transition metal‐catalyzed reactions, resulting in a high excess relative to the amount of peptide, as is common for on‐resin peptide modification.^[^ ^13^, ^19^ ^]^

**Table 1:** Optimization of the late‐stage hydroboration of **P1═** .

Entry^a)^	Catalyst	Additive	Yield [%]^b)^		
			P1─B(OH)_2_	P1=	P1─
1	[Ir(COD)Cl]_2_/2dppm	–	78	3	19
2	[Ir(COD)Cl]_2_/2dppe	–	56	36	8
3	[Ir(COD)Cl]_2_/2dppf	–	30	7	63
4	[Cp*IrCl_2_]_2_	–	48	0	52
5	[Ir(COD)Cl]_2_/2dppm	DIPEA	90	2	8
6	[Ir(COD)Cl]_2_/4PCy_3_	DIPEA	90	1	9

^a)^ 8.7 mg of resin‐bound **P1═**, equivalent to 5 µmol peptide, was incubated with 0.5 mL dry dichloromethane (DCM), 20 µmol iridium catalyst and 0.5 mmol pinacolborane (HBpin) for 16 h at room temperature;

^b)^ Yields are based on HPLC analysis. COD: 1,5‐Cyclooctadiene, dppm: 1,1‐Bis(diphenylphosphino)methane, dppe: 1,2‐bis(diphenylphosphino)ethane, dppf: 1,1′‐bis(diphenylphosphino)ferrocene, Cp*: pentamethylcyclopentadiene, PCy_3_: tricyclohexylphosphine DIPEA: *N*,*N*‐diisopropylethylamine. For HPLC traces and MALDI‐TOF‐MS see Figure S1 (Supporting Information).

We then explored the scope and limitations of our method by application to peptides covering all canonical amino acids. First, we synthesized peptide ligands of immunological receptors (MHC‐II), such as **P2═**, derived from the influenza matrix protein M1,^[^ ^20^ ^]^ **P3═**, based on a peptide vaccine targeting IDH1(R132H) mutations in glioma,^[^ ^21^ ^]^ and the methionine‐containing peptide **P4═**, derived from the histone mutant H3.3_K27M.^[^ ^22^ ^]^ These peptides are of interest in fundamental and applied immunological research. We then increased the complexity and thus synthetic challenge with the cysteine containing zinc‐finger peptide **P5═**;^[^ ^23^ ^]^ the 34 amino acid WW domain **P6═**, derived from the native WW domain of hPin1;^[^ ^24^ ^]^ and the 58 amino acid SH3 domain **P7═**.^[^ ^25^ ^]^ Finally, we decided to test the limits of our approach by performing the late‐stage hydroboration on difficult peptides that are either hydrophobic, such as the A6K peptide‐surfactant **P8═** with allylglycine at the C‐terminus followed by an alanine tail;^[^ ^26^ ^]^ or the bee‐venom melittin derived peptide **P9═**, with allylglycine placed in the hydrophobic part of the peptide;^[^ ^27^ ^]^ or are hydrophobic and aggregation‐prone, like the human calcitonin peptide hormone derived **P10═**.^[^ ^28^ ^]^ We complemented our scope by testing the phosphoserine containing peptide **P11═**, derived from the RNA polymerase II large subunit's C‐terminal domain^[^ ^29^ ^]^ and the peptoid **P12═**.

For **P2═**, **P3═**, **P5═**, **P6═**, **P7═** and **P12═**, the reaction worked very well and the hydroboration product was formed in good to very good yields (**Table** **2**, **Figure** **2**, Figures S5–S35, Supporting Information for yields see Table S1, Supporting Information), with only small amounts of norvaline formed as by‐product. The hydroboration of the phosphopeptide **P11═** was quantitative. However, when the late‐stage hydroboration was performed on the hydrophobic peptides **P8═**, **P9═**, and **P10═**, the yield was drastically reduced and most of the allylglycine was not converted within 16 h (Figures S22–S30, Supporting Information). We solved this problem by replacing the dppm ligand with PCy_3_ and extending the reaction time. In the case of **P9═**, the reaction time was increased to 40 h to maximize the yield, whereas in the case of **P8═** and **P10═**, the amounts of catalyst, DIPEA and HBpin were doubled, and the reaction time was increased to 88 h, resulting in a yield of 83% for **P8═**. The yield of **P10─**B(OH)_2_ could not be determined accurately, due to aggregation.

**Table 2:** Sequences of peptides subjected to late‐stage hydroboration.

Peptide^a)^	Sequence
**P1**	Ac‐GFXFGG‐NH_2_
**P2** ^b)^	H‐GPXKAEIAQRLE‐NH_2_
**P3**	H‐GWVKPIIIGHHAXGDQYRAT‐OH
**P4**	H‐KAPRKQLATKAARMSAPSTXGVKKPHR‐NH_2_
**P5**	H‐XPYKCPQCGKSFSQSSNLQKHQRTH‐NH_2_
**P6**	Ac‐KLPPGWEKR*Nle*SRSSGRVYYFNHITNASQXERPSG‐NH_2_
**P7**	H‐AEYVRALFDFNGNXEEDLPFKKGEILRIRDKPEEQWWNAENSEGKRGLIPVPYVEKYG‐NH_2_
**P8**	Ac‐AAAAAAX‐NH_2_
**P9**	H‐GIGAXLKVLTTGLPALISWIKRKRQQ‐NH_2_
**P10**	H‐CGNLSTCXLGTYTQDFNKFHTFPQTAIGVGAP‐NH_2_
**P11**	H‐YpSPTXPS‐NH_2_
**P12** ^c)^	Ac‐GF‐*N*(allyl/propargyl)‐GFGG‐NH_2_

^a)^ All peptides were synthesized with either allylglycine or propargylglycine at position X;

^b)^ **P2** was used as *tert*‐butoxycarbonyl *N*‐terminally protected resin‐bound peptide;

^c)^ **P12** was synthesized either *N*‐allyl‐ or *N*‐propargyl‐modified. For the structure, see Figures S35 and S89 (Supporting Information). Ac = acetyl, *Nle* = norleucine. For the yields, see Table S1 (Supporting Information).

**Figure 2:**
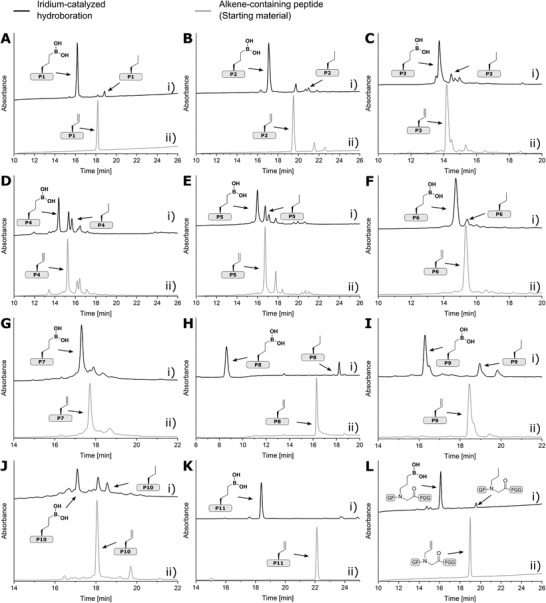
Scope of the late‐stage hydroboration of peptide alkenes. HPLC traces of crude hydroboration products A‐i)**P1─**B(OH)_2_, B‐i) **P2─**B(OH)_2_, C‐i) **P3─**B(OH)_2_, D‐i) **P4─**B(OH)_2_, E‐i) **P5─**B(OH)_2_ **_,_** F‐i) **P6─**B(OH)_2_, G‐i) **P7─**B(OH)_2_, H‐i) **P8─**B(OH)_2_, I‐i) **P9─**B(OH)_2_, J‐i) **P10─**B(OH)_2_, K‐i) **P11─**B(OH)_2_ and L‐i) **P12─**B(OH)_2_ after iridium‐catalyzed hydroboration; and crude A‐ii) **P1═**, B‐ii) **P2═**, C‐ii) **P3═**, D‐ii) **P4═**, E‐ii) **P5═** F‐ii) **P6═**, G‐i) **P7═**, H‐i) **P8═**, I‐i) **P9═**, J‐i) **P10═**, K‐i) **P11═** and L‐i) **P12═**. Hydroboration conditions are summarized in Section S2.3.5 in the Supporting Information.

In all cases, the peptide boronic acids could be readily separated from the allylglycine‐ or norvaline‐containing peptides during HPLC purification, even in case of longer peptides such as **P6** and **P7** (Figure 2). For **P4═** and **P5═**, containing cysteine or methionine residues, the hydroboration yield was lower compared to the other peptides (Figure 2D,E). The late‐stage hydroboration also works with other alkene groups, as demonstrated with the peptoid **P12═**. **P12═** was prepared by a literature‐known microwave‐assisted substitution of resin‐bound bromoacetic acid with allylamine,^[^ ^30^ ^]^ and reacted in excellent yield of 91% in the late‐stage hydroboration (Figure 2F).

### 2.2 Late‐Stage Hydroboration of Peptide Alkynes

**Figure 3:**
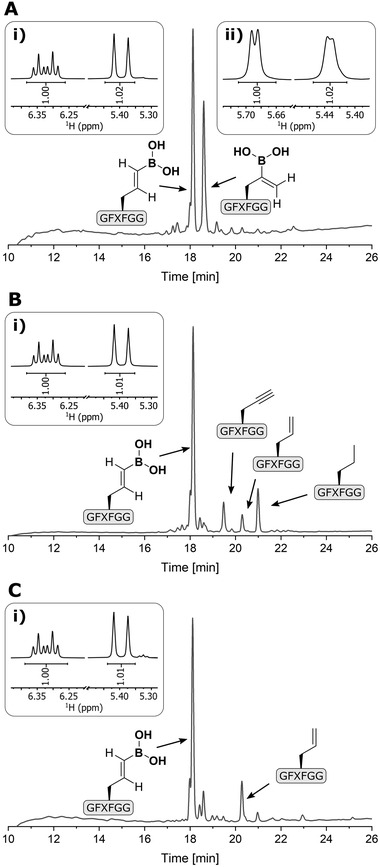
Stereoselectivity of late‐stage hydroboration of peptide alkynes. A) HPLC trace of crude **P1═** B(OH)_2_ after iridium‐catalyzed hydroboration. The peaks were separated and confirmed as anti‐Markovnikov and Markovnikov adduct based on ^1^H‐NMR of the products with a retention time of 18.1 min (i) and with a retention time of 18.6 min (ii). B) HPLC trace of crude **P1═**B(OH)_2_ from the [Ru(CO)(Cl)H(PPh_3_)_3_]‐catalyzed hydroboration (Reaction Conditions A). The main peak was separated and confirmed as anti‐Markovnikov adduct based on ^1^H‐NMR (i). C) HPLC trace of crude **P1═**B(OH)_2_ from the [Cp*Ru(PPh_3_)_2_Cl]‐catalyzed hydroboration (Reaction Conditions B). The main peak was separated and confirmed as anti‐Markovnikov adduct based on ^1^H‐NMR (i).

Vinylboronic acids display higher oxidative stability compared to aliphatic boronic acids and are conformationally constrained, which can be advantageous for the design of specific intermolecular recognition elements.^[^ ^31^ ^]^ To our knowledge, there is only one example of a vinylboronic acid being incorporated in peptides by amide coupling to a glutamate residue to form macrocyclic structures via a Petasis‐Borono‐Mannich reaction.^[^ ^3b^ ^]^ Given our success in late‐stage hydroboration at alkene moieties in resin‐bound peptides, we evaluated this reaction procedure on resin‐bound alkyne‐modified peptides to produce peptide vinylboronic acids. The peptide **P1≡** (**≡** triple bond) was thus synthesized containing the amino acid residue propargylglycine. The hydroboration of alkynes is challenging in terms of stereoselectivity and the reduction of the triple bond that yields either the alkene or the fully reduced by‐product.^[^ ^32^ ^]^ To address the issue of stereoselectivity, we initially tested the conditions of the (*Z*)‐selective rhodium‐ or iridium‐catalyzed hydroboration reported by Miyaura et al. using [Ir(COD)Cl]_2_/2dppm and [Ir(COD)Cl]_2_/4PCy_3_ as catalysts, cyclohexane as solvent and the alkyne peptide **P1≡** as model system.^[^ ^15b^ ^]^ However, no on‐resin hydroboration was observed under these conditions (Figures S36–S38, Supporting Information). We then used [Ir(COD)Cl]_2_/2dppm as catalyst and DCM as solvent, which was also used in the on‐resin hydroboration of alkenes, and observed complete hydroboration of **P1≡** (Figure S39, Supporting Information). HPLC analysis of the reaction mixture revealed two main peaks that were identified as *anti*‐Markovnikov adduct, the (*E*)‐vinylboronic acid (**Figure** **3**A‐i), and Markovnikov adduct (Figure 3A‐ii) by ^1^H‐NMR and ^1^H,^1^H‐COSY NMR experiments (Figures S40 and S41, Supporting Information). In order to improve the regioselectivity, other catalysts were tested, such as Schwartz's reagent [Cp_2_Zr(H)Cl],^[^ ^33^ ^]^ and [Rh(COD)Cl]_2_/2dppm, where the former did not catalyze the on‐resin hydroboration and the latter gave a mixture of by‐products, including a product with a mass indicative of double hydroboration, which according to the literature is the *gem*‐diborylalkane (Figures S42 and S43, Supporting Information).^[^ ^34^ ^]^ The catalyst [Rh(CO)(PPh_3_)_2_Cl], a variation of Wilkinson's catalyst known to selectively catalyze anti‐Markovnikov hydroboration,^[^ ^35^ ^]^ displayed improved regioselectivity for the anti‐Markovnikov product with low amounts of allylglycine and norvaline by‐products after reaction at room temperature for 16 h, but also resulted in a substantial amount of aliphatic boronic acid (Figure S44, Supporting Information). Finally, we tried [Ru(CO)(Cl)H(PPh_3_)_3_] as a catalyst, which is known to mediate *cis*‐hydroboration selectively, but its high air sensitivity makes it more difficult to handle in solid‐phase synthesis than, e.g., the iridium catalyst used for alkene hydroboration.^[^ ^36^ ^]^ Indeed, the ruthenium catalyst favored the formation of the (*E*)‐vinylboronic acid with small amounts of allylglycyl and norvalyl peptides as by‐products (**Table** **3**, Figure 3B and Figure S45, Supporting Information). Fortunately, the by‐products were readily separated from the vinylboronic acid by HPLC. The *E*‐configuration of the hydroboration product was confirmed by ^1^H NMR (Figure 3B‐i and Figure S46, Supporting Information). Due to its excellent regio‐ and stereoselectivity, [Ru(CO)(Cl)H(PPh_3_)_3_] is far superior to [Ir(COD)Cl]_2_/2dppm as a catalyst in the on‐resin hydroboration of alkyne‐containing peptides, although the undesired reduction of the triple bond was more pronounced under these reaction conditions. Performing the reaction at 60 °C resulted in the formation of the aliphatic boronic acid **P1─**B(OH)_2_ as byproduct, which could be suppressed by lowering the reaction temperature to 40 °C. Thereby, the reduction of the triple bond was also suppressed to a certain extent and the yield of the hydroboration product was increased to 67% if the reaction time was extended accordingly (Table 3, entry 4). The problem of stereoselectivity was solved, but the new reaction conditions required higher temperatures and longer reaction times, the latter resulting in higher catalyst loads due to the air sensitivity of the ruthenium catalyst and the difficulty of working under oxygen‐free conditions on the solid phase. We therefore investigated other ruthenium catalysts and found that [CpRu(PPh_3_)_2_Cl] (Cp: cyclopentadienyl), not previously reported in the context of hydroboration but known from the ruthenium‐catalyzed azide‐alkyne click reaction,^[^ ^37^ ^]^ yielded the vinylboronic acid at room temperature, with DCM proving to be a better solvent than toluene (Table 3, entries 6‐7; Figure S47, Supporting Information). However, the starting material was not fully converted after 16 h and extending the reaction time resulted in increased formation of by‐products (Figure S47, Supporting Information). Using the electron‐rich analog [Cp*Ru(PPh_3_)_2_Cl] (Cp*: pentamethylcyclopentadienyl) resulted in complete conversion of the starting material within 16 h at room temperature (Table 3, entry 8; Figures S47 and S48, Supporting Information). Reducing the catalyst load by half gave the desired hydroboration product in a very good yield of 73%. We also tested other ruthenium catalysts, [CpRu(MeCN)_3_]PF_6_ and [Cp*Ru(MeCN)_3_]PF_6_, known for the hydroboration of internal alkynes,^[^ ^38^ ^]^ but could not further improve the yield (Figures S49 and S50, Supporting Information).

**Table 3:** Conditions for the hydroboration of **P1≡**. .

Entry^a)^	Catalyst	Catalyst amount [µmol]	Solvent	HBpin [mmol]	Time [h]	Temperature [°C]	Yield [%]^b)^				
							P1═ B(OH)_2_ ^c)^	Isomers of P1═ B(OH)_2_^d)^	**P1≡**	**P 1═**	**P1─**
1	[Ru(CO)(Cl)H(PPh_3_)_3_]	20	0.8 mL toluene	0.5	16	60	40	6	7	8	39
2	[Ru(CO)(Cl)H(PPh_3_)_3_]	20	0.8 mL toluene	1.5	16	60	57	5	9	4	25
3	[Ru(CO)(Cl)H(PPh_3_)_3_]	20	0.8 mL toluene	1.5	16	40	47	5	35	10	3
4	[Ru(CO)(Cl)H(PPh_3_)_3_]	20	0.8 mL toluene	1.5	48	40	67	6	9	5	13
5	[CpRu(PPh_3_)_2_Cl]	20	0.5 mL DCM	0.5	16	25	26	3	44	25	2
6	[CpRu(PPh_3_)_2_Cl]	20	0.5 mL toluene	0.5	16	25	11	7	66	14	2
7	[CpRu(PPh_3_)_2_Cl]	20	0.5 mL DCM	0.5	40	25	29	11	25	31	4
8	[Cp*Ru(PPh_3_)_2_Cl]	20	0.5 mL DCM	0.5	16	25	61	14	0	19	6
9	[Cp*Ru(PPh_3_)_2_Cl]	10	0.5 mL DCM	0.5	16	25	73	14	0	11	2
10	[Cp*Ru(PPh_3_)_2_Cl]	7.5	0.5 mL DCM	0.5	16	25	71	12	0	14	3
11	[Cp*Ru(PPh_3_)_2_Cl]	5	0.5 mL DCM	0.5	16	25	54	12	13	18	3

^a)^ 8.7 mg of resin‐bound **P1≡**, equivalent to 5 µmol peptide;

^b)^ Yields are based on HPLC analysis;

^c)^ Including traces of aliphatic boronic acid by‐product; d) Regi‐o and stereoisomers. For HPLC traces and MALDI‐TOF‐MS (see Figures S45 and S47, Supporting Information).

**Figure 4:**
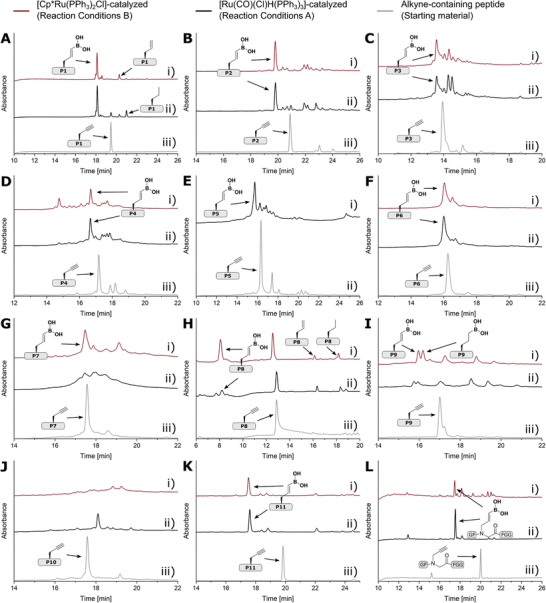
Scope of the late‐stage hydroboration of peptide alkynes. HPLC traces of crude A‐i) **P1═**B(OH)_2_, B‐i) **P2═**B(OH)_2_, C‐i) **P3═**B(OH)_2_, D‐i) **P4═**B(OH)_2_, F‐i) **P6═**B(OH)_2_, G‐i) **P7═**B(OH)_2_, H‐i) **P8═**B(OH)_2_, I‐i) **P9═**B(OH)_2_, reaction mixture of J‐i) **P10≡**, K‐i) **P11═**B(OH)_2_ and L‐i) **P12═**B(OH)_2_ after [Cp*Ru(PPh_3_)_2_Cl]‐mediated hydroboration; crude A‐ii) **P1═**B(OH)_2_, B‐ii) **P2═**B(OH)_2_, C‐ii) **P3═**B(OH)_2_, D‐ii) **P4═**B(OH)_2_, E‐i) **P5═**B(OH)_2_, F‐ii) **P6═**B(OH)_2_, G‐ii) **P7═**B(OH)_2_, H‐ii) **P8═**B(OH)_2_, I‐ii) **P9═**B(OH)_2_, reaction mixture of J‐ii) **P10≡**, K‐ii) **P11═**B(OH)_2_ and L‐ii) **P12═**B(OH)_2_ after [Ru(CO)(Cl)H(PPh_3_)_3_]‐mediated hydroboration; and crude A‐iii) **P1≡**, B‐iii) **P2≡**, C‐iii) **P3≡**, D‐iii) **P4≡**, E‐ii) **P5≡**, F‐iii) **P6≡**, G‐iii) **P7≡**, H‐iii) **P8≡**, I‐iii) **P9≡**, J‐iii) **P10≡**, K‐iii) **P11≡** and L‐iii) **P12≡**. Hydroboration conditions are summarized in Sections S2.3.7 and S2.3.8 in the Supporting Information.

Having optimized two reaction conditions with **P1≡**, we explored their general applicability to our peptide scope. **P2≡** to **P12≡** were first subjected to optimized hydroboration conditions with [Ru(CO)(Cl)H(PPh_3_)_3_] (Reaction Conditions A, Table 3, entry 4) and then to optimized hydroboration conditions with [Cp*Ru(PPh_3_)_2_Cl] (Reaction Conditions B; Table 3, entry 10). Hydroboration with Reaction Conditions A initially led to side reactions due to the high amount of pinacolborane, which was therefore reduced to a third while the reaction time was increased to 64 h. These conditions ensured successful hydroboration of **P2≡, P3≡, P4≡, P5≡,** and **P6≡** (**Figure** **4**B–F, Figures S51–S67 and Table S2, Supporting Information). In the case of **P11≡**, the reaction time had to be reduced to 16 hours and reaction temperature was reduced to room temperature, as the unusually high reactivity of **P11≡** would otherwise lead to an increase of by‐products (Figures S85–S88, Supporting Information). A similar effect was observed with **P12≡**, where a large amount of aliphatic boronic acid **P12─**B(OH)_2_ was formed under the standard Reaction Conditions A. We therefore shortened the reaction time to 16 h, thereby suppressing the undesired reduction of the hydroboration product. The hydroboration of **P7≡** did not give a satisfactory yield (Figure 4G and Figures S89–S92, Supporting Information) and the hydroboration of the difficult peptides **P8≡**, **P9≡**, and **P10≡** was not successful (Figure 4H–K, Figures S73–S80, Supporting Information).

The application of Reaction Conditions B to our peptide scope was successful for almost all peptides. Even the difficult peptides **P8≡** and **P9≡** as well as the 58 amino acid peptide **P7≡** were hydroborated to give the vinylboronic acid in moderate to good yields, although the alkyl boronic acid was formed in 28% for **P9≡** and in traces for **P7≡** (Figure 4G–J and Figures S70–S72, Supporting Information).

Hydroboration of the aggregation‐prone peptide **P10≡** was not successful, demonstrating the limitations of our method (Figure 4K). In addition, hydroboration of **P4≡** resulted in a mixture of unidentifiable products, possibly indicating an incompatibility of [Cp*Ru(PPh_3_)_2_Cl] and cysteine. Despite these limitations, the scope chosen impressively demonstrated the practicality of the on‐resin hydroboration for a wide range of propargylated peptides to peptide vinylboronic acids, which is unprecedented to our knowledge.

### 2.3 Petasis‐Borono‐Mannich Reaction of Peptide Vinylboronic Acids

The Petasis‐Borono‐Mannich multicomponent reaction between an amine, carbonyl component and boronic acid is widely used in diversity‐oriented organic and medicinal chemistry^[^ ^39^ ^]^ and has also been shown to be applicable to solid‐phase synthesis.^[^ ^3^, ^40^ ^]^

**Figure 5:**
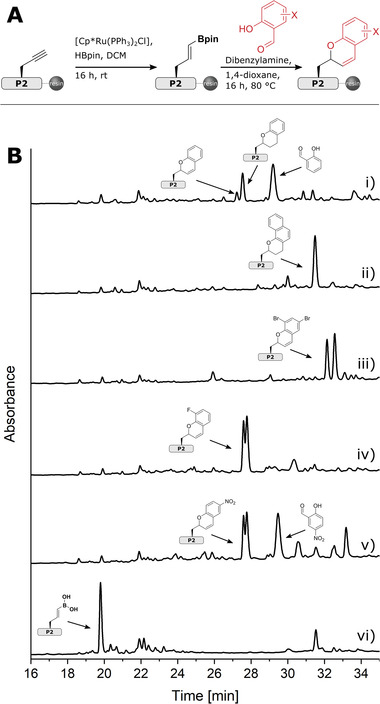
Late‐stage Petasis‐Borono‐Mannich reaction. A) Reaction scheme. B‐i‐v) HPLC traces of crude peptides after Petasis reaction in comparison to B‐vi) **P2═**B(OH)_2_.

Vinylboronic acids and trifluorovinylborates can be subjected in a Petasis reaction with salicylaldehyde derivatives, with subsequent ejection of the amine, to access 2*H*‐chromenes, which are found in many natural products and pharmaceuticals.^[^ ^41^ ^]^ We were therefore interested in whether our protected resin‐bound peptide vinylboronates would be suitable starting materials for introducing 2*H*‐chromene moieties into peptides. We applied the conditions of Wang and Finn^[^ ^41^ ^]^ on the solid phase and to peptide **P2** as a feasibility study. Since the hydrolysis of vinylboronates reported in the literature is very time consuming (four cycles of transesterification with diethanolamine for 4–18 h each),^[^ ^3^ ^]^ we decided to perform the on‐resin Petasis reaction directly on the peptide vinylboronates. After hydroboration of **P2≡** using Reaction Conditions B, we equilibrated the peptide vinylboronate with dibenzylamine and five different salicylaldehyde derivatives—salicylaldehyde, 1‐hydroxy‐2‐naphthaldehyde, 3,5‐dibromo‐salicylaldehyde, 3‐fluoro‐salicylaldehyde and 5‐nitro‐salicylaldehyde—for 16 h at 80 °C (**Figure** **5** and Figures S93–S97, Supporting Information). Complete conversion of the starting material was observed in all cases. The formation of a stereogenic center gives rise to two diastereomers, some of which are resolved by HPLC (Figure 5B‐iii–v). Interestingly, the Petasis reactions with salicylaldehyde and 1‐hydroxy‐2‐naphthaldehyde yielded the corresponding chromane derivatives as major products, which we attribute to residual ruthenium species adsorbed on the peptide resin.^[^ ^42^ ^]^ As this is a feasibility study, which is not the focus of this manuscript, we have not investigated this result further at this stage. However, chromane moieties are also commonly found in natural products and future studies should focus on this peptide derivatization, which is new to our knowledge, as well as the development of a diastereoselective Petasis reaction on resin‐bound peptides.

### 2.4 Catch and Release of Peptide Boronic Acids

**Figure 6:**
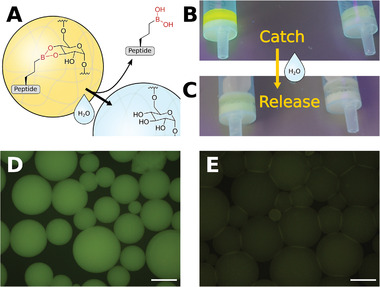
Catch and release of peptide boronic acids on Sephadex beads. A) Schematic of peptide boronic acids immobilized to dextran beads in DMSO and being released upon hydrolysis. B) Dextran after incubation with FAM**‐P5─**B(OH)_2_ (left) and FAM‐**P5═** (right), followed by washing with DMSO. C) FAM‐**P5─**B(OH)_2_ (left) is released upon hydrolysis with water. D,E) Fluorescence microscopy images of dextran beads after incubation with D) FAM**‐P5─**B(OH)_2_ and E) FAM‐**P5═**, followed by washing with DMSO. Excitation wavelength *λ*_ex_ =  475 nm, scale bar = 200 µm.

The ability of boronic acids to form esters with diols, especially carbohydrates, allows a wide range of applications for drug delivery, as shown by Ding et al. with a pH‐responsive bortezomib‐dextran conjugate or by Almutairi et al. with a reactive oxygen species (ROS)‐scavenging naproxen‐dextran conjugate.^[^ ^2^, ^43^ ^]^ To demonstrate that the peptide boronic acids prepared by our method can be used for such applications, we designed a catch‐release model experiment, in which we immobilized 5(6)‐carboxyfluorescein (FAM)‐labeled FAM**‐P5─**B(OH)_2_ on cross‐linked dextran polymer beads (Sephadex G‐100, **Figure** **6**A). FAM**‐P5─**B(OH)_2_ was prepared by hydroboration of **P5═** and subsequent amide coupling with FAM (Figures S98 and S99, Supporting Information). The boronic acid FAM‐**P5─**B(OH)_2_ and the control peptide FAM**‐P5═** were incubated with the dextran beads in DMSO (dimethylsulfoxide) in presence of DIPEA as base. After washing with DMSO, FAM**‐P5─**B(OH)_2_ was shown to be immobilized to the dextran beads, whereas FAM‐**P5═** was washed off (Figure 6B). Upon addition of water, the peptide boronic acid was released (Figure 6C). The beads treated with FAM**‐P5─**B(OH)_2_ were highly fluorescent, indicating that the peptide was successfully immobilized on the dextran polymer (Figure 6D), whereas beads treated with FAM‐**P5═** showed only weak fluorescence most likely caused by non‐specific peptide binding (Figure 6E).

## 3 Conclusion

This work presents a new method for the preparation of peptide boronic acids by on‐resin late‐stage hydroboration of alkene or alkyne peptides and peptoids. We incorporated allyl‐ and propargyl glycine, as well as allyl and propargyl backbone modifications into peptides by solid‐phase peptide synthesis and subjected them to metal‐catalyzed hydroboration with pinacolborane. The hydroboration of alkenes was almost quantitatively achieved with the iridium catalyst [Ir(COD)Cl]_2_/2dppm, selectively yielding the *anti*‐Markovnikov adduct. This method was applied to short and long peptides such as a 34 amino acid WW domain or a 58 amino acid SH3 domain, and could even be optimized for “difficult” peptides such as melittin (**P9**) and to some extend the aggregation‐prone calcitonin (**P10**). Thus, researchers interested in using this functionalization approach are provided with a range of conditions to successfully address their peptide sequence of interest. On‐resin hydroboration of alkynes was carried out using the ruthenium catalyst Ru(CO)(Cl)H(PPh_3_)_3_, which selectively yields the (*E*)‐vinylboronic acid. More complex peptides such as the WW domain **P6≡** were hydroborated under these conditions, but long reaction times and elevated temperatures were required, leading to increased by‐product formation, especially for less reactive peptides. The use of [Cp*Ru(PPh_3_)_2_Cl], which was not known in the context of hydroboration prior to this study, allowed the reaction temperature to be reduced to room temperature, improving yield and catalyst loading. Alkyne hydroboration was successful with all peptides except the difficult **P10**. Despite these few limitations this is the first report on the incorporation of vinylboronic acids via a late‐stage synthesis approach and in a stereoselective manner. In a feasibility study, we have demonstrated that the peptide vinylboronates can be further derivatized using the Petasis‐Borono‐Mannich reaction with salicylaldehyde derivatives. By applying a catch‐release approach of fluorescently labeled peptide FAM‐**P5─**B(OH)_2_, we highlighted the prospects of peptide boronic acids in biomedical research and development, e.g., for drug delivery or glycan‐specific target recognition.

Peptide boronic acids have enormous potential for medicinal chemistry,^[^ ^1^, ^17^, ^44^ ^]^ and drug delivery, particularly due to their ability to reversibly form boronate esters with, e.g., carbohydrate residues,^[^ ^2b^ ^]^ and methods have been described that provide site‐selective borylation at dehydroalanine and cysteine residues in unprotected proteins and peptides.^[^ ^11^, ^12^ ^]^ While these reactions are indeed very powerful, they are carried out in solution, restricting the versatility of the peptide/protein boronic acids formed, e.g. with respect to subsequent chemical transformations.

Previously reported on‐resin borylation reactions typically include conjugation of boronic acid‐containing residues to cysteine or lysine side chains, resulting in long and flexible linkers.^[^ ^10^ ^]^ Our on‐resin hydroboration is considerably more flexible: we can introduce aliphatic and vinylboronic acids, either at an amino acid side chain or as backbone modification. The resulting linkers are short and do not contain hetero atoms, which means that the peptide boronic acids synthesized in this way resemble their natural analogs. This allows for targeted interactions with geometrically restricted enzymatic active sites, ligand binding pockets or glycan residues on glycoproteins. In addition, on‐resin functionalization approaches for protected peptides are characterized by simple and rapid work‐up and allow further chemical diversification: Vinylboronic acids are versatile starting materials for the Petasis reaction, as we have shown, and the Suzuki‐Miyaura or Chan‐Lam couplings, opening up new avenues for the synthesis of as‐yet inaccessible peptide‐hybrids with applications in bioorganic and medicinal chemistry.^[^ ^39^, ^45^ ^]^ Alkene and alkyne groups are valuable and widely available handles for late‐stage functionalization by azide–alkyne click reactions or cross‐coupling reactions.^[^ ^19^, ^46^ ^]^ On‐resin late‐stage hydroboration further increases their potential for peptide diversification.

## Conflict of Interest

The authors declare no conflict of interest.

## Supporting information

Supporting Information

## Data Availability

The data that support the findings of this study are available in the Supporting Information of this article.
