# Supplementary material for: Peptide Boronic Acids by Late‐Stage Hydroboration on the Solid Phase
Source: Adv Sci (Weinh). 2024 May 29;11(28):2400640. doi: 10.1002/advs.202400640 (PMC11267286; doi:10.1002/advs.202400640)
Supplement: Supplementary file 1 — Supporting Information [file ADVS-11-2400640-s001.pdf]

ADVANCED SCIENCE
Open Access

Supporting Information

for *Adv. Sci.*, DOI 10.1002/advs.202400640

Peptide Boronic Acids by Late-Stage Hydroboration on the Solid Phase

Marius Werner, Julian Brinkhofer, Leon Hammermüller, Thomas Heim, Truc Lam Pham, Jonas Huber, Christian Klein and Franziska Thomas**

Supporting Information

Peptide Boronic Acids by Hydroboration on the Solid Phase

Marius Werner,[a,b] Julian Brinkhofer,[a] Leon Hammermüller,[a] Thomas Heim,[a] Truc Lam Pham,[a] Jonas Huber,[a] Christian Klein[b] and Franziska Thomas*[a]*

[a]Institute of Organic Chemistry, Heidelberg University, Im Neuenheimer Feld 270, 69120 Heidelberg, Germany

[b]Medicinal Chemistry, Institute of Pharmacy and Molecular Biotechnology (IPMB), Heidelberg University, Im Neuenheimer Feld 364, 69120 Heidelberg, Germany

*Correspondence:
c.klein@uni-heidelberg.de
franziska.thomas@oci.uni-heidelberg.de

Table of Contents

1 Supplementary figures S1
2 Experimental procedures S100
2.1 Reagents and solvents S100
2.2 Software for data analysis and visualization S100
2.3 Peptide synthesis, functionalization and purification S100
2.4 Catch-release experiment and fluorescence microscopy S111
2.5 NMR spectroscopy S113
2.6 Mass spectrometry S113
3 ESI-MS S114
4 Abbreviation S143
5 Literature S144

1 Supplementary figures

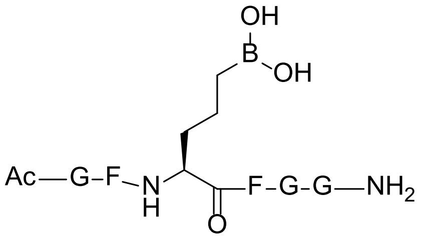

P1–B(OH)$_2$

Chemical Formula: $C_{31}H_{42}BN_7O_9$

Exact Mass: 667.31

Molecular Weight: 667.53

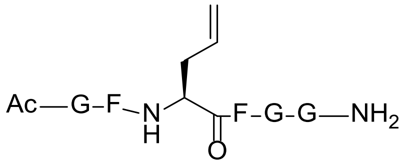

P1=

Chemical Formula: $C_{31}H_{39}N_7O_7$

Exact Mass: 621.29

Molecular Weight: 621.70

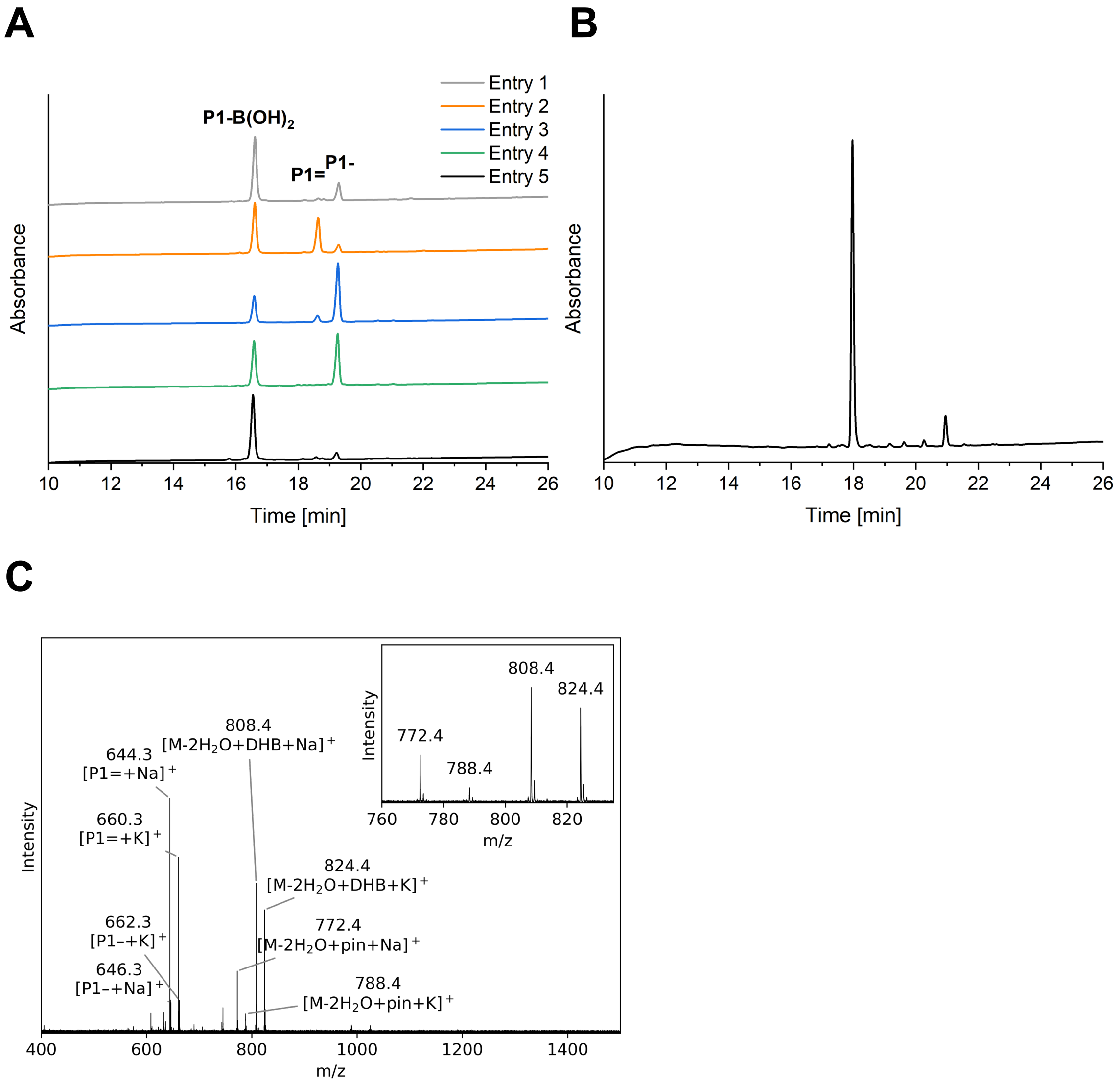

D

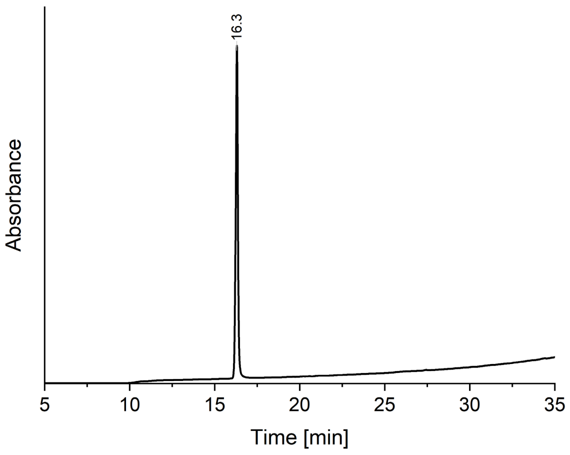

E

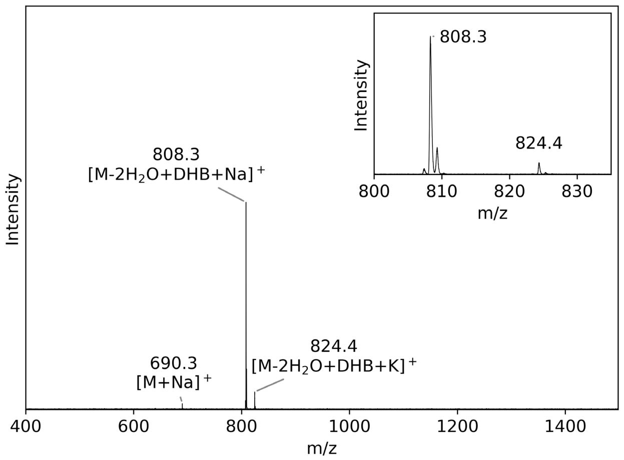

Figure S1: **P1–B(OH)$_2$** A) Analytical HPLC (220 nm, column A) of entry 1 to 5 referring to conditions of Table 1. B) Analytical HPLC (220 nm, column B) of entry 6. C) MALDI-TOF MS spectrum of crude peptide from entry 5. The formation of adducts with 2,5-dihydroxybenzoic acid (DHB) was observed, which is typical for boronic acids and described in the literature.[1] D) Analytical HPLC (220 nm, column A) of purified **P1–B(OH)$_2$** peptide. E) MALDI-TOF MS spectrum of purified **P1–B(OH)$_2$** peptide. Calculated mass: [**P1=**+Na]$^+$ 644.3, [**P1=**+K]$^+$ 660.3, [**P1–**+Na]$^+$ 646.3, [**P1–**+K]$^+$ 662.3, [M+Na]$^+$ 690.3, [M-2H$_2$O+pin+Na]$^+$ 772.4, [M-2H$_2$O+pin+K]$^+$ 788.4, [M-2H$_2$O+DHB+Na]$^+$ 808.3, [M-2H$_2$O+DHB+K]$^+$ 824.3.

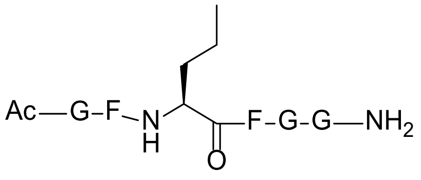

P1–

Chemical Formula: $C_{31}H_{41}N_7O_7$

Exact Mass: 623.31

Molecular Weight: 623.71

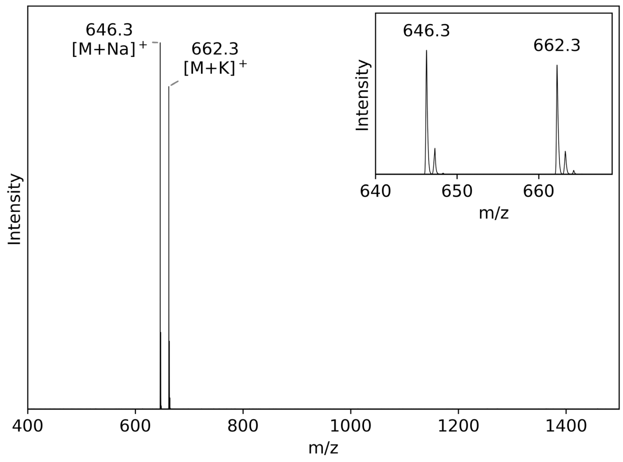

Figure 2: MALDI-TOF MS spectrum of purified **P1–** peptide. Calculated mass: [M+Na]$^+$ 646.3, [M+K]$^+$ 662.3.

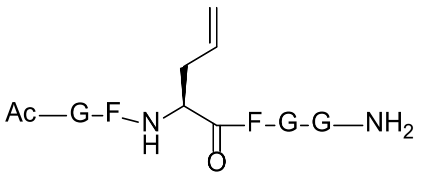

P1=

Chemical Formula: $C_{31}H_{39}N_7O_7$

Exact Mass: 621.29

Molecular Weight: 621.70

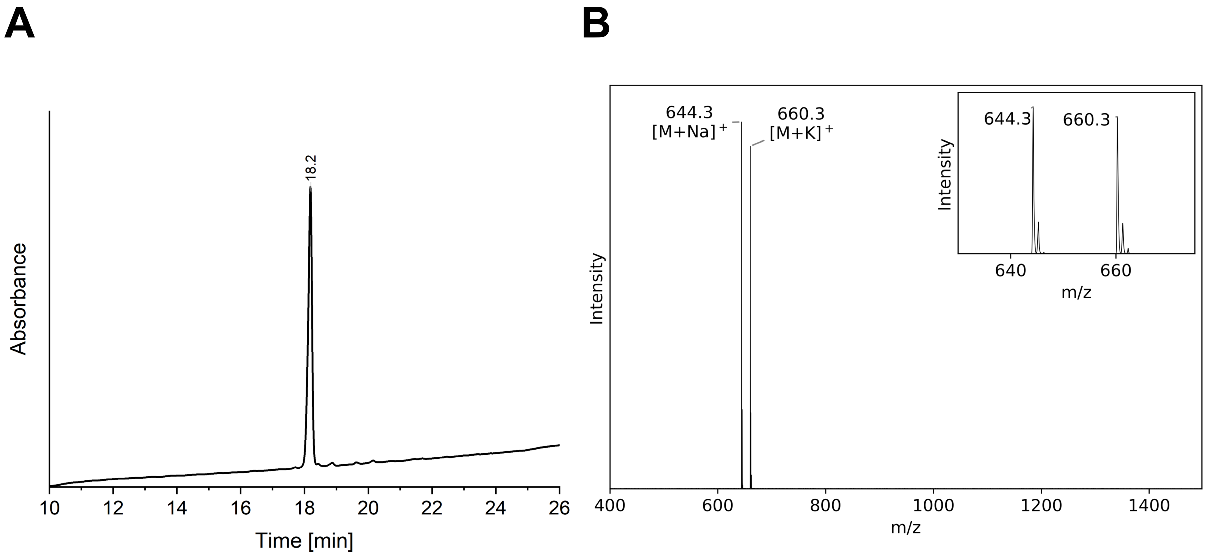

Figure S3: **P1=** A) Analytical HPLC (220 nm, column A) of crude **P1=** peptide. B) MALDI-TOF MS spectrum of crude **P1=** peptide. Calculated mass: $[M+Na]^+$ 644.3, $[M+K]^+$ 660.3.

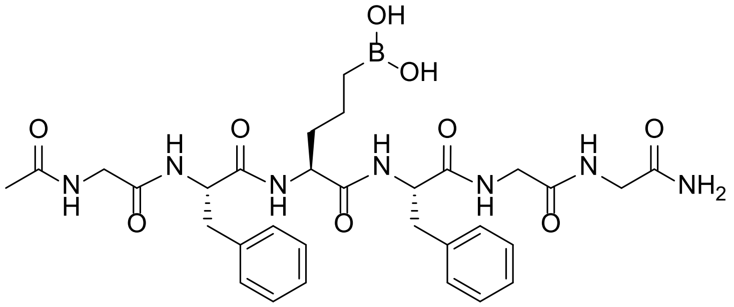

^{1}H-NMR (400 MHz, DMSO-d_6, 295 K):
δ [ppm] = 7.24 − 7.14 (m, 10H, H-Aryl Phe), 4.53 − 4.48 (m, 2H, α-CH Phe), 4.18 − 4.13 (m, 1H, α-CH X), 3.80 − 3.51 (m, 6H, CH_2 Gly), 3.07 − 2.94 (m, 2H, β-CH_2 Phe), 2.86 − 2.66 (m, 2H, β-CH_2 Phe), 1.81 (s, 3H, Ac), 1.59 – 1.40 (m, 2H, β-C<u>H</u>2-CH2-CH2-X), 1.38 – 1.17 (m, 2H, β-CH2-C<u>H</u>2-CH2-X), 0.68 – 0.49 (m, 2H, β-CH2-CH2-C<u>H</u>2-X)

A

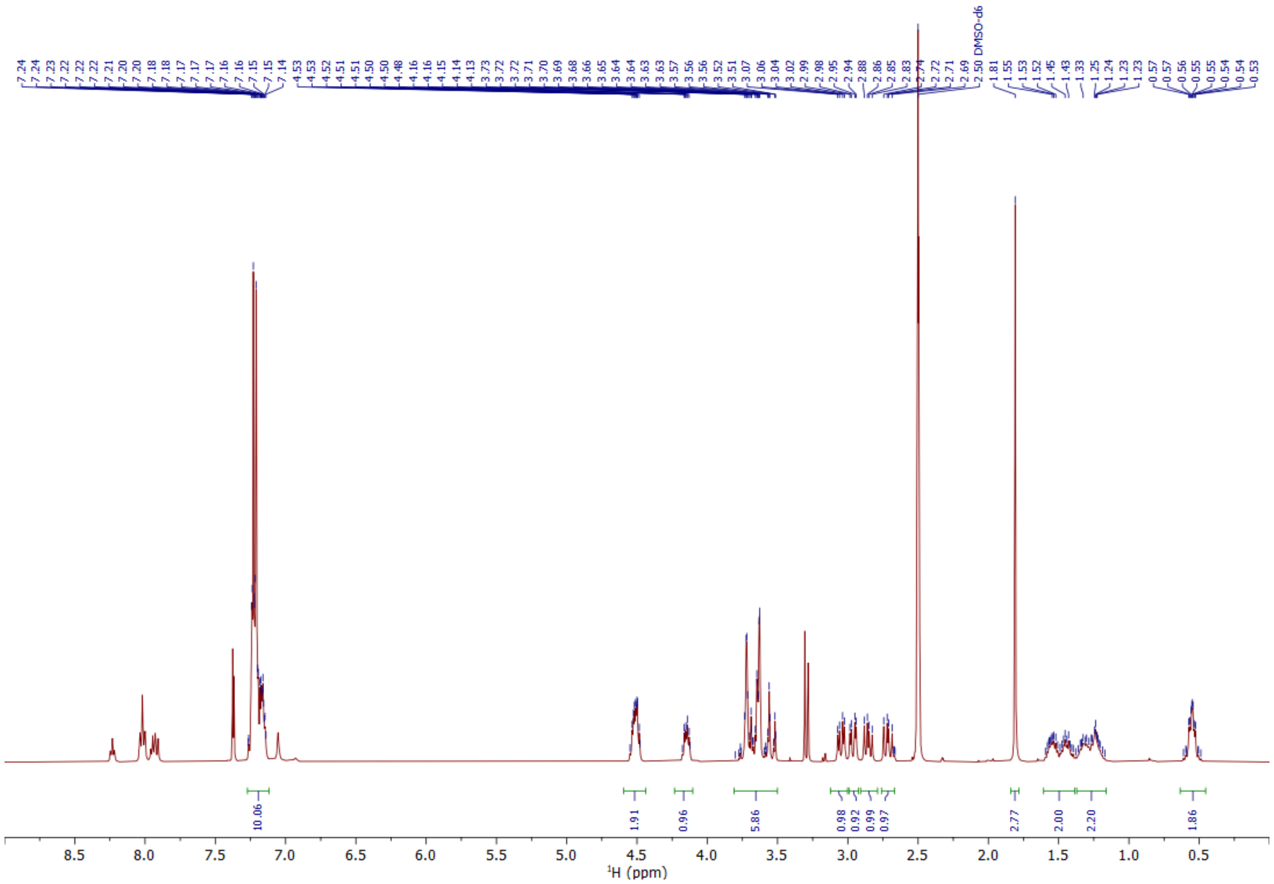

B

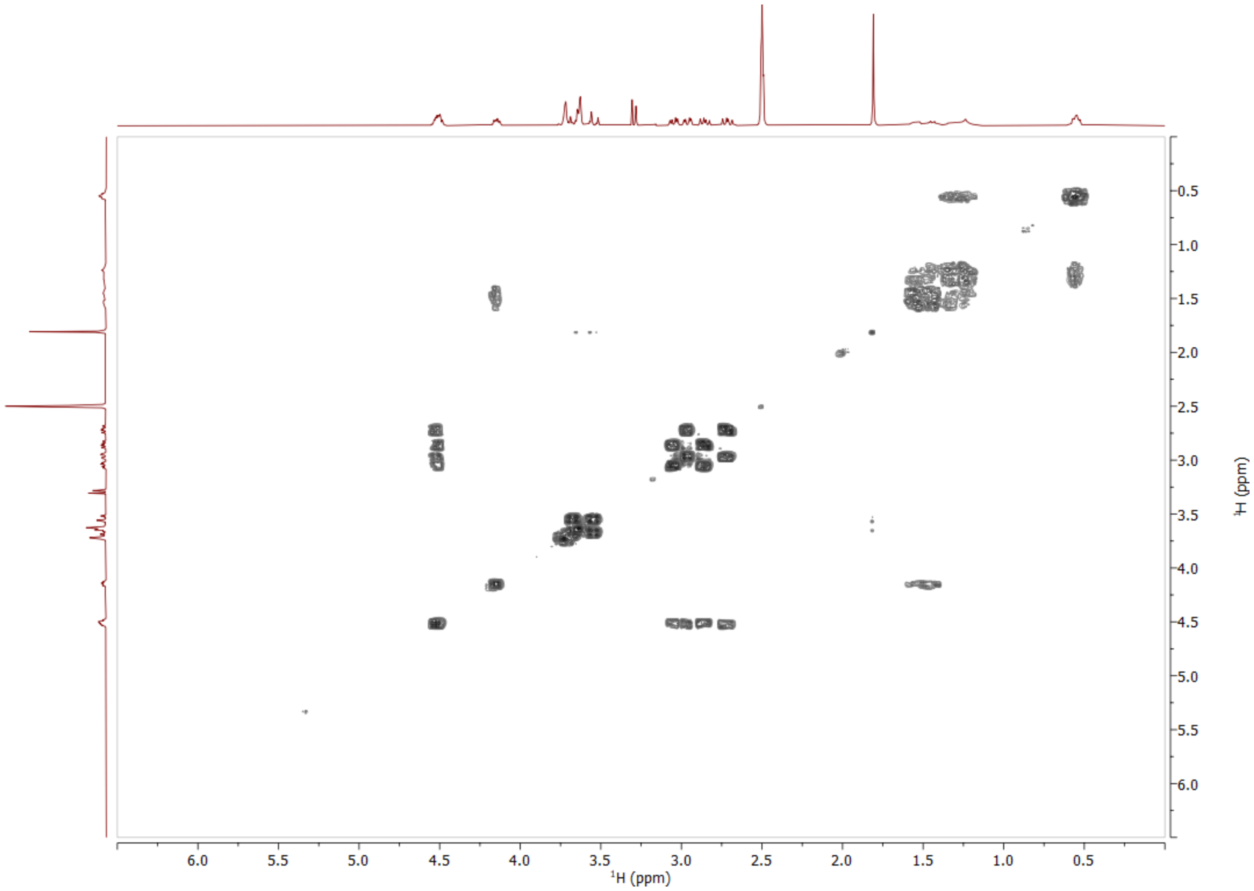

Figure S4: ^{1}H-NMR (400 MHz, DMSO-d$_6$, 295 K) (A) and ^{1}H-^{1}H-COSY (400 MHz, 400 MHz, DMSO-d$_6$, 295 K) (B) of **P1–B(OH)$_2$**. Peaks at 7.5-8.5 ppm are from incomplete hydrogen-deuterium exchange of peptide backbone amide protons.

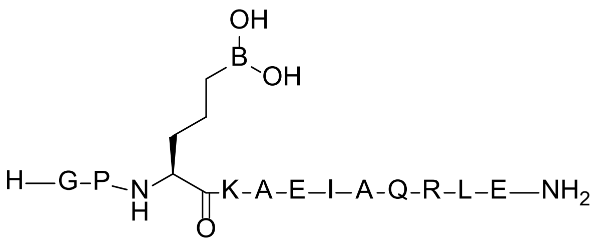

P2–B(OH)$_2$

Chemical Formula: $C_{57}H_{101}BN_{18}O_{19}$

Exact Mass: 1352.76

Molecular Weight: 1353.35

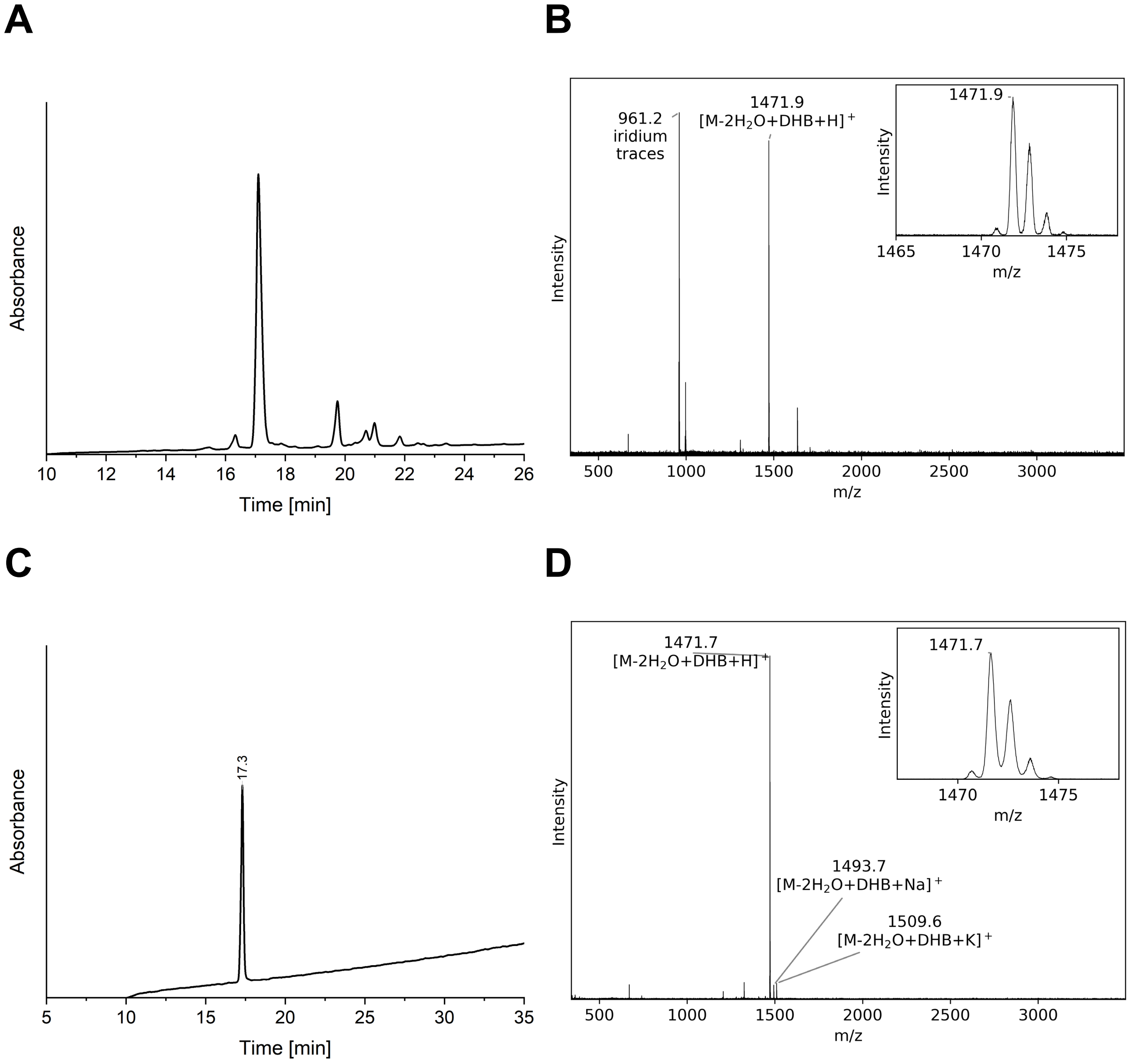

Figure S5: **P2–B(OH)$_2$** A) Analytical HPLC (220 nm, column A) of crude peptide. B) MALDI-TOF MS spectrum of crude peptide. C) Analytical HPLC (220 nm, column A) of purified peptide. D) MALDI-TOF MS spectrum of purified peptide. Calculated mass: $[M\text{-}2H_2O\text{+}DHB\text{+}H]^+$ 1471.8, $[M\text{-}2H_2O\text{+}DHB\text{+}Na]^+$ 1493.8, $[M\text{-}2H_2O\text{+}DHB\text{+}K]^+$ 1509.7.

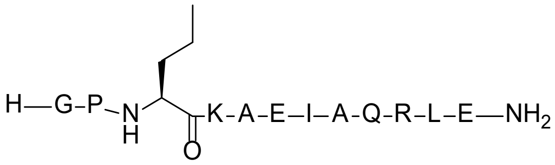

P2–

Chemical Formula: $C_{57}H_{100}N_{18}O_{17}$
Exact Mass: 1308.75
Molecular Weight: 1309.54

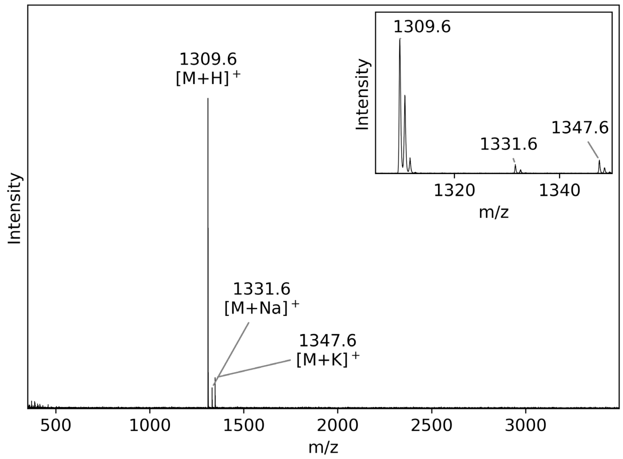

Figure S6: MALDI-TOF MS spectrum of purified **P2–** peptide. Calculated mass: $[M+H]^+$ 1309.8, $[M+Na]^+$ 1331.7, $[M+K]^+$ 1347.7.

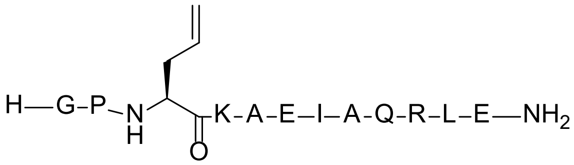

P2=

Chemical Formula: $C_{57}H_{98}N_{18}O_{17}$
Exact Mass: 1306.74
Molecular Weight: 1307.52

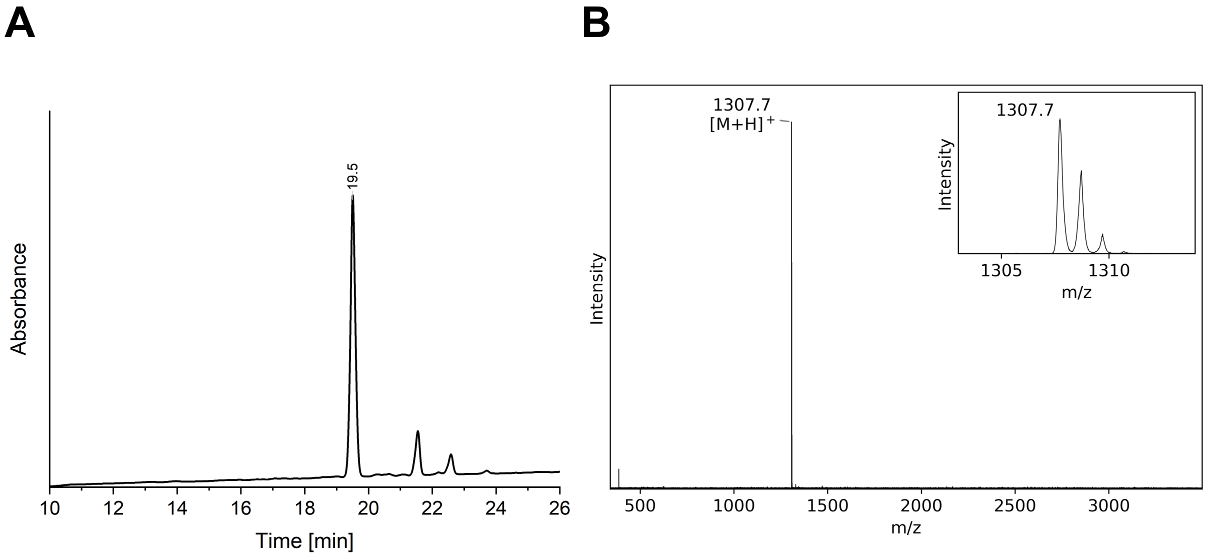

Figure S7: **P2=** A) Analytical HPLC (220 nm, column A) of crude peptide. B) MALDI-TOF MS spectrum of crude peptide. Calculated mass: $[M+H]^+$ 1307.7.

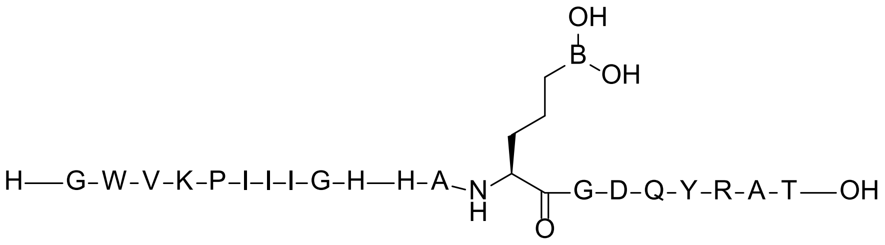

P3–B(OH)$_2$

Chemical Formula: $C_{102}H_{157}BN_{30}O_{28}$

Exact Mass: 2261.19

Molecular Weight: 2262.37

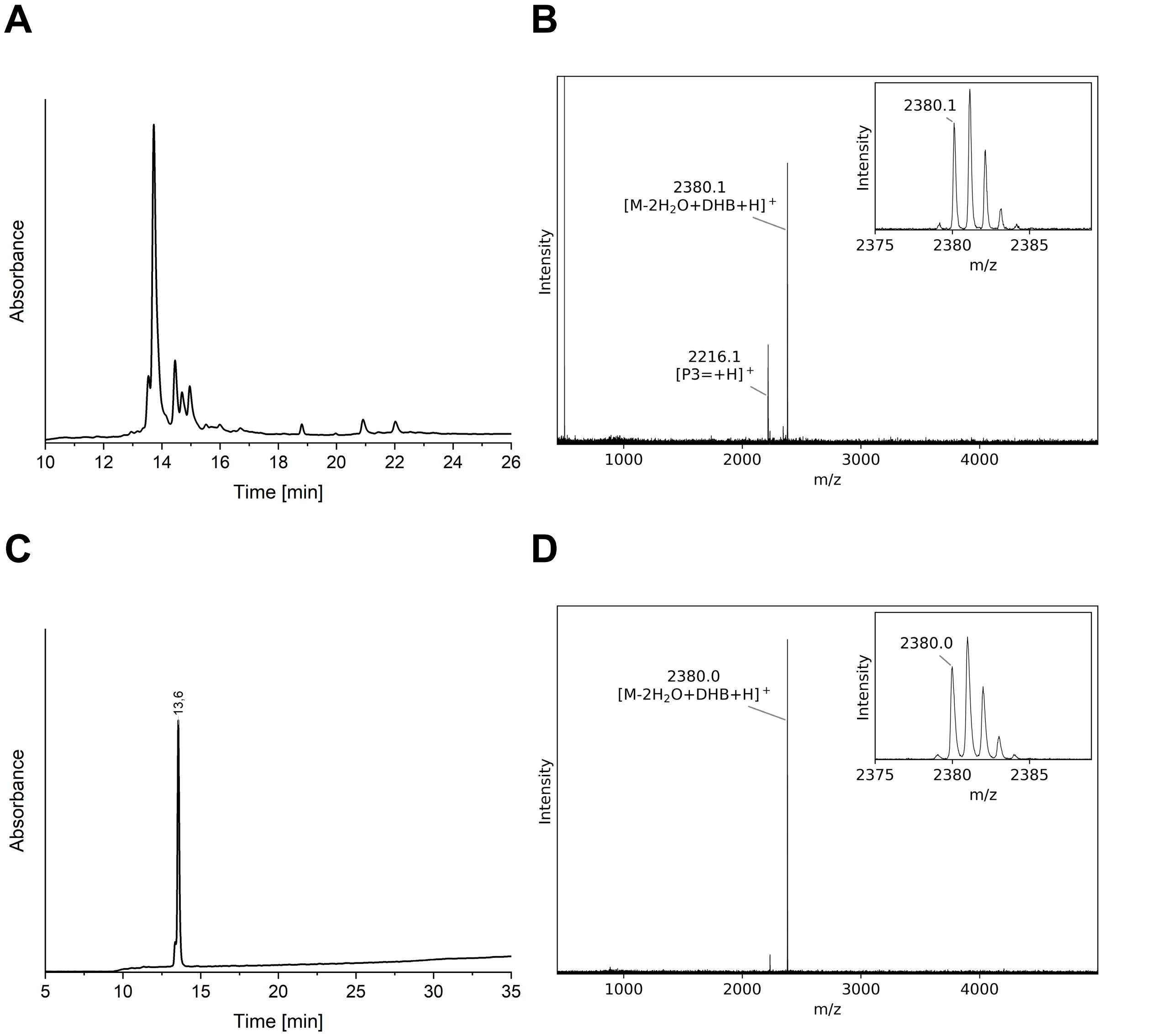

Figure S8: **P3–B(OH)$_2$** A) Analytical HPLC (220 nm, column B) of crude peptide. B) MALDI-TOF MS spectrum of crude peptide. C) Analytical HPLC (220 nm, column B) of purified peptide. D) MALDI-TOF MS spectrum of purified peptide. Calculated mass: [**P3=**+H]$^+$ 2216.2, [M-2H_2O+DHB+H]$^+$ 2380.2.

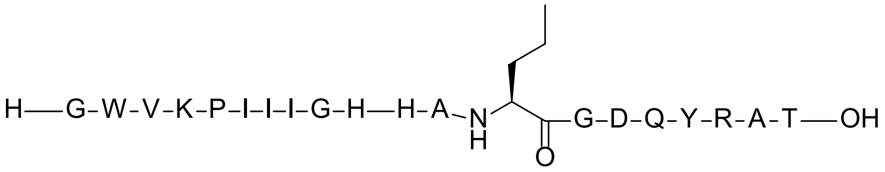

P3–

Chemical Formula: $C_{102}H_{156}N_{30}O_{26}$
Exact Mass: 2217.18
Molecular Weight: 2218.55

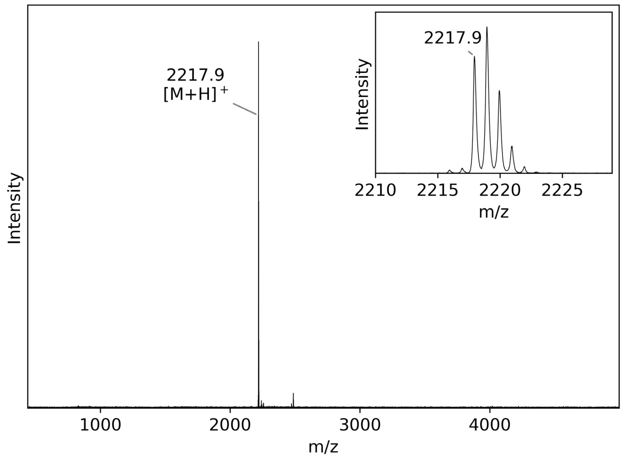

Figure S9: MALDI-TOF MS spectrum of purified **P3–**. Calculated mass: $[M+H]^+$ 2218.2.

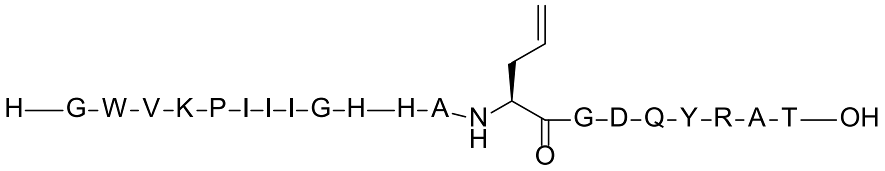

P3=

Chemical Formula: $C_{102}H_{154}N_{30}O_{26}$
Exact Mass: 2215.17
Molecular Weight: 2216.54

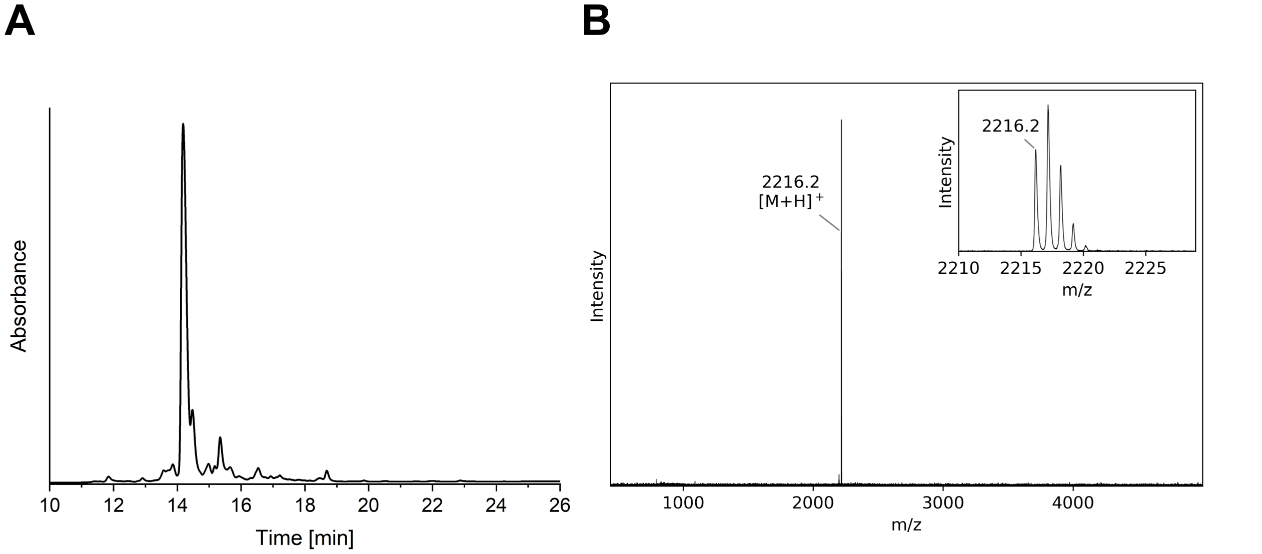

Figure S10: **P3=** A) Analytical HPLC (220 nm, column B) of crude peptide. B) MALDI-TOF MS spectrum of crude peptide. Calculated mass: $[M+H]^+$ 2216.2.

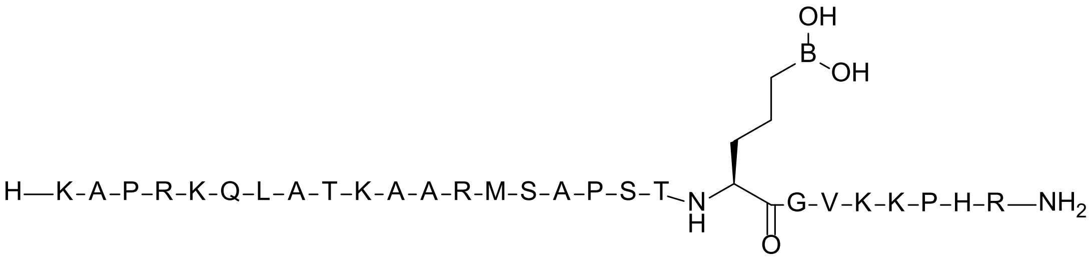

P4–B(OH)$_2$

Chemical Formula: $C_{126}H_{226}BN_{45}O_{34}S$

Exact Mass: 2956.72

Molecular Weight: 2958.35

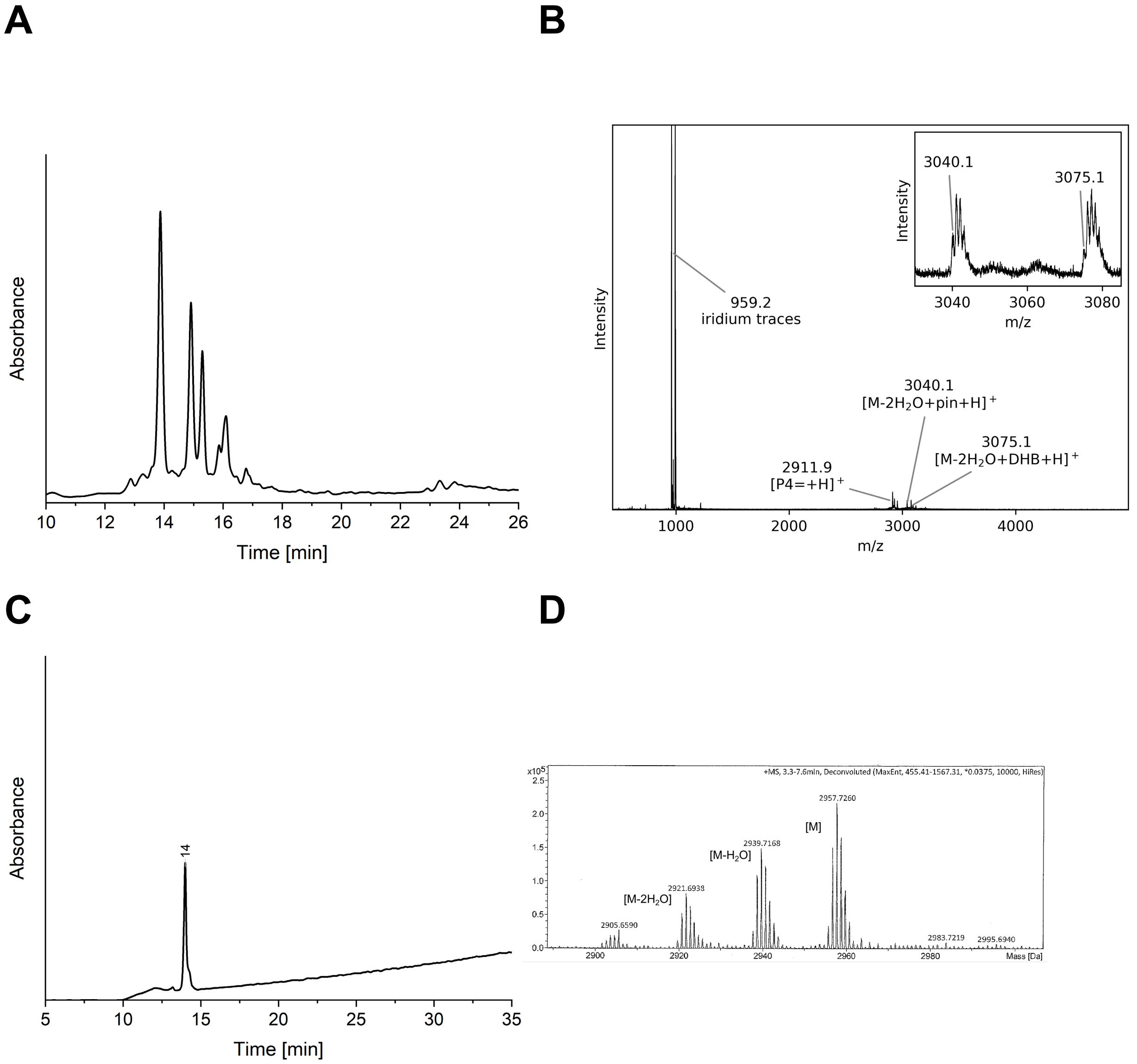

Figure S11: **P4–B(OH)$_2$** A) Analytical HPLC (220 nm, column A) of crude peptide. B) MALDI-TOF MS spectrum of crude peptide. C) Analytical HPLC (220 nm, column A) of purified peptide. D) ESI HR-MS spectrum of purified peptide. Full HR-MS spectrum in section 3. Calculated mass: [**P4=**+H]$^+$ 2911.7, [M-2H$_2$O] 2920.7, [M-H$_2$O] 2938.7, [M] 2956.7, [M-2H$_2$O+pin+H]$^+$ 3039.8, [M-2H$_2$O+DHB+H]$^+$ 3075.7.

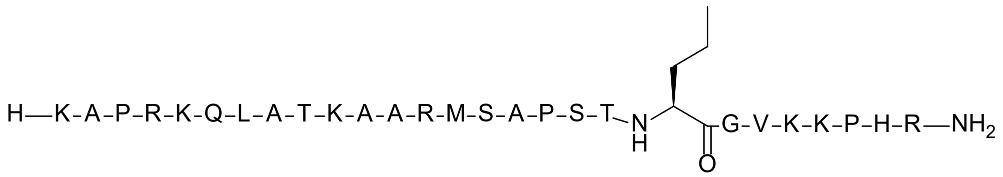

P4–

Chemical Formula: $C_{126}H_{225}N_{45}O_{32}S$
Exact Mass: 2912.71
Molecular Weight: 2914.53

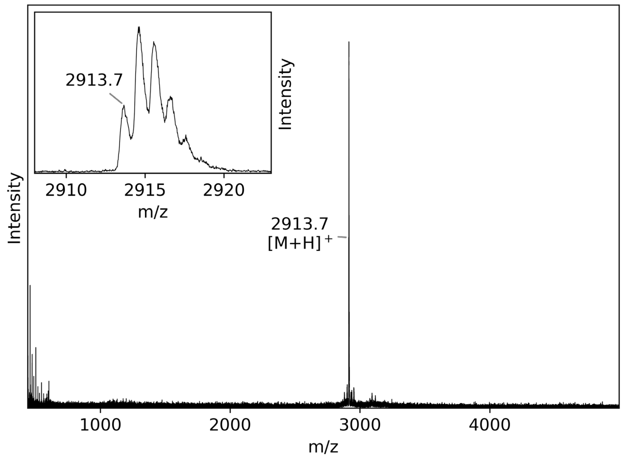

Figure S12: MALDI-TOF MS spectrum of purified **P4–** peptide. Calculated mass: $[M+H]^+$ 2913.7.

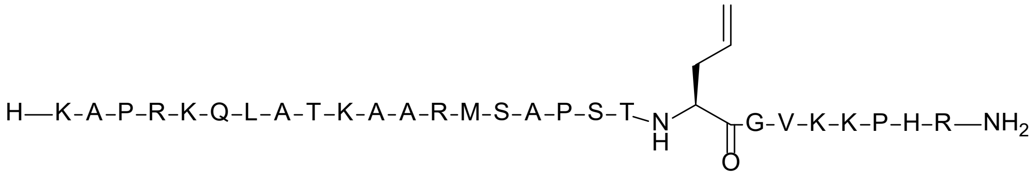

P4=

Chemical Formula: $C_{126}H_{223}N_{45}O_{32}S$
Exact Mass: 2910.69
Molecular Weight: 2912.51

A **B**

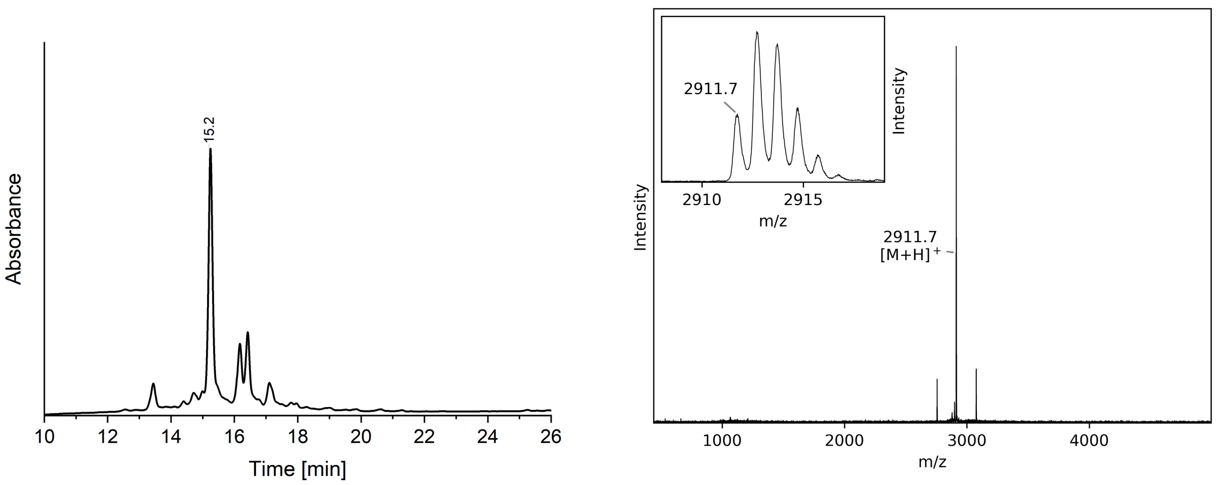

Figure S13: **P4=** A) Analytical HPLC (220 nm, column A) of crude peptide. B) MALDI-TOF MS spectrum of crude peptide. Calculated mass: $[M+H]^+$ 2911.7.

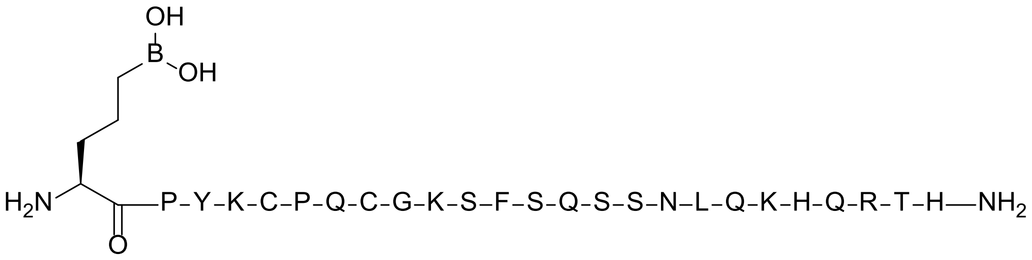

P5–B(OH)$_2$

Chemical Formula: $C_{123}H_{196}BN_{41}O_{38}S_2$

Exact Mass: 2930.42

Molecular Weight: 2932.10

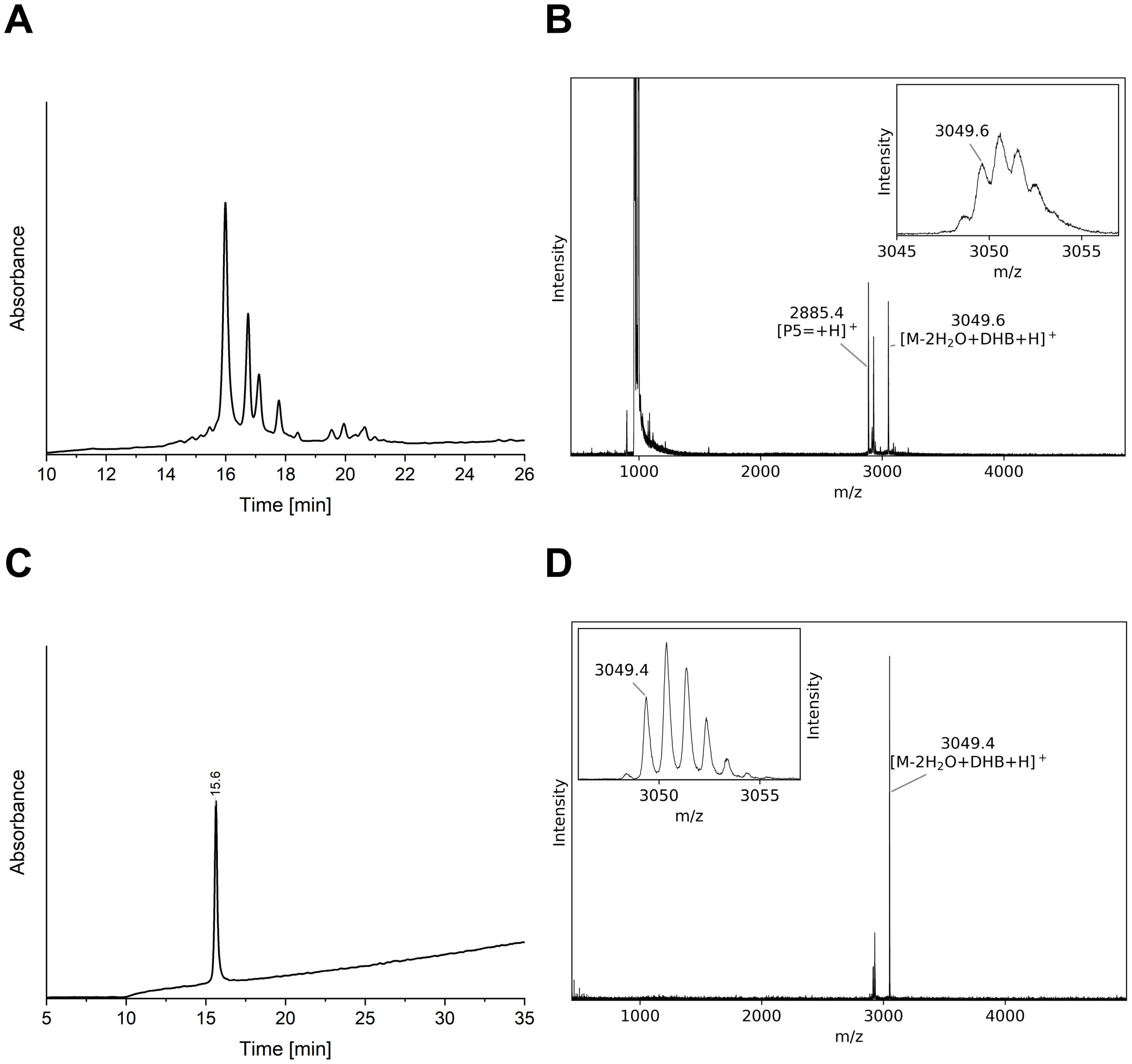

Figure S14: **P5–B(OH)$_2$** A) Analytical HPLC (220 nm, column A) of crude peptide. B) MALDI-TOF MS spectrum of crude peptide. C) Analytical HPLC (220 nm, column A) of purified peptide. D) MALDI-TOF MS spectrum of purified peptide. Calculated mass: [**P5=**+H]$^+$ 2885.4, [M-2H$_2$O+DHB+H]$^+$ 3049.4.

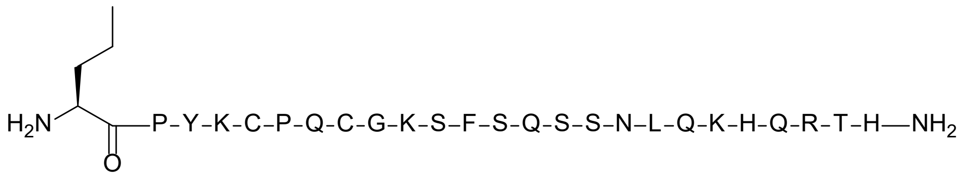

P5–

Chemical Formula: $C_{123}H_{195}N_{41}O_{36}S_2$

Exact Mass: 2886.41

Molecular Weight: 2888.28

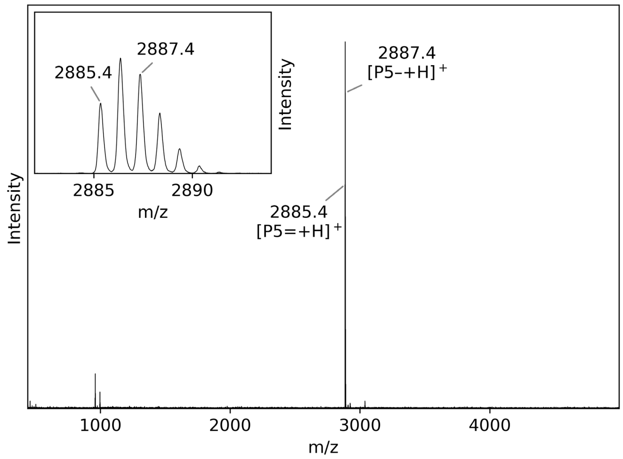

Figure S15: MALDI-TOF MS spectrum of purified **P5–** and **P5=** mixture. Calculated mass: [**P5=**+H]$^+$ 2885.4, [**P5–**+H]$^+$ 2887.4.

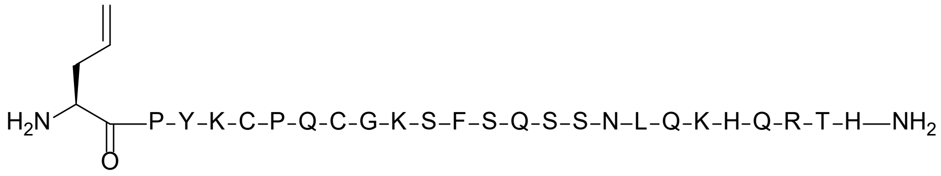

P5=

Chemical Formula: $C_{123}H_{193}N_{41}O_{36}S_2$

Exact Mass: 2884.40

Molecular Weight: 2886.27

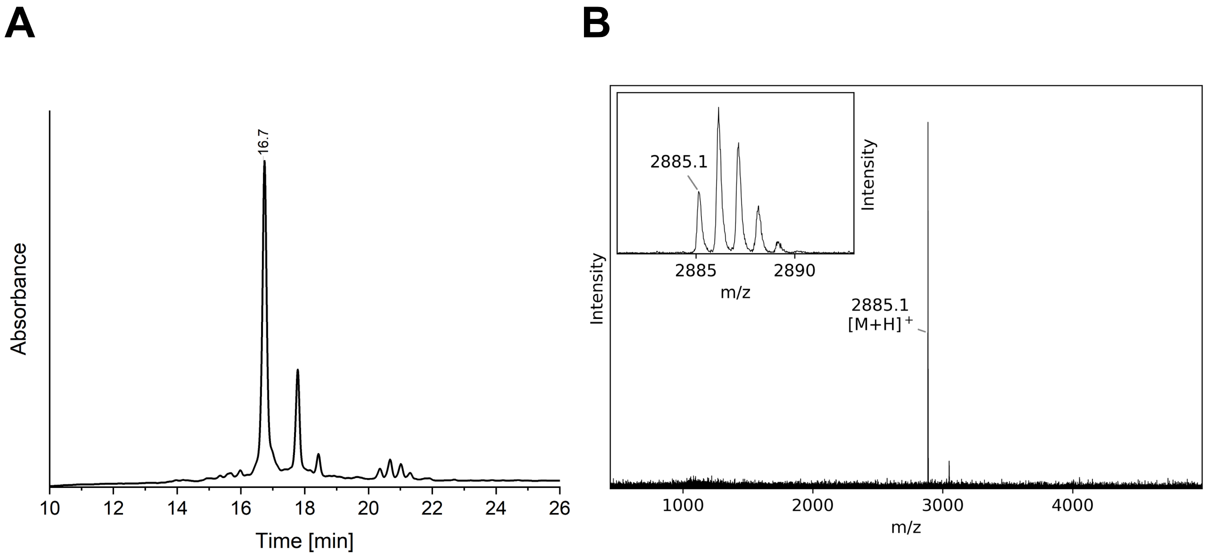

Figure S16: **P5=** A) Analytical HPLC (220 nm, column A) of crude peptide. B) MALDI-TOF MS spectrum of crude peptide. Calculated mass: [M+H]$^+$ 2885.4.

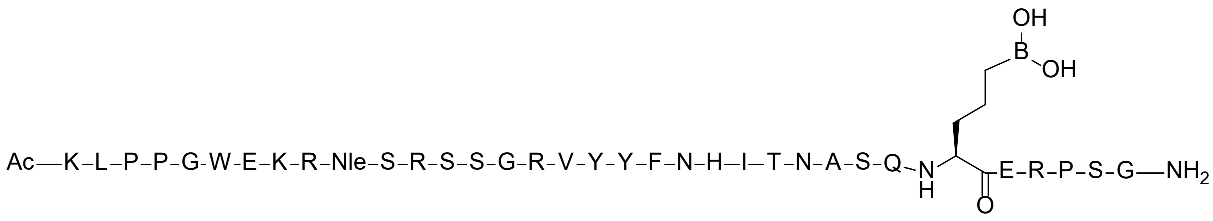

P6–B(OH)$_2$

Chemical Formula: $C_{176}H_{274}BN_{55}O_{52}$

Exact Mass: 4001.06

Molecular Weight: 4003.27

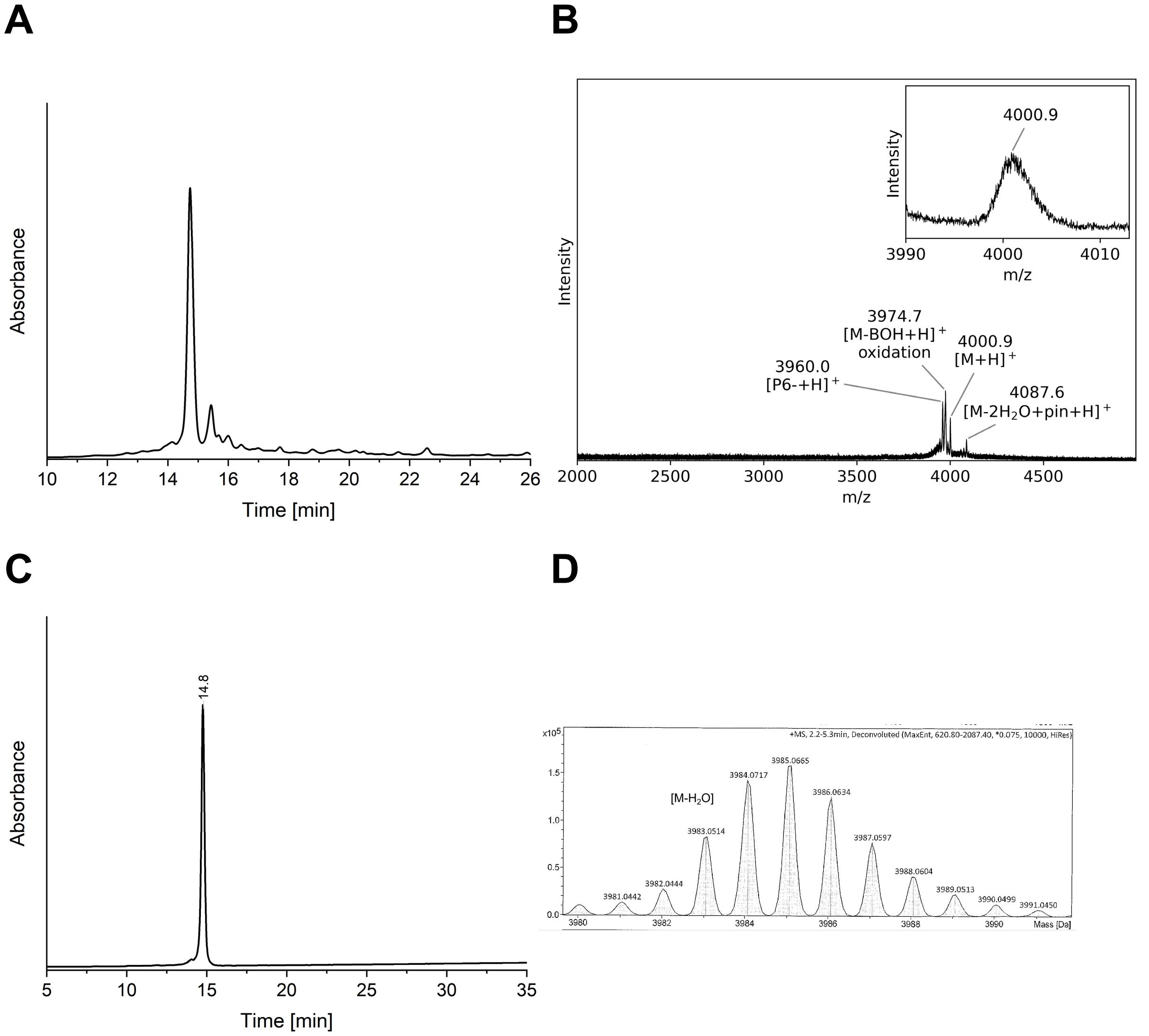

Figure S17: **P6–B(OH)$_2$** A) Analytical HPLC (220 nm, column A) of crude peptide. B) MALDI-TOF MS spectrum of crude peptide. C) Analytical HPLC (220 nm, column A) of purified peptide. D) ESI HR-MS spectrum of purified peptide. Full HR-MS spectrum in section 3. Calculated mass: [**P6–**+H]$^+$ 3958.1, [M-BOH+H]$^+$ 3974.1, [M-H$_2$O] 3983.1, [M+H]$^+$ 4002.1, [M-2H$_2$O+pin+H]$^+$ 4084.1.

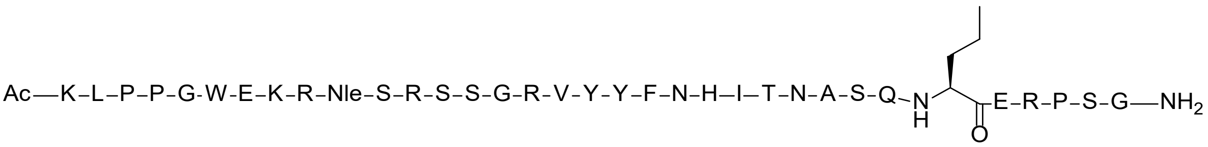

P6–

Chemical Formula: $C_{176}H_{273}N_{55}O_{50}$

Exact Mass: 3957.05

Molecular Weight: 3959.46

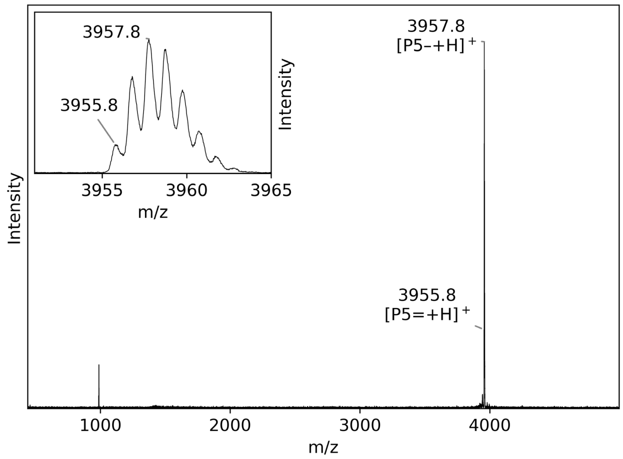

Figure S18: MALDI-TOF MS spectrum of purified **P6–** and **P6=** mixture. Calculated mass: [**P6=**+H]$^+$ 3956.0, [**P6–**+H]$^+$ 3958.1.

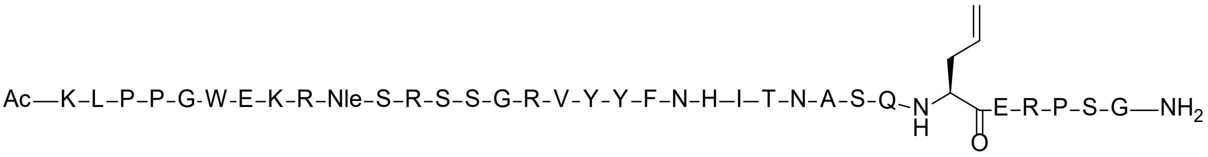

P6=

Chemical Formula: $C_{176}H_{271}N_{55}O_{50}$

Exact Mass: 3955.04

Molecular Weight: 3957.44

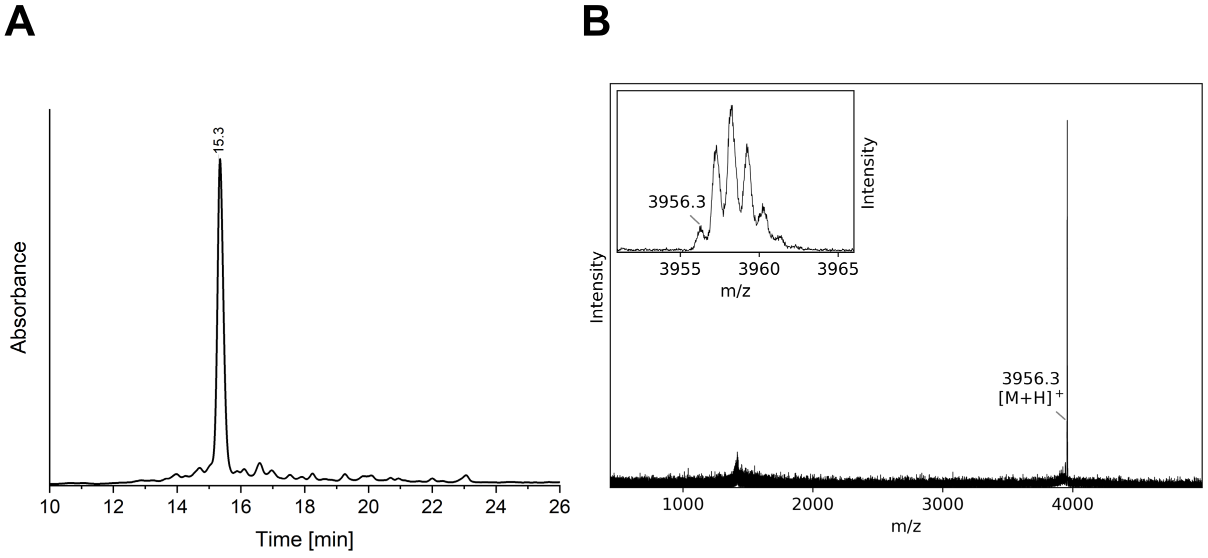

Figure S19: **P6=** A) Analytical HPLC (220 nm, column A) of crude peptide. B) MALDI-TOF MS spectrum of crude peptide. [**P6=**+H]$^+$ 3956.0.

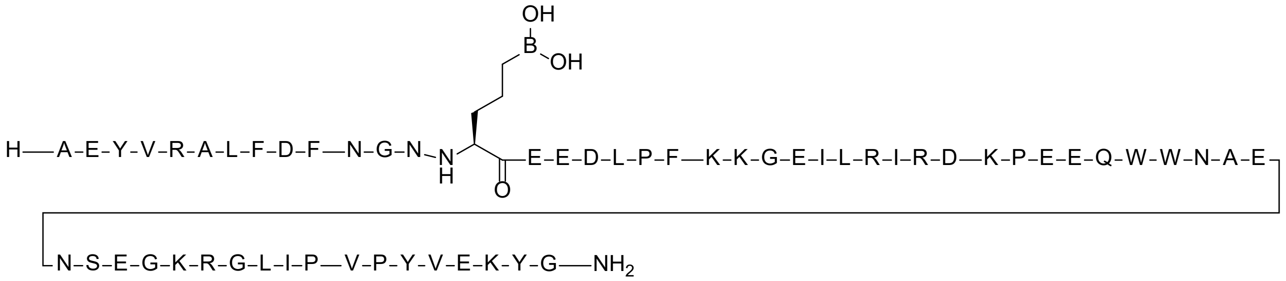

P7–B(OH)$_2$

Chemical Formula: $C_{312}H_{472}BN_{83}O_{93}$

Exact Mass: 6880.48

Molecular Weight: 6884.51

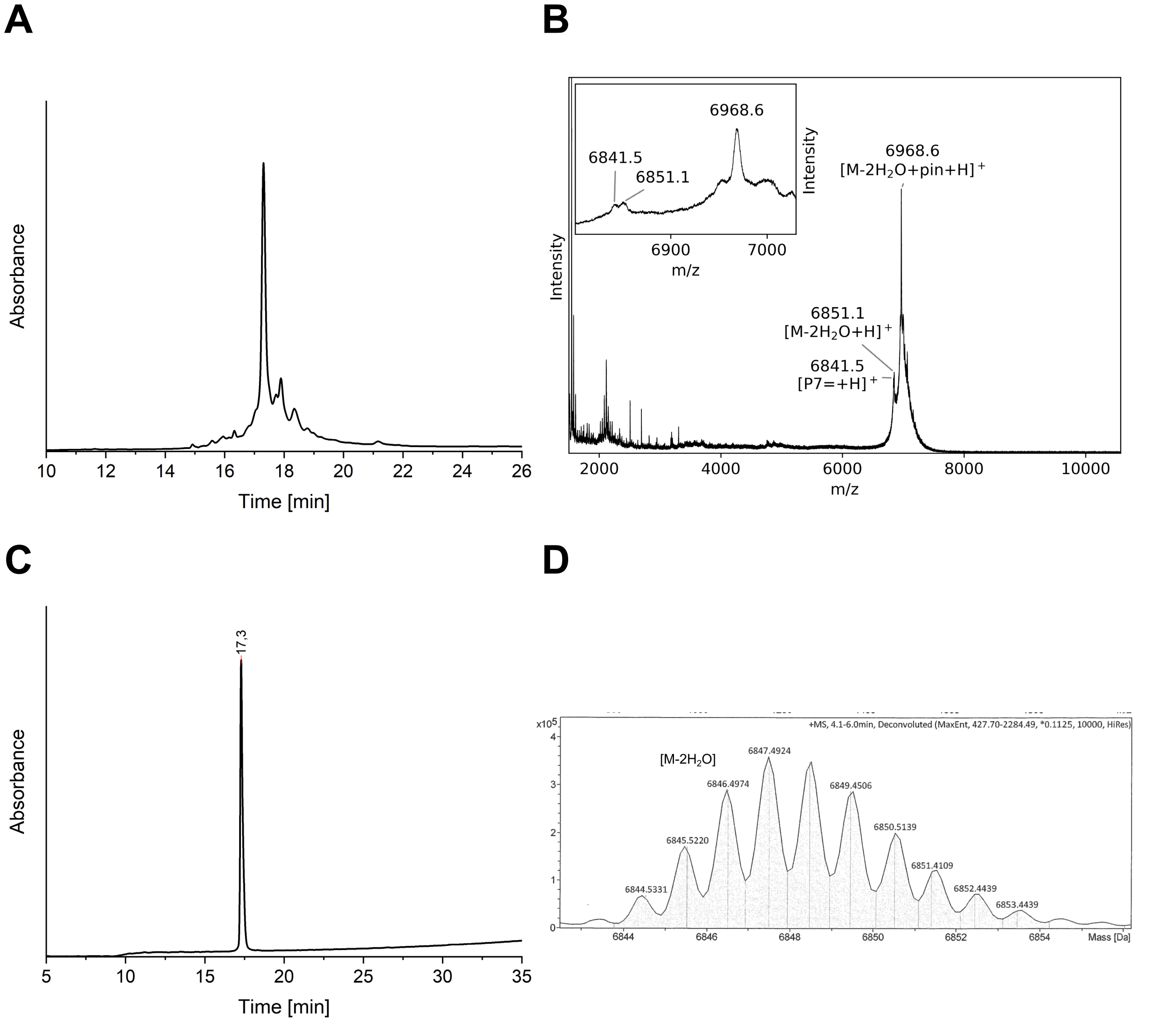

Figure S20: **P7–B(OH)$_2$** A) Analytical HPLC (220 nm, column B) of crude peptide. B) MALDI-TOF MS spectrum of crude peptide. C) Analytical HPLC (220 nm, column B) of purified peptide. D) MALDI-TOF MS spectrum of purified peptide. Calculated mass: [**P7=**+H]$^+$ 6835.5, [M-2H$_2$O]$^+$ 6845.5, [M-2H$_2$O+pin+H]$^+$ 6963.6.

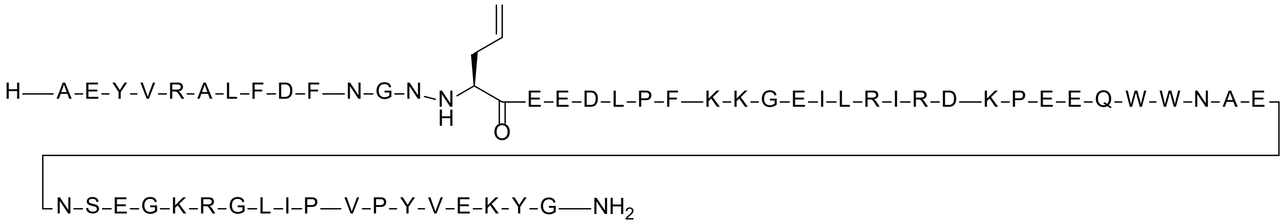

P7=

Chemical Formula: $C_{312}H_{469}N_{83}O_{91}$

Exact Mass: 6834.46

Molecular Weight: 6838.67

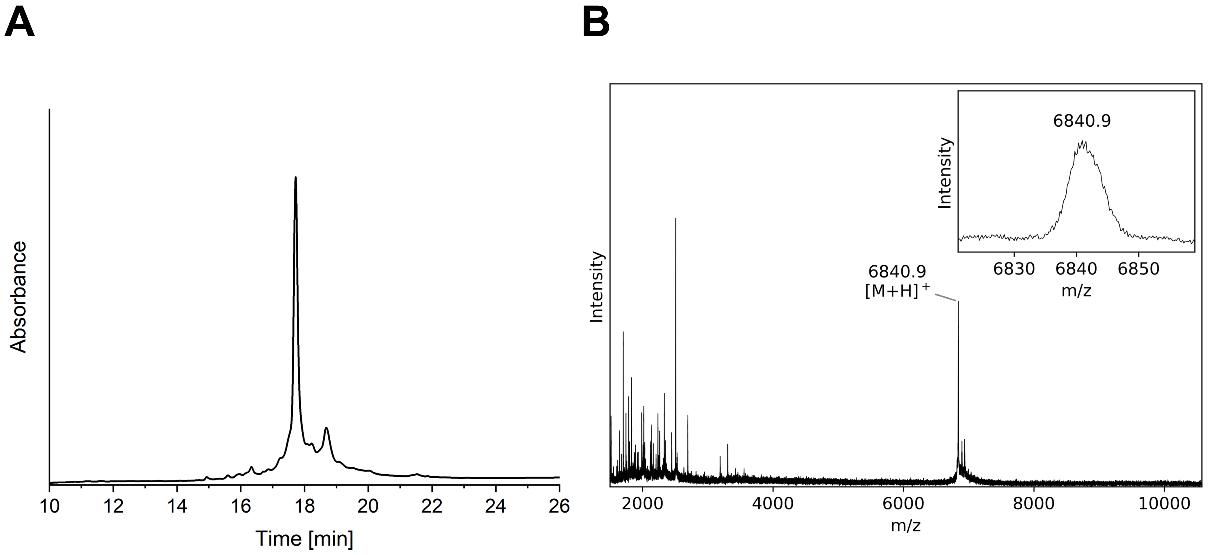

Figure S21: **P7=** A) Analytical HPLC (220 nm, column B) of crude peptide. B) MALDI-TOF MS spectrum of crude peptide. Calculated mass: $[M+H]^+$ 6835.5.

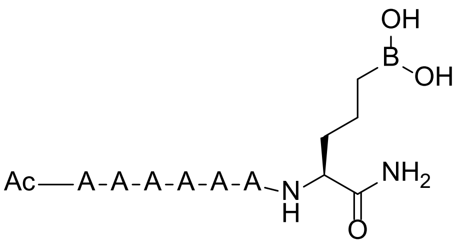

P8–B(OH)$_2$

Chemical Formula: $C_{25}H_{45}BN_8O_{10}$

Exact Mass: 628.34

Molecular Weight: 628.49

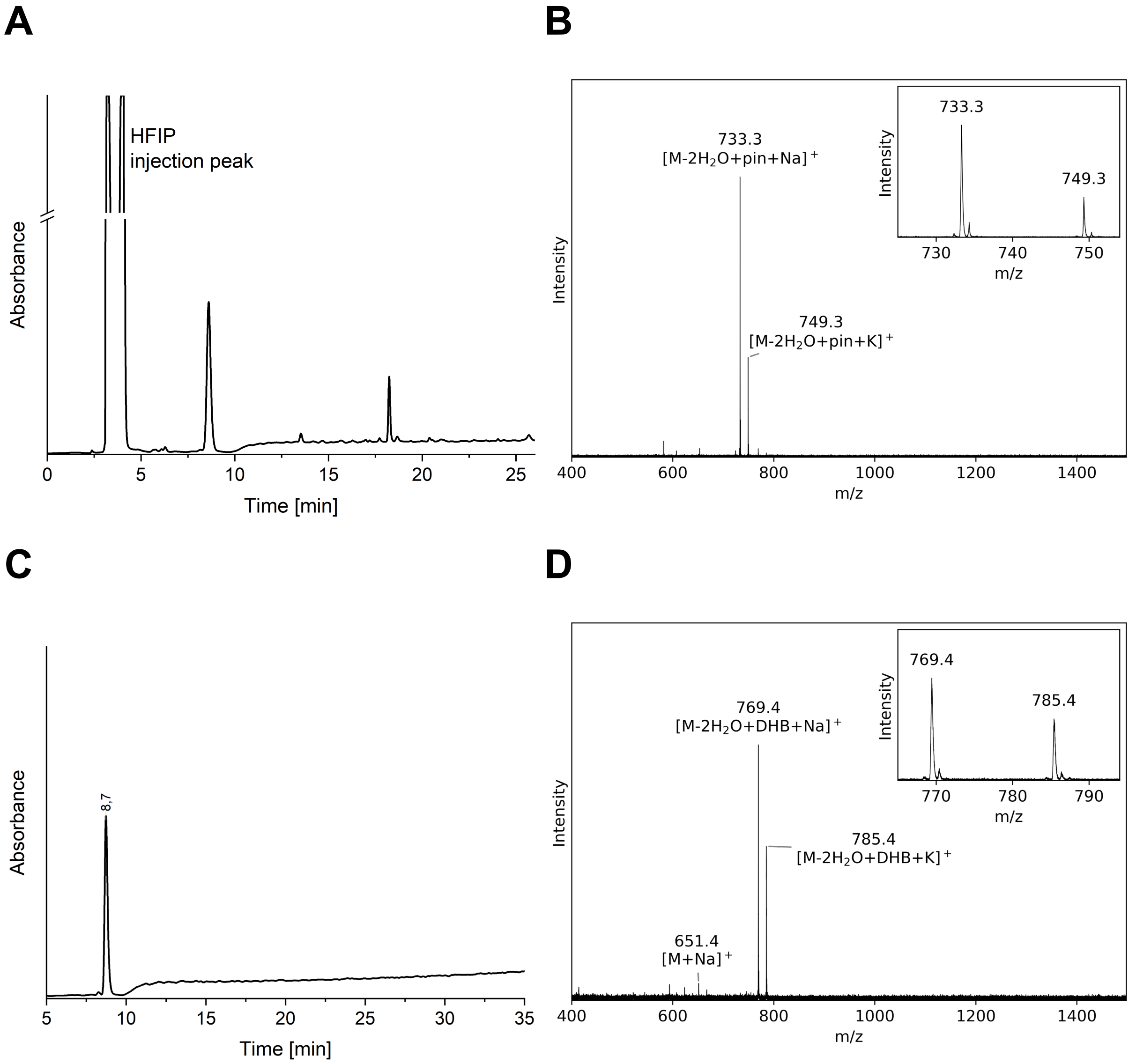

Figure S22: **P8–B(OH)$_2$** A) Analytical HPLC (220 nm, column B) of crude peptide. B) MALDI-TOF MS spectrum of crude peptide. C) Analytical HPLC (220 nm, column B) of purified peptide. D) MALDI-TOF MS spectrum of purified peptide. Calculated mass: $[M+Na]^+$ 651.3, $[M-2H_2O+pin+Na]^+$ 733.4, $[M-2H_2O+pin+K]^+$ 749.4, $[M-2H_2O+DHB+Na]^+$ 769.3, $[M-2H_2O+DHB+K]^+$ 785.3.

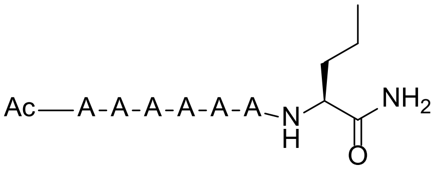

P8–

Chemical Formula: $C_{25}H_{44}N_8O_8$
Exact Mass: 584.33
Molecular Weight: 584.67

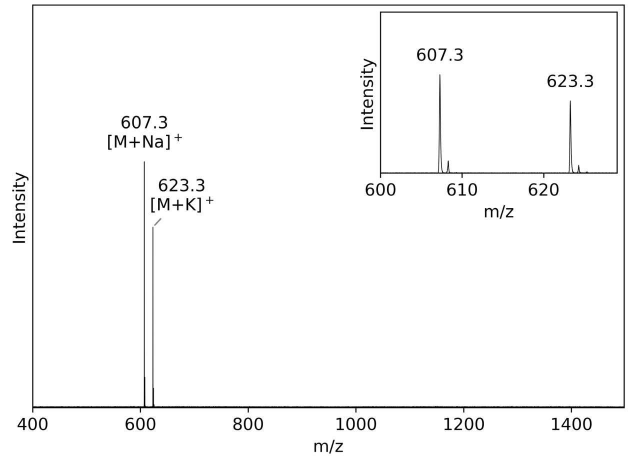

Figure S23: MALDI-TOF MS spectrum of purified **P8–**. Calculated mass: $[M+Na]^+$ 607.3, $[M+K]^+$ 623.3.

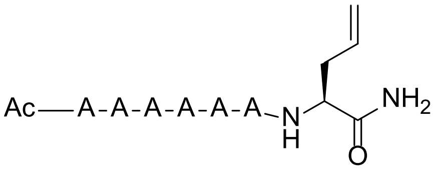

P8=

Chemical Formula: $C_{25}H_{42}N_8O_8$
Exact Mass: 582.31
Molecular Weight: 582.66

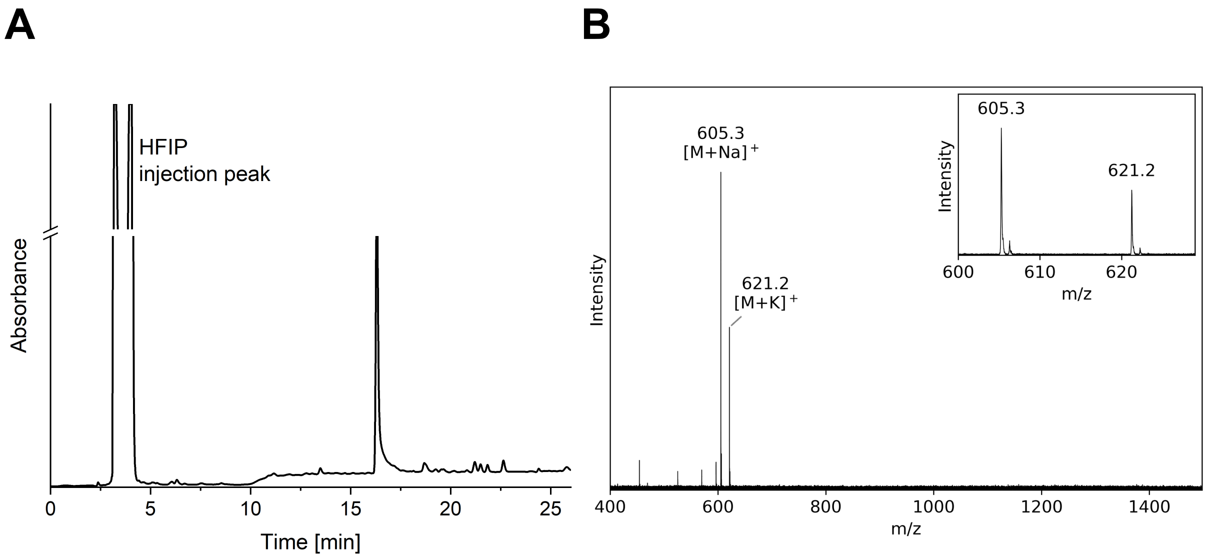

Figure S24: **P8=** A) Analytical HPLC (220 nm, column B) of crude peptide. B) MALDI-TOF MS spectrum of crude peptide. Calculated mass: $[M+Na]^+$ 605.3, $[M+K]^+$ 621.3.

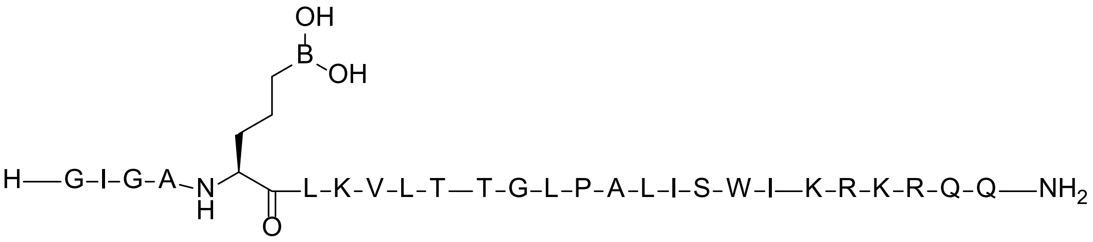

P9–B(OH)$_2$

Chemical Formula: $C_{131}H_{230}BN_{39}O_{33}$

Exact Mass: 2888.76

Molecular Weight: 2890.33

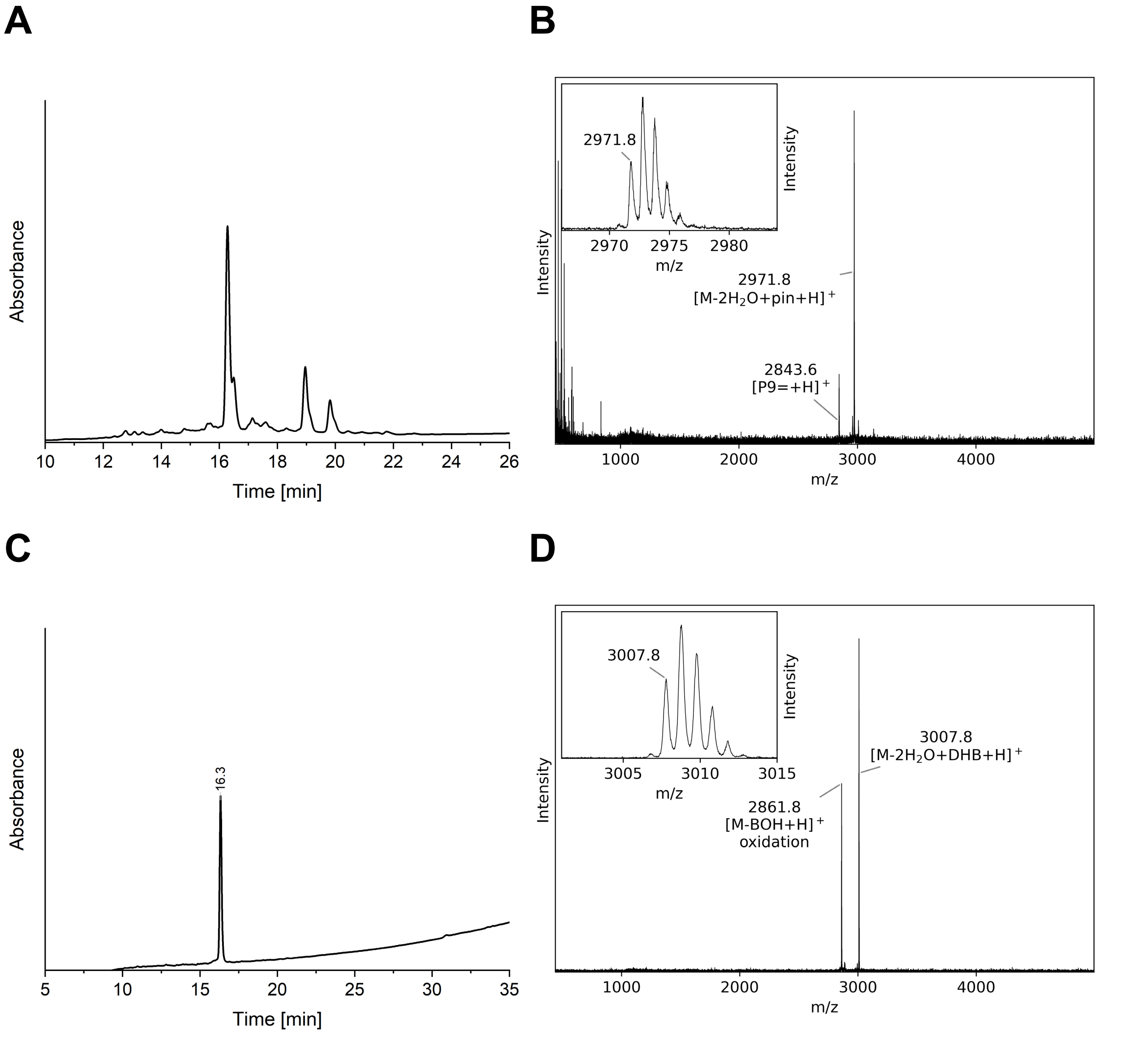

Figure S25: **P9–B(OH)$_2$** A) Analytical HPLC (220 nm, column B) of crude peptide. B) MALDI-TOF MS spectrum of crude peptide. C) Analytical HPLC (220 nm, column B) of purified peptide. D) MALDI-TOF MS spectrum of purified peptide. Calculated mass: [**P9=**+H]$^+$ 2843.6, [M-BOH+H]$^+$ 2861.8, [M-2H$_2$O+pin+H]$^+$ 2971.8, [M-2H$_2$O+DHB+H]$^+$ 3007.8.

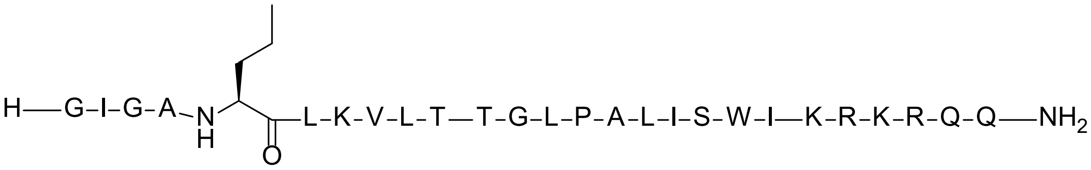

P9–

Chemical Formula: $C_{131}H_{229}N_{39}O_{31}$
Exact Mass: 2844.75
Molecular Weight: 2846.52

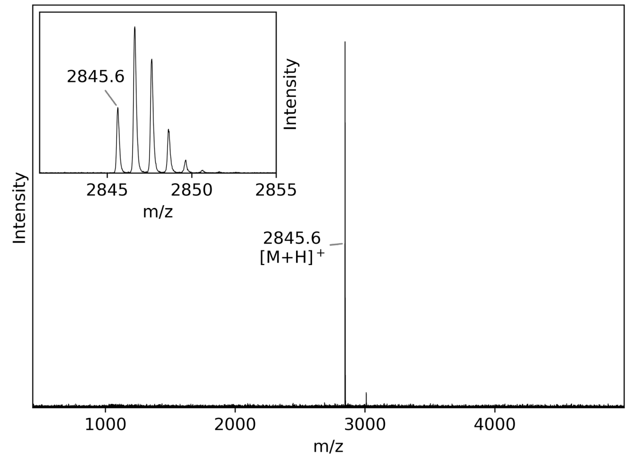

Figure S26: MALDI-TOF MS spectrum of purified **P9–**. Calculated mass: $[M+H]^+$ 2845.8.

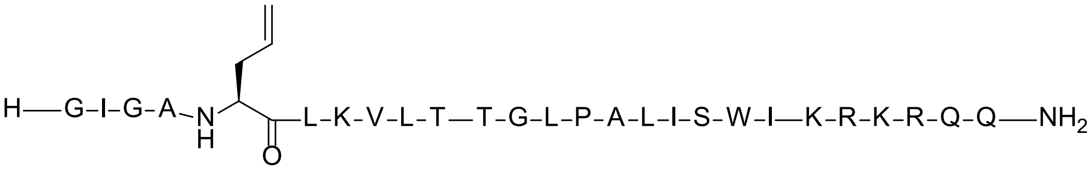

P9=

Chemical Formula: $C_{131}H_{227}N_{39}O_{31}$
Exact Mass: 2842.74
Molecular Weight: 2844.50

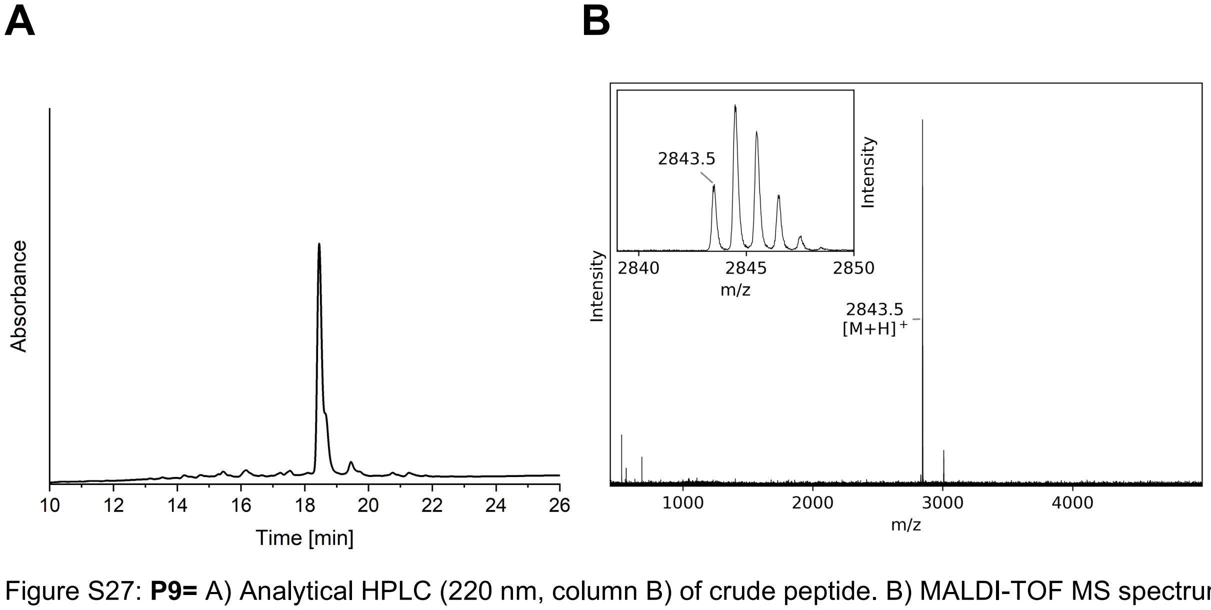

Figure S27: **P9=** A) Analytical HPLC (220 nm, column B) of crude peptide. B) MALDI-TOF MS spectrum of crude peptide. Calculated mass: $[M+H]^+$ 2843.6.

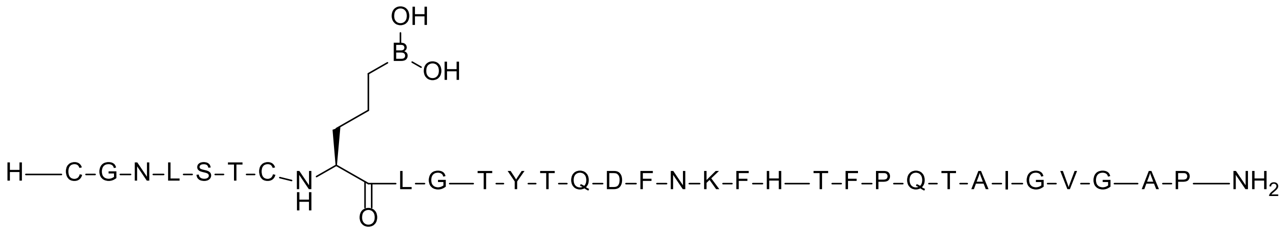

P10–B(OH)$_2$

Chemical Formula: $C_{151}H_{229}BN_{40}O_{47}S_2$

Exact Mass: 3429.63

Molecular Weight: 3431.66

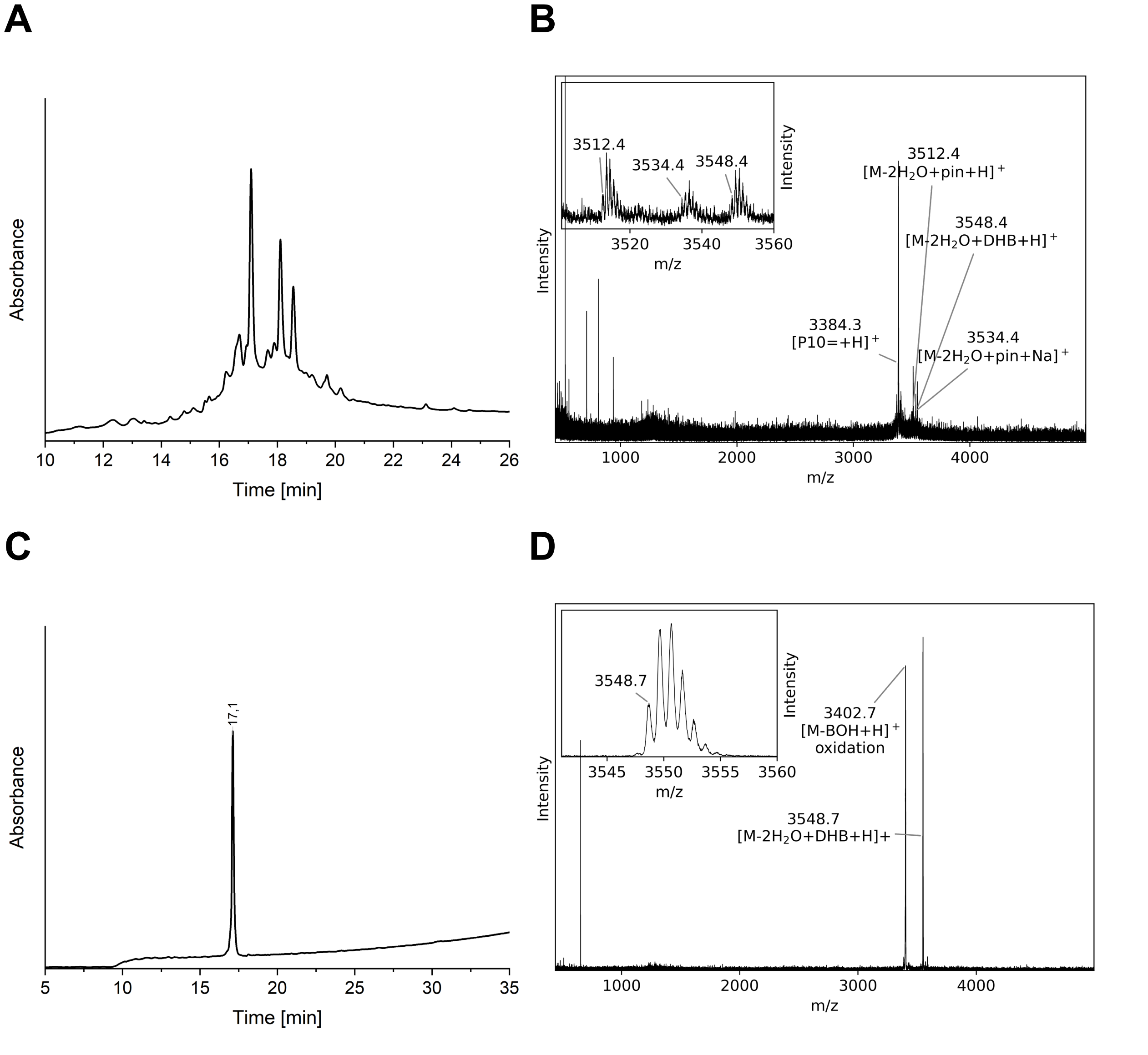

Figure S28: **P10–B(OH)$_2$** A) Analytical HPLC (220 nm, column B) of crude peptide. B) MALDI-TOF MS spectrum of crude peptide. C) Analytical HPLC (220 nm, column B) of purified peptide. D) MALDI-TOF MS spectrum of purified peptide. Calculated mass: [**P10=**+H]$^+$ 3384.6, [M-BOH+H]$^+$ 3402.6, [M-2H$_2$O+pin+H]$^+$ 3512.7, [M-2H$_2$O+pin+Na]$^+$ 3534.7, [M-2H$_2$O+DHB+H]$^+$ 3548.6.

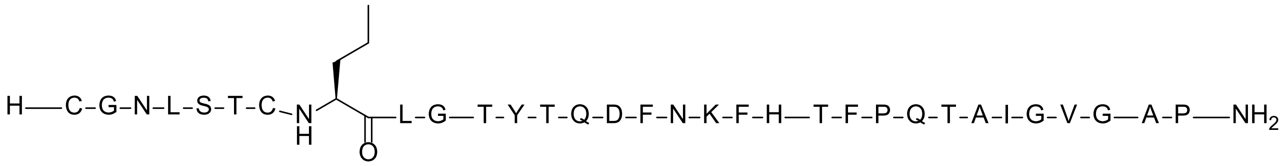

P10–

Chemical Formula: $C_{151}H_{228}N_{40}O_{45}S_2$
Exact Mass: 3385.62
Molecular Weight: 3387.84

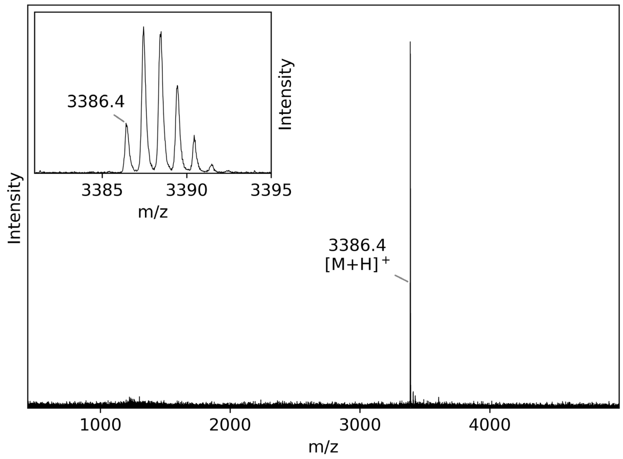

Figure S29: MALDI-TOF MS spectrum of purified **P10–**. Calculated mass: $[M+H]^+$ 3386.6.

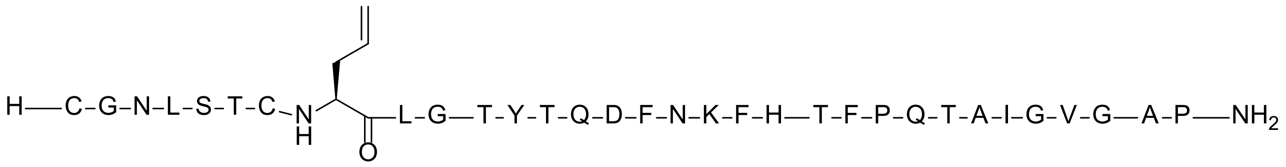

P10=

Chemical Formula: $C_{151}H_{226}N_{40}O_{45}S_2$
Exact Mass: 3383.61
Molecular Weight: 3385.82

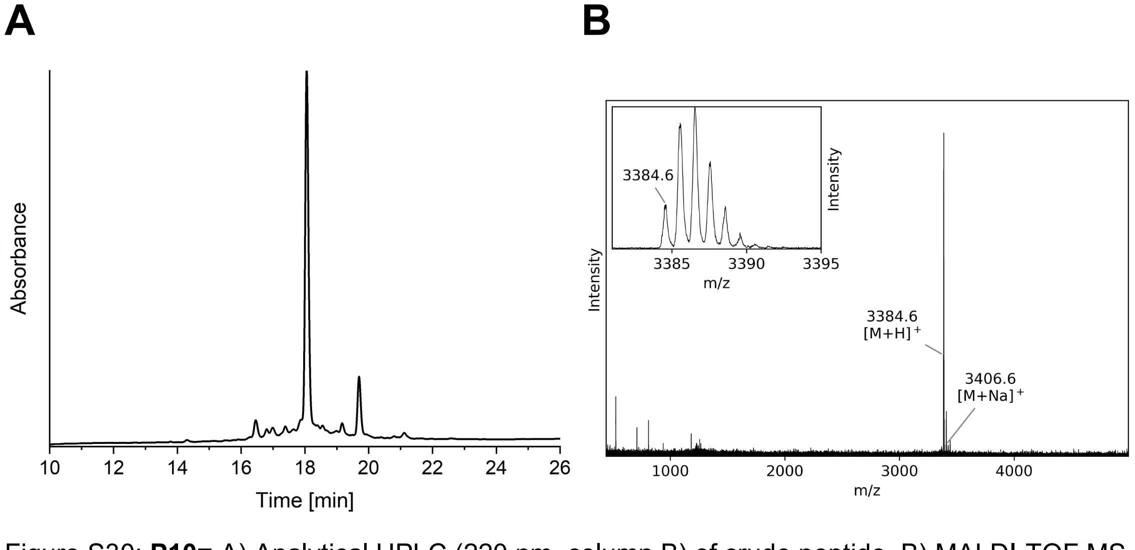

Figure S30: **P10=** A) Analytical HPLC (220 nm, column B) of crude peptide. B) MALDI-TOF MS spectrum of crude peptide. Calculated mass: $[M+H]^+$3384.6, $[M+Na]^+$3406.6.

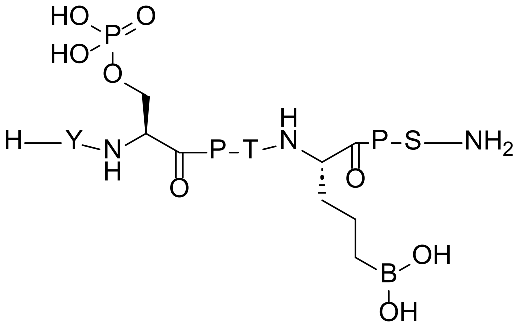

P11–B(OH)$_2$

Chemical Formula: $C_{34}H_{54}BN_8O_{16}P$

Exact Mass: 872.35

Molecular Weight: 872.63

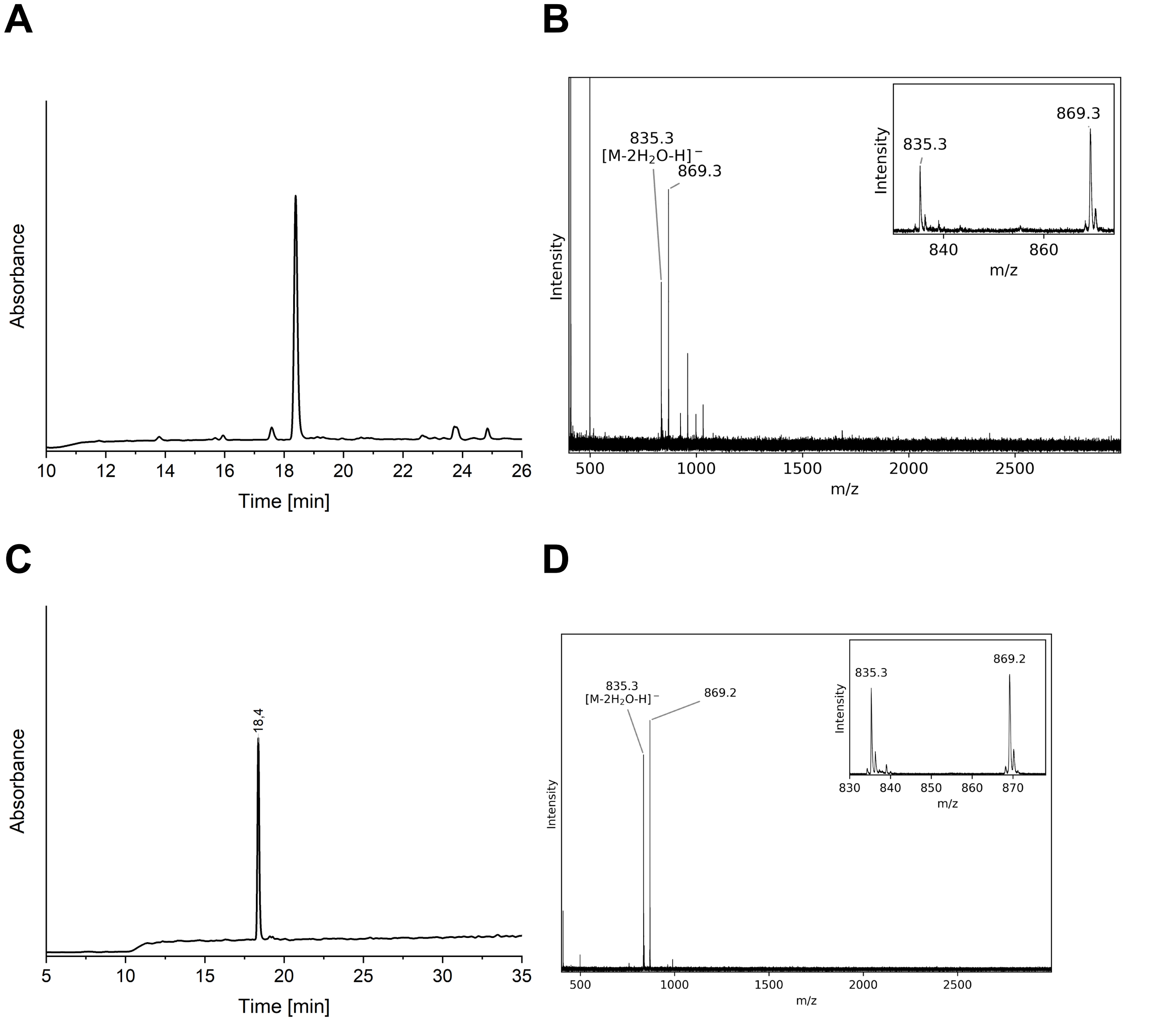

Figure S31: **P11–B(OH)$_2$** A) Analytical HPLC (220 nm, column B) of crude peptide. B) MALDI-TOF MS spectrum of crude peptide. C) Analytical HPLC (220 nm, column B) of purified peptide. D) MALDI-TOF MS spectrum of purified peptide. Calculated mass: $[M\text{-}2H_2O\text{-}H]^-$ 835.3.

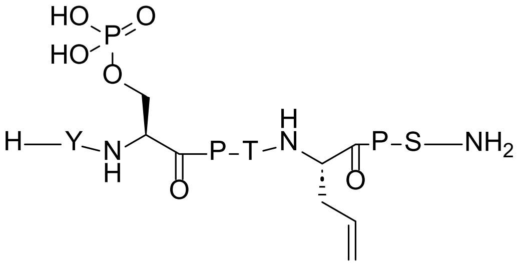

P11=

Chemical Formula: $C_{34}H_{51}N_8O_{14}P$
Exact Mass: 826.33
Molecular Weight: 826.80

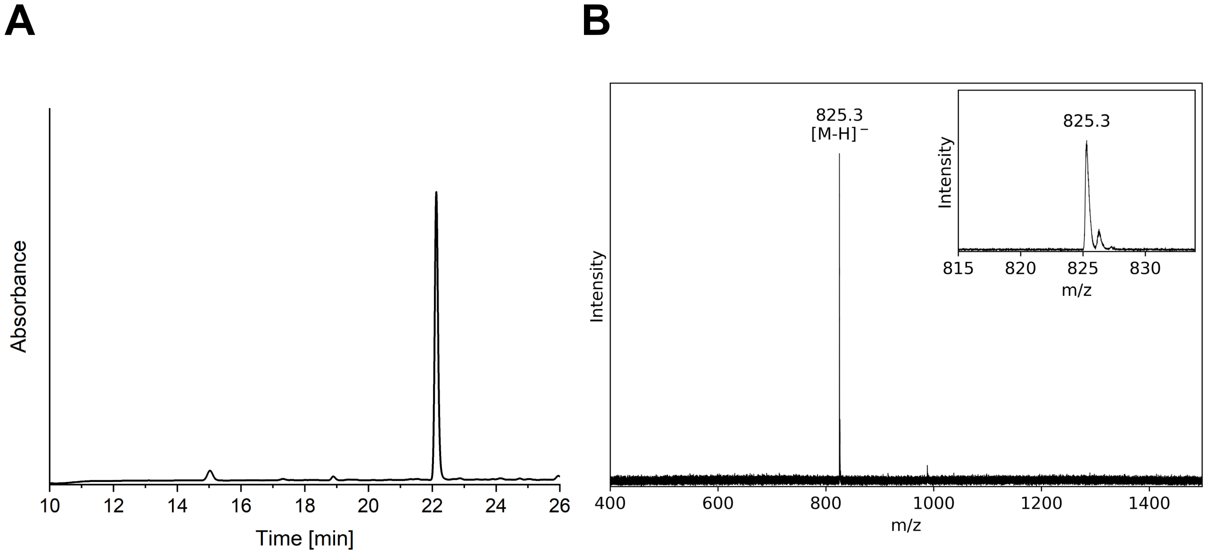

Figure S32: **P11=** A) Analytical HPLC (220 nm, column B) of crude peptide. B) MALDI-TOF MS spectrum of crude peptide. Calculated mass: $[M-H]^-$ 825.3.

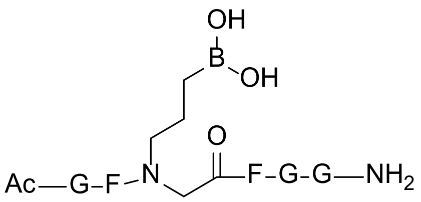

P12–B(OH)$_2$

Chemical Formula: $C_{31}H_{42}BN_7O_9$

Exact Mass: 667.31

Molecular Weight: 667.53

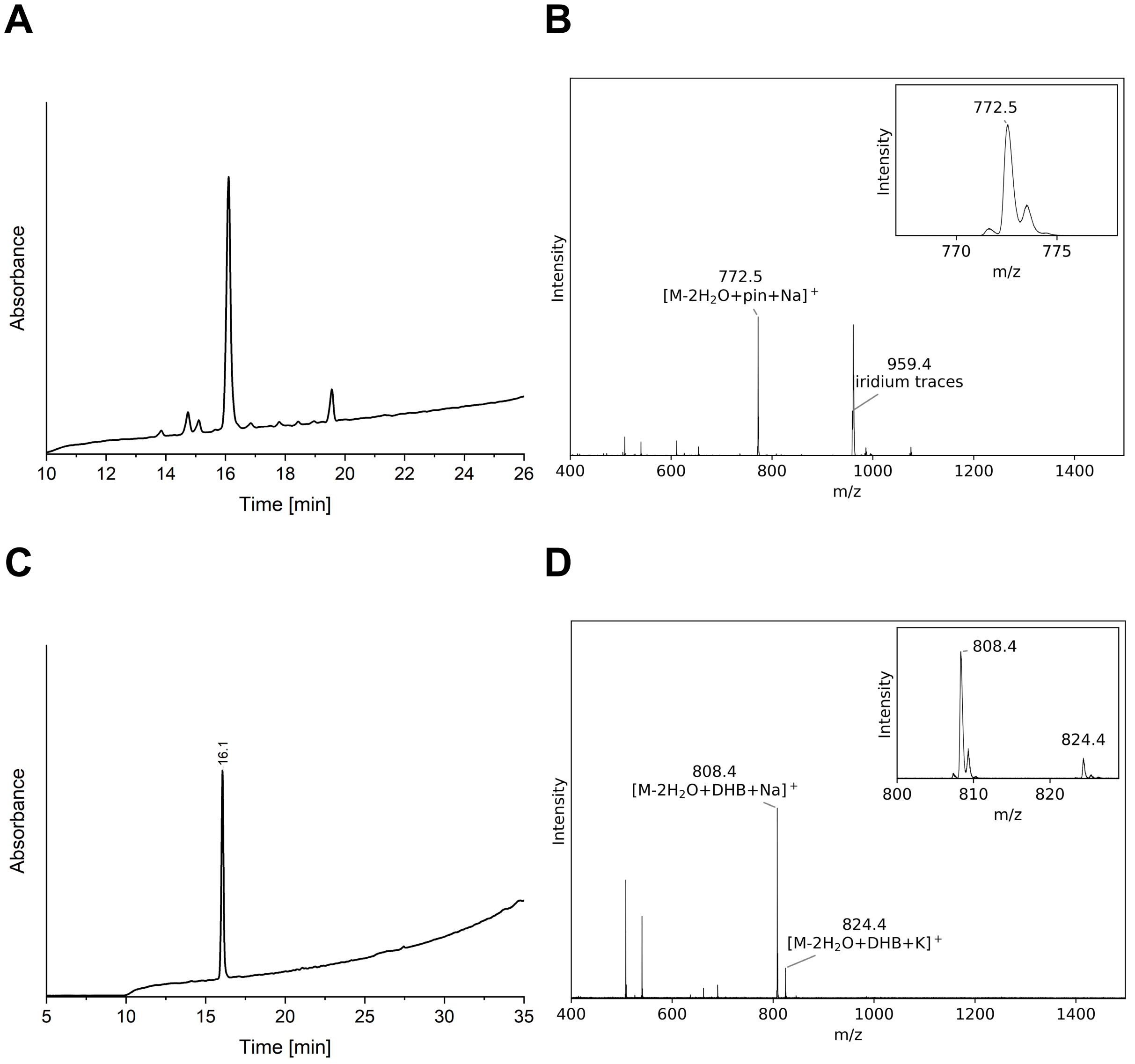

Figure S33: **P12–B(OH)$_2$** A) Analytical HPLC (220 nm, column A) of crude peptide. B) MALDI-TOF MS spectrum of crude peptide. C) Analytical HPLC (220 nm, column A) of purified peptide. D) MALDI-TOF MS spectrum of purified peptide. Calculated mass: $[M\text{-}2H_2O\text{+}pin\text{+}Na]^+$ 772.4, $[M\text{-}2H_2O\text{+}DHB\text{+}Na]^+$ 808.3, $[M\text{-}2H_2O\text{+}DHB\text{+}K]^+$ 824.3.

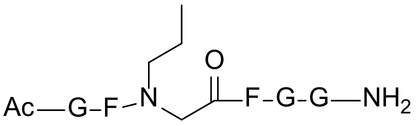

P12–

Chemical Formula: $C_{31}H_{41}N_{7}O_{7}$
Exact Mass: 623.31
Molecular Weight: 623.71

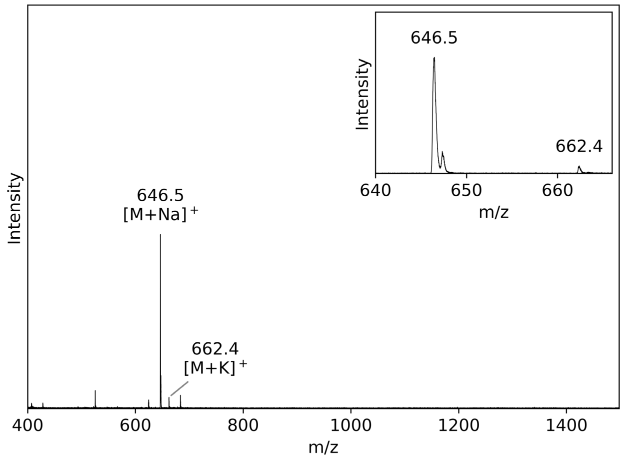

Figure S34: MALDI-TOF MS spectrum of purified **P12–** peptide. Calculated mass: $[M+Na]^+$ 646.3, $[M+K]^+$ 662.3.

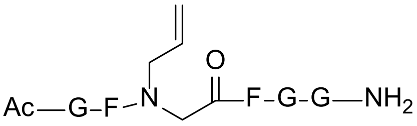

P12=

Chemical Formula: $C_{31}H_{39}N_{7}O_{7}$
Exact Mass: 621.29
Molecular Weight: 621.70

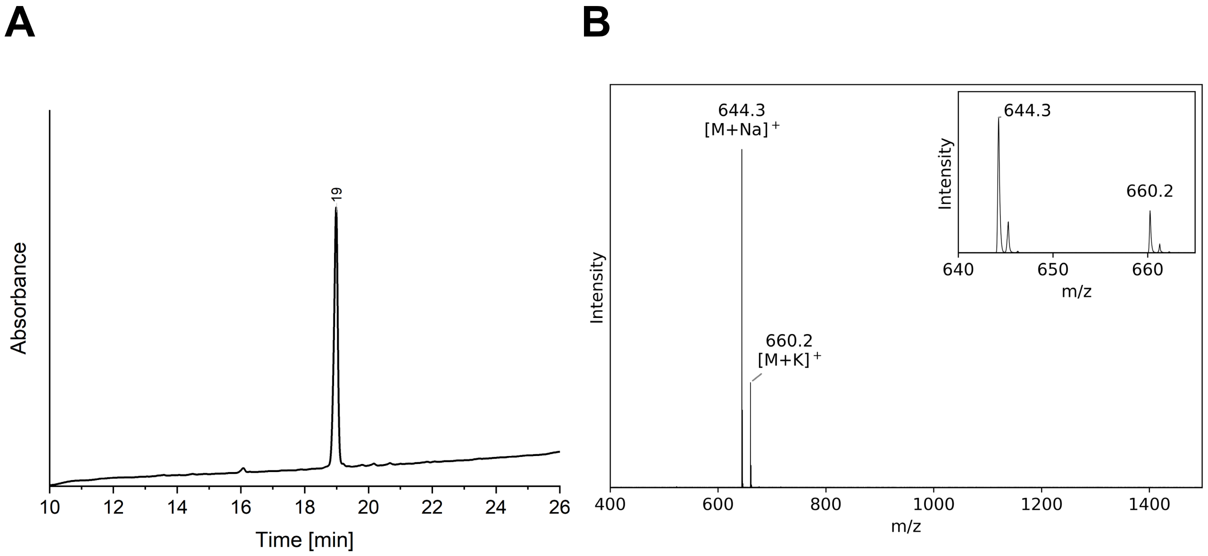

Figure S35: **P12=** A) Analytical HPLC (220 nm, column A) of crude peptide. B) MALDI-TOF MS spectrum of crude peptide. Calculated mass: $[M+Na]^+$ 644.3, $[M+K]^+$ 660.3.

Table S1: Yields of peptide boronic acids prepared by hydroboration based on HPLC traces.

Peptide	Yield
**P1–B(OH)$_2$**	90%
**P2–B(OH)$_2$**	97%
**P3–B(OH)$_2$**	82%
**P4–B(OH)$_2$**	66%
**P5–B(OH)$_2$**	74%
**P6–B(OH)$_2$**	88%
**P7–B(OH)$_2$**	89%
**P8–B(OH)$_2$**	83%
**P9–B(OH)$_2$**	73%
**P10–B(OH)$_2$**	undefined
**P11–B(OH)$_2$**	>99%
**P12–B(OH)$_2$**	91%

Yields relate to the amount of hydroborated products including hydroborated by-products.

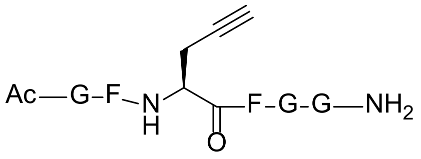

P1≡

Chemical Formula: $C_{31}H_{37}N_7O_7$

Exact Mass: 619.28

Molecular Weight: 619.68

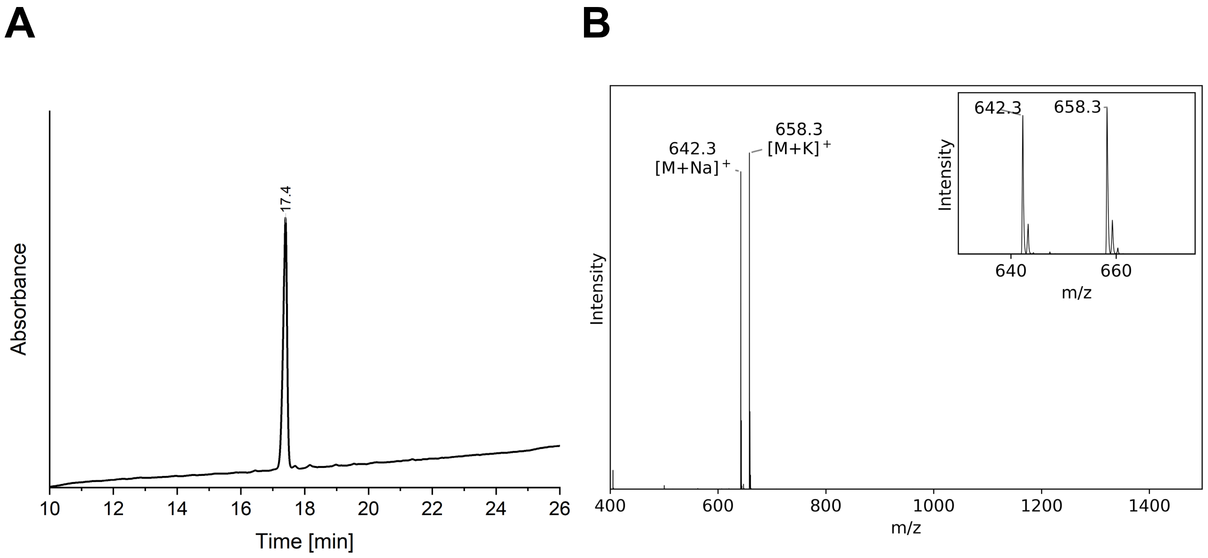

Figure S36: **P1≡** A) Analytical HPLC (220 nm, column A) of crude peptide. B) MALDI-TOF MS spectrum of crude peptide. Calculated mass: $[M+Na]^+$ 642.3, $[M+K]^+$ 658.2.

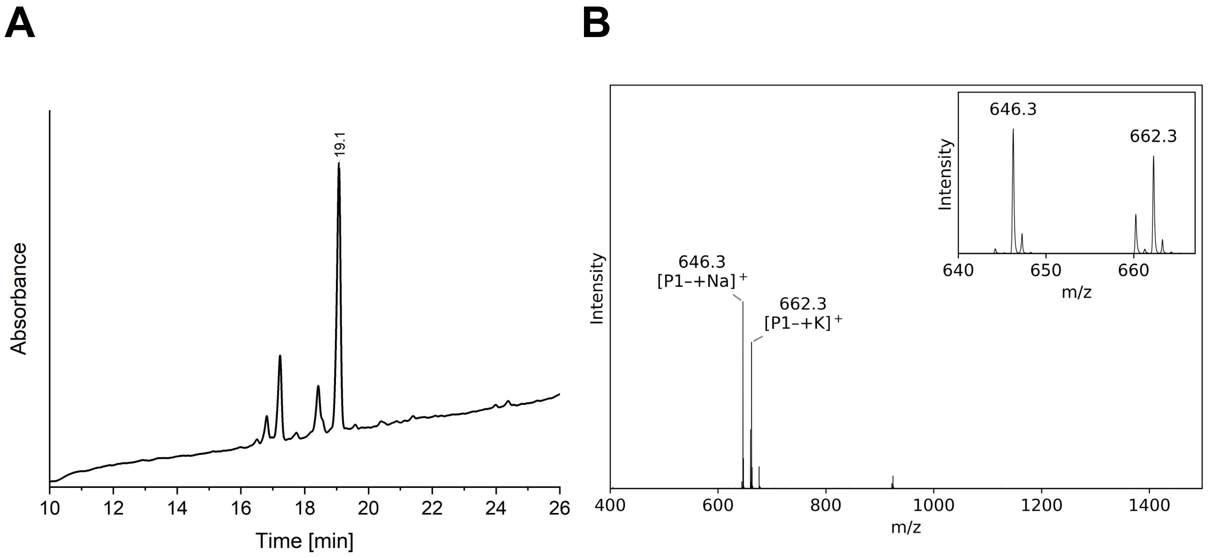

Figure S37: Attempt of hydroboration of **P1≡** with $[Ir(COD)Cl]_2$/2dppm in cyclohexane. A) Analytical HPLC (220 nm, column A) of crude peptide. The by-product with a retention time of 19.1 min refers to the norvaline-containing peptide. B) MALDI-TOF MS spectrum of crude peptide.

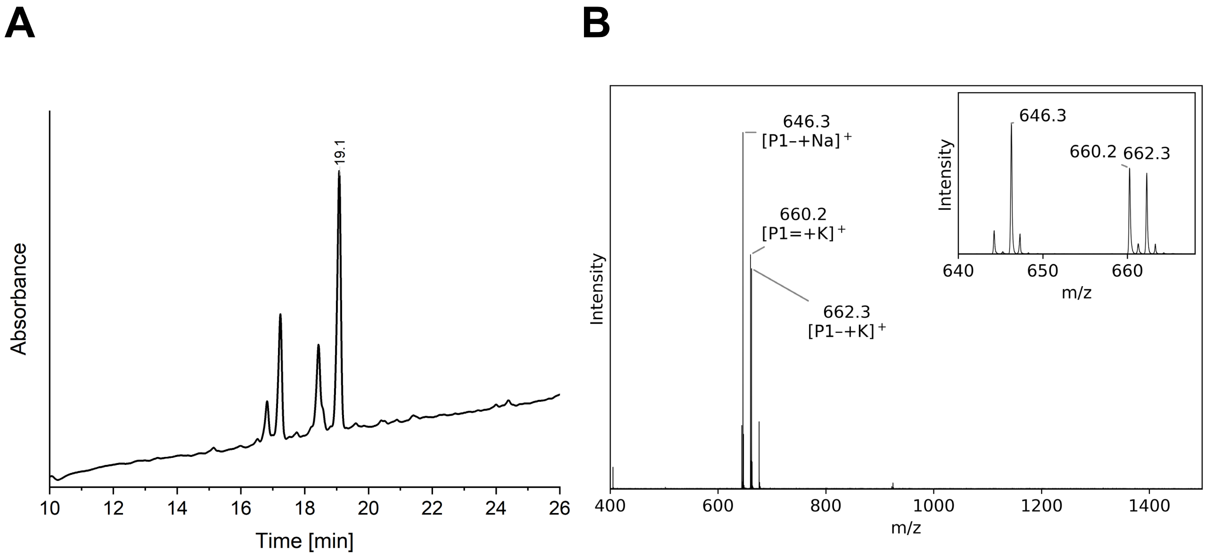

Figure S38: Attempt of hydroboration of **P1≡** with $[Ir(COD)Cl]_2$/4PCy_3 in cyclohexane. A) Analytical HPLC (220 nm, column A) of crude peptide. The by-product with a retention time of 19.1 min refers to the norvaline-containing peptide. B) MALDI-TOF MS spectrum of crude peptide.

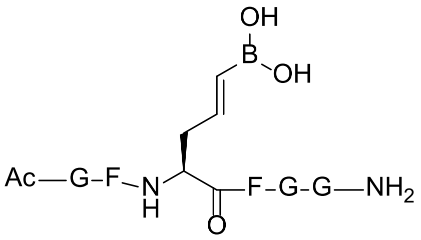

P1=B(OH)$_2$

Chemical Formula: $C_{31}H_{40}BN_7O_9$

Exact Mass: 665.30

Molecular Weight: 665.51

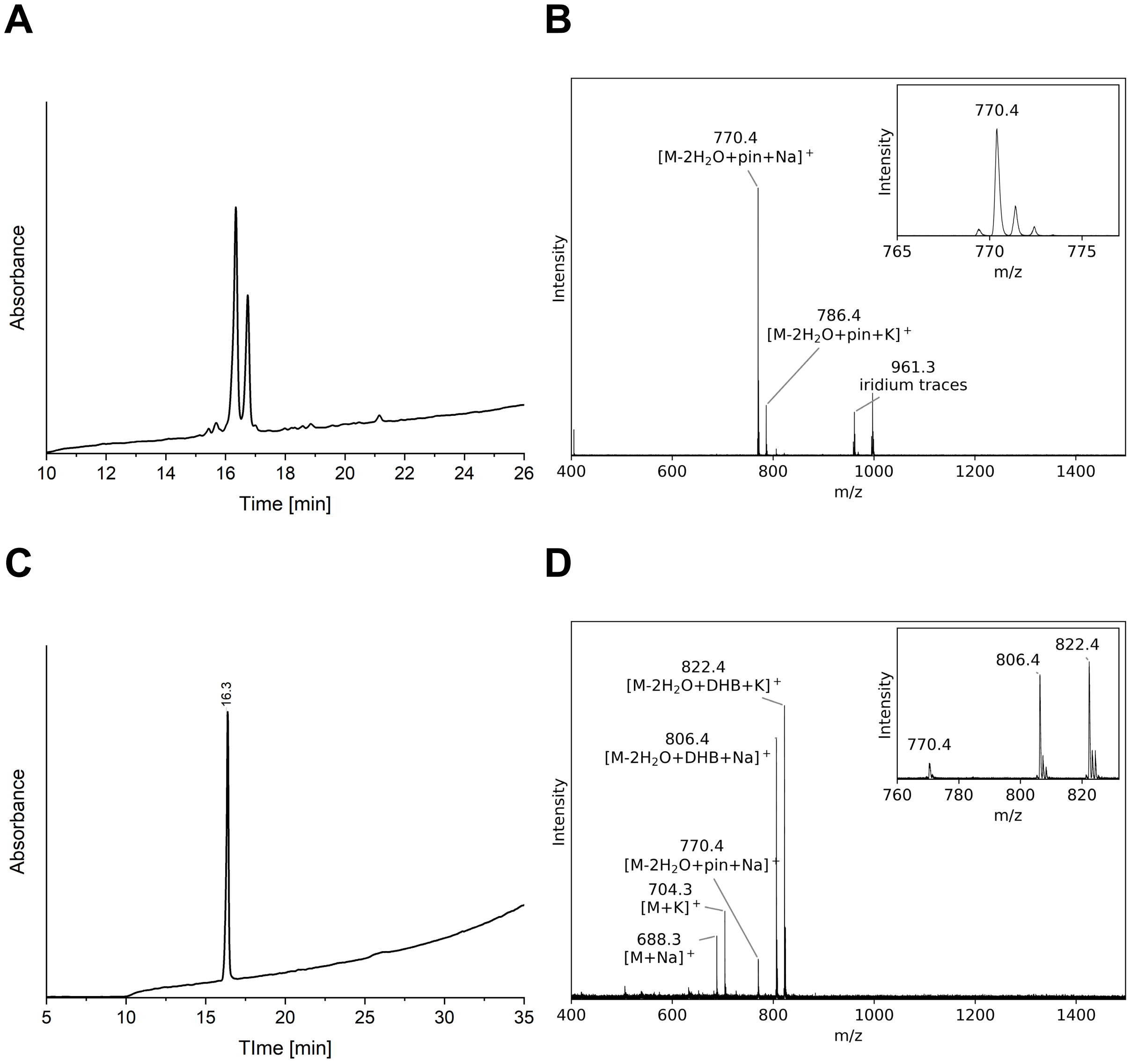

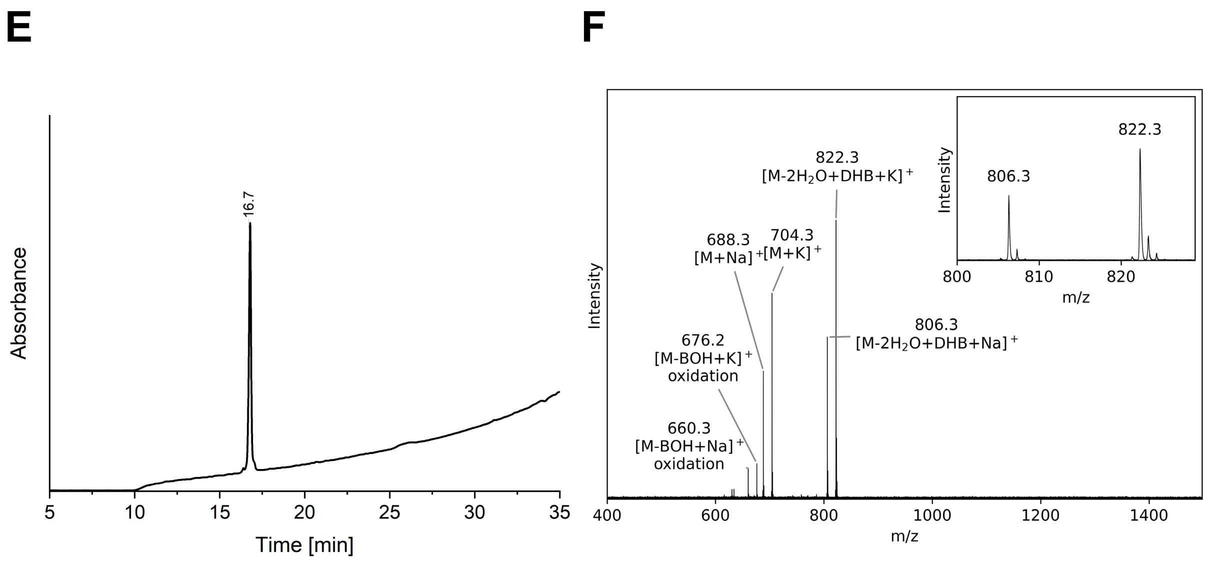

Figure S39: **P1=B(OH)$_2$** Iridium-catalyzed hydroboration. A) Analytical HPLC (220 nm, column A) of crude peptide. B). MALDI-TOF MS spectrum of crude peptide of entry. C) Analytical HPLC (220 nm, column A) of purified peptide with a retention time of 16.3 min. D) MALDI-TOF MS spectrum of purified peptide with a retention time of 16.3 min. E) Analytical HPLC (220 nm, column A) of purified peptide with a retention time of 16.7 min. F) MALDI-TOF MS spectrum of purified peptide with a retention time of 16.7 min. Calculated mass: $[M-BOH+Na]^+$ 660.3, $[M-BOH+K]^+$ 676.2, $[M+Na]^+$ 688.3, $[M+K]^+$ 704.3, $[M-2H_2O+pin+Na]^+$ 770.4, $[M-2H_2O+pin+K]^+$ 786.3, $[M-2H_2O+DHB+Na]^+$ 806.3, $[M-2H_2O+DHB+K]^+$ 822.3.

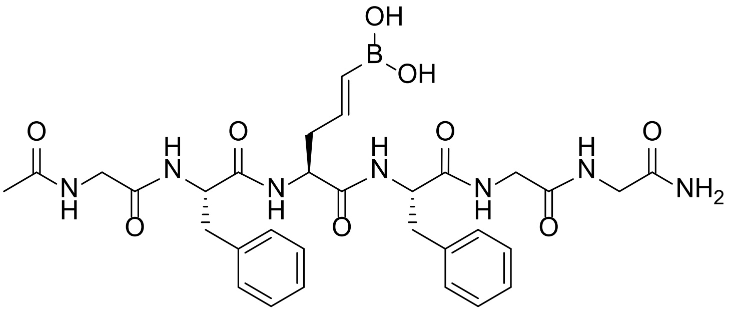

^{1}H-NMR (400 MHz, DMSO-d_6, 295 K):
δ [ppm] = 7.25 − 7.19 (m, 10H, H-Aryl Phe), 6.32 (dt, $^3J_{HH}$ = 17.8 Hz, 6.6 Hz, 1H, H-Alkene), 5.40 (d, $^3J_{HH}$ = 17.9 Hz, 1H, H-Alkene), 4.54 − 4.48 (m, 2H, α-CH Phe), 4.32 − 4.26 (m, 1H, α-CH X), 3.73 − 3.63 (m, 6H, CH_2 Gly), 3.07 − 2.94 (m, 2H, β-CH_2 Phe), 2.89 − 2.66 (m, 2H, β-CH_2 Phe), 2.47 – 2.28 (m, 2H, β-CH_2 -X), 1.81 (s, 3H, Ac)

A

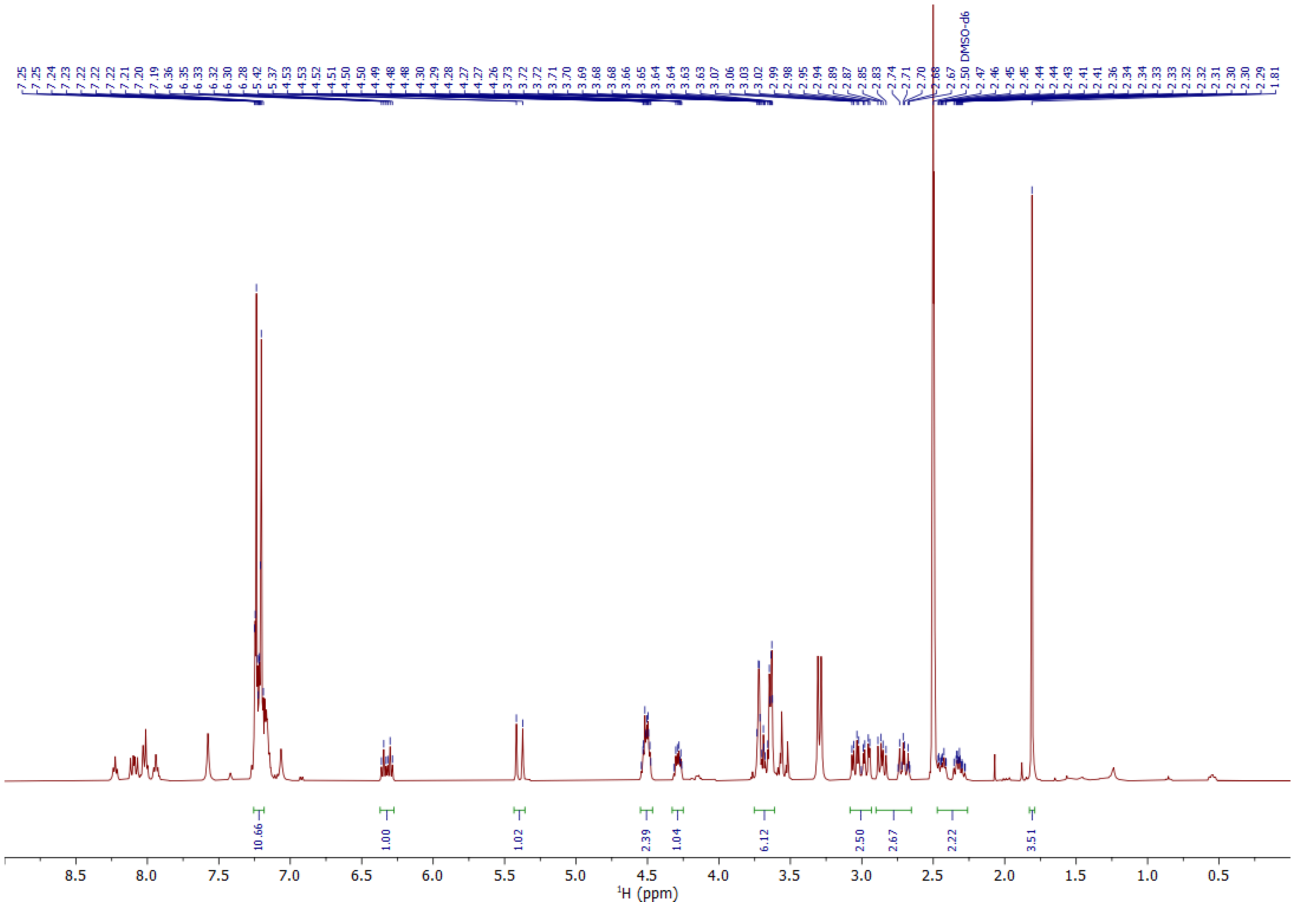

B

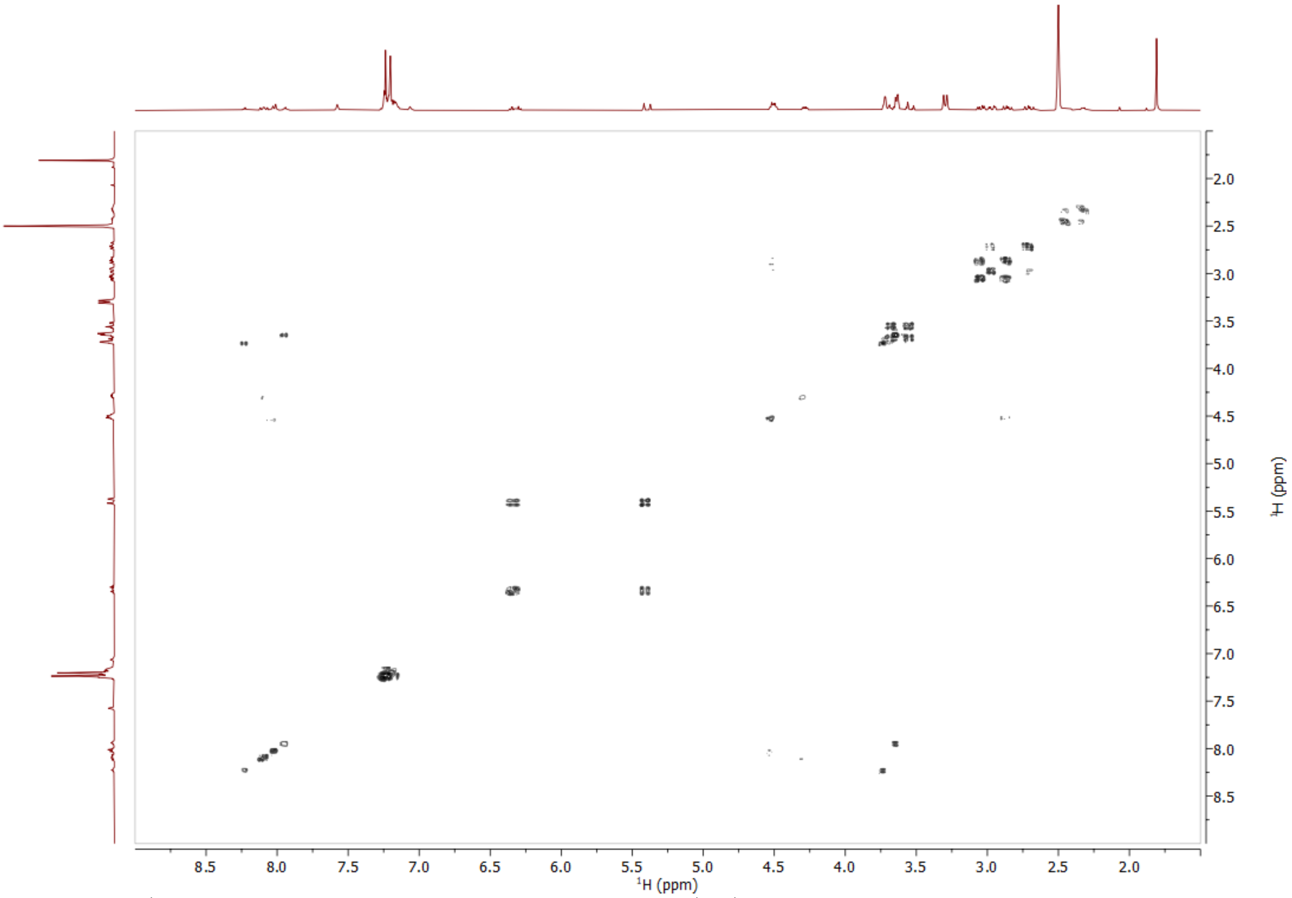

Figure S40: ^{1}H-NMR (400 MHz, DMSO-d_6, 295 K) (A) and ^{1}H-^{1}H-COSY (400 MHz, 400 MHz, DMSO-d_6, 295 K) (B) of **P1=B(OH)$_2$** with a retention time of 16.3 min (column A), prepared by iridium-catalyzed hydroboration. Peaks at 7.5-8.5 ppm are from incomplete hydrogen-deuterium exchange of peptide backbone amide protons.

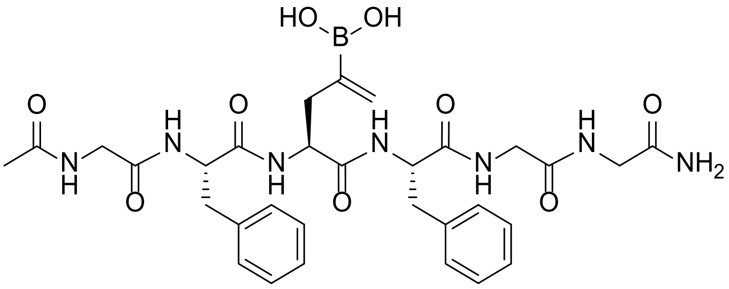

^{1}H-NMR (400 MHz, DMSO-d$_6$, 295 K):
δ [ppm] = 7.26 − 7.21 (m, 10H, H-Aryl Phe), 5.69 (d, $^{2}J_{HH}$ = 3.5 Hz, 1H, H-Alkene)
5.43 (d, $^{2}J_{HH}$ = 3.5 Hz, 1H, H-Alkene), 4.54 − 4.46 (m, 2H, α-CH Phe), 4.33 − 4.27 (m, 1H, α-CH X),
3.74 − 3.61 (m, 6H, CH_2 Gly), 3.08 − 2.95 (m, 2H, β-CH_2 Phe), 2.89 − 2.66 (m, 2H, β-CH_2 Phe),
2.45 – 2.23 (m, 2H, β-CH_2 -X), 1.81 (s, 3H, Ac)

A

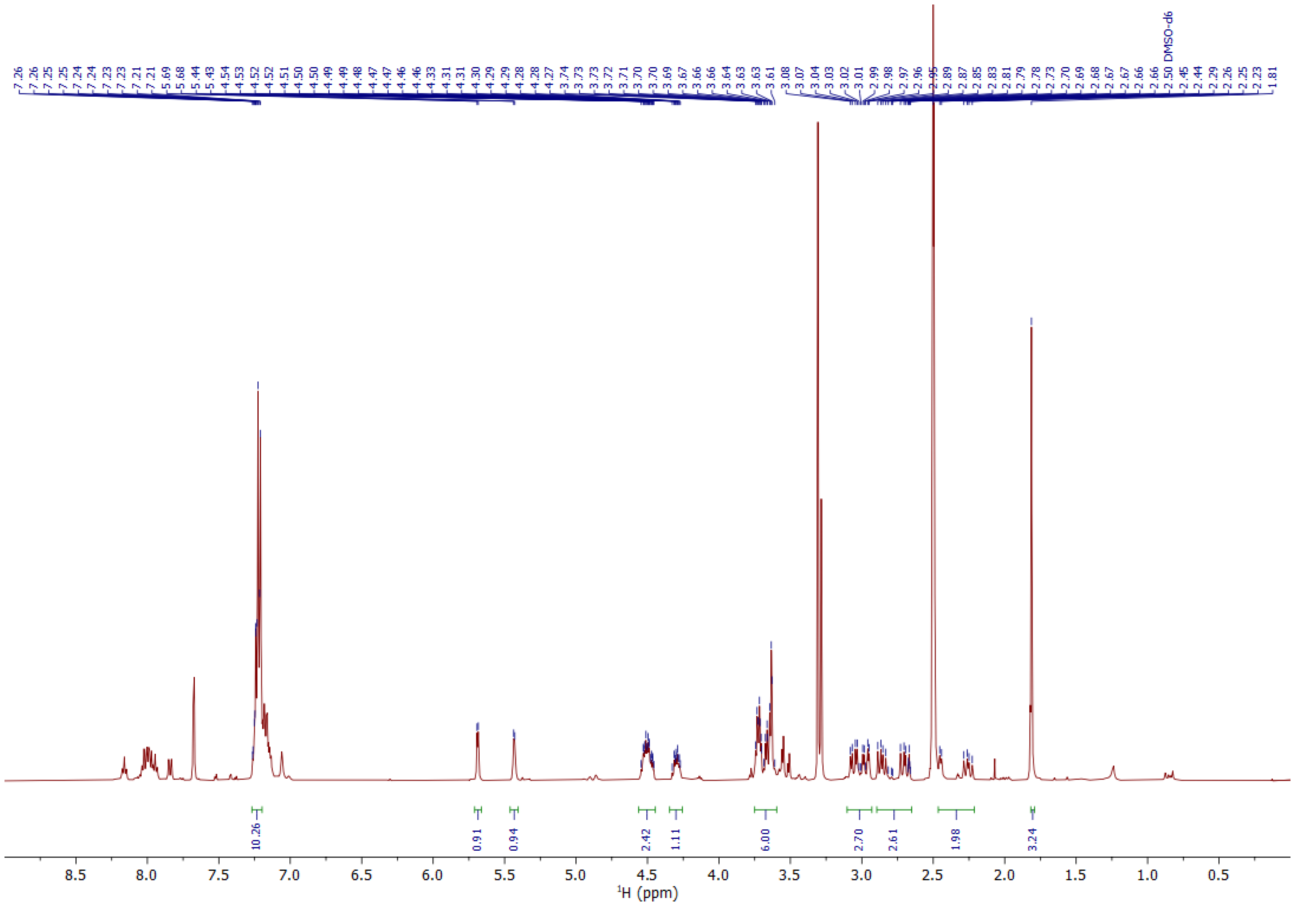

B

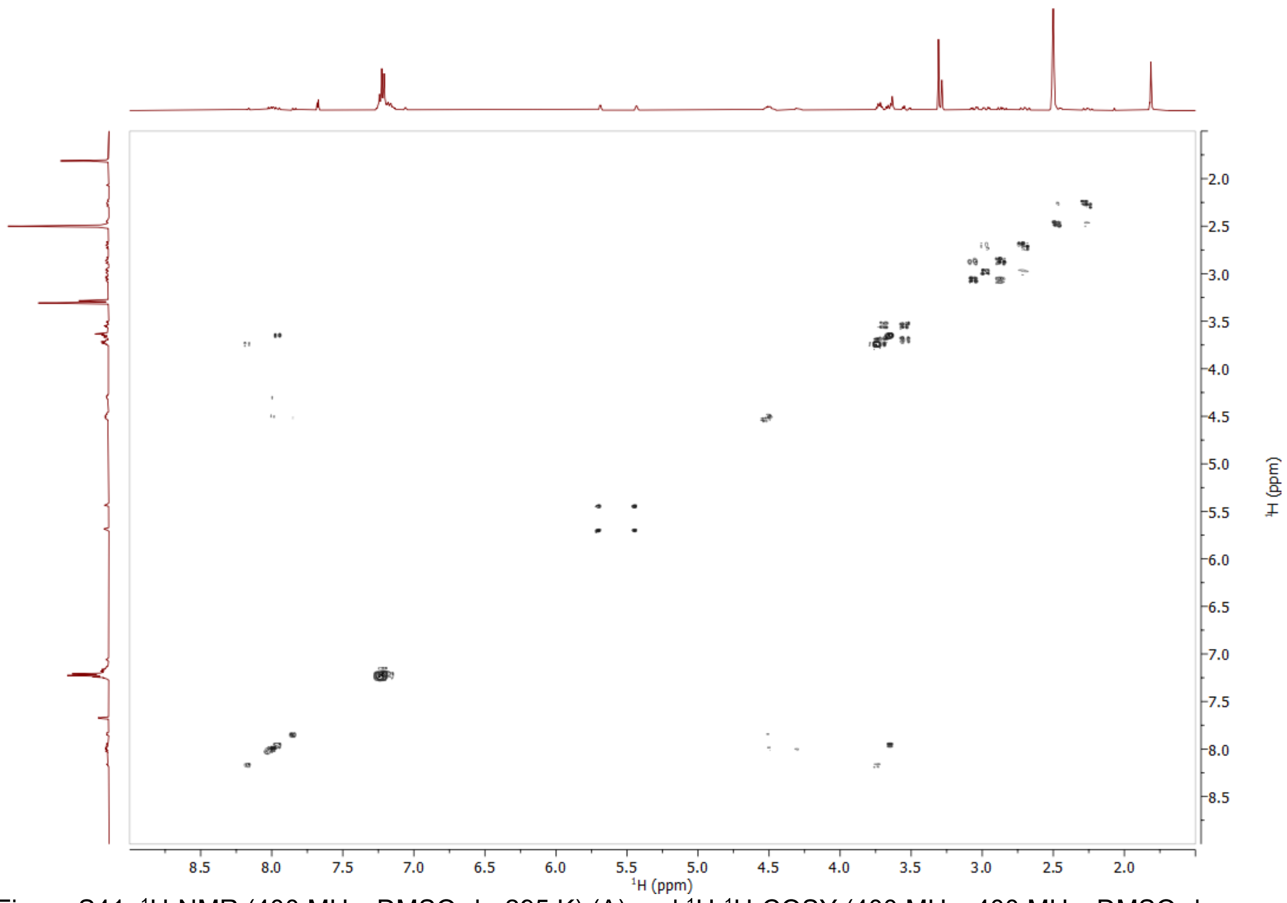

Figure S41: ^{1}H-NMR (400 MHz, DMSO-d_6, 295 K) (A) and ^{1}H-^{1}H-COSY (400 MHz, 400 MHz, DMSO-d_6, 295 K) (B) of **P1=B(OH)$_2$** with a retention time of 16.7 min (column A), prepared by iridium-catalyzed hydroboration. Peaks at 7.5-8.5 ppm are from incomplete hydrogen-deuterium exchange of peptide backbone amide protons.

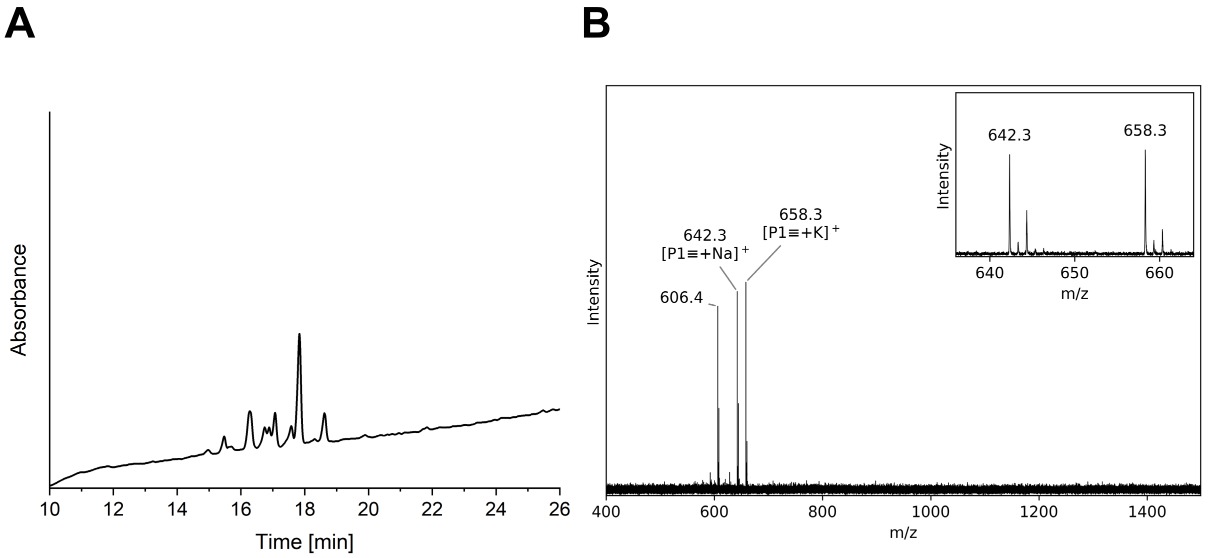

Figure S42: Attempt of hydroboration of **P1≡** with [$Cp_2Zr(H)Cl$]. A) Analytical HPLC (220 nm, column A) of crude peptide. B) MALDI-TOF MS spectrum of crude peptide. The signal of 606.4 m/z was not further identified. Calculated mass: [**P1≡**+Na]$^+$ 642.3, [**P1≡** +K]$^+$ 658.2.

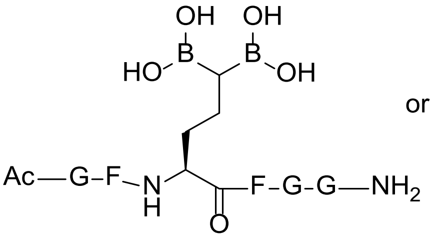

or isomers

Chemical Formula: $C_{31}H_{43}B_2N_7O_{11}$
Exact Mass: 711.3
Molecular Weight: 711.3

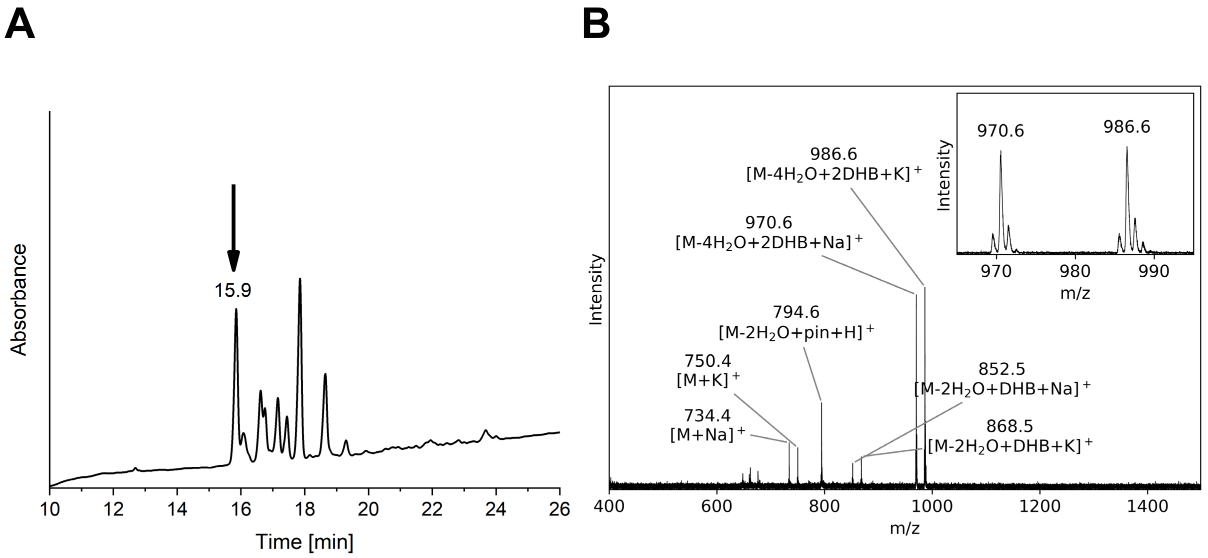

Figure S43: Attempt of hydroboration of **P1≡** with Rh(COD)$Cl]_2$/2dppm. A) Analytical HPLC (220 nm, column A) of crude peptide. B) MALDI-TOF MS spectrum of purified peptide with a retention time of 15.9 min. Calculated mass: [M+Na]$^+$ 734.3, [M+K]$^+$ 750.3, [M-2H_2O+pin+H]$^+$ 794.4, [M-2H_2O+DHB+Na]$^+$ 852.3, [M-2H_2O+DHB+K]$^+$ 868.3, [M-4H_2O+2DHB+Na]$^+$ 970.3, [M-4H_2O+2DHB+K]$^+$ 986.3.

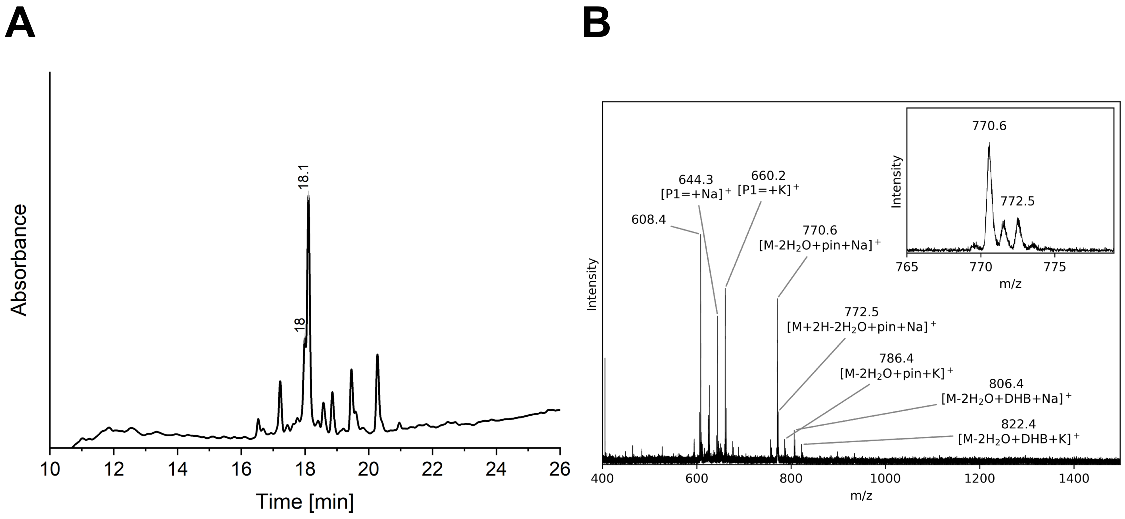

Figure S44: Attempt of hydroboration of **P1≡** with [Rh(CO)(PPh_3)$_2$Cl]. A) Analytical HPLC (220 nm, column B) of crude peptide. B) MALDI-TOF MS spectrum of crude peptide. The signal of 608.4 m/z was not further identified. Calculated mass: [**P1=**+Na]$^+$ 644.3, [**P1=**+K]$^+$ 660.3, [M-2H_2O+pin+Na]$^+$ 770.4, [M+2H-2H_2O+pin+Na]$^+$ 772.4, [M-2H_2O+pin+K]$^+$ 786.3, [M-2H_2O+DHB+Na]$^+$ 806.3, [M-2H_2O+DHB+K]$^+$ 822.3.

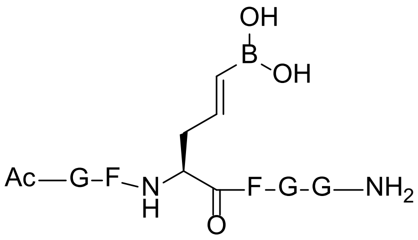

P1=B(OH)$_2$

Chemical Formula: $C_{31}H_{40}BN_7O_9$

Exact Mass: 665.30

Molecular Weight: 665.51

A

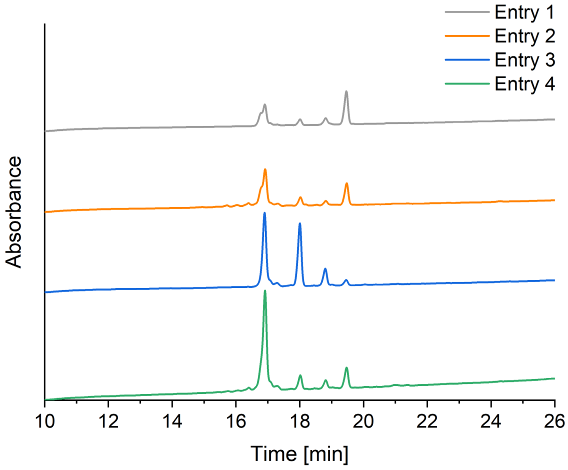

B

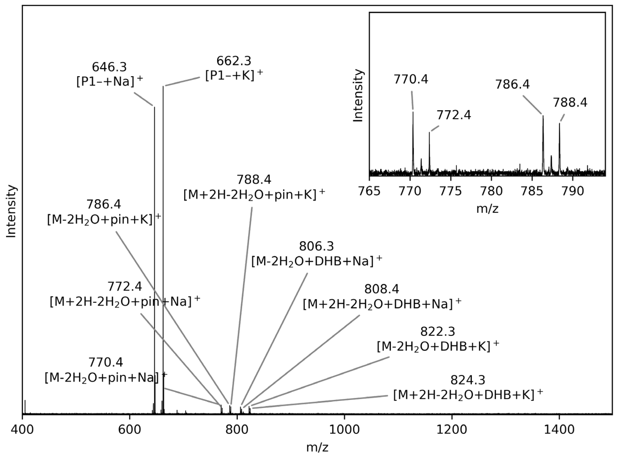

C

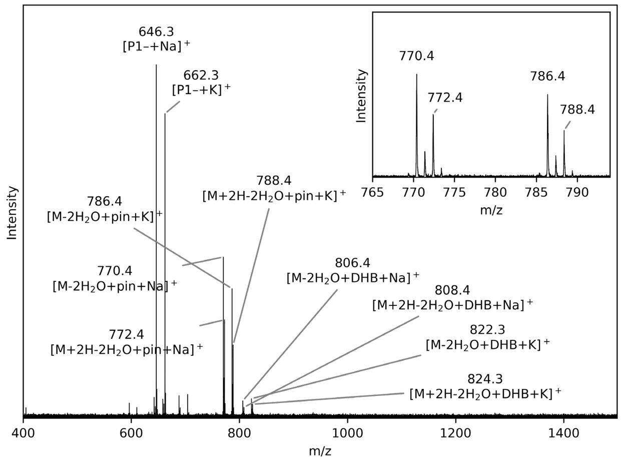

D

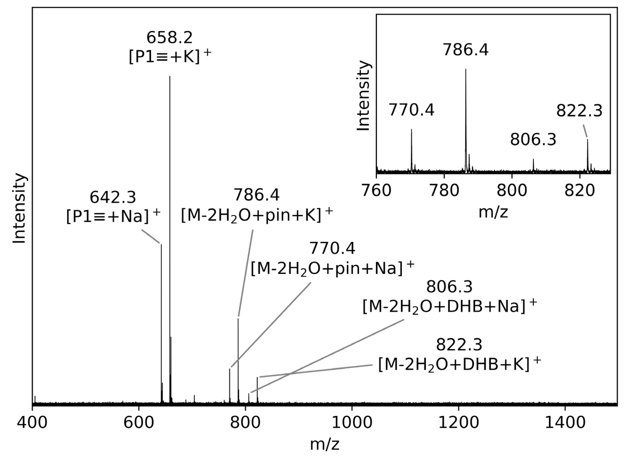

E

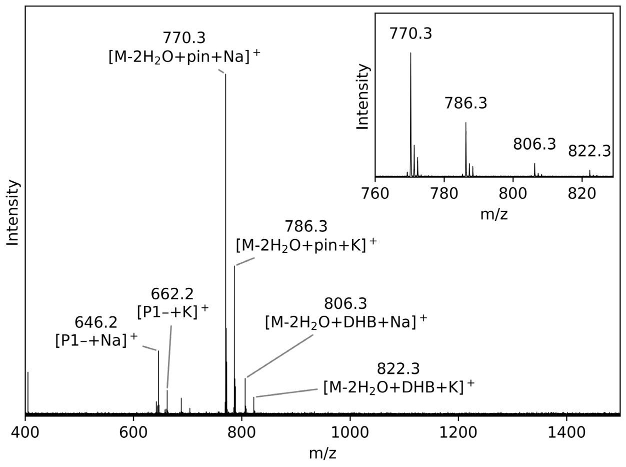

F

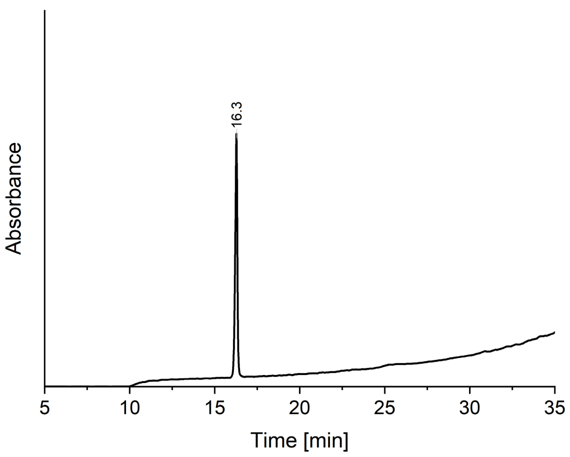

G

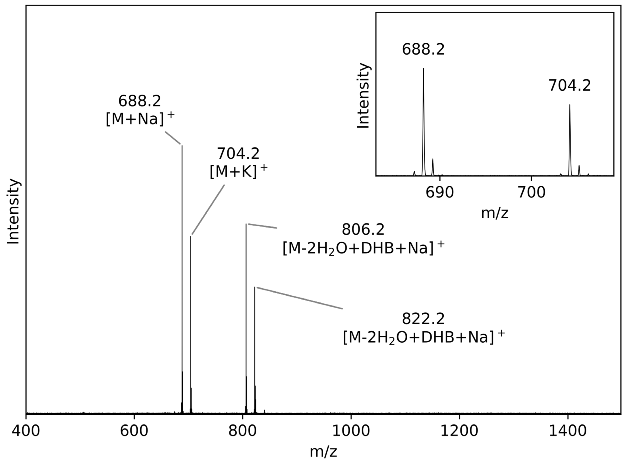

Figure S45: **P1=B(OH)₂** [Ru(CO)(Cl)H(PPh_3)$_3$]-catalyzed hydroboration. A) Analytical HPLC (220 nm, column A) of entry 1 to 4 referring to conditions of Table 3. B) MALDI-TOF MS spectrum of crude peptide of entry 1. C) MALDI-TOF MS spectrum of crude peptide of entry 2. D) MALDI-TOF MS spectrum of crude peptide of entry 3.E) MALDI-TOF MS spectrum of crude peptide of entry 4. F) Analytical HPLC (220 nm, column A) of purified peptide. G) MALDI-TOF MS spectrum of purified peptide. Calculated mass: [**P1≡**+Na]$^+$ 642.3, [**P1–**+Na]$^+$ 646.3, [**P1≡** +K]$^+$ 658.2, [**P1–**+K]$^+$ 662.3, [M+Na]$^+$ 688.3, [M+K]$^+$ 704.3, [M-2H_2O+pin+Na]$^+$ 770.4, [M+2H-2H_2O+pin+Na]$^+$ 772.4, [M-2H_2O+pin+K]$^+$ 786.3, [M+2H-2H_2O+pin+K]$^+$ 788.4, [M-2H_2O+DHB+Na]$^+$ 806.3, [M+2H-2H_2O+DHB+Na]$^+$ 808.3, [M-2H_2O+DHB+K]$^+$ 822.3 [M+2H-2H_2O+DHB+K]$^+$ 824.3.

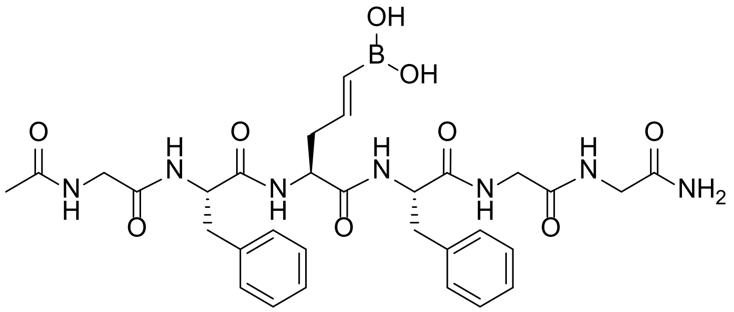

^{1}H-NMR (400 MHz, DMSO-d$_6$, 295 K):
δ [ppm] = 7.25 − 7.19 (m, 10H, H-Aryl Phe), 6.32 (dt, $^3J_{HH}$ = 17.8 Hz, 6.6 Hz, 1H, H-Alkene), 5.40 (d, $^3J_{HH}$ = 17.9 Hz, 1H, H-Alkene), 4.54 − 4.48 (m, 2H, α-CH Phe), 4.32 − 4.26 (m, 1H, α-CH X), 3.73 − 3.63 (m, 6H, CH_2 Gly), 3.07 − 2.94 (m, 2H, β-CH_2 Phe), 2.89 − 2.66 (m, 2H, β-CH_2 Phe), 2.47 – 2.28 (m, 2H, β-CH_2 -X) 1.81 (s, 3H, Ac)

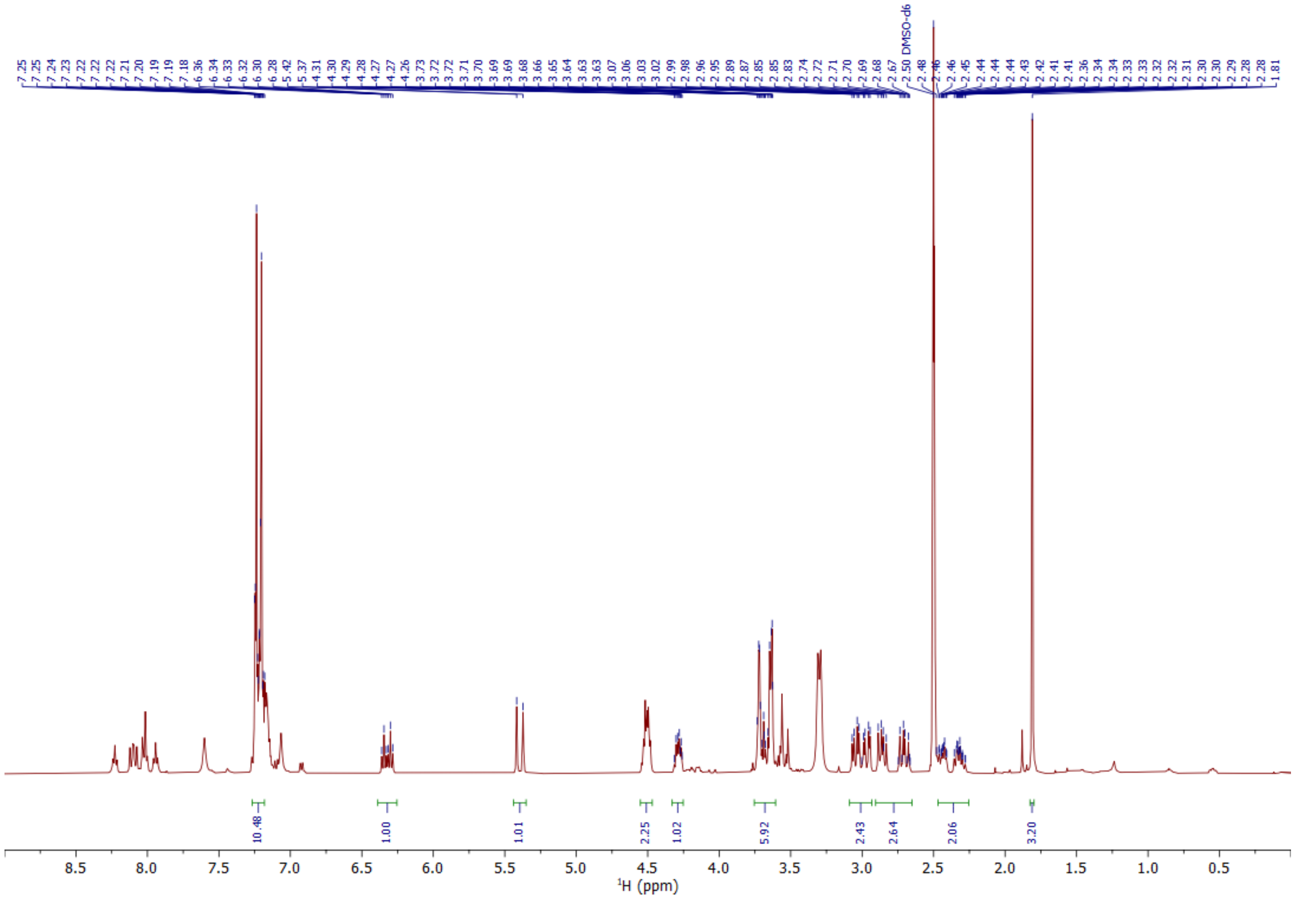

Figure S46: ^{1}H-NMR (400 MHz, DMSO-d$_6$, 295 K) of **P1=B(OH)$_2$**, prepared by [Ru(CO)(Cl)H(PPh$_3$)$_3$]-catalyzed hydroboration.

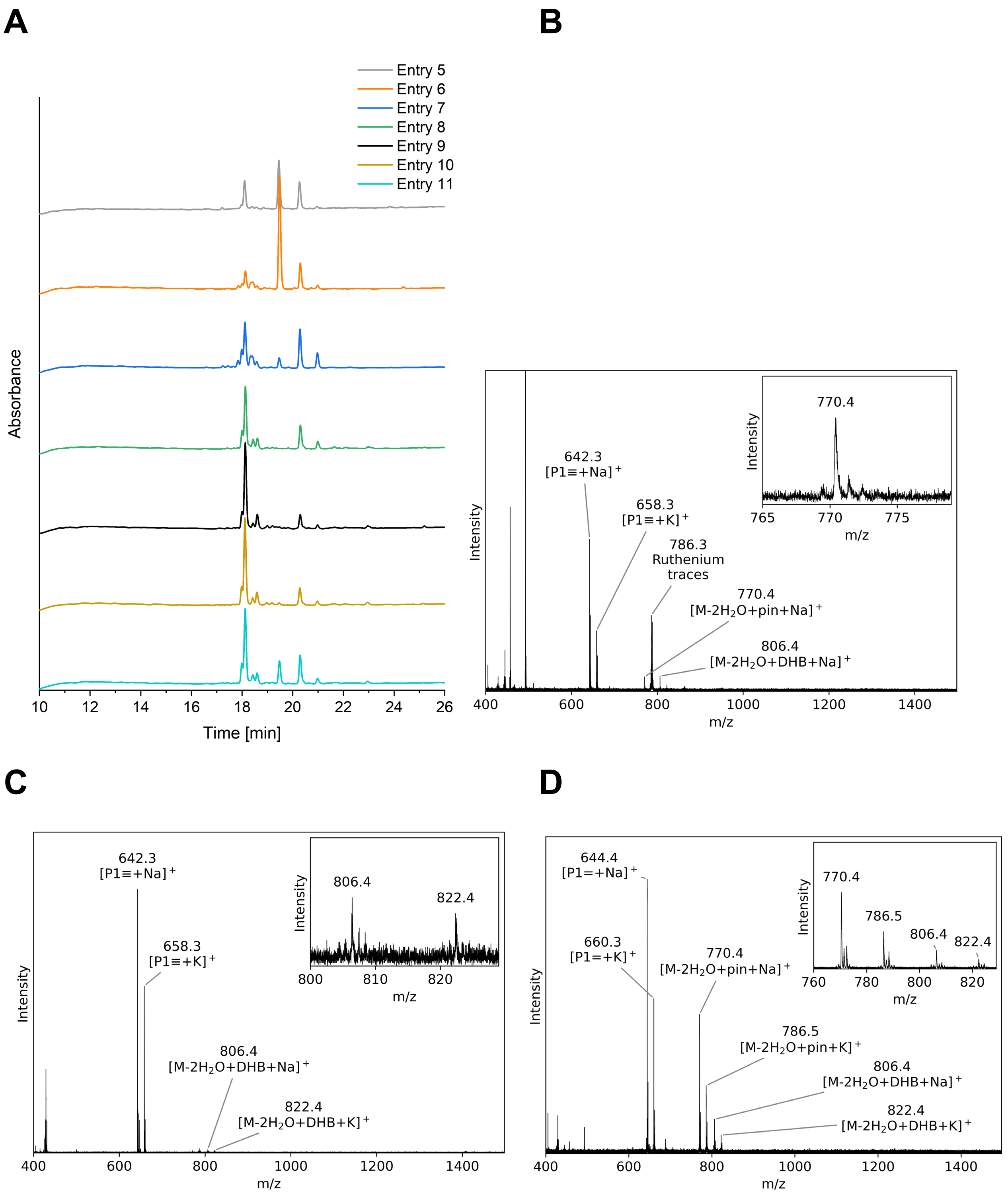
A
Entry 5
Entry 6
Entry 7
Entry 8
Entry 9
Entry 10
Entry 11
Absorbance
10 12 14 16 18 20 22 24 26
Time [min]
B
Intensity
770.4
765 770 775
m/z
642.3
[P1≡+Na]$^+$
658.3
[P1≡+K]$^+$
786.3
Ruthenium
traces
770.4
[M-2H$_2$O+pin+Na]$^+$
806.4
[M-2H$_2$O+DHB+Na]$^+$
400 600 800 1000 1200 1400
m/z
C
Intensity
642.3
[P1≡+Na]$^+$
658.3
[P1≡+K]$^+$
806.4
822.4
800 810 820
m/z
806.4
[M-2H$_2$O+DHB+Na]$^+$
822.4
[M-2H$_2$O+DHB+K]$^+$
400 600 800 1000 1200 1400
m/z
D
Intensity
644.4
[P1=+Na]$^+$
660.3
[P1=+K]$^+$
770.4
[M-2H$_2$O+pin+Na]$^+$
770.4
786.5
806.4
822.4
760 780 800 820
m/z
786.5
[M-2H$_2$O+pin+K]$^+$
806.4
[M-2H$_2$O+DHB+Na]$^+$
822.4
[M-2H$_2$O+DHB+K]$^+$
400 600 800 1000 1200 1400
m/z

E

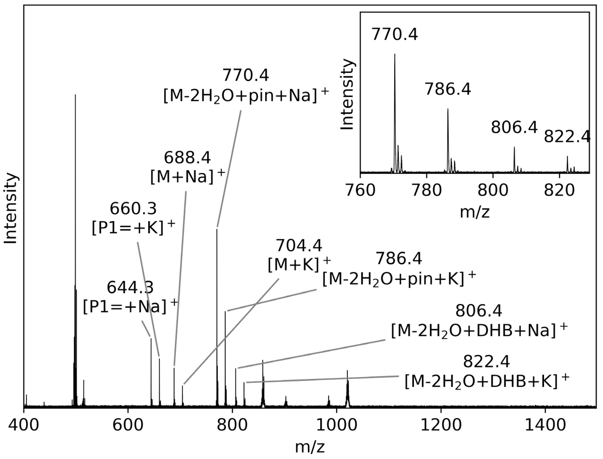

F

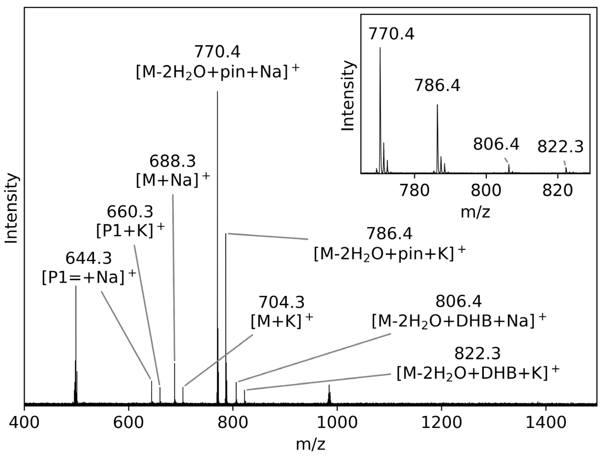

G

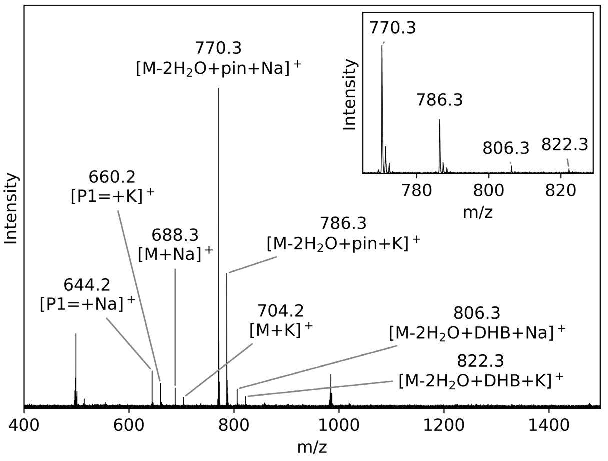

H

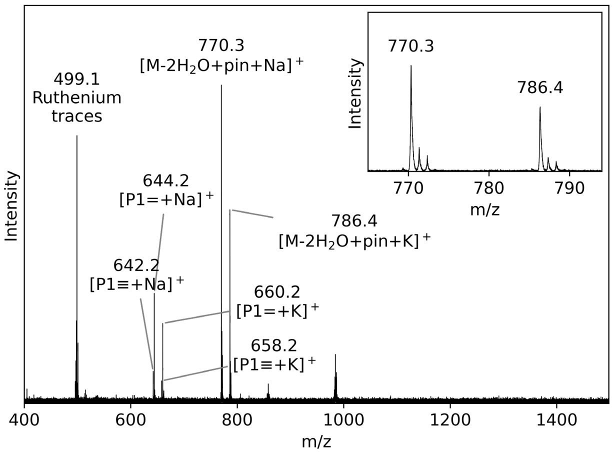

I

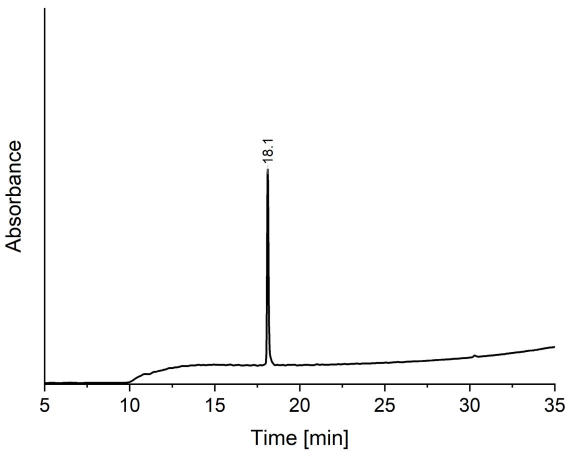

J

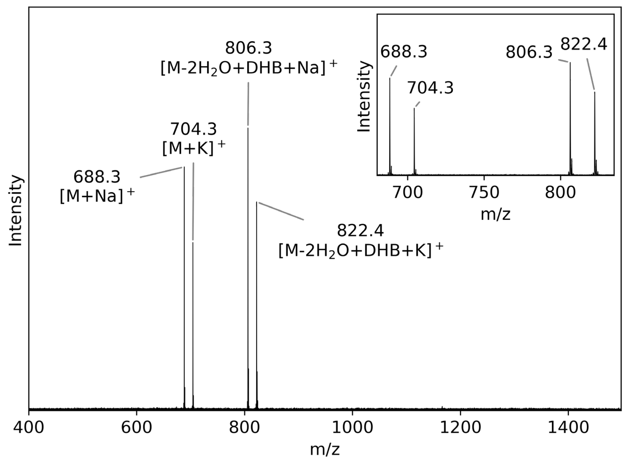

Figure S47: **P1=B(OH)$_2$** [CpRu(PPh$_3$)$_2$Cl]- and [Cp*Ru(PPh$_3$)$_2$Cl]-catalyzed hydroboration. A) Analytical HPLC (220 nm, column B) of entry 5 to 11 referring to conditions of Table 3. B) MALDI-TOF MS spectrum of crude peptide of entry 5. C) MALDI-TOF MS spectrum of crude peptide of entry 6. D) MALDI-TOF MS spectrum of crude peptide of entry 7. E) MALDI-TOF MS spectrum of crude peptide of entry 8. F) MALDI-TOF MS spectrum of crude peptide of entry 9. G) MALDI-TOF MS spectrum of crude peptide of entry 10. H) MALDI-TOF MS spectrum of crude peptide of entry 11. I) Analytical HPLC (220 nm, column B) of purified peptide. H) MALDI-TOF MS spectrum of purified peptide. Calculated mass: [**P1≡**+Na]$^+$ 642.3, [**P1–**+Na]$^+$

646.3, [**P1≡** +K]+ 658.2, [**P1–**+K]+ 662.3, [M+Na]+ 688.3, [M+K]+ 704.3, [M-2H_2O+pin+Na]+ 770.4, [M-2H_2O+pin+K]+ 786.3, [M-2H_2O+DHB+Na]+ 806.3, [M-2H_2O+DHB+K]+ 822.3.

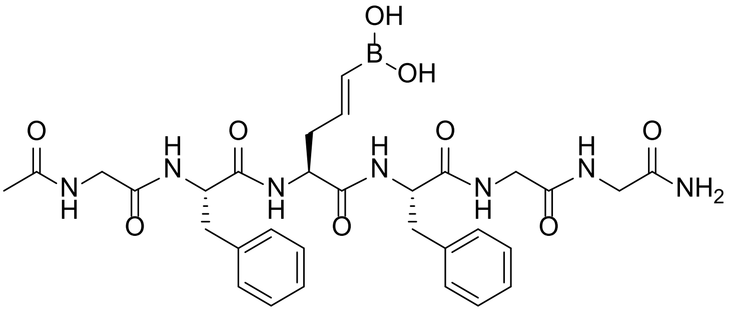

^{1}H-NMR (400 MHz, DMSO-d_6, 295 K):
δ [ppm] = 7.25 − 7.18 (m, 10H, H-Aryl Phe), 6.32 (dt, $^3J_{HH}$ = 17.8 Hz, 6.6 Hz, 1H, H-Alkene), 5.40 (d, $^3J_{HH}$ = 17.9 Hz, 1H, H-Alkene), 4.54 − 4.48 (m, 2H, α-CH Phe), 4.31 − 4.26 (m, 1H, α-CH X), 3.73 − 3.63 (m, 6H, CH_2 Gly), 3.07 − 2.95 (m, 2H, β-CH_2 Phe), 2.89 − 2.66 (m, 2H, β-CH_2 Phe), 2.48 – 2.28 (m, 2H, β-CH_2 -X) 1.81 (s, 3H, Ac)

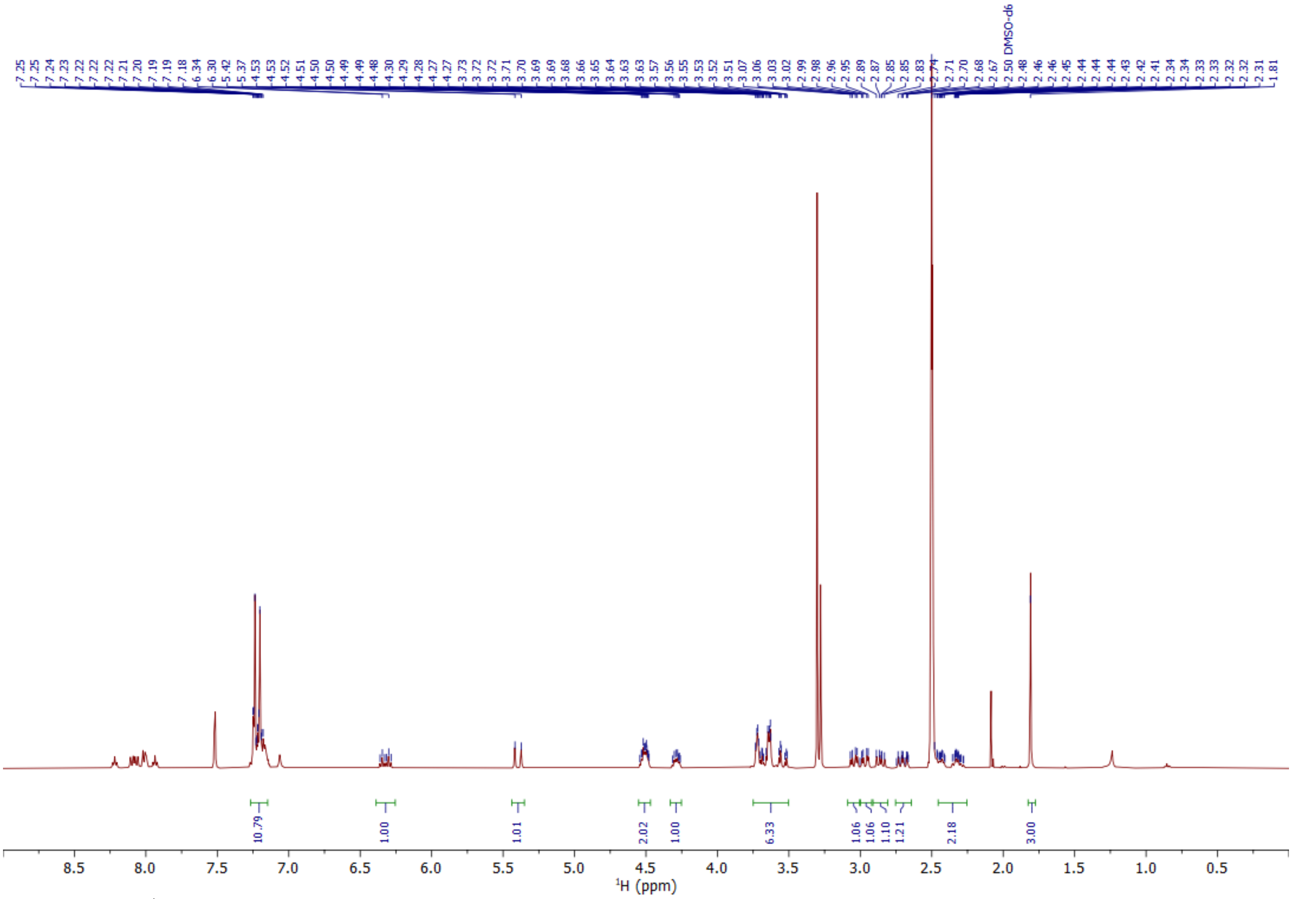

Figure S48: ^{1}H-NMR (400 MHz, DMSO-d_6, 295 K) of **P1=B(OH)₂**, prepared by [Cp*Ru(PPh_3)$_2$Cl]-catalyzed hydroboration. Peaks at 7.5-8.5 ppm are from incomplete hydrogen-deuterium exchange of peptide backbone amide protons.

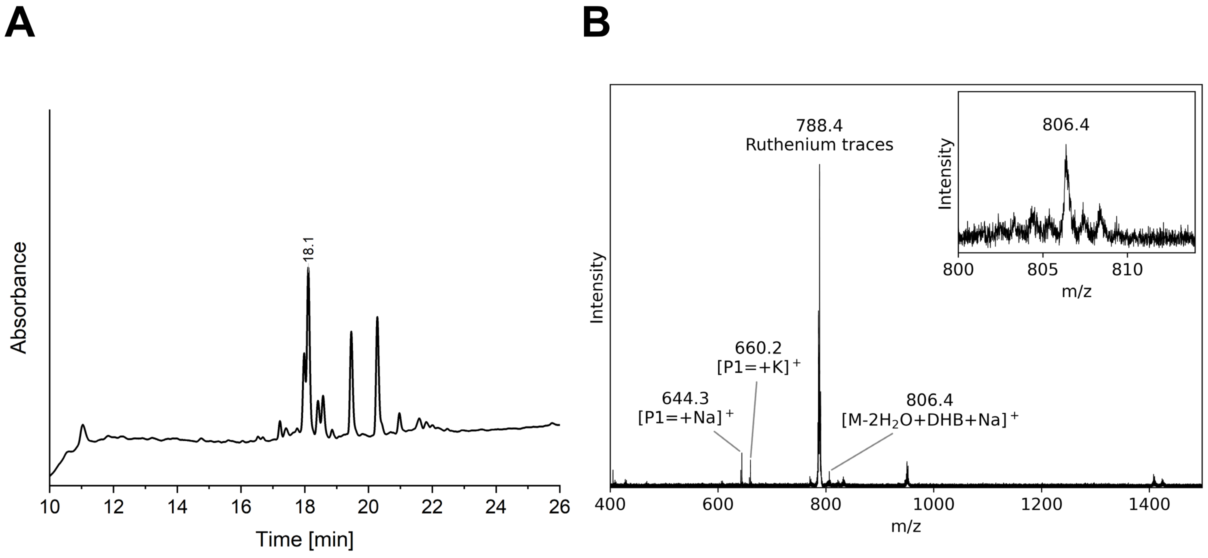

Figure S49: Attempt of hydroboration of **P1≡** with $[CpRu(MeCN)_3]PF_6$. A) Analytical HPLC (220 nm, column B) of crude peptide. B) MALDI-TOF MS spectrum of crude peptide. Calculated mass: [**P1=**+Na]$^+$ 644.3, [**P1=**+K]$^+$ 660.3, [M-2H_2O+DHB+Na]$^+$ 806.3.

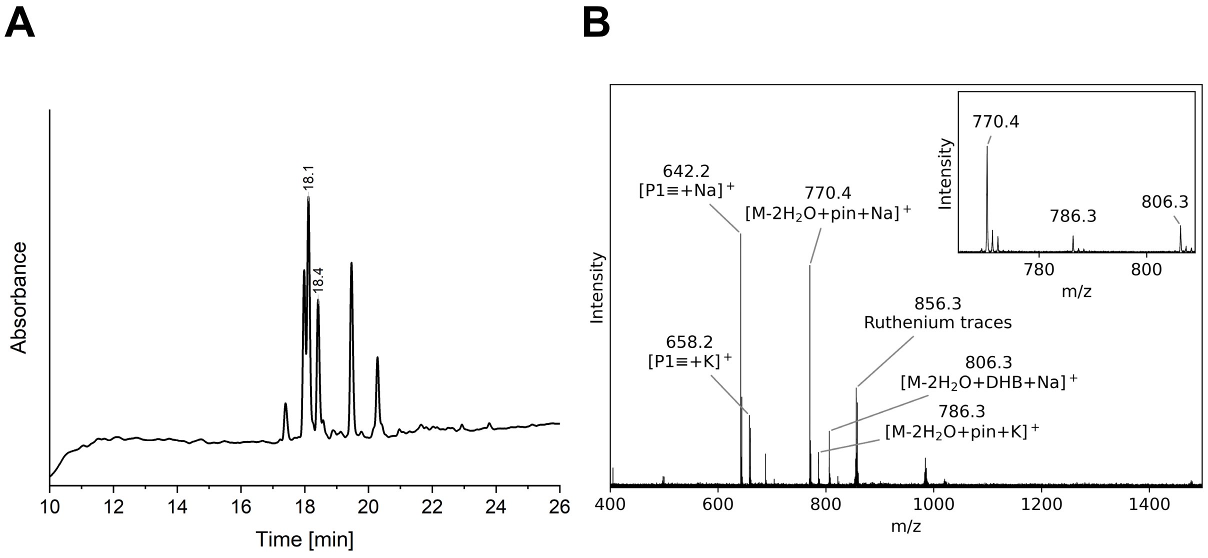

Figure S50: Attempt of hydroboration of **P1≡** with $[Cp^*Ru(MeCN)_3]PF_6$. A) Analytical HPLC (220 nm, column B) of crude peptide. B) MALDI-TOF MS spectrum of crude peptide. Calculated mass: [**P1≡**+Na]$^+$ 642.3, [**P1≡**+K]$^+$ 658.2, [M-2H_2O+pin+Na]$^+$ 770.4, [M-2H_2O+pin+K]$^+$ 786.3, [M-2H_2O+DHB+Na]$^+$ 806.3, [M-2H_2O+DHB+K]$^+$ 822.3.

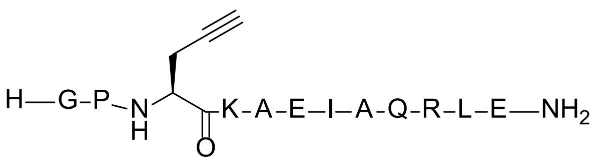

P2≡

Chemical Formula: $C_{57}H_{96}N_{18}O_{17}$

Exact Mass: 1304.72

Molecular Weight: 1305.50

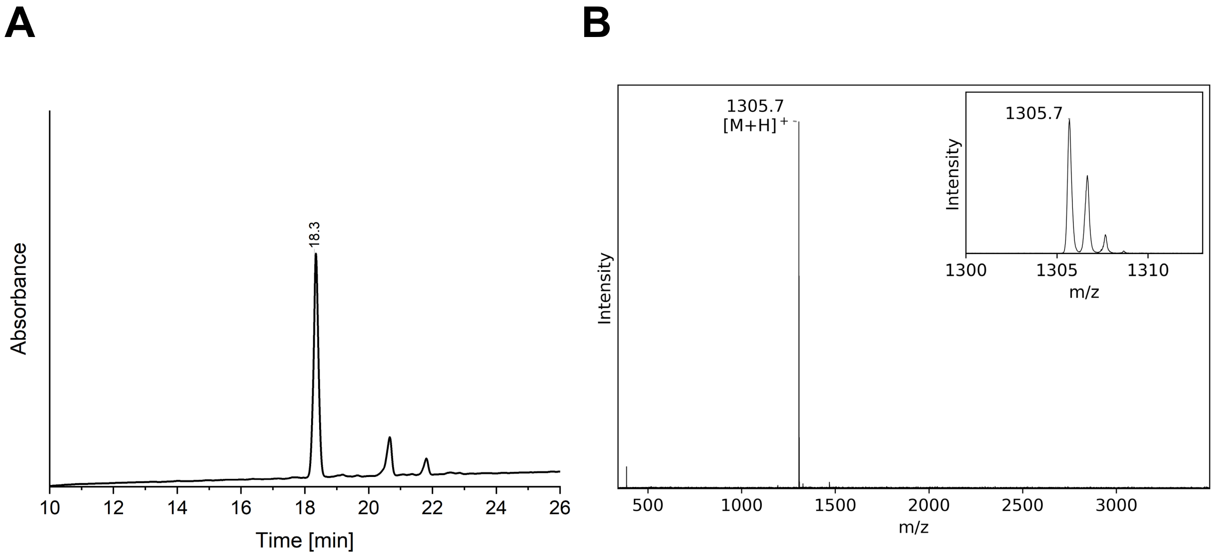

Figure S51: **P2≡** A) Analytical HPLC (220 nm, column A) of crude peptide. B) MALDI-TOF MS spectrum of crude peptide. Calculated mass: $[M+H]^+$ 1305.7.

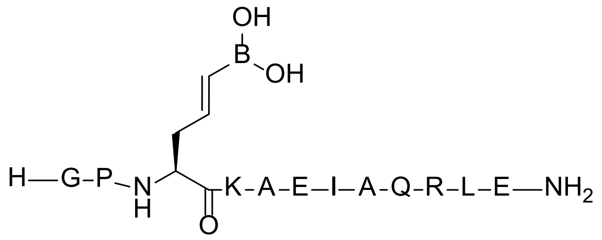

P2=B(OH)$_2$

Chemical Formula: $C_{57}H_{99}BN_{18}O_{19}$

Exact Mass: 1350.74

Molecular Weight: 1351.34

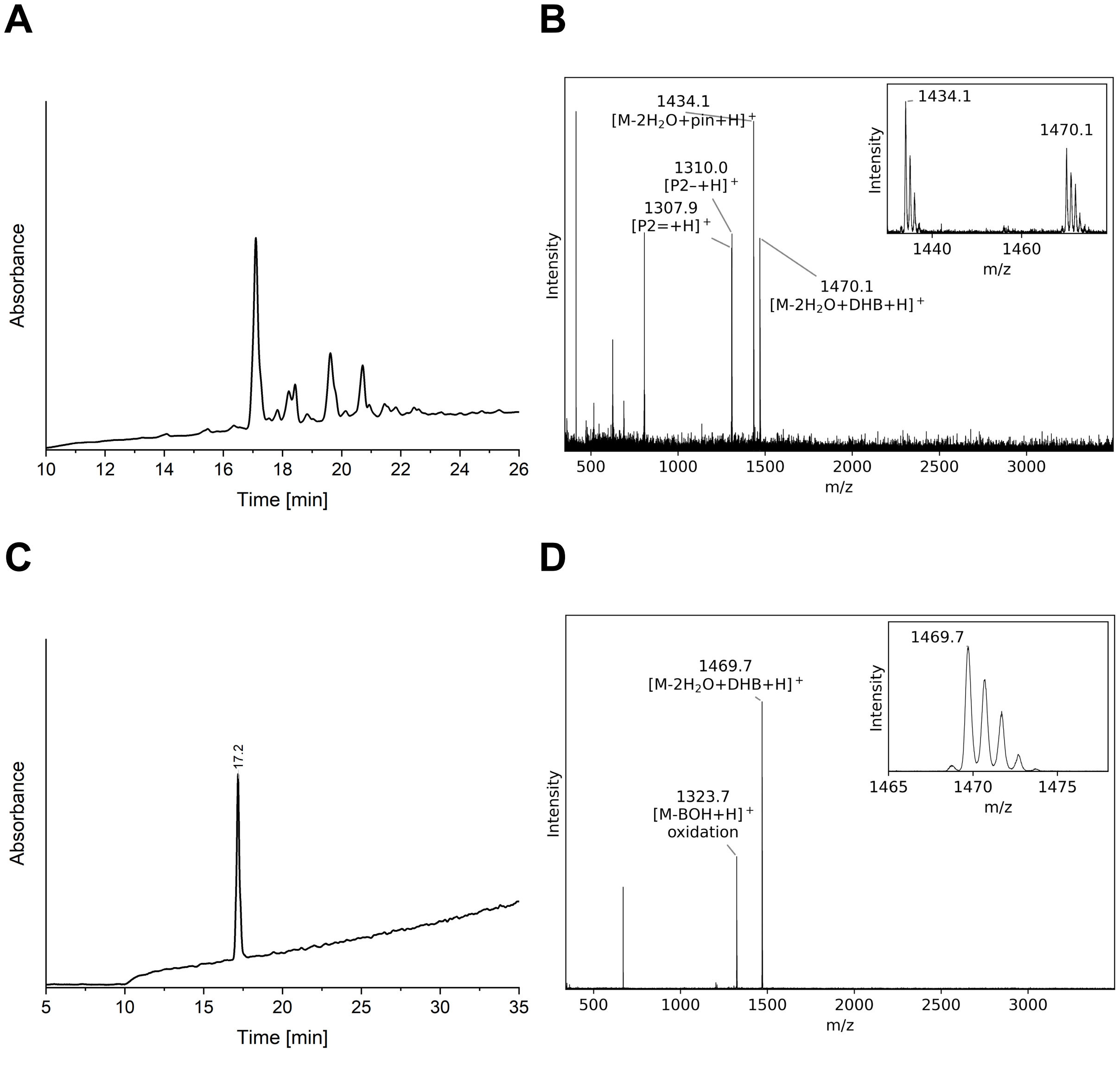

Figure S52: **P2=B(OH)$_2$** [Ru(CO)(Cl)H(PPh$_3$)$_3$]-catalyzed hydroboration. A) Analytical HPLC (220 nm, column A) of crude peptide. B) MALDI-TOF MS spectrum of crude peptide. C) Analytical HPLC (220 nm, column A) of purified peptide. D) MALDI-TOF MS spectrum of purified peptide. Calculated mass: [**P2=**+H]$^+$ 1307.7, [**P2–**+H]$^+$ 1309.8, [M-BOH+H]$^+$ 1323.7 [M-2H$_2$O+pin+H]$^+$ 1433.8, [M-2H$_2$O+DHB+H]$^+$ 1469.8.

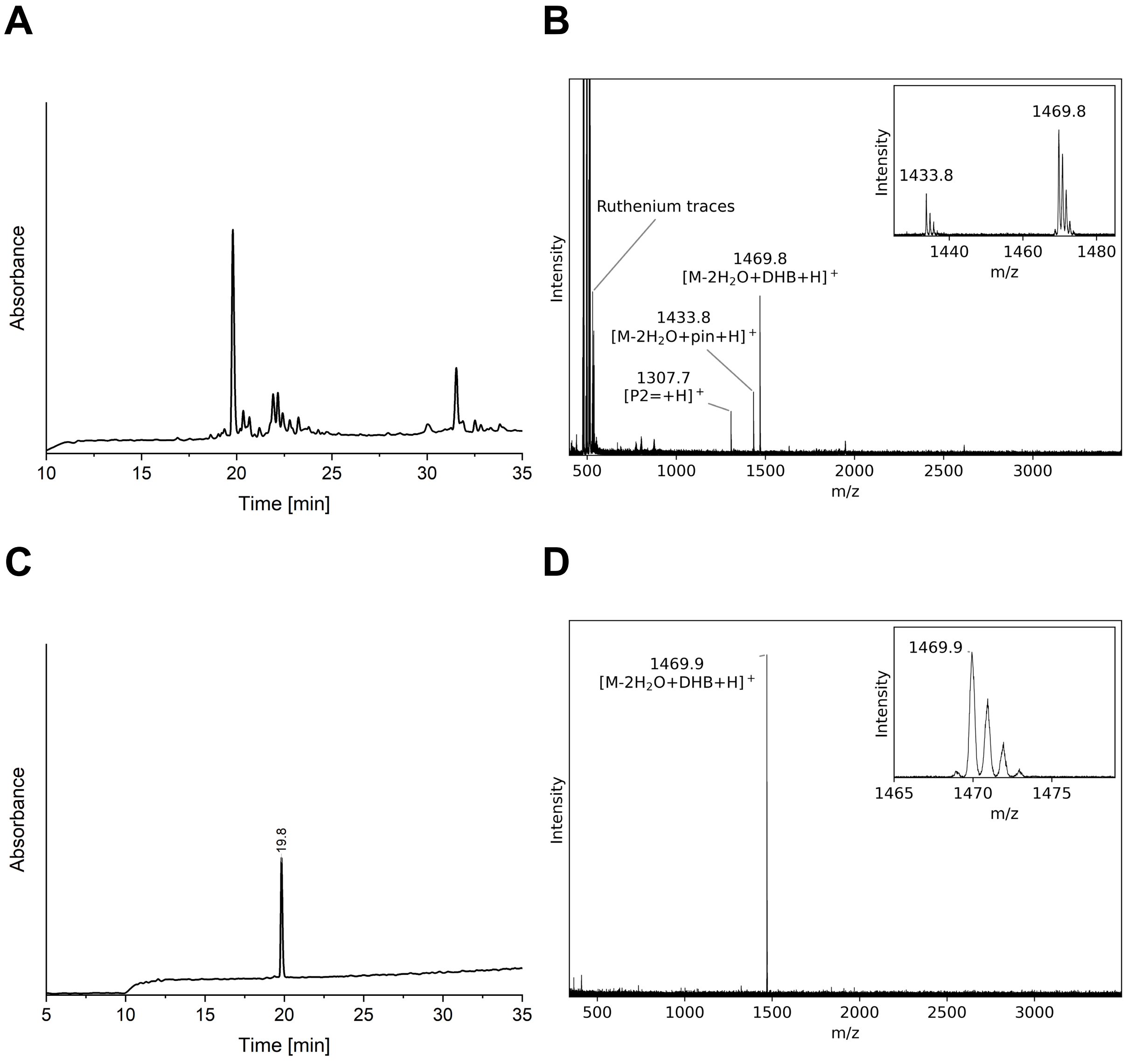

Figure S53: **$P2=B(OH)_2$** $[Cp^*Ru(PPh_3)_2Cl]$-catalyzed hydroboration. A) Analytical HPLC (220 nm, column B) of crude peptide. B) MALDI-TOF MS spectrum of crude peptide. C) Analytical HPLC (220 nm, column B) of purified peptide. D) MALDI-TOF MS spectrum of purified peptide. Calculated mass: [**P2=**+H]$^+$ 1307.7, [M-$2H_2O$+pin+H]$^+$ 1433.8, [M-$2H_2O$+DHB+H]$^+$ 1469.8.

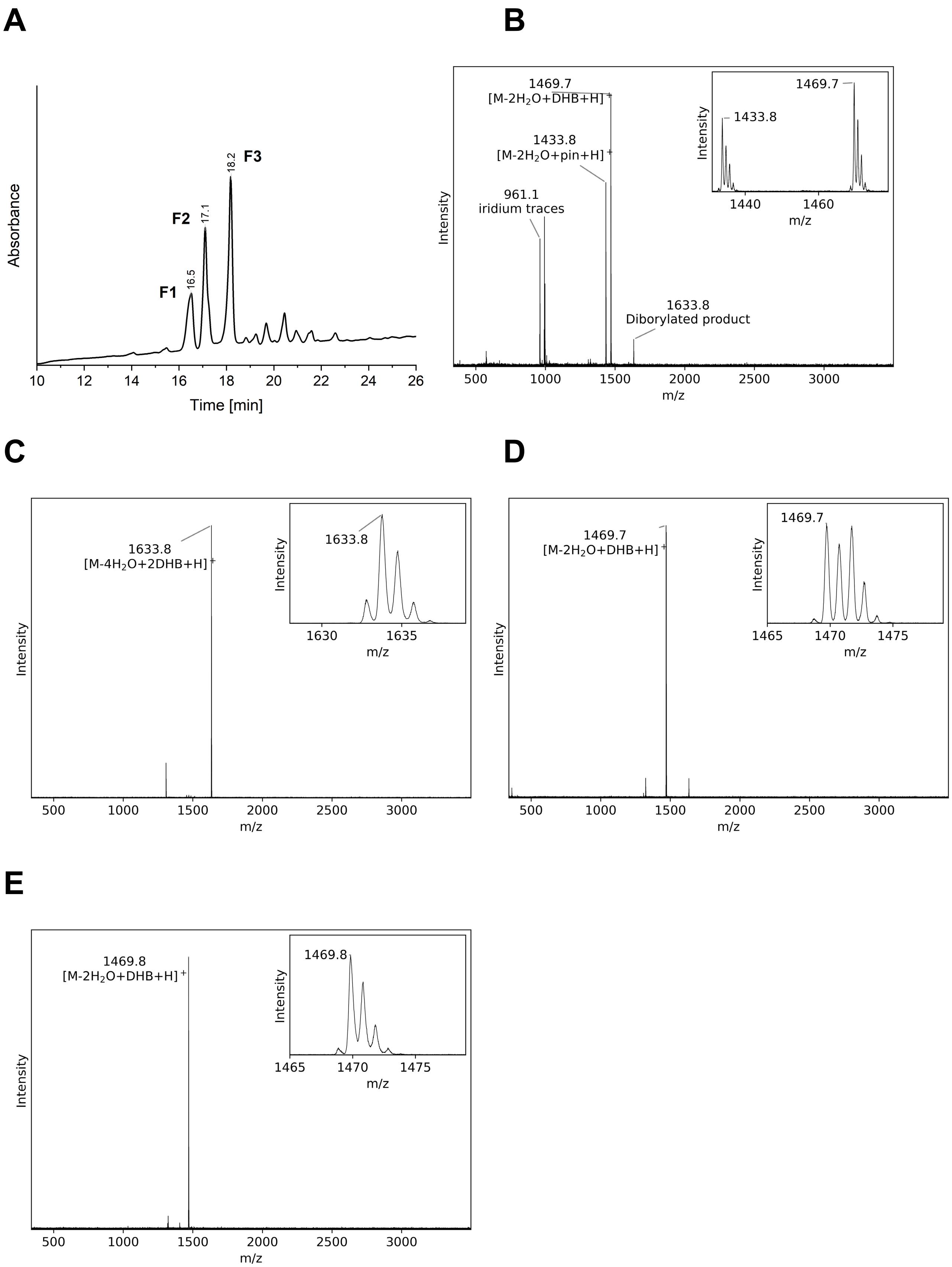

Figure S54: **P2=B(OH)$_2$** Iridium-catalyzed hydroboration. A) Analytical HPLC (220 nm, column A) of crude peptide. B) MALDI-TOF MS spectrum of crude peptide. C) MALDI-TOF MS spectrum of purified fraction **F1**. M refers to the diborylated product. Calculated mass: [M-4H$_2$O+2DHB+H]$^+$ 1633.8. D) MALDI-TOF MS spectrum of purified fraction **F2**. E) MALDI-TOF MS spectrum of purified fraction **F3**. Calculated mass: [M-2H$_2$O+pin+H]$^+$ 1433.8, [M-2H$_2$O+DHB+H]$^+$ 1469.8.

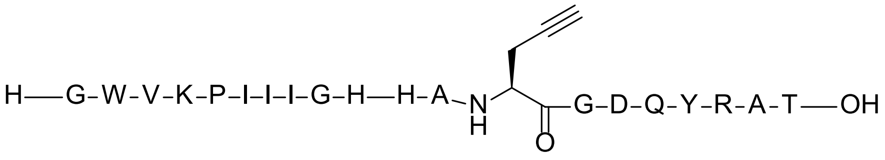

P3≡

Chemical Formula: $C_{102}H_{152}N_{30}O_{26}$

Exact Mass: 2213.15

Molecular Weight: 2214.52

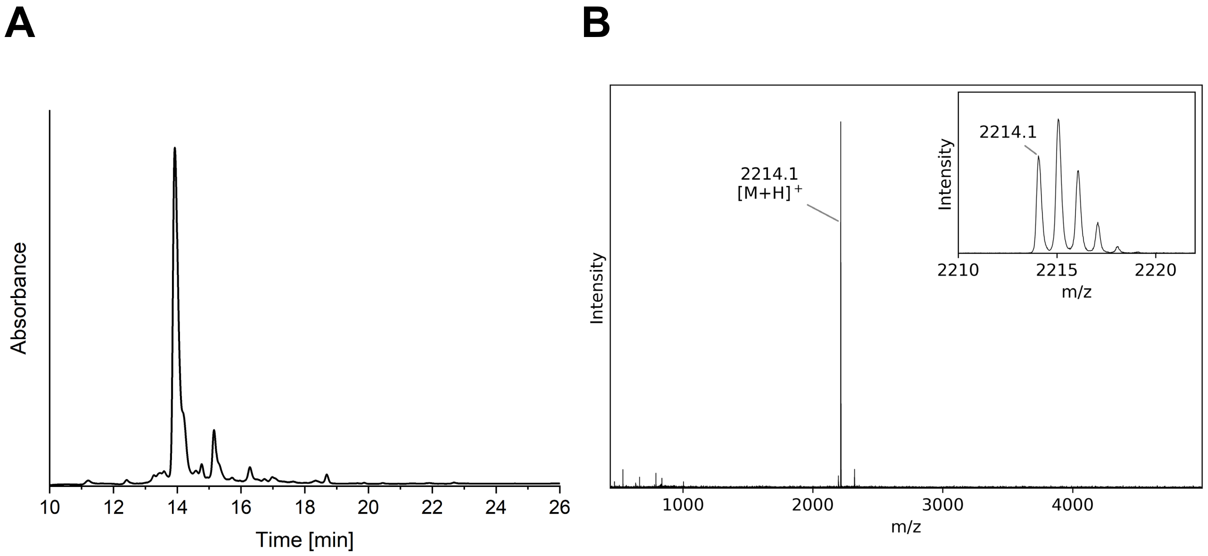

Figure S55: **P3≡** A) Analytical HPLC (220 nm, column B) of crude peptide. B) MALDI-TOF MS spectrum of crude peptide. Calculated mass: $[M+H]^+$ 2214.2.

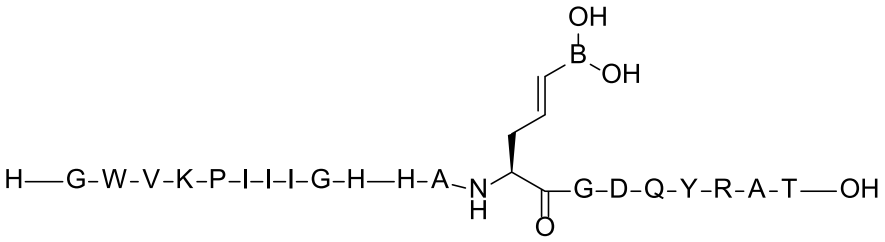

P3=B(OH)$_2$

Chemical Formula: $C_{102}H_{155}BN_{30}O_{28}$

Exact Mass: 2259.17

Molecular Weight: 2260.35

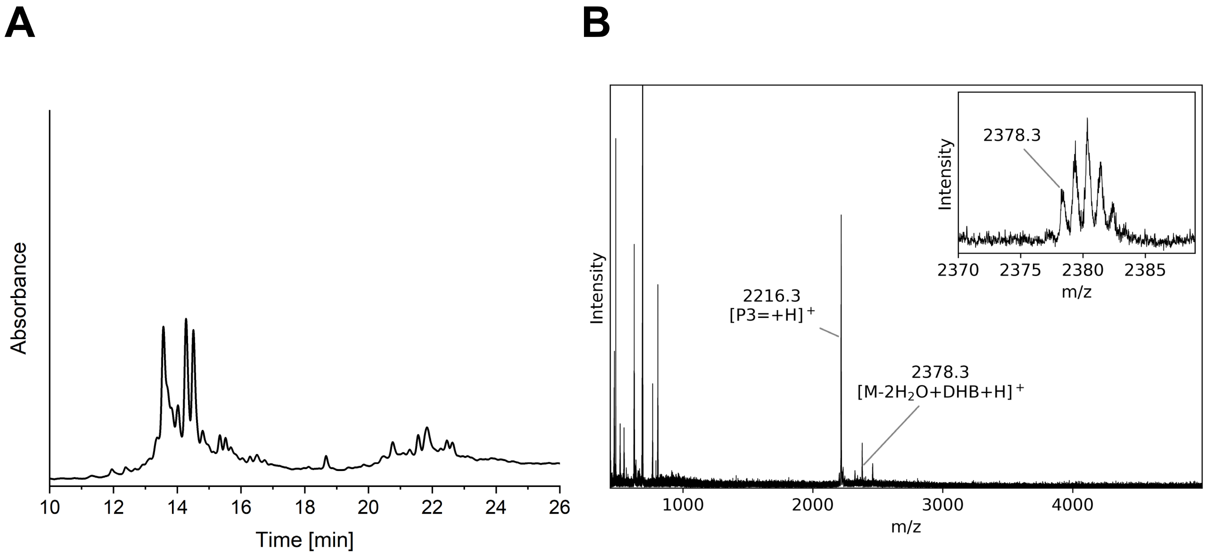

Figure S56 **P3=B(OH)$_2$** [Ru(CO)(Cl)H(PPh$_3$)$_3$]-catalyzed hydroboration. A) Analytical HPLC (220 nm, column B) of crude peptide. B) MALDI-TOF MS spectrum of crude peptide. Calculated mass: [**P3=**+H]$^+$ 2216.2, [M-2H$_2$O+DHB+H]$^+$ 2378.2.

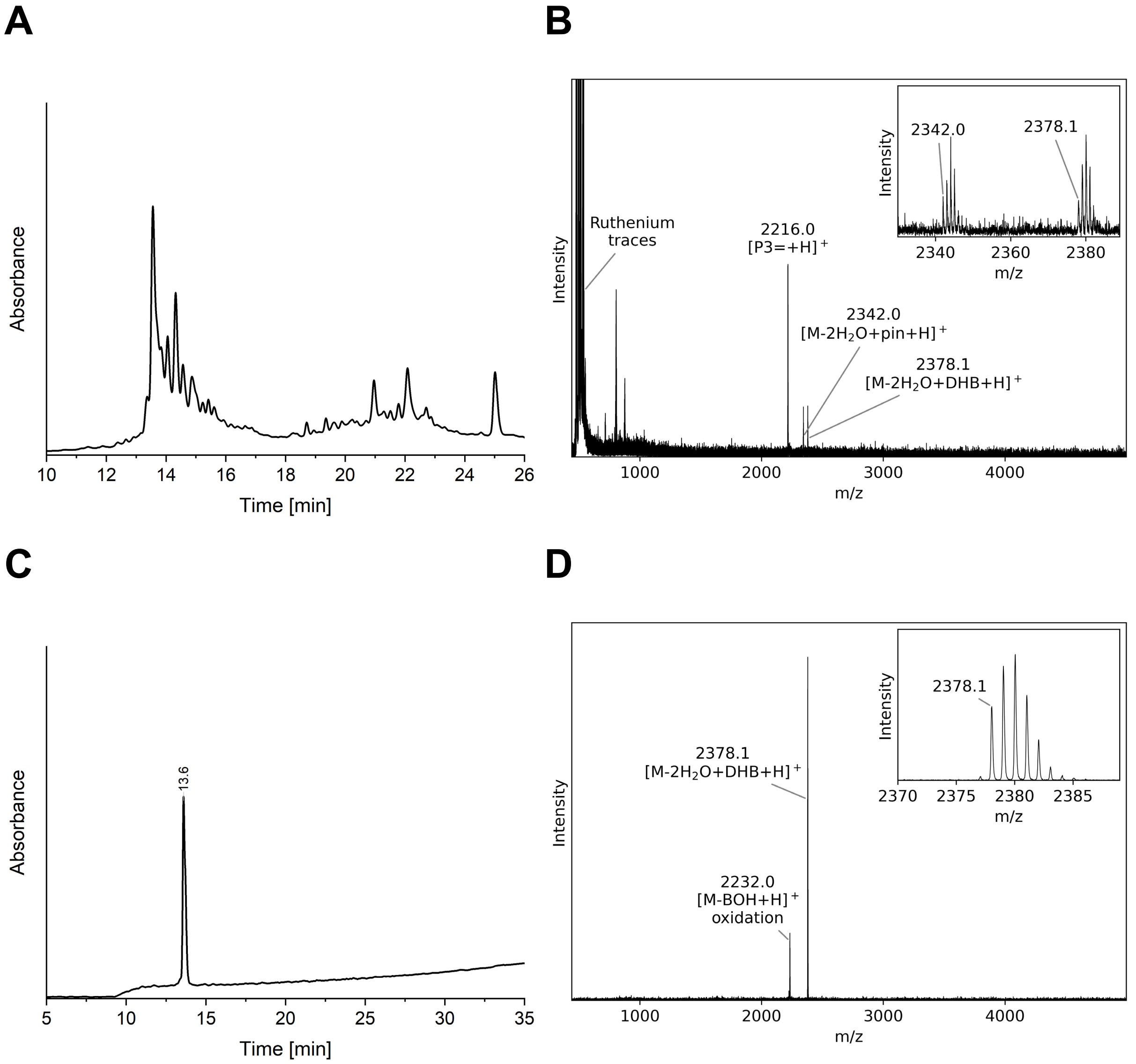

Figure S57: **P3=B(OH)$_2$** [Cp*Ru(PPh$_3$)$_2$Cl]-catalyzed hydroboration. A) Analytical HPLC (220 nm, column B) of crude peptide. B) MALDI-TOF MS spectrum of crude peptide. C) Analytical HPLC (220 nm, column B) of purified peptide. D) MALDI-TOF MS spectrum of purified peptide. Calculated mass: [**P3=**+H]$^+$ 2216.2, [M-BOH+H]$^+$ 2232.2, [M-2H_2O+pin+H]$^+$ 2342.3, [M-2H_2O+DHB+H]$^+$ 2378.2.

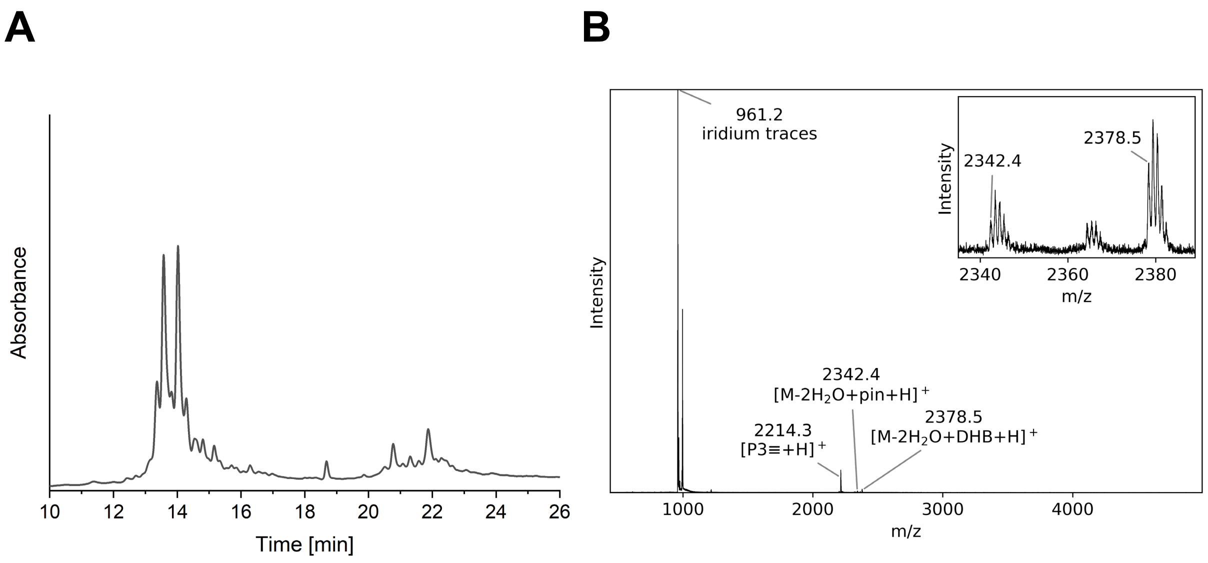

Figure S58: **P3=B(OH)$_2$** Iridium-catalyzed hydroboration. A) Analytical HPLC (220 nm, column A) of crude peptide. B) MALDI-TOF MS spectrum of crude peptide. Calculated mass: [**P3=**+H]$^+$ 2216.2, [M-2H$_2$O+pin+H]$^+$ 2342.3, [M-2H$_2$O+DHB+H]$^+$ 2378.2.

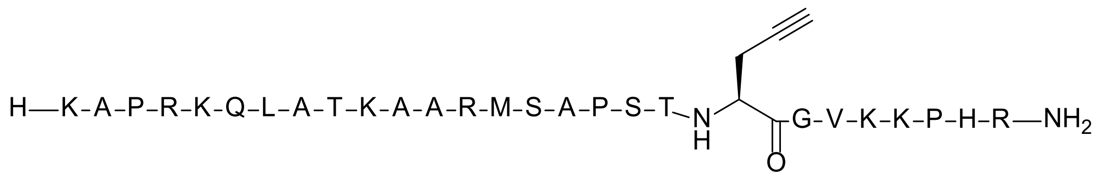

P4≡

Chemical Formula: $C_{126}H_{221}N_{45}O_{32}S$

Exact Mass: 2908.68

Molecular Weight: 2910.50

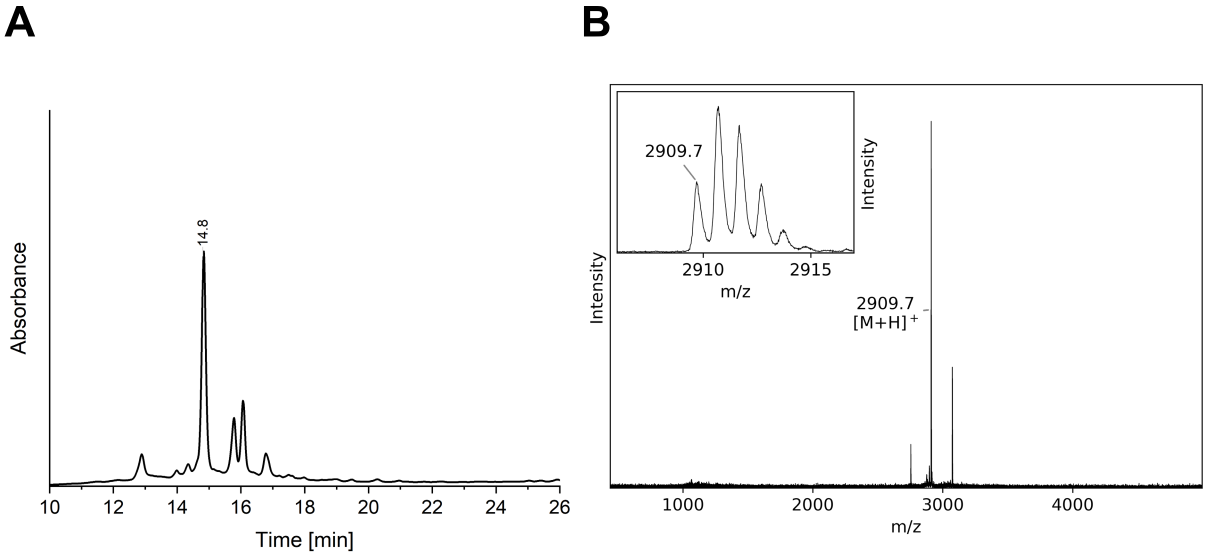

Figure S59: **P4≡** A) Analytical HPLC (220 nm, column A) of crude peptide. B) MALDI-TOF MS spectrum of crude peptide. Calculated mass: $[M+H]^+$ 2909.7.

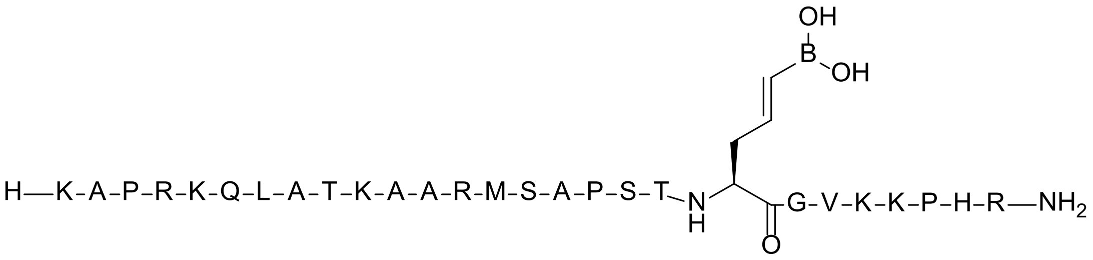

P4=B(OH)$_2$

Chemical Formula: $C_{126}H_{224}BN_{45}O_{34}S$

Exact Mass: 2954.70

Molecular Weight: 2956.33

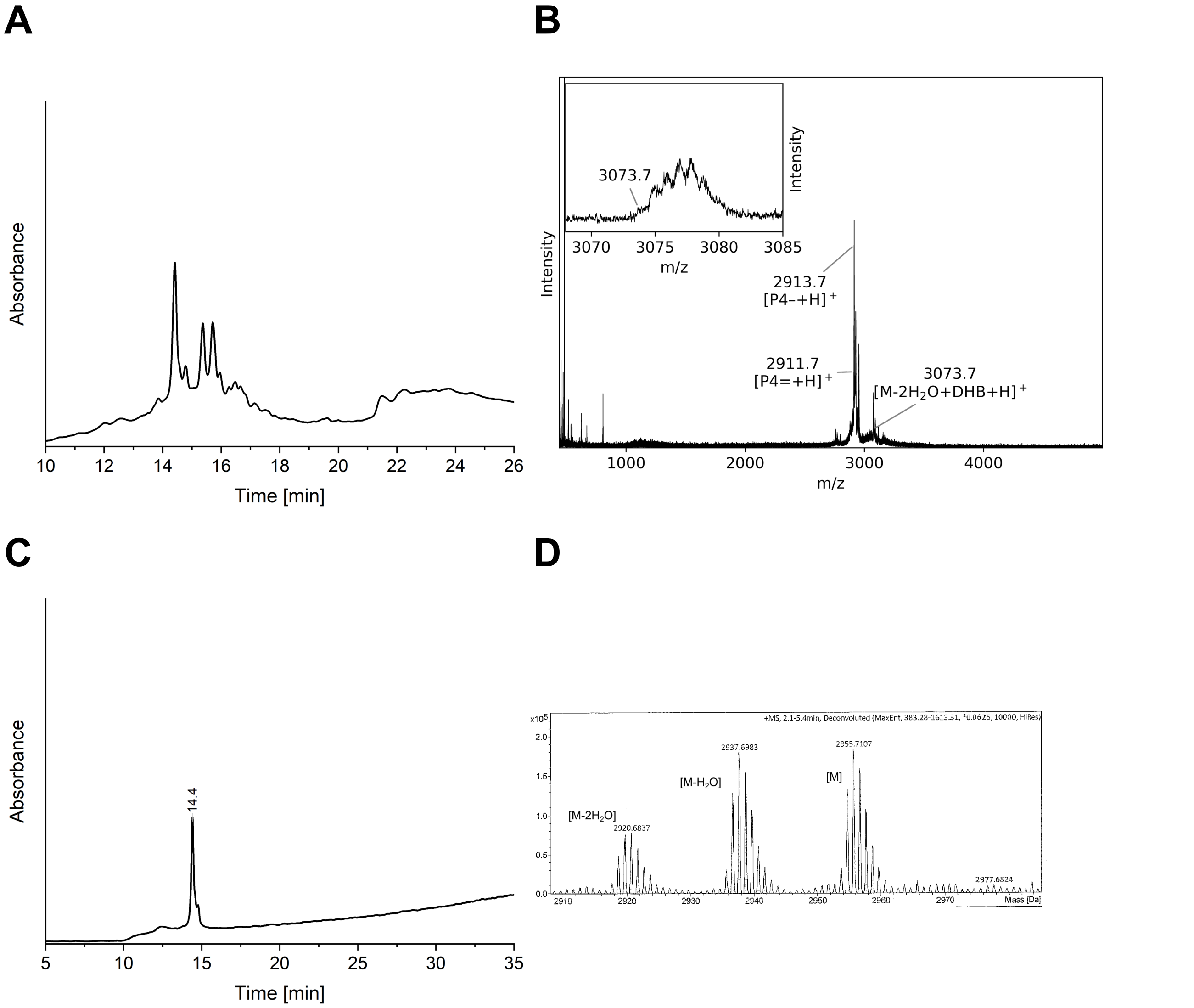

Figure S60: **P4=B(OH)$_2$** [Ru(CO)(Cl)H(PPh$_3$)$_3$]-catalyzed hydroboration. A) Analytical HPLC (220 nm, column A) of crude peptide. B) MALDI-TOF MS spectrum of crude peptide. C) Analytical HPLC (220 nm, column A) of purified peptide. D) Deconvoluted ESI HR-MS spectrum of purified peptide. Full HR-MS spectrum in section 3. Calculated mass: [**P4=**+H]$^+$ 2911.7, [**P4–**+H]$^+$ 2913.7, [M-2H$_2$O]$^+$ 2918.7, [M-H$_2$O] 2936.7, [M] 2954.7, [M-2H$_2$O+DHB+H]$^+$ 3072.7.

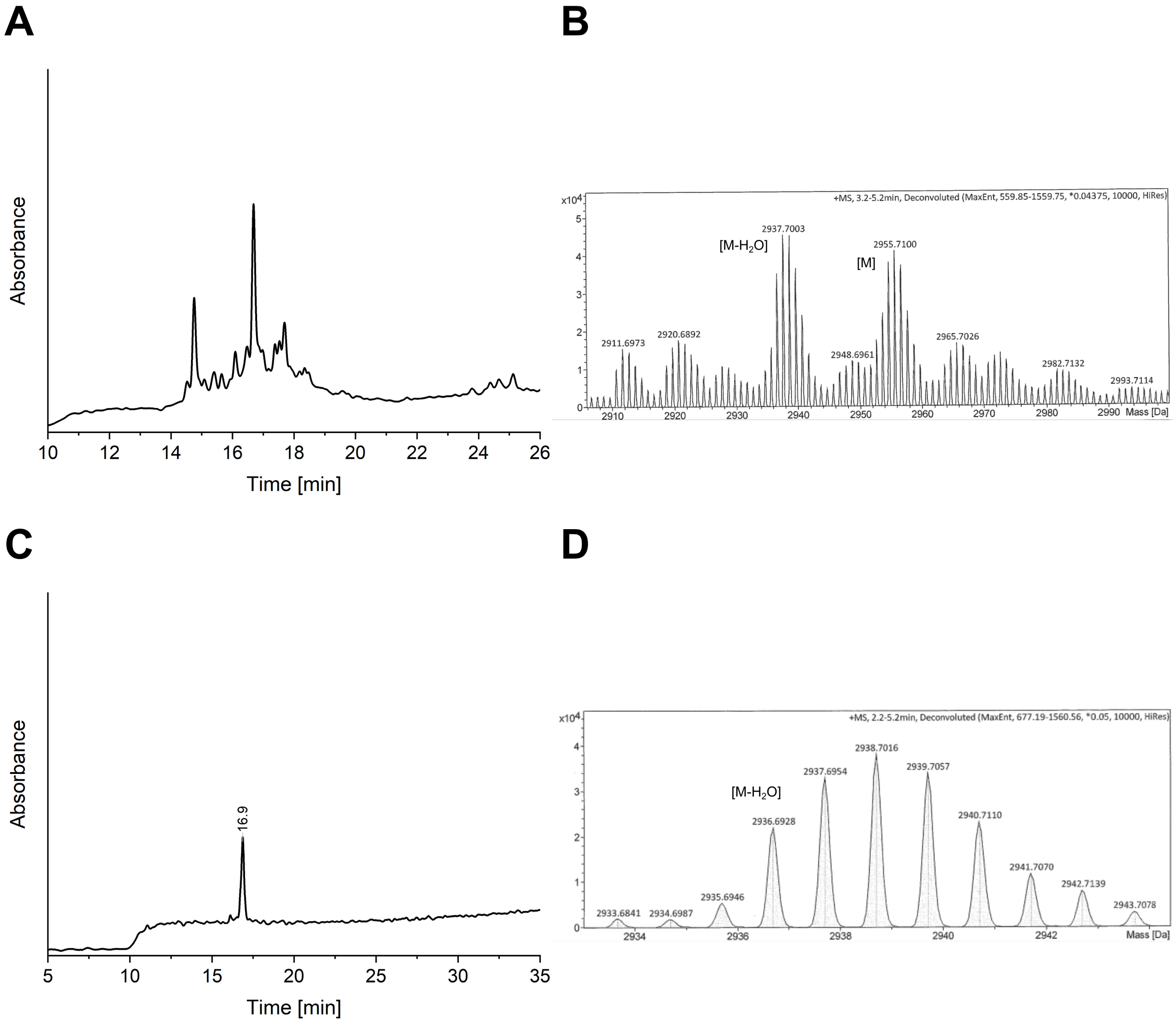

Figure S61: **P4=B(OH)$_2$** [Cp*Ru(PPh$_3$)$_2$Cl]-catalyzed hydroboration. A) Analytical HPLC (220 nm, column B) of crude peptide. B) Deconvoluted ESI HR-MS spectrum of crude peptide. C) Analytical HPLC (220 nm, column B) of purified peptide. D) Deconvoluted ESI HR-MS spectrum of purified peptide. Full HR-MS spectrum in section 3. Calculated mass: [M-H$_2$O] 2936.7, [M] 2954.7.

A B

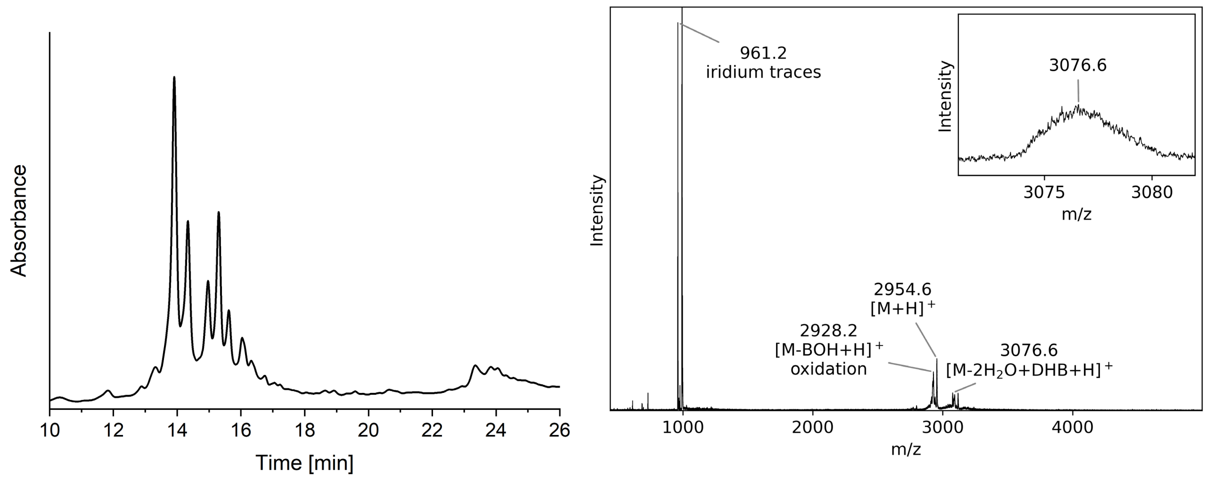

Figure S62: **P4=B(OH)$_2$** Iridium-catalyzed hydroboration. A) Analytical HPLC (220 nm, column A) of crude peptide. B) MALDI-TOF MS spectrum of crude peptide. Calculated mass: $[M\text{-}BOH+H]^+$ 2926.7, $[M+H]^+$ 2955.7, $[M\text{-}2H_2O+DHB+H]^+$ 3072.7.

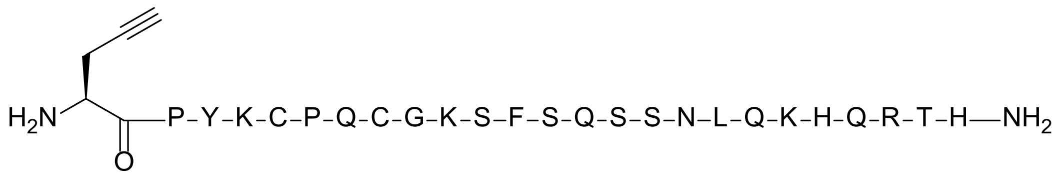

P5≡

Chemical Formula: $C_{123}H_{191}N_{41}O_{36}S_2$

Exact Mass: 2882.38

Molecular Weight: 2884.25

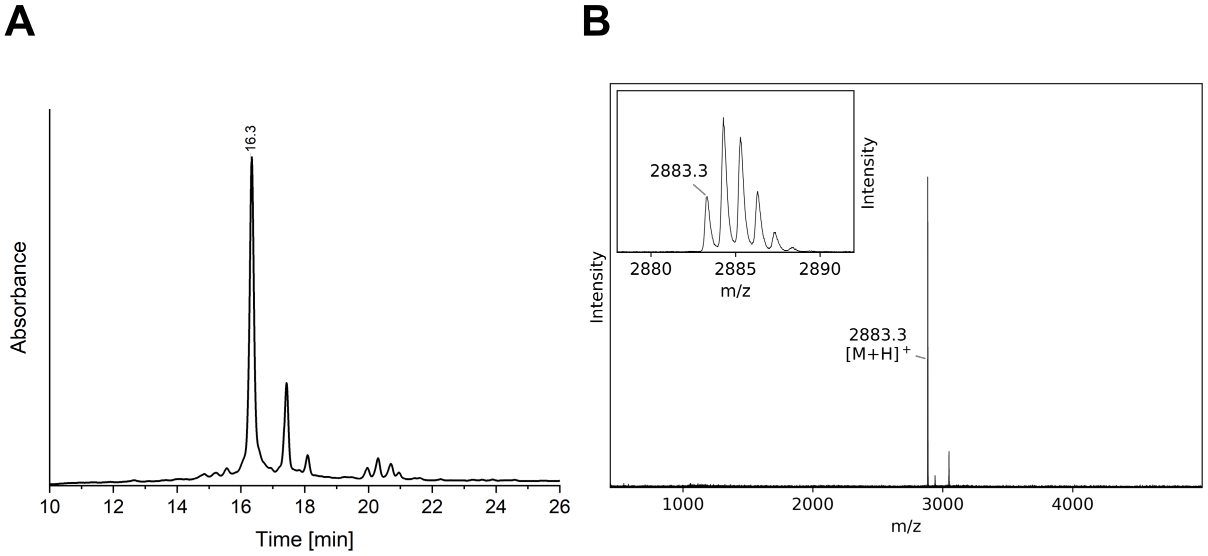

Figure S63: **P5≡** A) Analytical HPLC (220 nm, column A) of crude peptide. B) MALDI-TOF MS spectrum of crude peptide. Calculated mass: $[M+H]^+$ 2883.4.

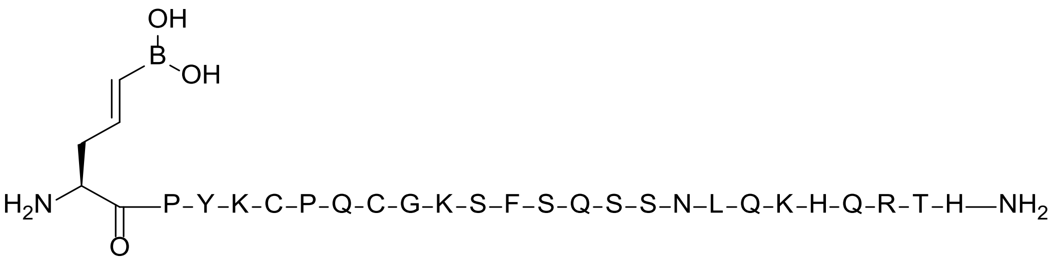

P5=B(OH)$_2$

Chemical Formula: $C_{123}H_{194}BN_{41}O_{38}S_2$

Exact Mass: 2928.40

Molecular Weight: 2930.08

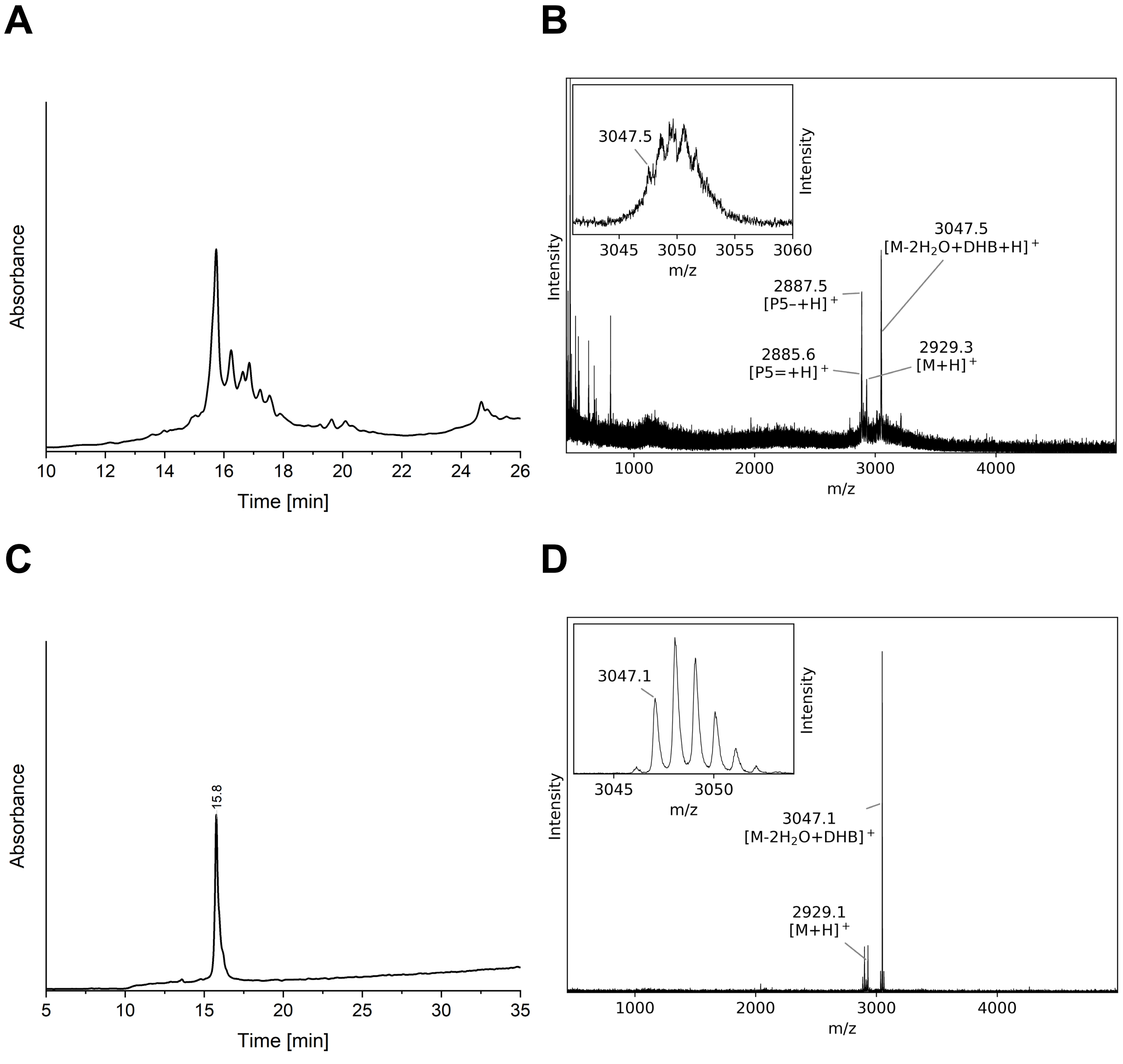

Figure S64 **P5=B(OH)$_2$** [Ru(CO)(Cl)H(PPh$_3$)$_3$]-catalyzed hydroboration. A) Analytical HPLC (220 nm, column A) of crude peptide. B) MALDI-TOF MS spectrum of crude peptide. C) Analytical HPLC (220 nm, column A) of purified peptide. D) MALDI-TOF MS spectrum of purified peptide. Calculated mass: [**P5=**+H]$^+$ 2885.4, [**P5–**+H]$^+$ 2887.4, [M+H]$^+$ 2929.4, [M-2H$_2$O+DHB+H]$^+$ 3047.4.

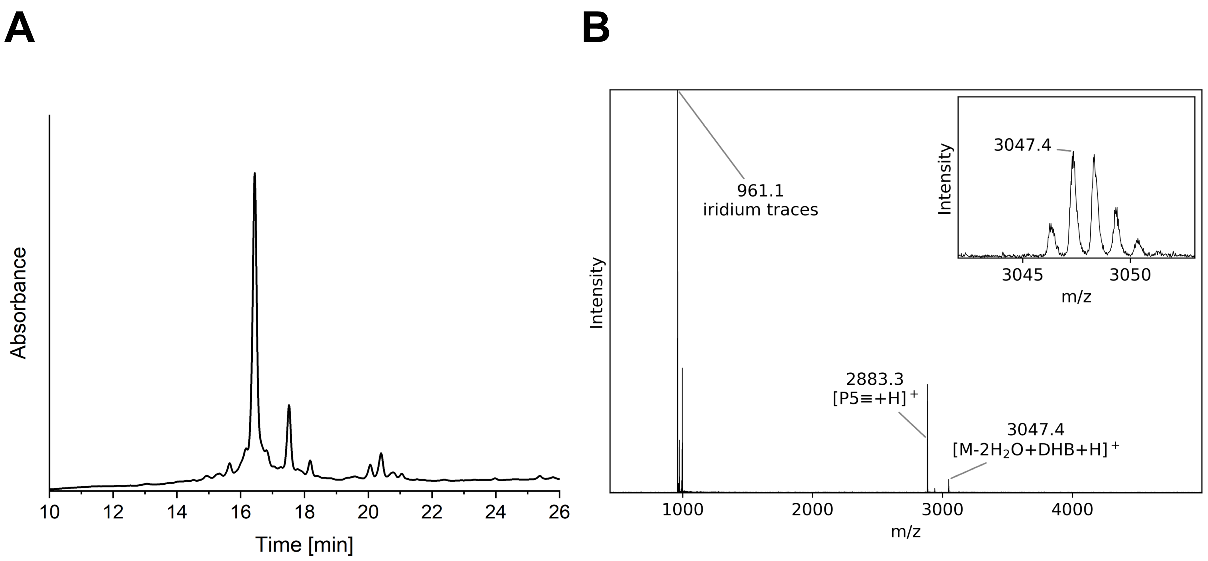

Figure S65: **P5=B(OH)$_2$** Iridium-catalyzed hydroboration. A) Analytical HPLC (220 nm, column A) of crude peptide. B) MALDI-TOF MS spectrum of crude peptide. Calculated mass: [**P5=**+H]$^+$ 2885.4, [M-2H_2O+DHB+H]$^+$ 3047.4.

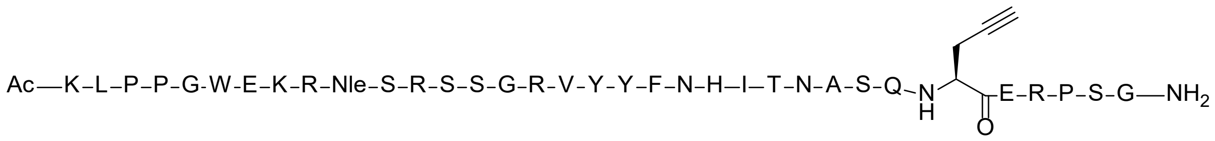

P6≡

Chemical Formula: $C_{176}H_{269}N_{55}O_{50}$

Exact Mass: 3953.02

Molecular Weight: 3955.42

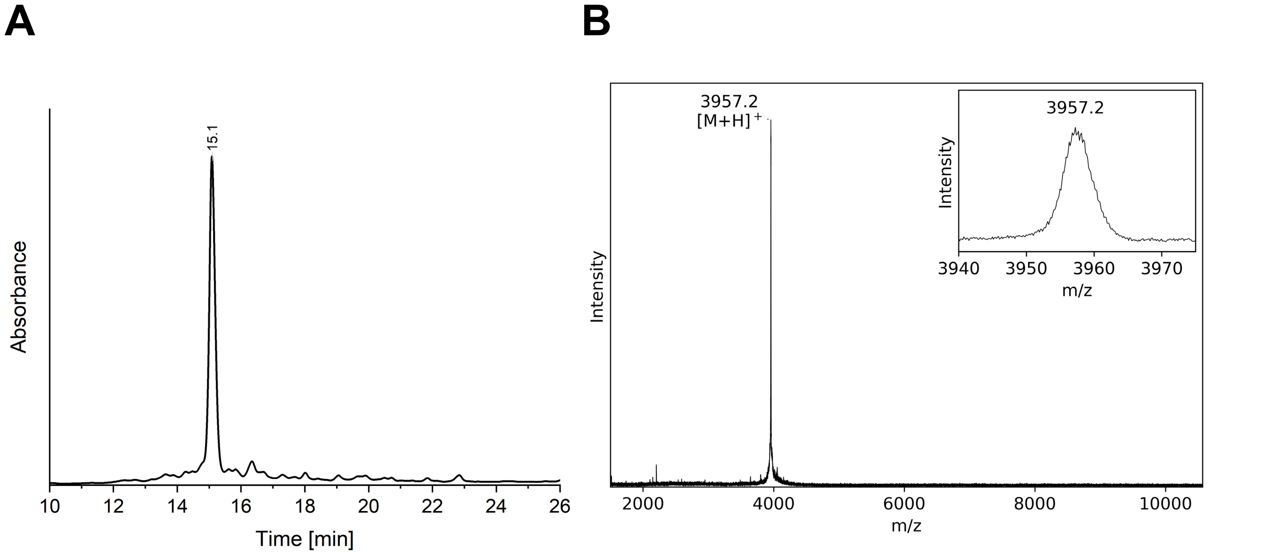

Figure S66: **P6≡** A) Analytical HPLC (220 nm, column A) of crude peptide. B) MALDI-TOF MS spectrum of crude peptide. Calculated mass: $[M+H]^+$ 3954.0.

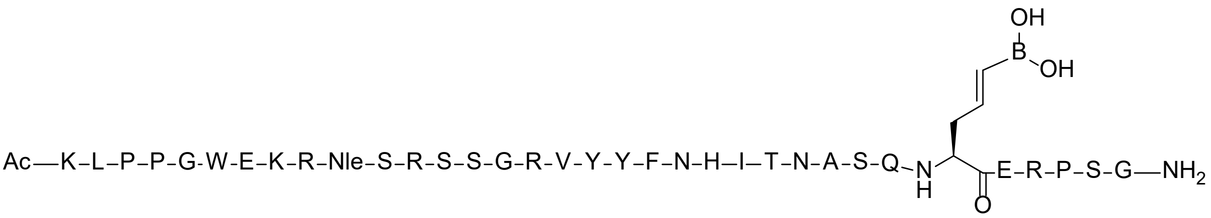

P6=B(OH)$_2$

Chemical Formula: $C_{176}H_{272}BN_{55}O_{52}$

Exact Mass: 3999.04

Molecular Weight: 4001.26

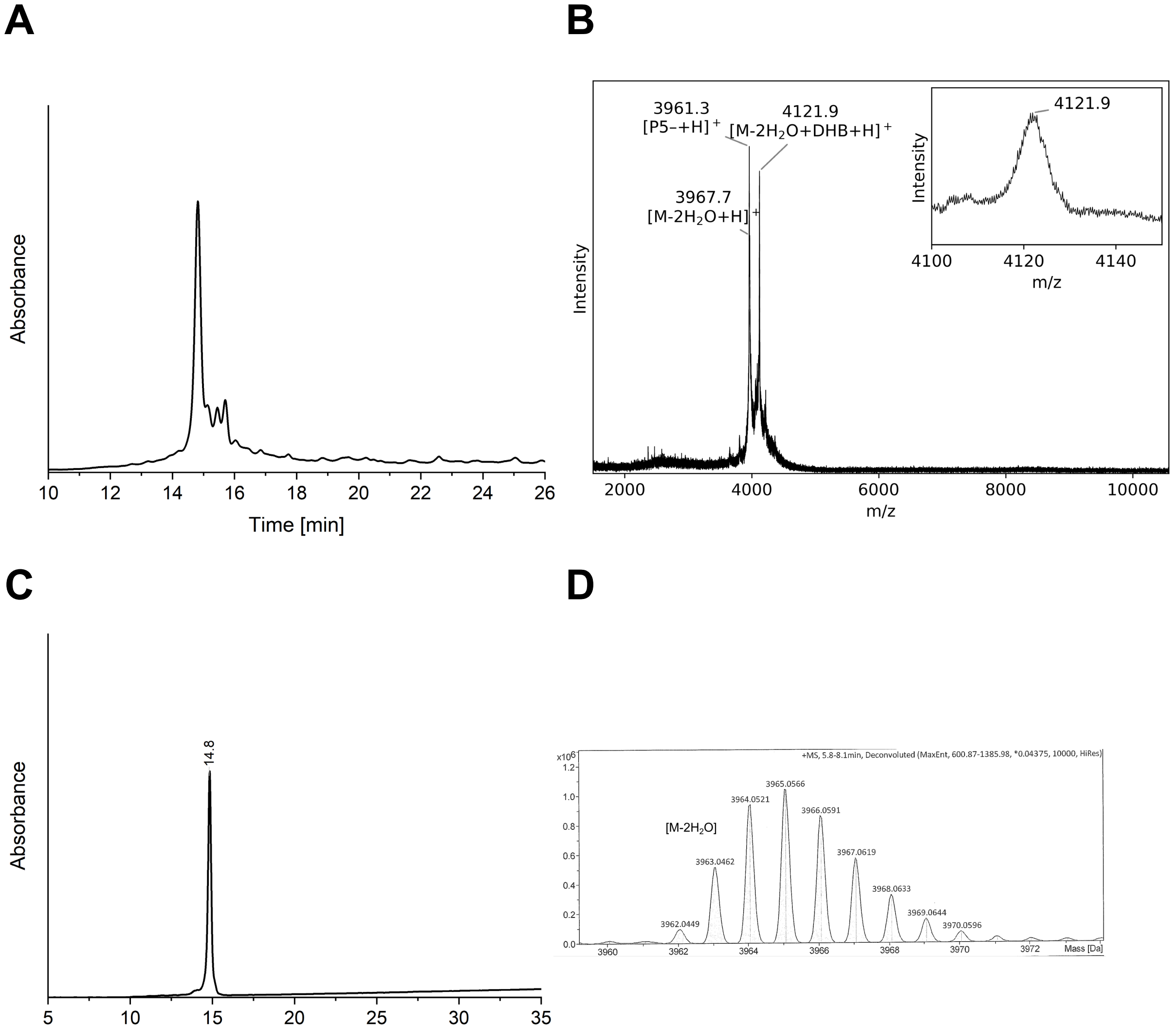

Figure S67: **P6=B(OH)$_2$** [Ru(CO)(Cl)H(PPh$_3$)$_3$]-catalyzed hydroboration. A) Analytical HPLC (220 nm, column A) of crude peptide. B) MALDI-TOF MS spectrum of crude peptide. C) Analytical HPLC (220 nm, column A) of purified peptide. D) Deconvoluted ESI HR-MS spectrum of purified peptide. Full HR-MS spectrum in section 3. Calculated mass: [**P6**–+H]$^+$ 3958.1, [M-2H$_2$O]$^+$ 3963.0, [M-2H$_2$O+H]$^+$ 3964.0, [M-2H$_2$O+DHB+H]$^+$ 4118.1.

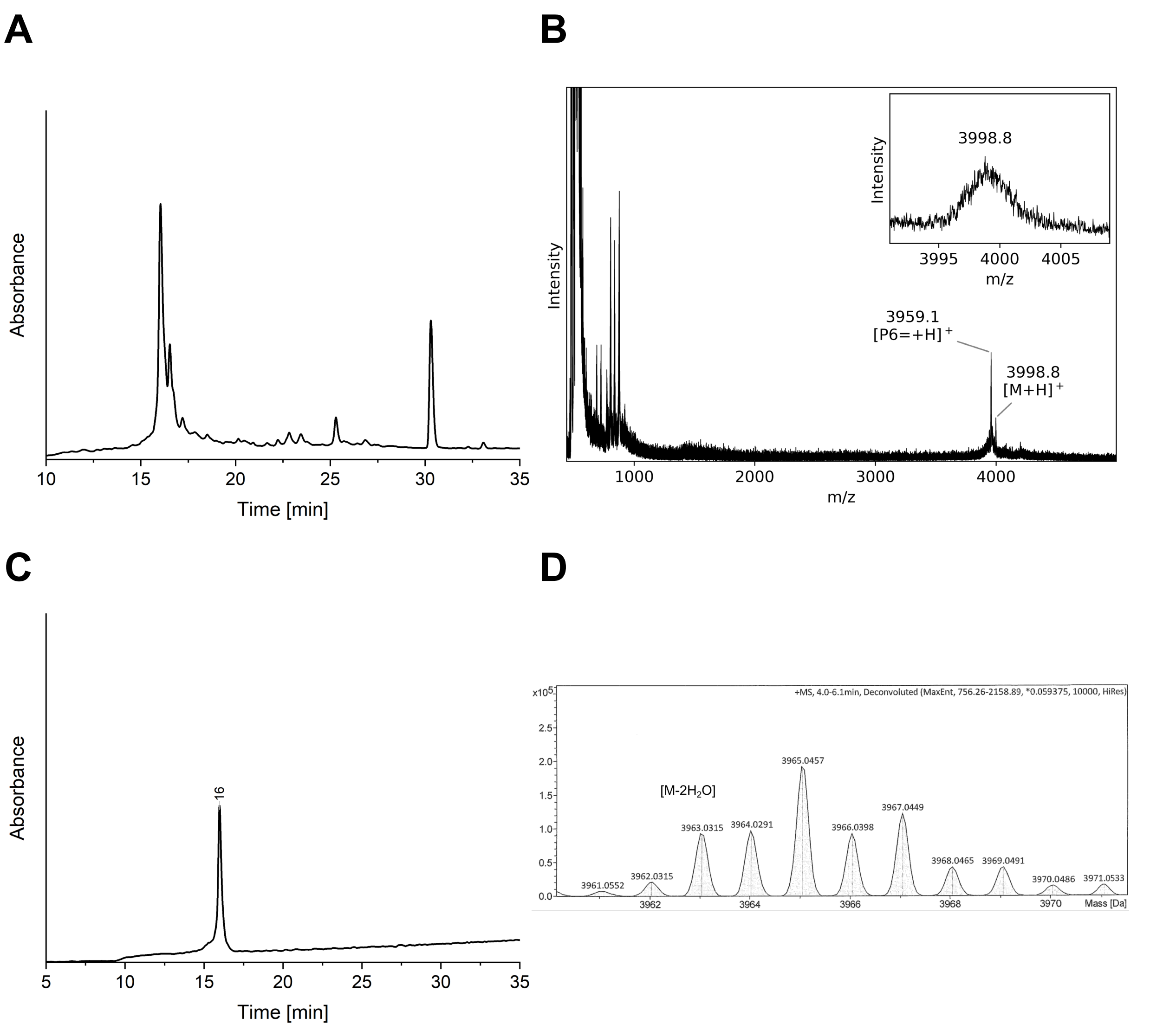

Figure S68: **P6=B(OH)$_2$** [Cp*Ru(PPh$_3$)$_2$Cl]-catalyzed hydroboration. A) Analytical HPLC (220 nm, column B) of crude peptide. B) MALDI-TOF MS spectrum of crude peptide. C) Analytical HPLC (220 nm, column B) of purified peptide. D) Deconvoluted ESI HR-MS spectrum of purified peptide. Calculated mass: [**P6=**+H]$^+$ 3960.1, [M-2H$_2$O]$^+$ 4000.0.

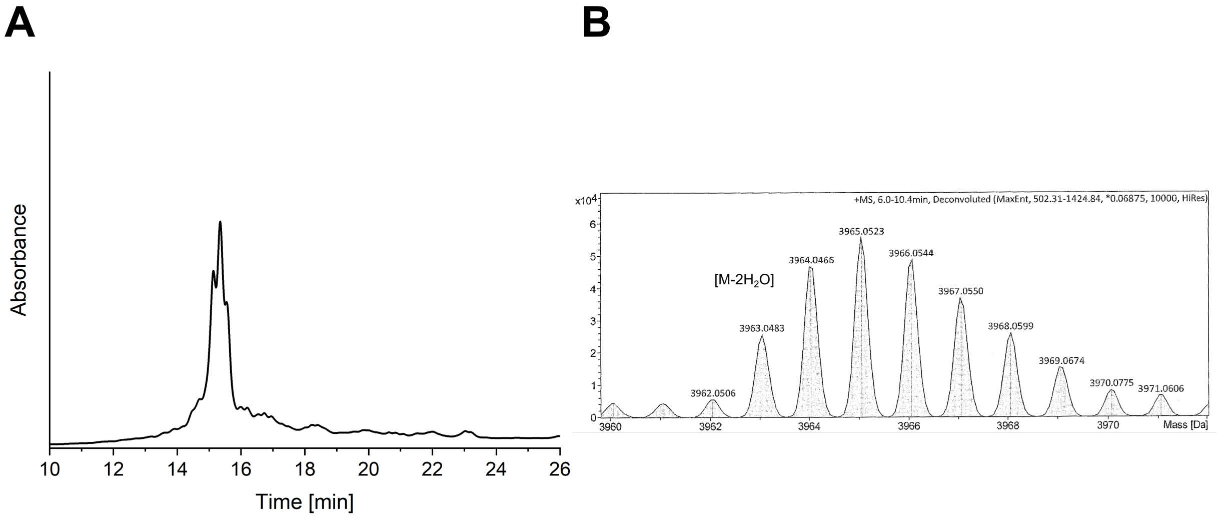

Figure S69: **P6=B(OH)₂** Iridium-catalyzed hydroboration. A) Analytical HPLC (220 nm, column A) of crude peptide. B) Deconvoluted ESI HR-MS spectrum of purified peptide. Full HR-MS spectrum in section 3. Calculated mass: [M-2H_2O] 3963.0.

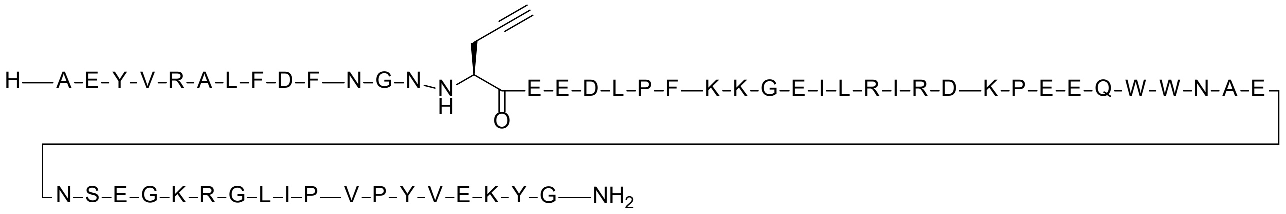

P7≡

Chemical Formula: $C_{312}H_{467}N_{83}O_{91}$

Exact Mass: 6832.45

Molecular Weight: 6836.66

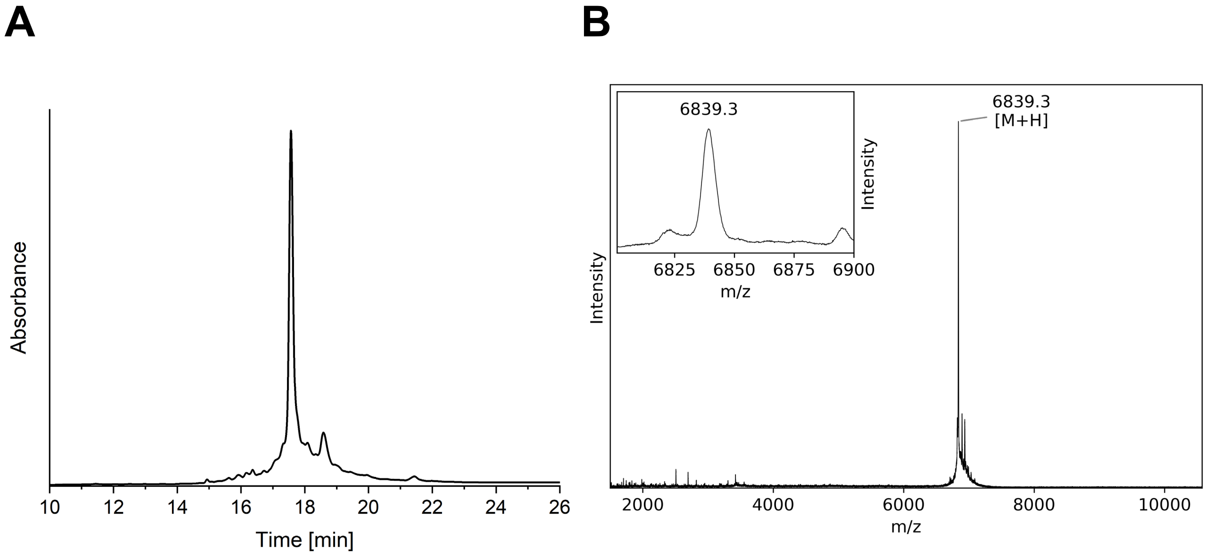

Figure S70: **P7≡** A) Analytical HPLC (220 nm, column B) of crude peptide. B) MALDI-TOF MS spectrum of crude peptide. Calculated mass: $[M+H]^+$ 6833.5.

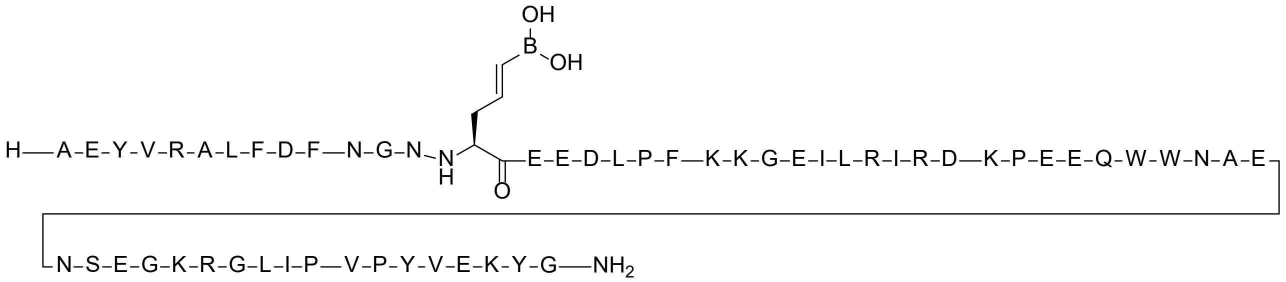

P7=B(OH)$_2$

Chemical Formula: $C_{312}H_{470}BN_{83}O_{93}$

Exact Mass: 6878.47

Molecular Weight: 6882.49

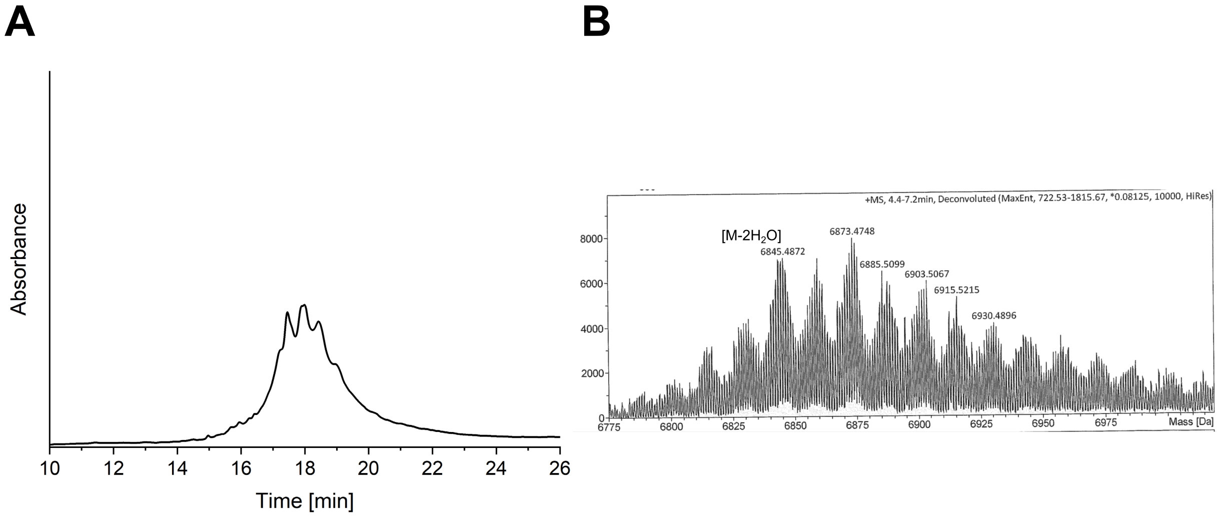

Figure S71 **P7=B(OH)$_2$** [Ru(CO)(Cl)H(PPh$_3$)$_3$]-catalyzed hydroboration. A) Analytical HPLC (220 nm, column B) of crude peptide. B) Deconvoluted ESI HR-MS spectrum of crude peptide. Full HR-MS spectrum in section 3. Calculated mass: $[M-2H_2O]^+$ 6842.5.

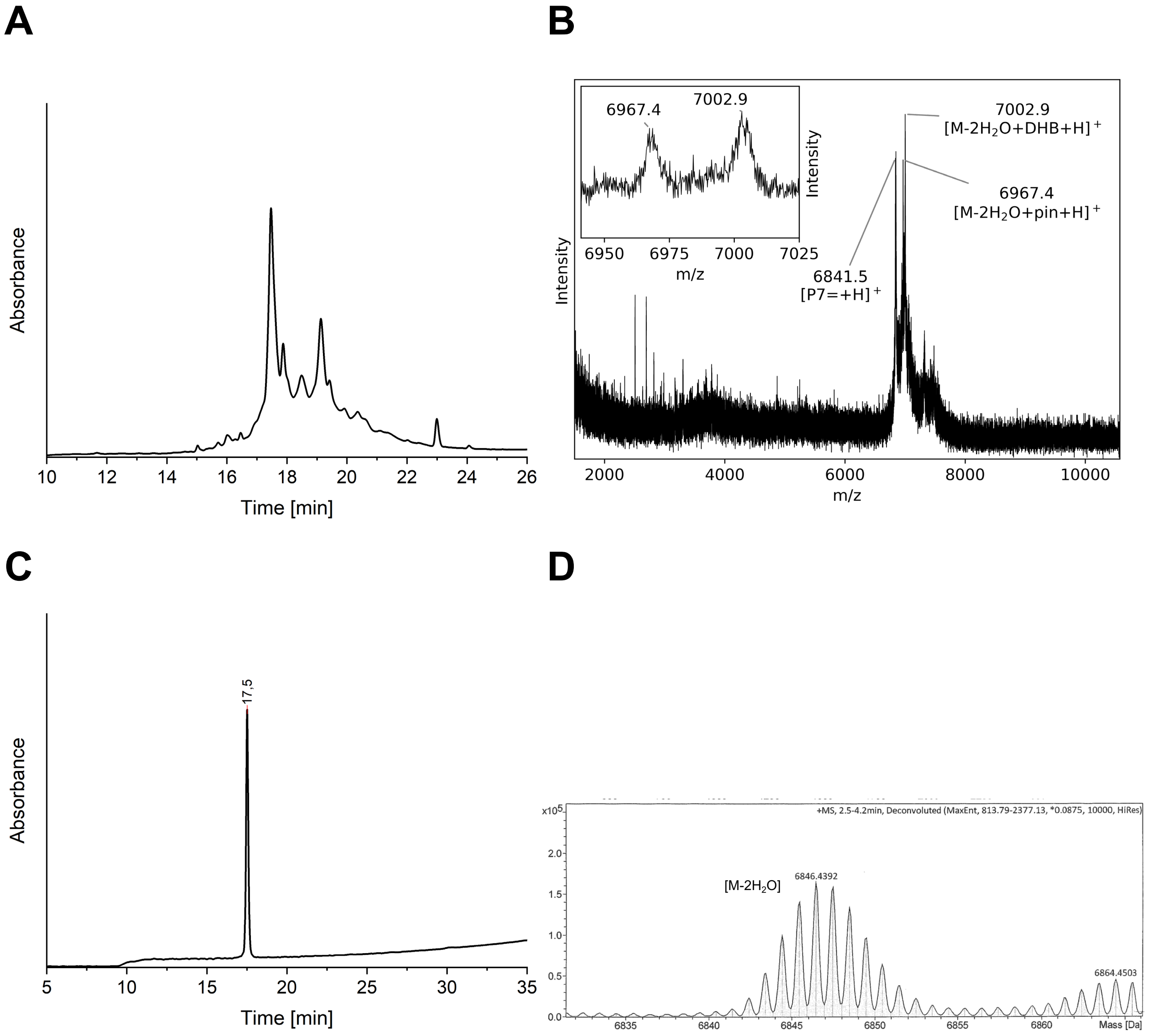

Figure S72: **P7=B(OH)₂** [Cp*Ru(PPh_3)$_2$Cl]-catalyzed hydroboration. A) Analytical HPLC (220 nm, column B) of crude peptide. B) MALDI-TOF MS spectrum of crude peptide. C) Analytical HPLC (220 nm, column B) of purified peptide. D) MALDI-TOF MS spectrum of purified peptide. Full HR-MS spectrum in section 3. Calculated mass: [**P7=**+H]$^+$ 6835.5, [M-2H_2O]$^+$ 6842.5, [M-2H_2O+DHB+H]$^+$ 6961.6, [M-2H_2O+DHB+H]$^+$ 6997.5.

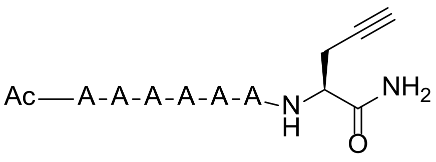

P8≡

Chemical Formula: $C_{25}H_{40}N_8O_8$

Exact Mass: 580.30

Molecular Weight: 580.64

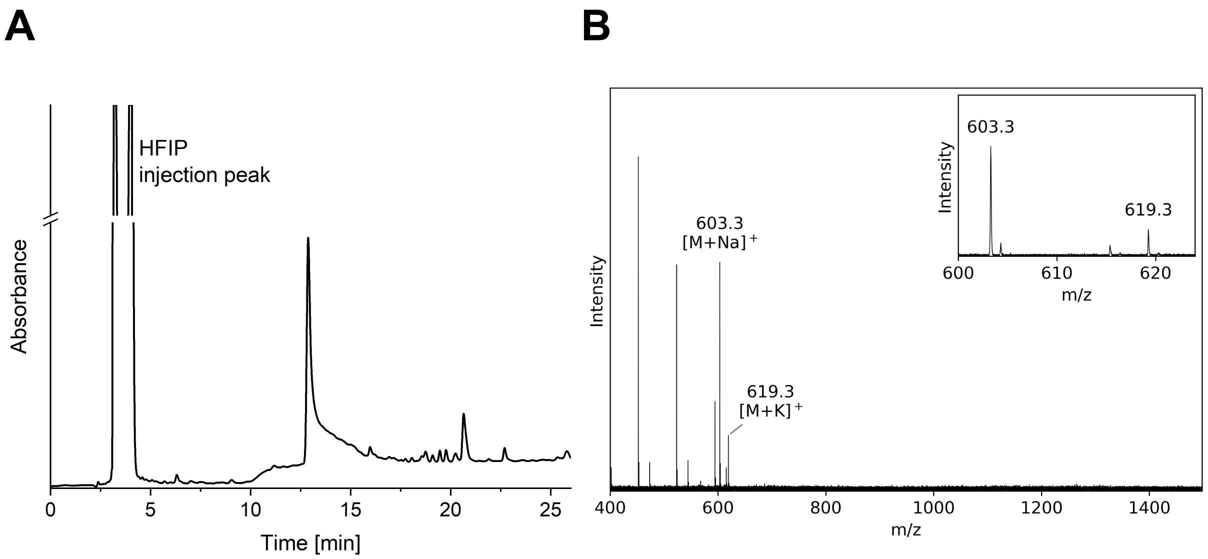

Figure S73: **P8≡** A) Analytical HPLC (220 nm, column B) of crude peptide. B) MALDI-TOF MS spectrum of crude peptide. Calculated mass: $[M+Na]^+$ 603.3, $[M+K]^+$ 619.3.

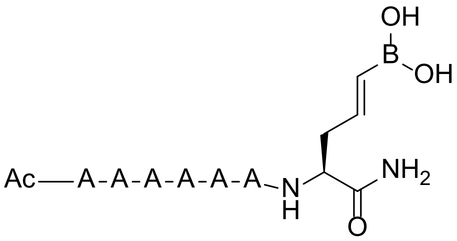

P8=B(OH)$_2$

Chemical Formula: $C_{25}H_{43}BN_8O_{10}$

Exact Mass: 626.32

Molecular Weight: 626.47

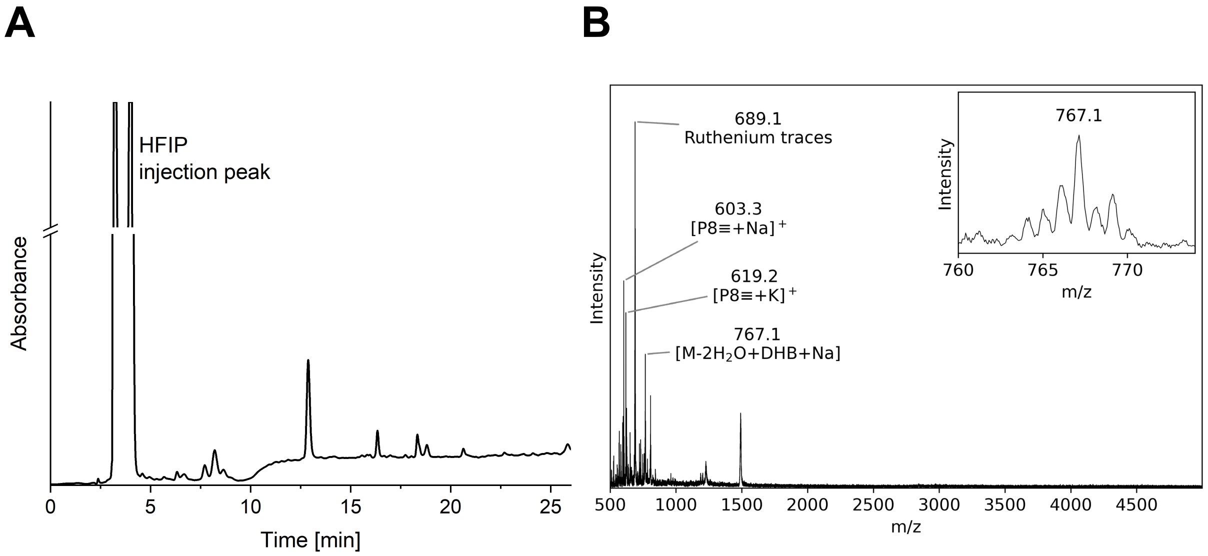

Figure S74 **P8=B(OH)$_2$** [Ru(CO)(Cl)H(PPh$_3$)$_3$]-catalyzed hydroboration. A) Analytical HPLC (220 nm, column B) of crude peptide. B) MALDI-TOF MS spectrum of crude peptide. Calculated mass: [**P8≡**+Na]$^+$ 603.3, [**P8≡**+K]$^+$ 619.3, [M-2H$_2$O+DHB+Na]$^+$ 767.3.

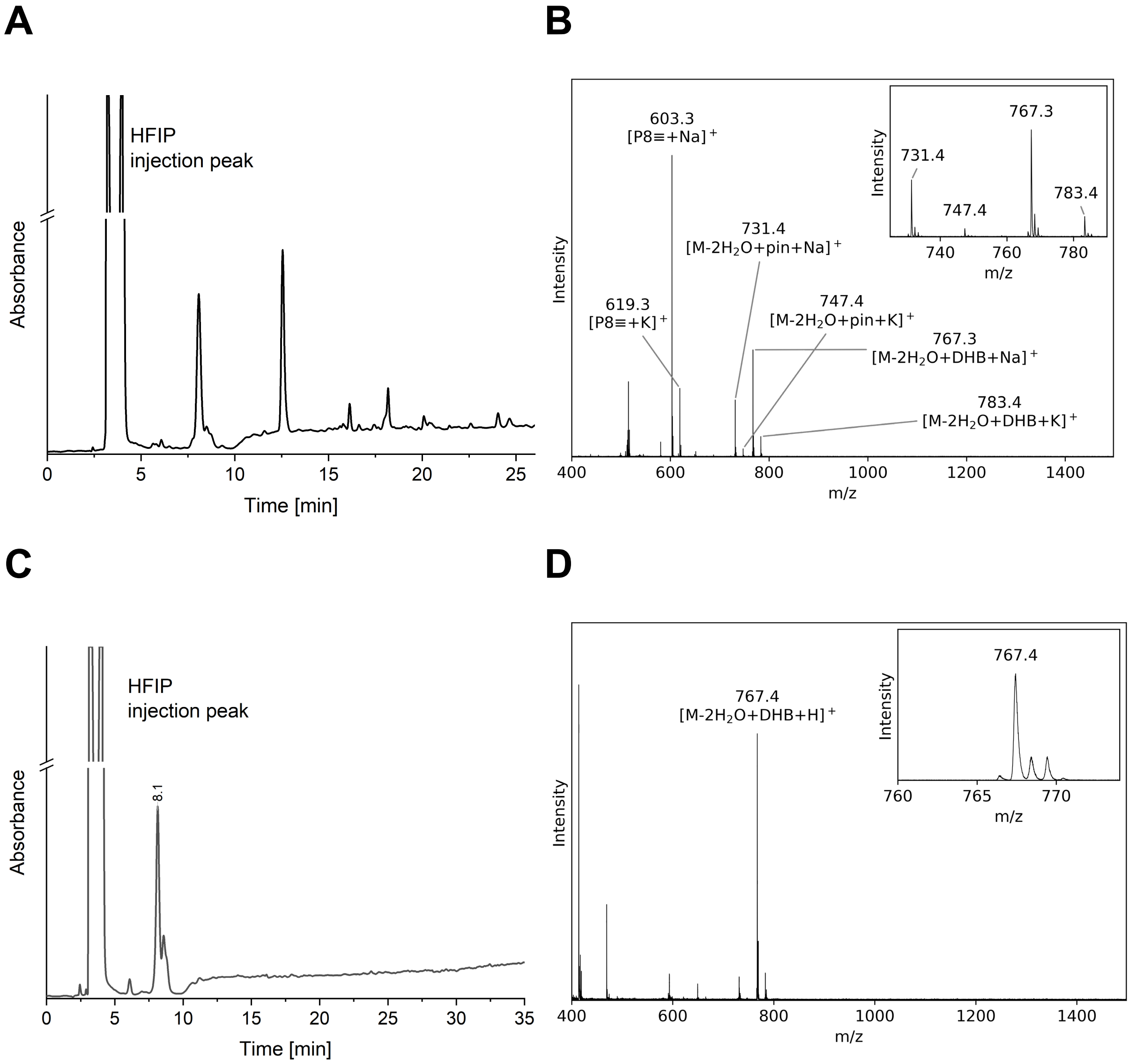

Figure S75: **P8=B(OH)₂** [Cp*Ru(PPh₃)₂Cl]-catalyzed hydroboration. A) Analytical HPLC (220 nm, column B) of crude peptide. B) MALDI-TOF MS spectrum of crude peptide. C) Analytical HPLC (220 nm, column B) of purified peptide. D) MALDI-TOF MS spectrum of purified peptide. Calculated mass: [**P8≡**+Na]$^+$ 603.3, [**P8≡**+K]$^+$ 619.3, [M-2H_2O+pin+Na]$^+$ 731.4, [M-2H_2O+pin+K]$^+$ 747.4, [M-2H_2O+DHB+Na]$^+$ 767.3, [M-2H_2O+DHB+K]$^+$ 783.3.

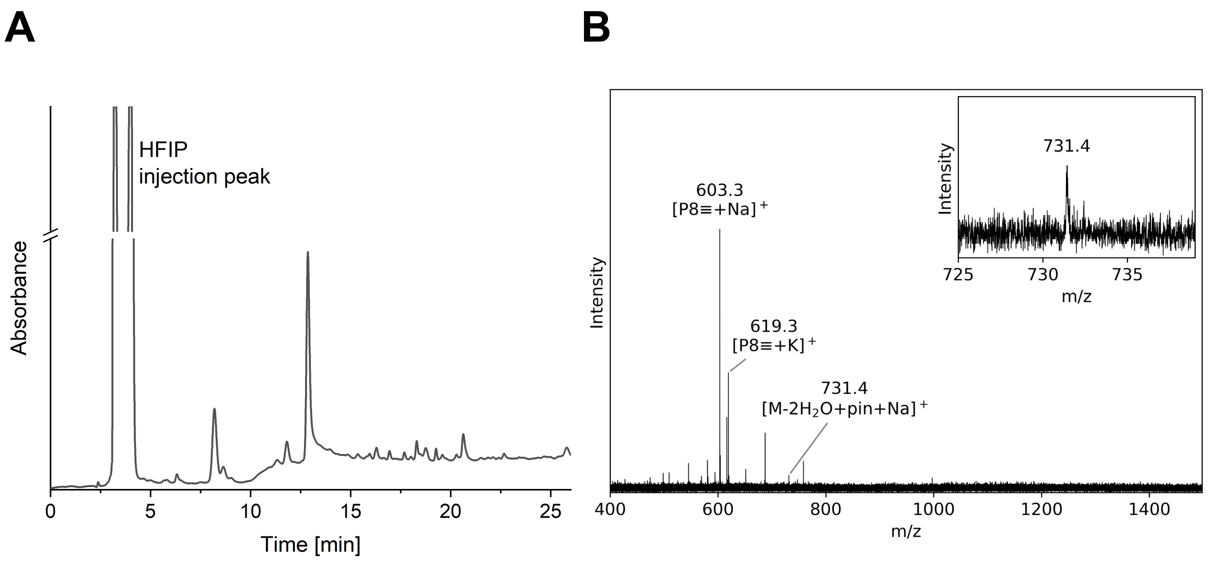

Figure S76: **P8=B(OH)$_2$** Iridium-catalyzed hydroboration. A) Analytical HPLC (220 nm, column B) of crude peptide. B) MALDI-TOF MS spectrum of crude peptide. Calculated mass: [**P8≡**+Na]$^+$ 603.3, [**P8≡**+K]$^+$ 619.3, [M-2H_2O+pin+Na]$^+$ 731.4.

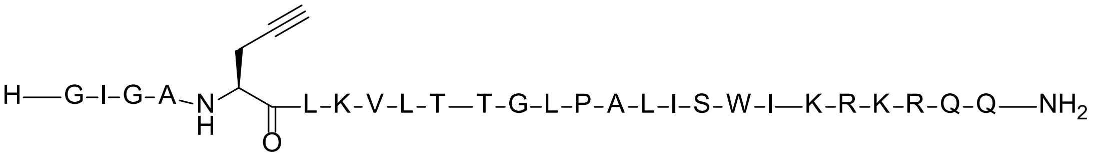

P9≡

Chemical Formula: $C_{131}H_{225}N_{39}O_{31}$

Exact Mass: 2840.72

Molecular Weight: 2842.48

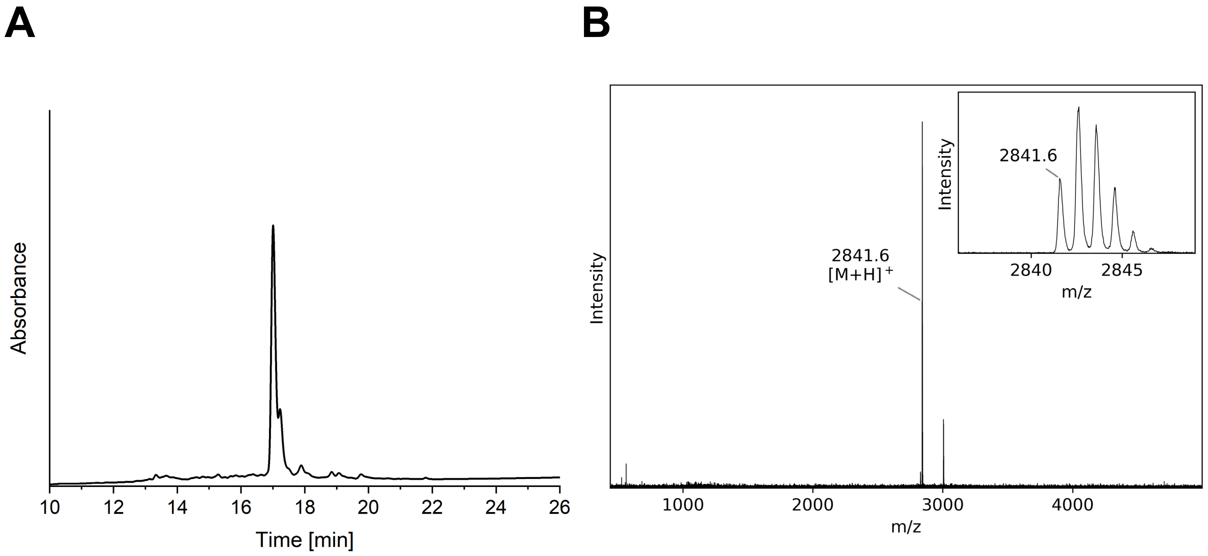

Figure S77: **P9≡** A) Analytical HPLC (220 nm, column B) of crude peptide. B) MALDI-TOF MS spectrum of crude peptide. Calculated mass: $[M+H]^+$ 2841.7.

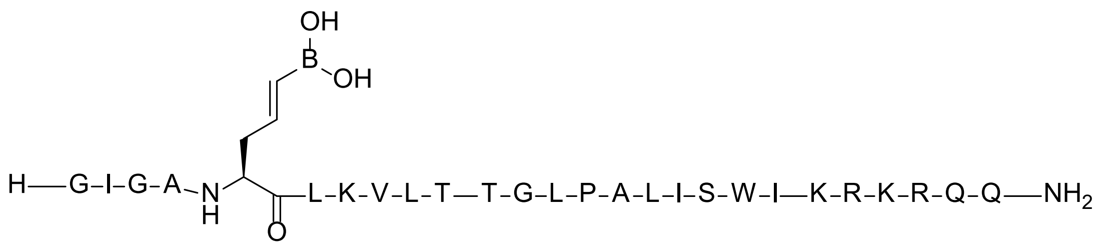

P9=B(OH)$_2$

Chemical Formula: $C_{131}H_{228}BN_{39}O_{33}$

Exact Mass: 2886.75

Molecular Weight: 2888.32

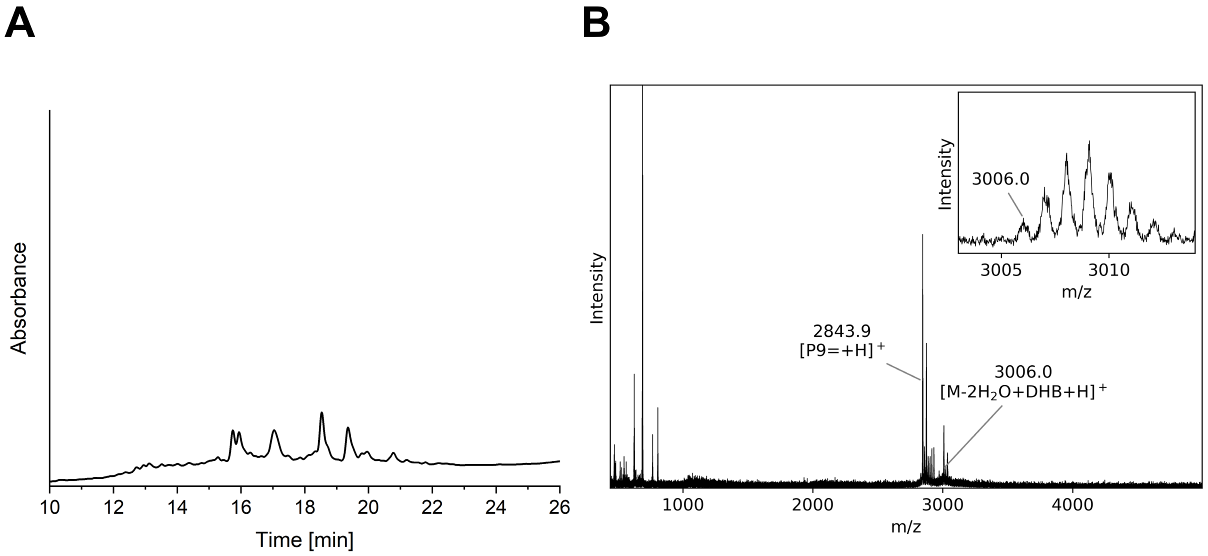

Figure S78 **P9=B(OH)$_2$** [Ru(CO)(Cl)H(PPh_3)$_3$]-catalyzed hydroboration. A) Analytical HPLC (220 nm, column B) of crude peptide. B) MALDI-TOF MS spectrum of crude peptide. Calculated mass: [**P9=**+H]$^+$ 2843.7, [M-2H_2O+DHB+H]$^+$ 3005.8.

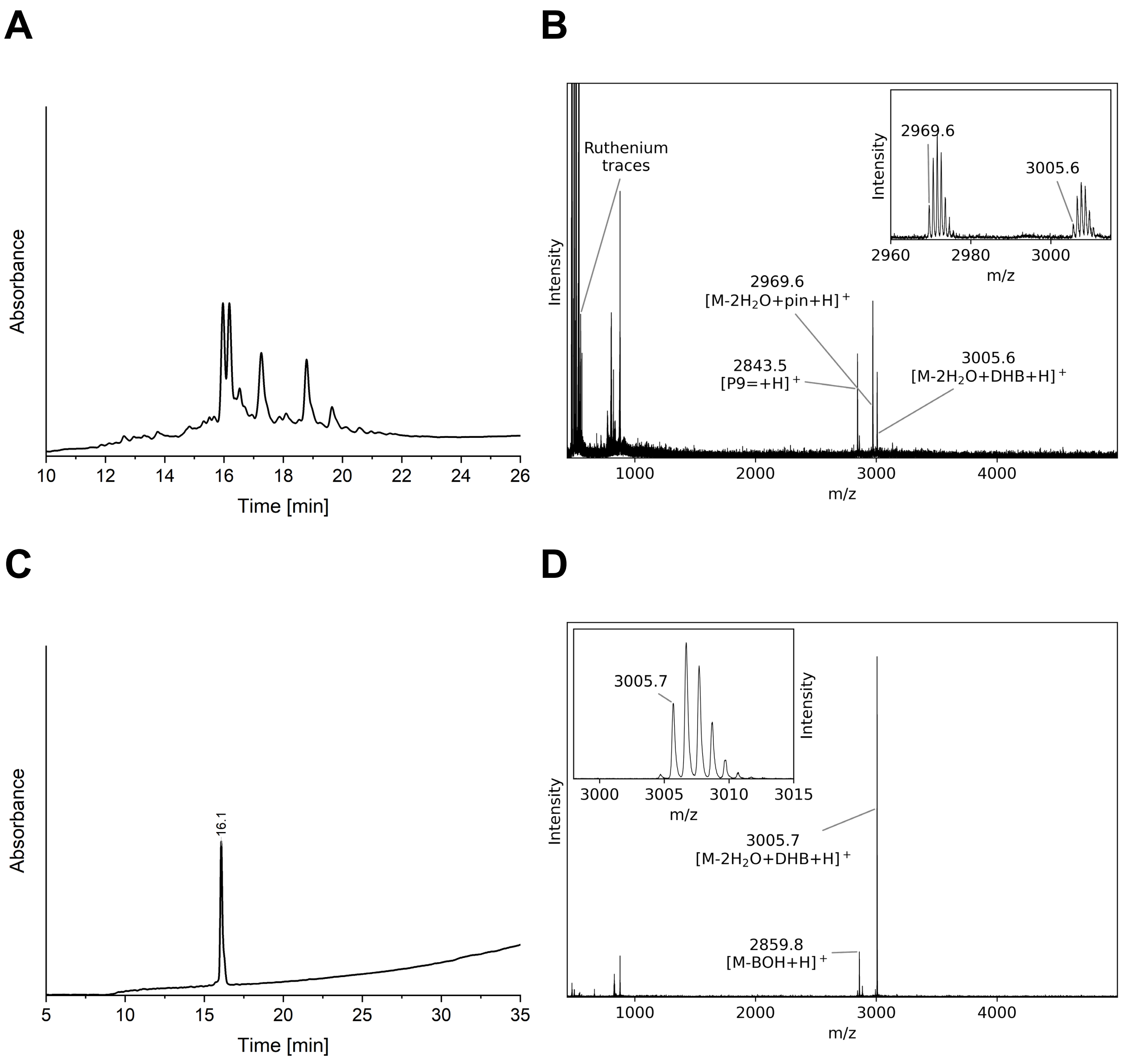

Figure S79: **P9=B(OH)₂** [Cp*Ru(PPh3)2Cl]-catalyzed hydroboration. A) Analytical HPLC (220 nm, column B) of crude peptide. B) MALDI-TOF MS spectrum of crude peptide. C) Analytical HPLC (220 nm, column B) of purified peptide. D) MALDI-TOF MS spectrum of purified peptide. Calculated mass: [**P9=**+H]+ 2843.7, [M-BOH+H]+ 2859.7, [M-2H2O+DHB+H]+ 3005.8.

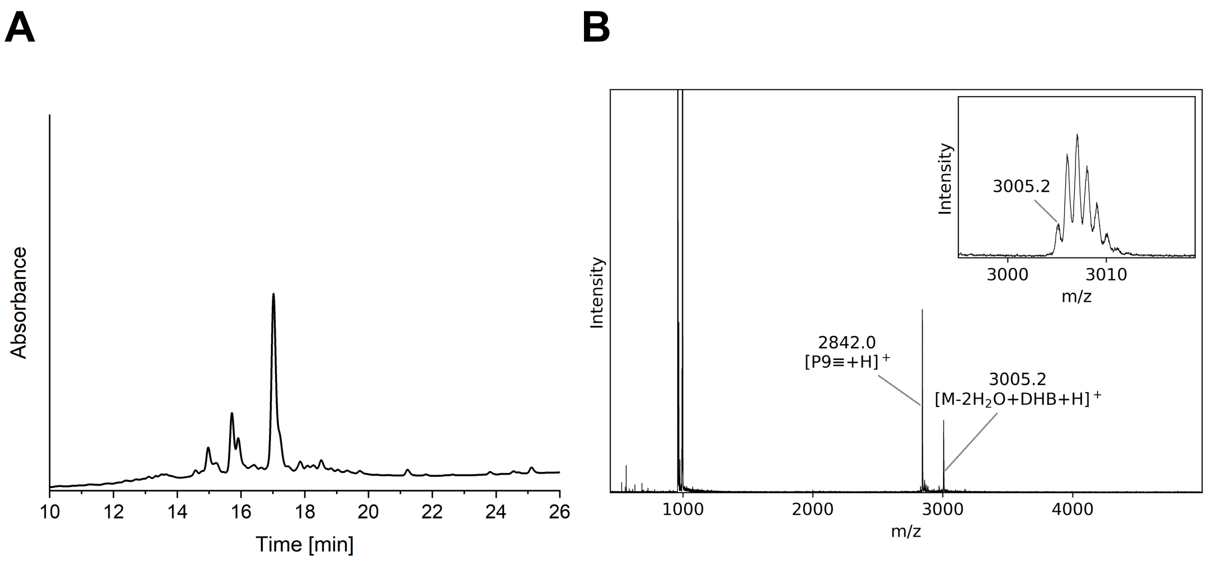

Figure S80: **P9=B(OH)$_2$** Iridium-catalyzed hydroboration. A) Analytical HPLC (220 nm, column B) of crude peptide. B) MALDI-TOF MS spectrum of crude peptide. Calculated mass [**P9≡**+H]$^+$ 2841.7, [M-BOH+H]$^+$ 2859.7, [M-2H_2O+DHB+H]$^+$ 3005.8.

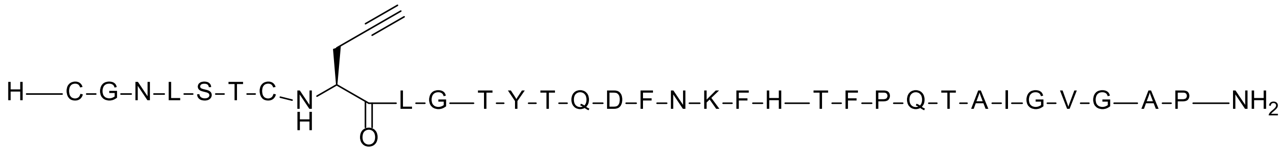

P10≡

Chemical Formula: $C_{151}H_{224}N_{40}O_{45}S_2$

Exact Mass: 3381.59

Molecular Weight: 3383.81

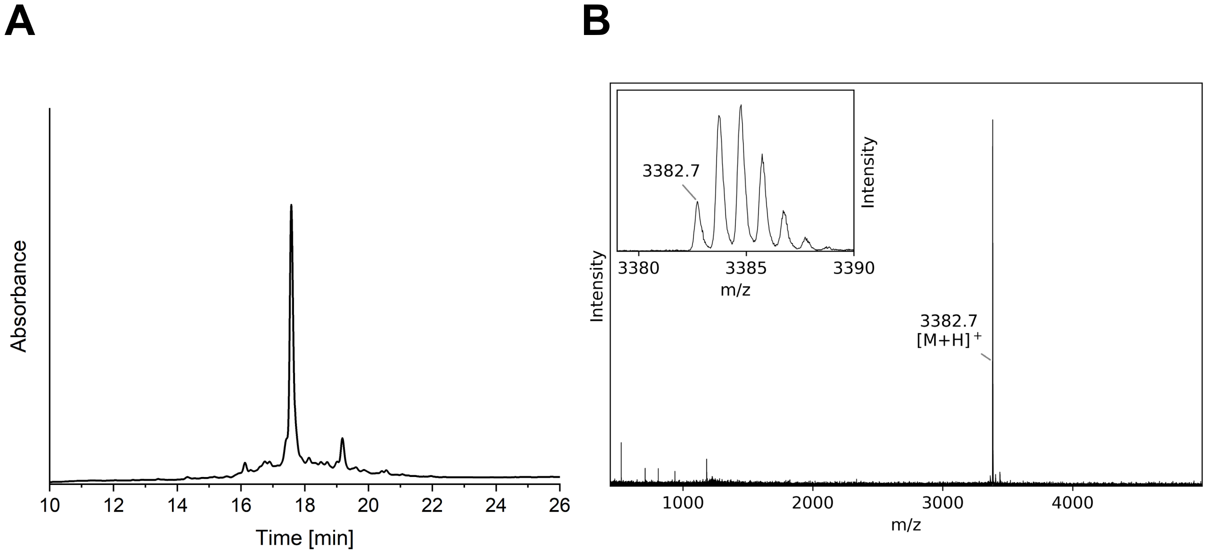

Figure S81: **P10≡** A) Analytical HPLC (220 nm, column B) of crude peptide. B) MALDI-TOF MS spectrum of crude peptide. Calculated mass: $[M+H]^+$ 3382.6.

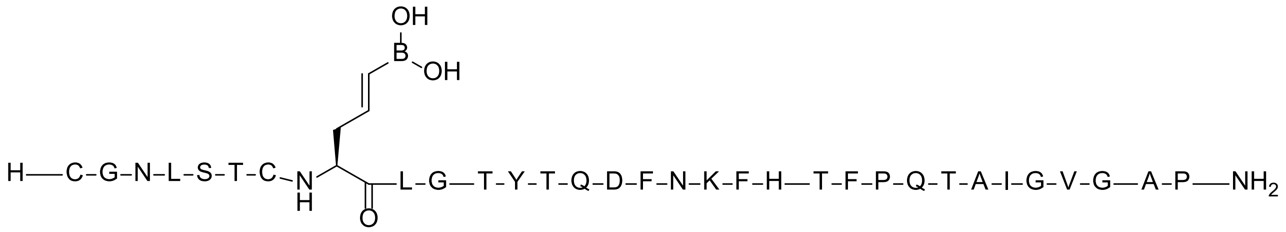

P10=B(OH)$_2$

Chemical Formula: $C_{151}H_{227}BN_{40}O_{47}S_2$

Exact Mass: 3427.61

Molecular Weight: 3429.64

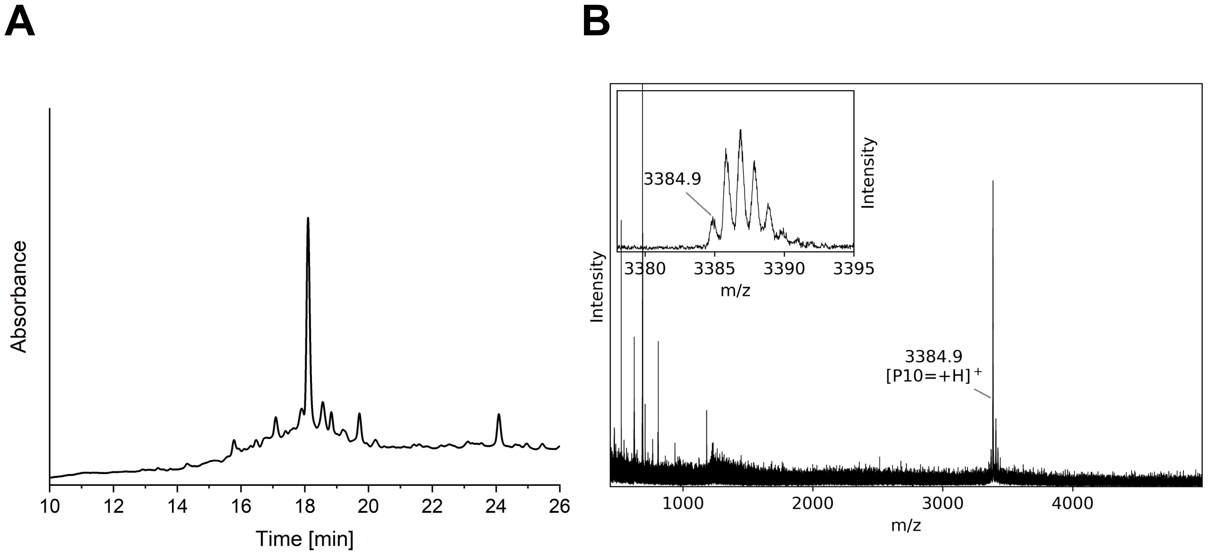

Figure S82 **P10=B(OH)₂** [Ru(CO)(Cl)H(PPh$_3$)$_3$]-catalyzed hydroboration. A) Analytical HPLC (220 nm, column B) of crude peptide. B) MALDI-TOF MS spectrum of crude peptide. Calculated mass: [**P10=**+H]$^+$ 3384.6.

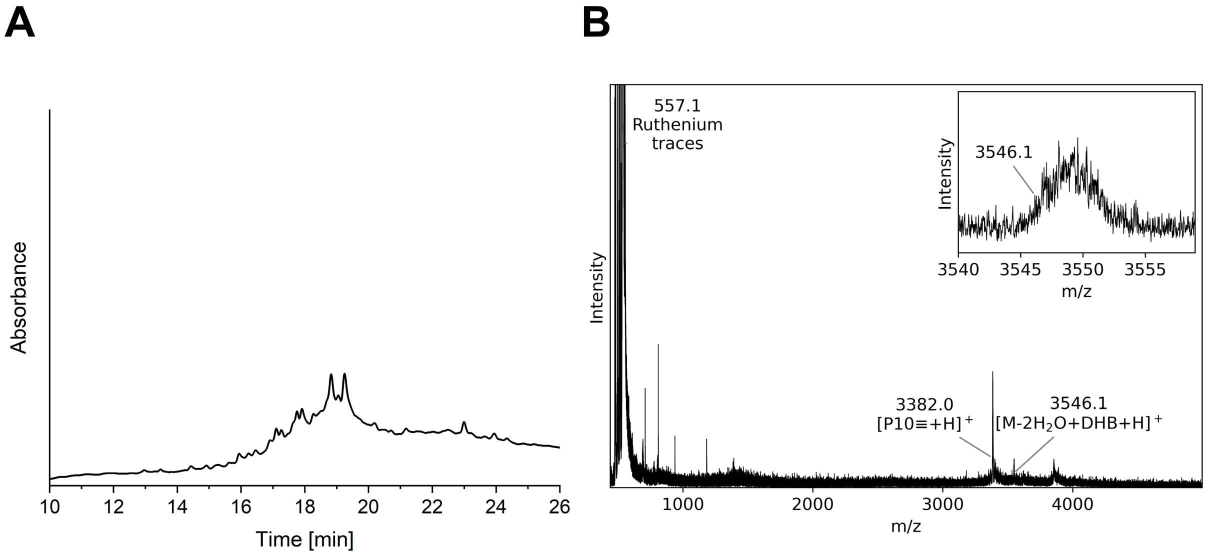

Figure S83: **P10=B(OH)₂** [Cp*Ru(PPh₃)₂Cl]-catalyzed hydroboration. A) Analytical HPLC (220 nm, column B) of crude peptide. B) MALDI-TOF MS spectrum of crude peptide. Calculated mass: [**P10≡**+H]$^+$ 3382.6, [M-2H_2O+DHB+H]$^+$ 3546.6.

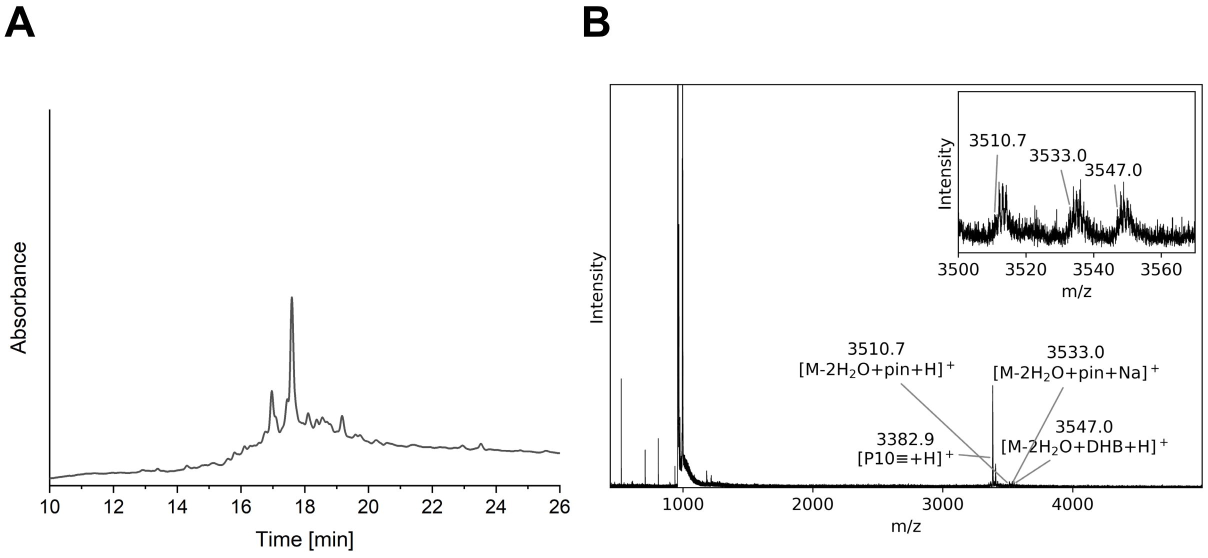

Figure S84: **P10=B(OH)₂** Iridium-catalyzed hydroboration. A) Analytical HPLC (220 nm, column B) of crude peptide. B) MALDI-TOF MS spectrum of crude peptide. Calculated mass: [**P10≡**+H]$^+$ 3382.6, [M-2H_2O+pin+H]$^+$ 3510.7 [M-2H_2O+pin+Na]$^+$ 3532.7 [M-2H_2O+DHB+H]$^+$ 3546.6.

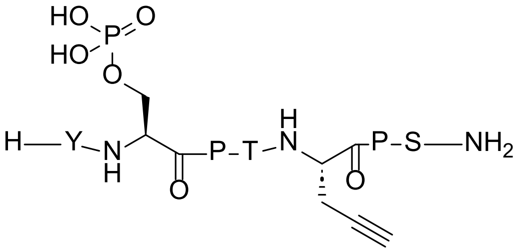

P11$^{\equiv}$

Chemical Formula: $C_{34}H_{49}N_{8}O_{14}P$

Exact Mass: 824.31

Molecular Weight: 824.78

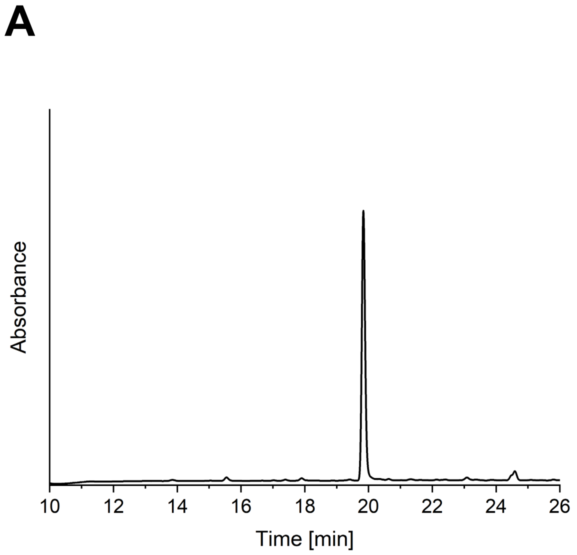

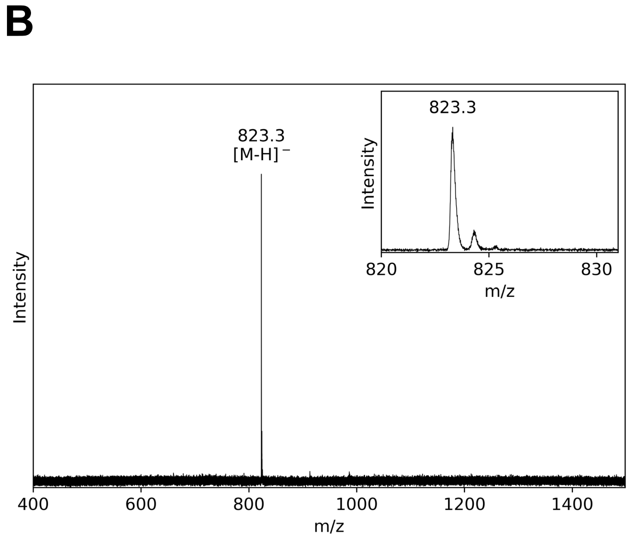

Figure S85: **P11≡** A) Analytical HPLC (220 nm, column B) of crude peptide. B) MALDI-TOF MS spectrum of crude peptide. Calculated mass: $[M-H]^-$ 823.3.

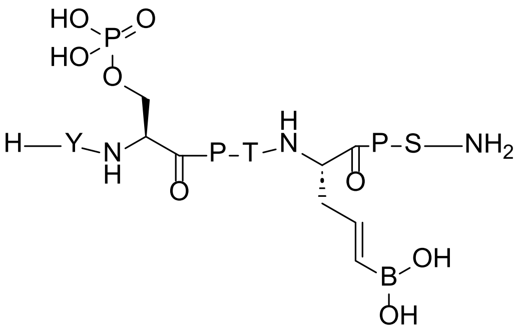

P11=B(OH)$_2$

Chemical Formula: $C_{34}H_{52}BN_8O_{16}P$

Exact Mass: 870.33

Molecular Weight: 870.61

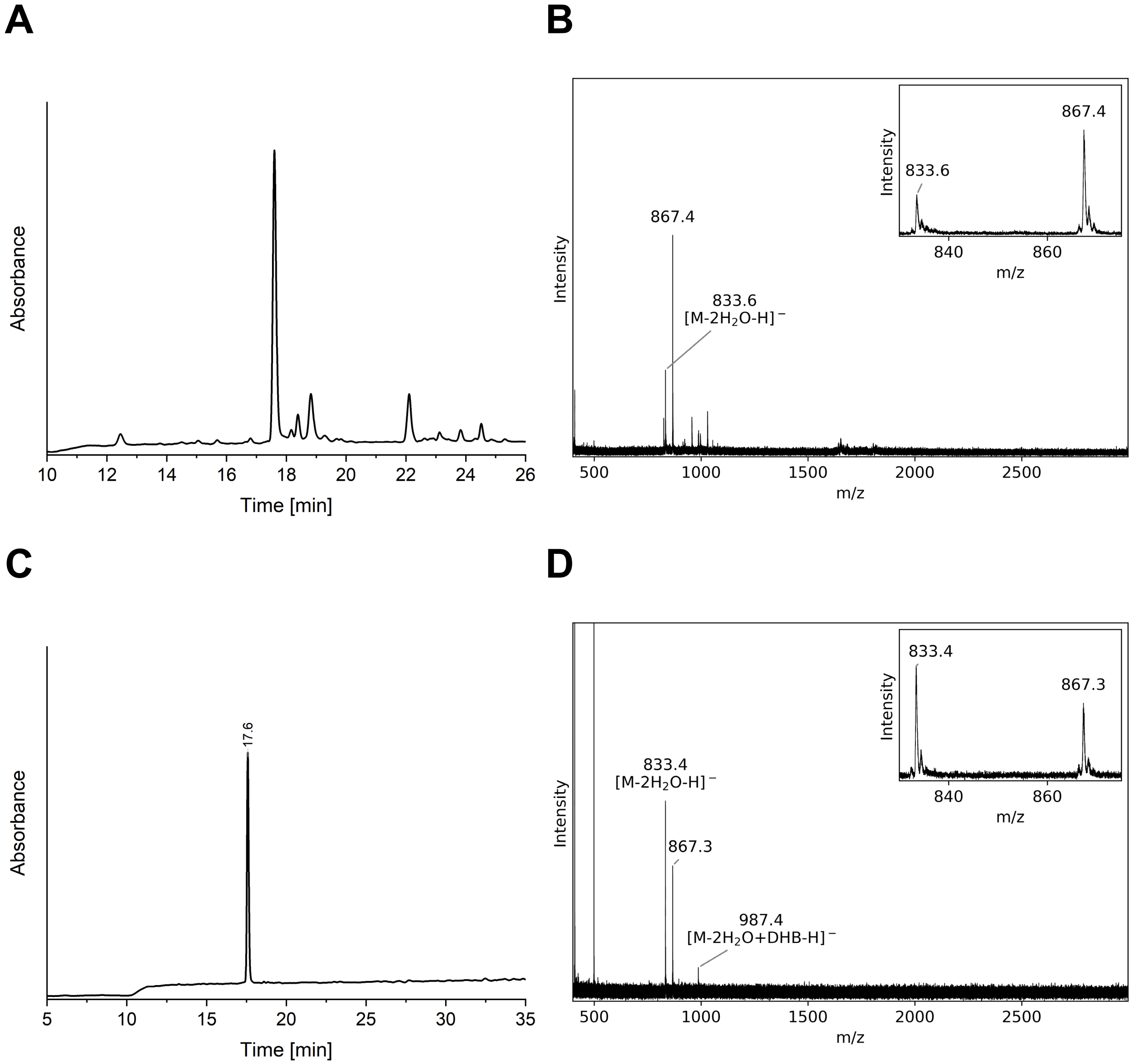

Figure S86 **P11=B(OH)$_2$** [Ru(CO)(Cl)H(PPh$_3$)$_3$]-catalyzed hydroboration. A) Analytical HPLC (220 nm, column B) of crude peptide. B) MALDI-TOF MS spectrum of crude peptide. C) Analytical HPLC (220 nm, column B) of purified peptide. D) MALDI-TOF MS spectrum of purified peptide. Calculated mass: $[M-2H_2O-H]^-$ 833.3, $[M-2H_2O+DHB-H]^-$ 987.3.

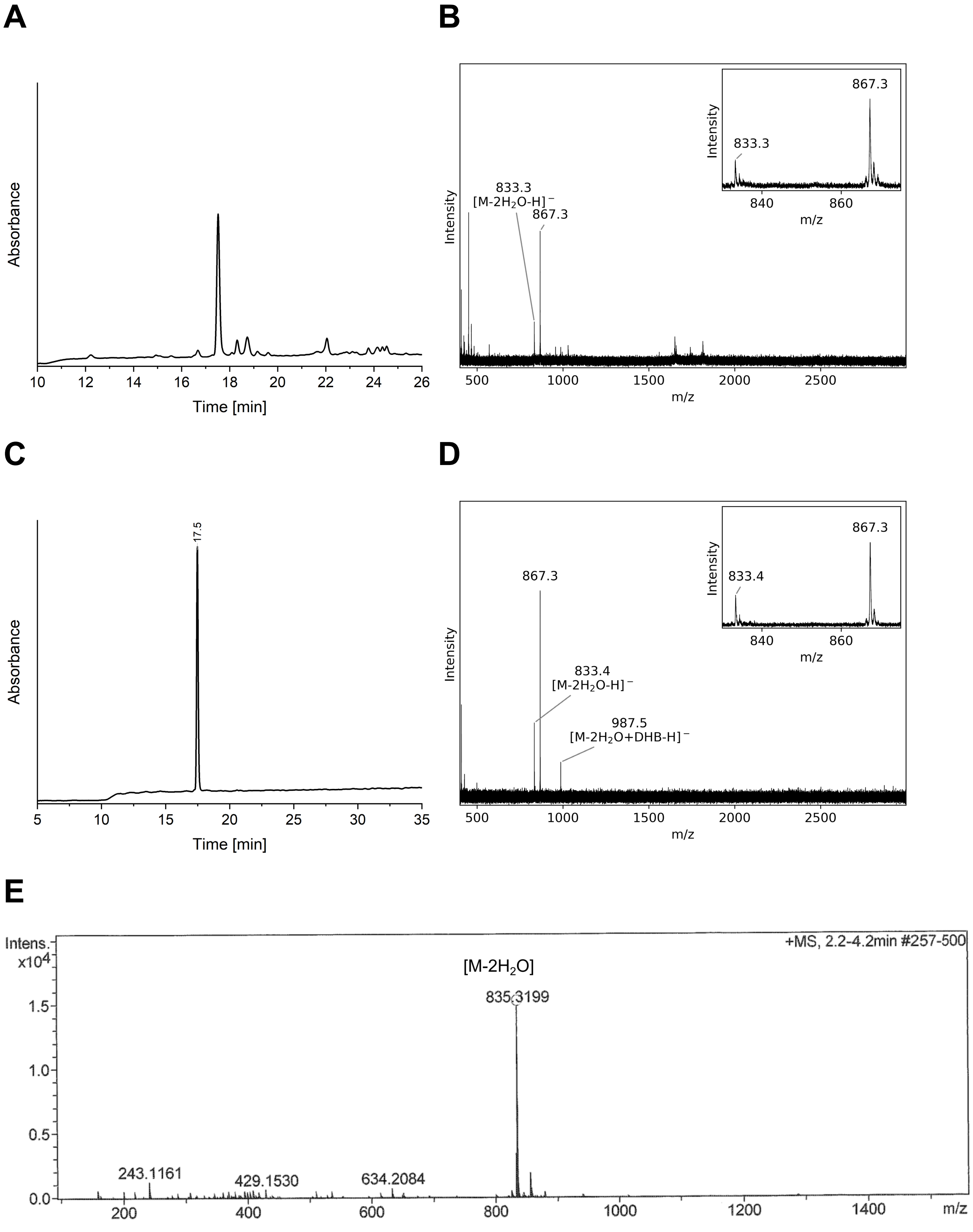

Figure S87: **P11=B(OH)$_2$** [Cp*Ru(PPh$_3$)$_2$Cl]-catalyzed hydroboration. A) Analytical HPLC (220 nm, column B) of crude peptide. B) MALDI-TOF MS spectrum of crude peptide. C) Analytical HPLC (220 nm, column B) of purified peptide. D) MALDI-TOF MS spectrum of purified peptide. E) ESI HR-MS spectrum of purified peptide. Full HR-MS spectrum in section 3. Calculated mass: $[M-2H_2O-H]^-$ 833.3, $[M-2H_2O+H]^+$ 835.3, $[M-2H_2O+DHB-H]^-$ 987.3.

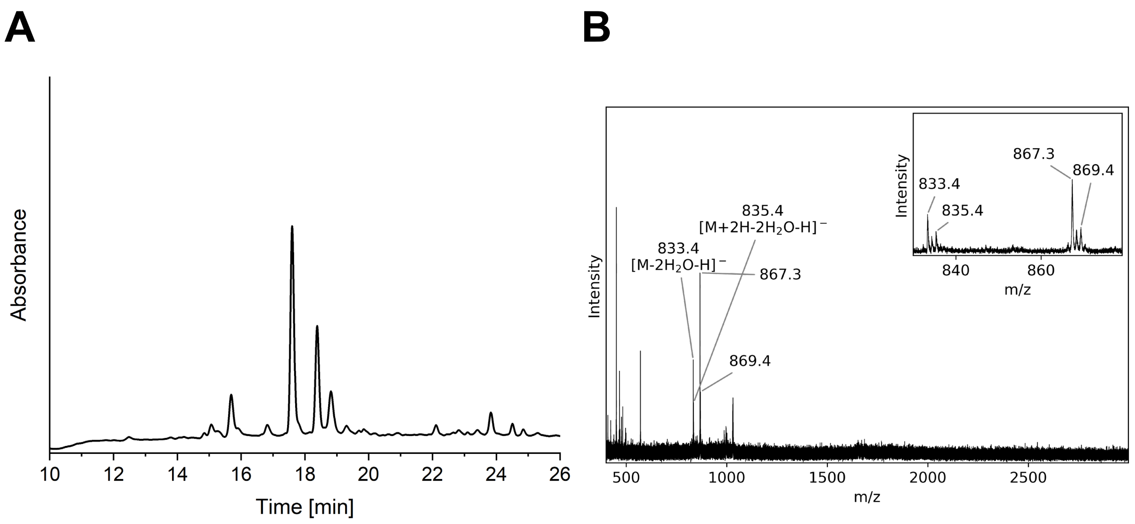

Figure S88: **P11=B(OH)₂** Iridium-catalyzed hydroboration. A) Analytical HPLC (220 nm, column B) of crude peptide. B) MALDI-TOF MS spectrum of crude peptide. Calculated mass: $[M-2H_2O-H]^-$ 833.3.

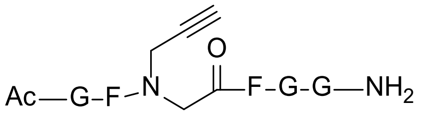

P12≡

Chemical Formula: $C_{31}H_{37}N_7O_7$
Exact Mass: 619.28
Molecular Weight: 619.68

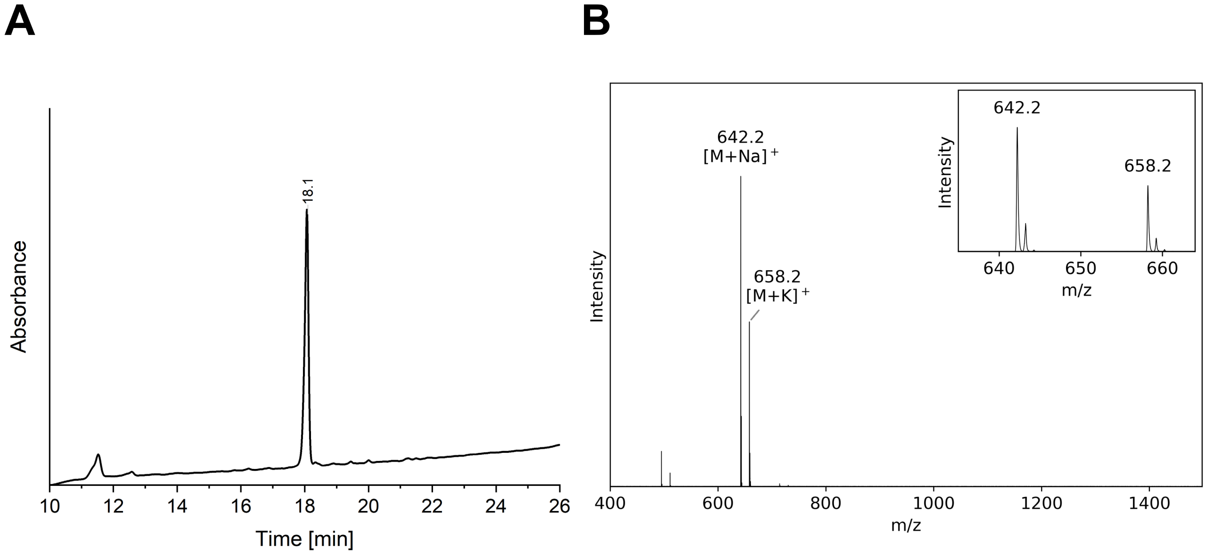

Figure S89: **P12≡** A) Analytical HPLC (220 nm, column A) of crude peptide. B) MALDI-TOF MS spectrum of crude peptide. Calculated mass: $[M+Na]^+$ 642.3, $[M+K]^+$ 658.2.

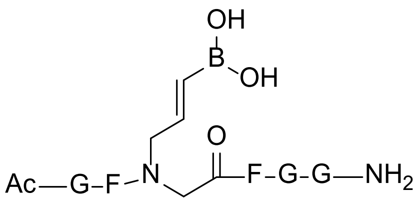

P12=B(OH)$_2$

Chemical Formula: $C_{31}H_{40}BN_7O_9$

Exact Mass: 665.30

Molecular Weight: 665.51

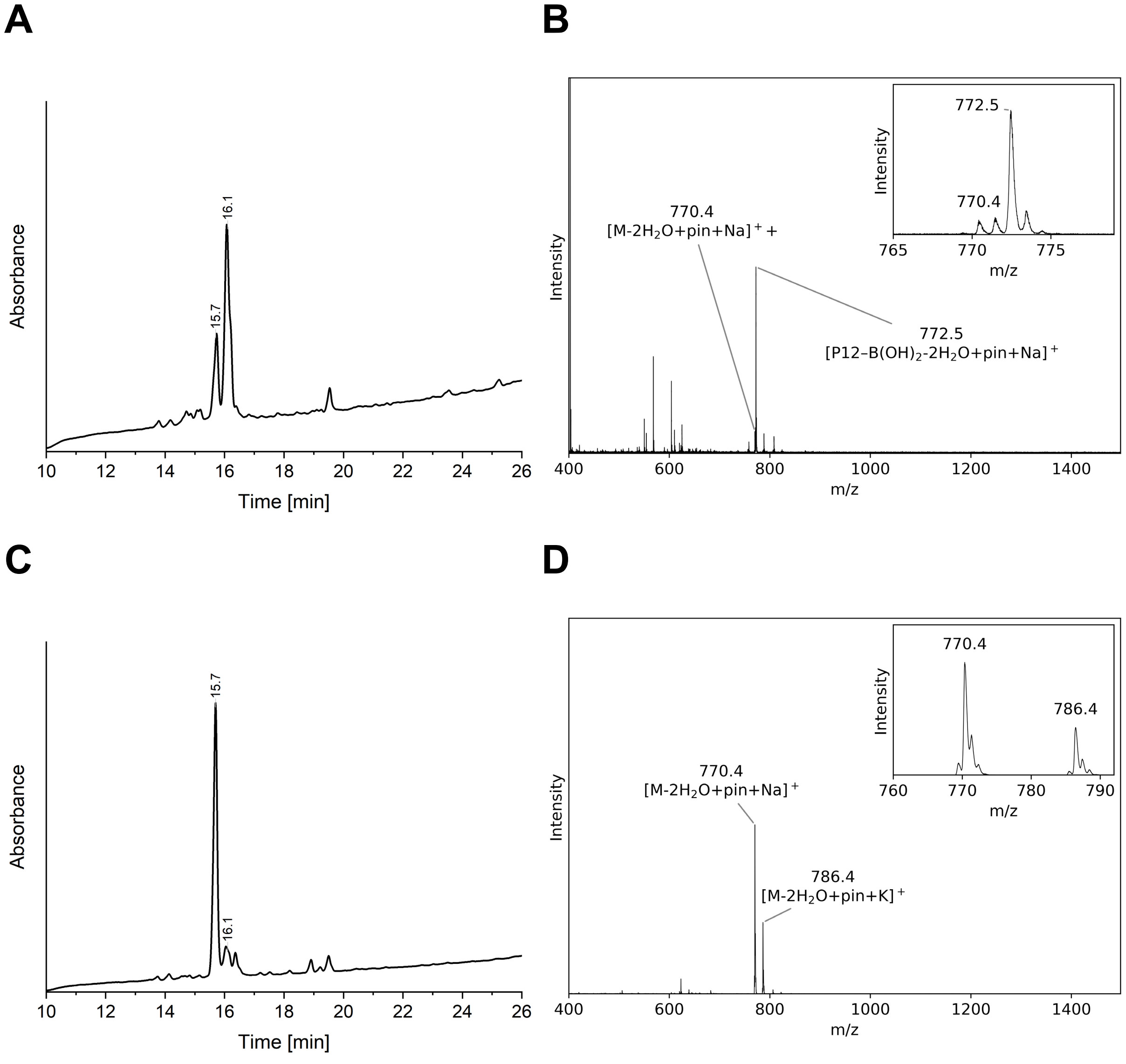

E

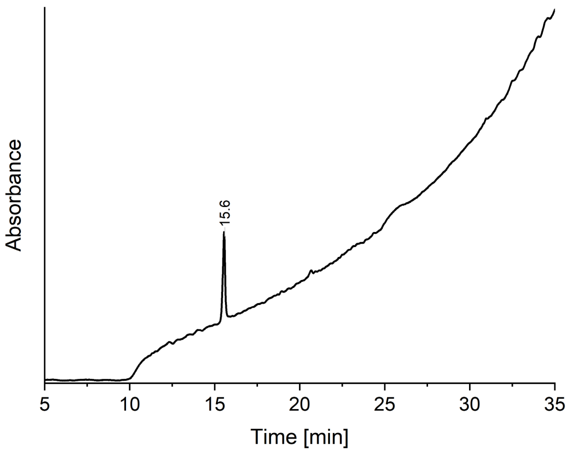

F

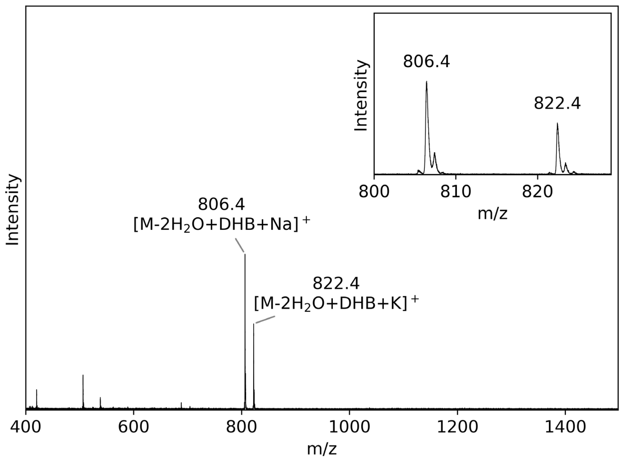

Figure S90: **P12=B(OH)$_2$** [Ru(CO)(Cl)H(PPh$_3$)$_3$]-catalyzed hydroboration. A) Analytical HPLC (220 nm, column A) of crude peptide after 64 h reaction time. B) MALDI-TOF MS spectrum of crude peptide after 64 h reaction time. C) Analytical HPLC (220 nm, column A) of crude peptide after 16 h reaction time. D) MALDI-TOF MS spectrum of crude peptide after 16 h reaction time. E) Analytical HPLC (220 nm, column A) of purified peptide. F) MALDI-TOF MS spectrum of purified peptide. Calculated mass: [M-2H$_2$O+pin+Na]$^+$ 770.4, [**P12–**2H$_2$O+pin+Na]$^+$ 772.4, [M-2H$_2$O+pin+K]$^+$ 786.3, [M-2H$_2$O+DHB+Na]$^+$ 806.3, [M-2H$_2$O+DHB+K]$^+$ 822.3.

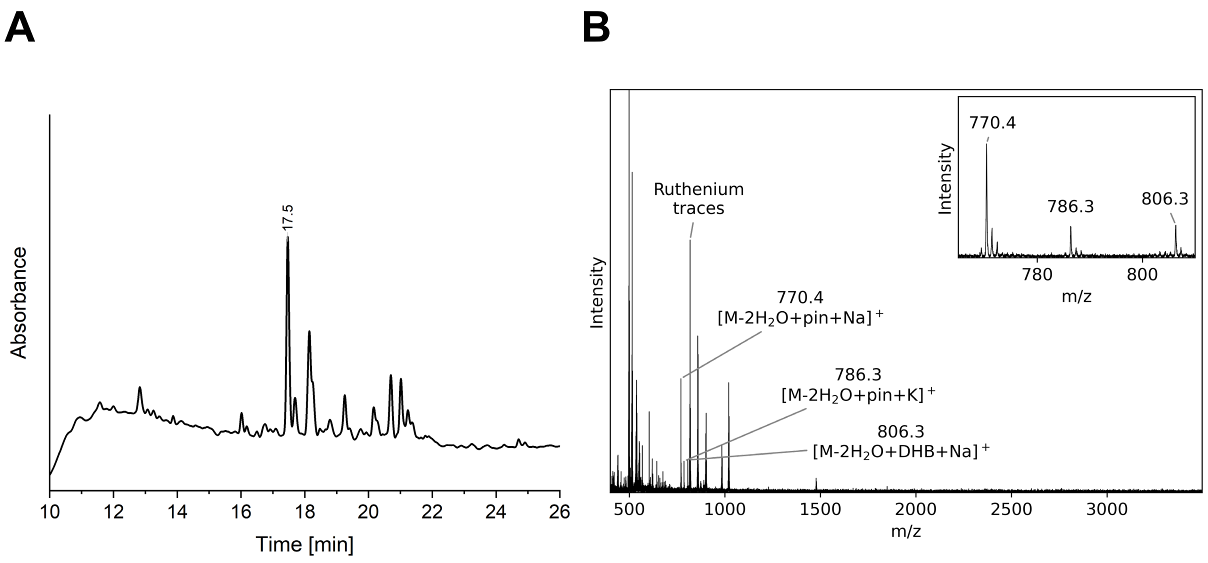

Figure S91: **P12=B(OH)₂** [Cp*Ru(PPh₃)₂Cl]-catalyzed hydroboration. A) Analytical HPLC (220 nm, column B) of crude peptide. B) MALDI-TOF MS spectrum of crude peptide. Calculated mass: $[M\text{-}2H_2O\text{+}pin\text{+}Na]^+$ 770.4, $[M\text{-}2H_2O\text{+}pin\text{+}K]^+$ 786.3, $[M\text{-}2H_2O\text{+}DHB\text{+}Na]^+$ 806.3.

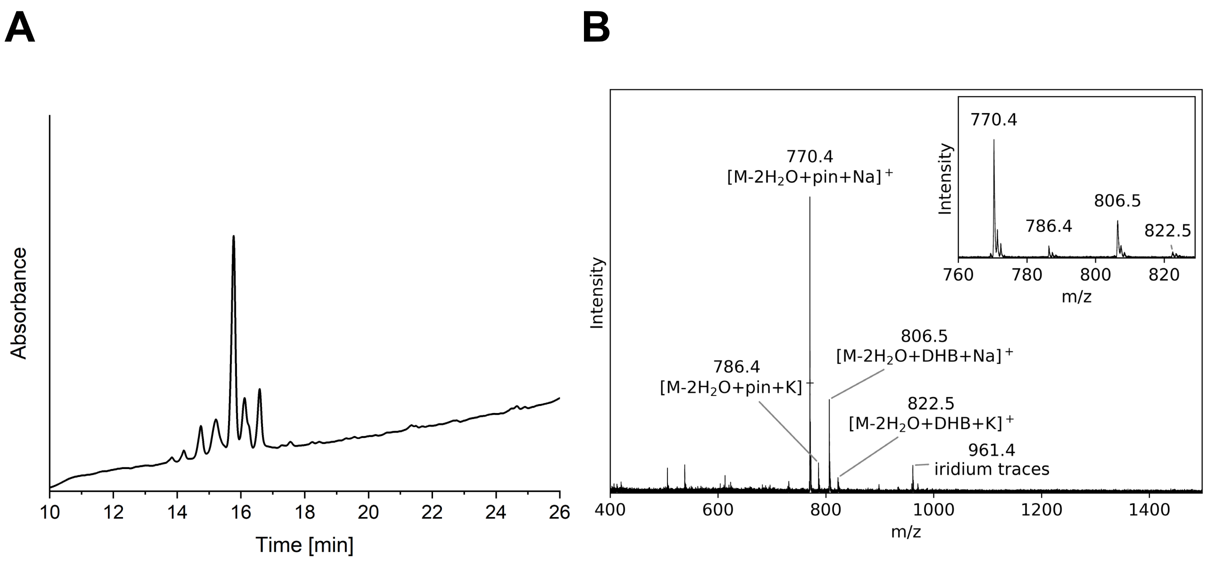

Figure S92: **P12=B(OH)$_2$** Iridium-catalyzed hydroboration. A) Analytical HPLC (220 nm, column A) of crude peptide. B) MALDI-TOF MS spectrum of crude peptide. Calculated mass: $[M-2H_2O+pin+Na]^+$ 770.4, $[M-2H_2O+pin+K]^+$ 786.3, $[M-2H_2O+DHB+Na]^+$ 806.3, $[M-2H_2O+DHB+K]^+$ 822.3.

Table S2: Yields of peptide boronic acids prepared by hydroboration based on HPLC traces.

**Peptide**	**Yield** [Ru(CO)(Cl)H(PPh_3)$_3$] (Reaction Conditions A)	**Yield** [Cp*Ru(PPh_3)$_2$Cl] (Reaction Conditions B)
**P1=B(OH)$_2$**	67%	71%
**P2=B(OH)$_2$**	45%	63%
**P3=B(OH)$_2$**	28%	37%
**P4=B(OH)$_2$**	58%	65%
**P5=B(OH)$_2$**	61%	-
**P6=B(OH)$_2$**	69%	53%
**P7=B(OH)$_2$**	undefined	undefined
**P8=B(OH)$_2$**	undefined	39%
**P9=B(OH)$_2$**	15%	29%
**P10=B(OH)$_2$**	-	-
**P11=B(OH)$_2$**	72%	76%
**P12=B(OH)$_2$**	77%	29%

Yields relate to the amount of hydroborated products including hydroborated by-products.

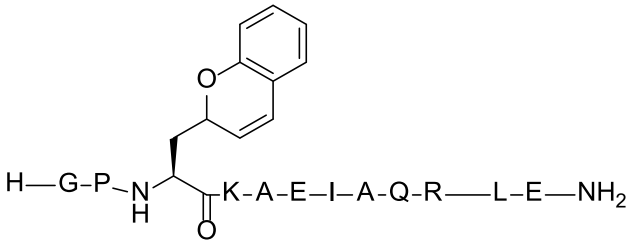

P2-1a

Chemical Formula: $C_{64}H_{102}N_{18}O_{18}$

Exact Mass: 1410,76

Molecular Weight: 1411,63

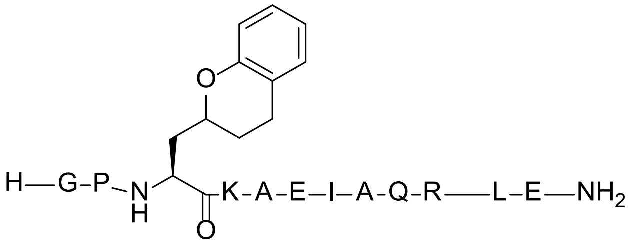

P2-1b

Chemical Formula: $C_{64}H_{104}N_{18}O_{18}$

Exact Mass: 1412,78

Molecular Weight: 1413,64

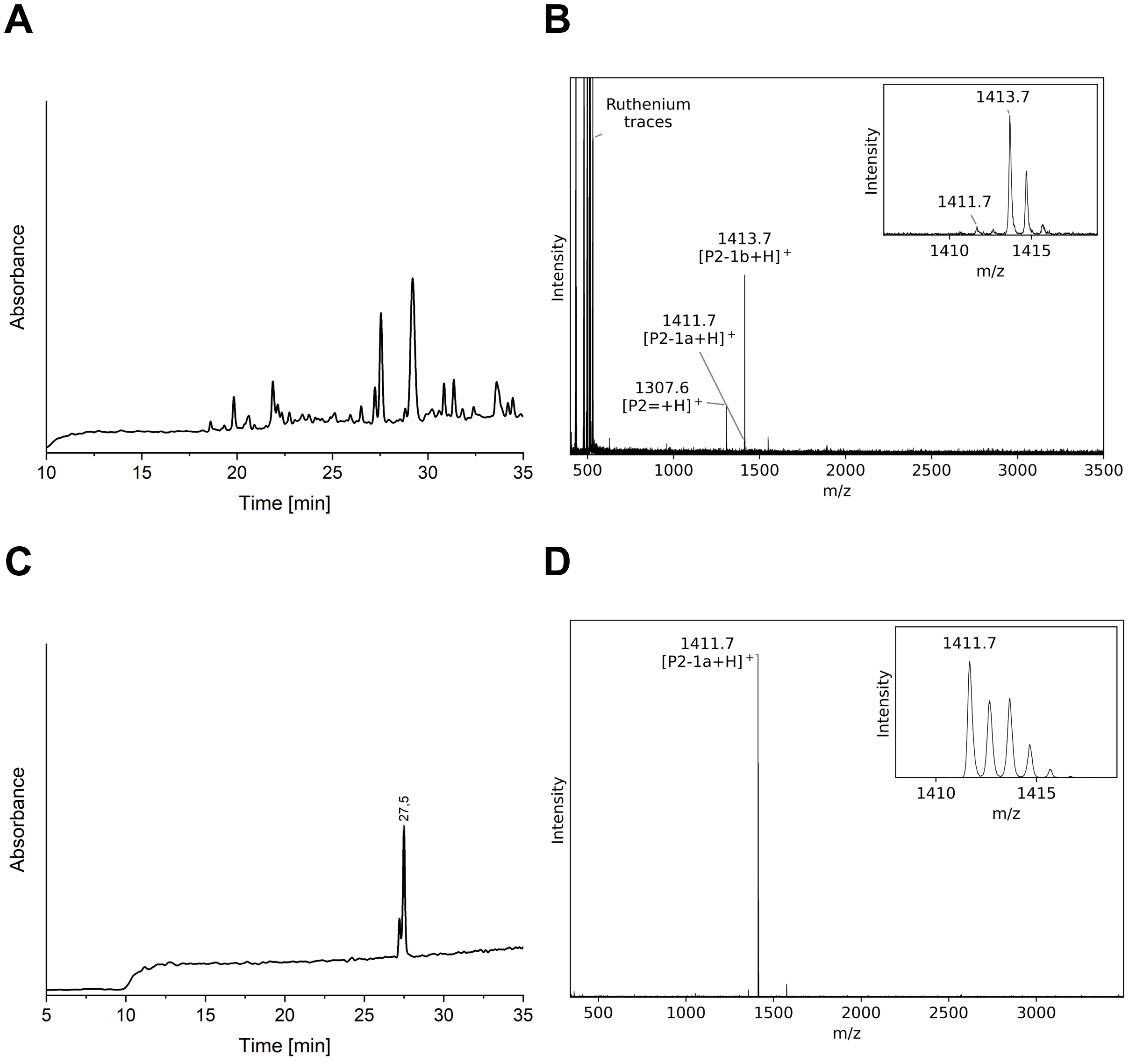

E

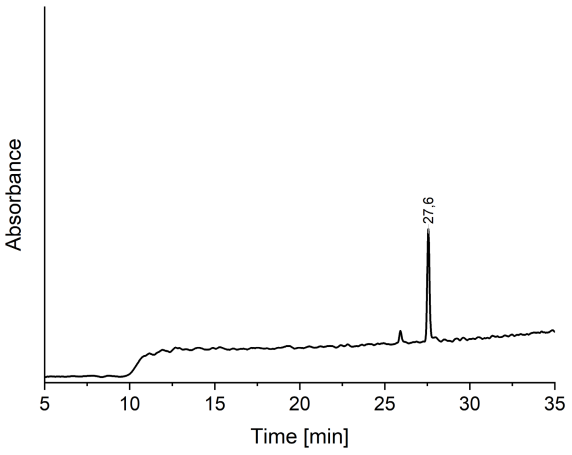

F

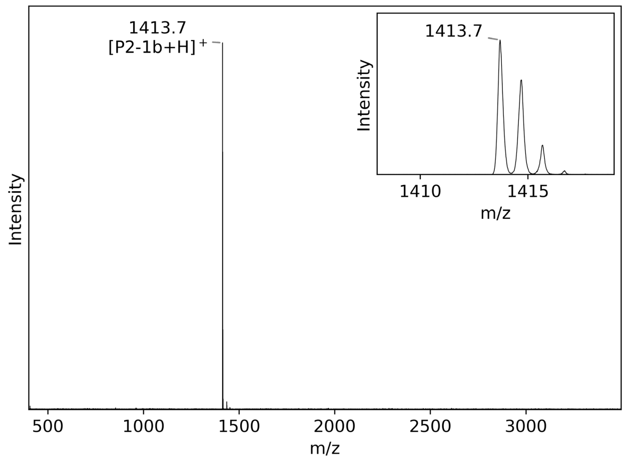

G

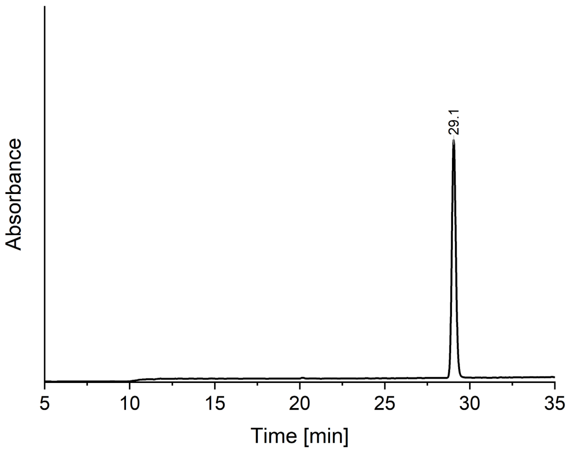

Figure S93: **P2-1** A) Analytical HPLC (220 nm, column B) of crude peptide. B) MALDI-TOF MS spectrum of crude peptide. C) Analytical HPLC (220 nm, column B) of purified peptide fraction 1. D) MALDI-TOF MS spectrum of purified peptide fraction 1. E) Analytical HPLC (220 nm, column B) of purified peptide fraction 2. F) MALDI-TOF MS spectrum of purified peptide fraction 2. G) Analytical HPLC (220 nm, column B) of salicylaldehyde. Calculated mass: [**P2=**+H]$^+$ 1307.7, [**P2-1a**+H]$^+$ 1411.8, [**P2-1b**+H]$^+$ 1413.8.

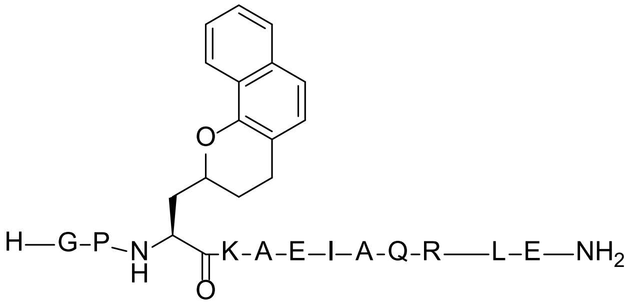

Chemical Formula: $C_{68}H_{106}N_{18}O_{18}$
Exact Mass: 1462,79
Molecular Weight: 1463,70

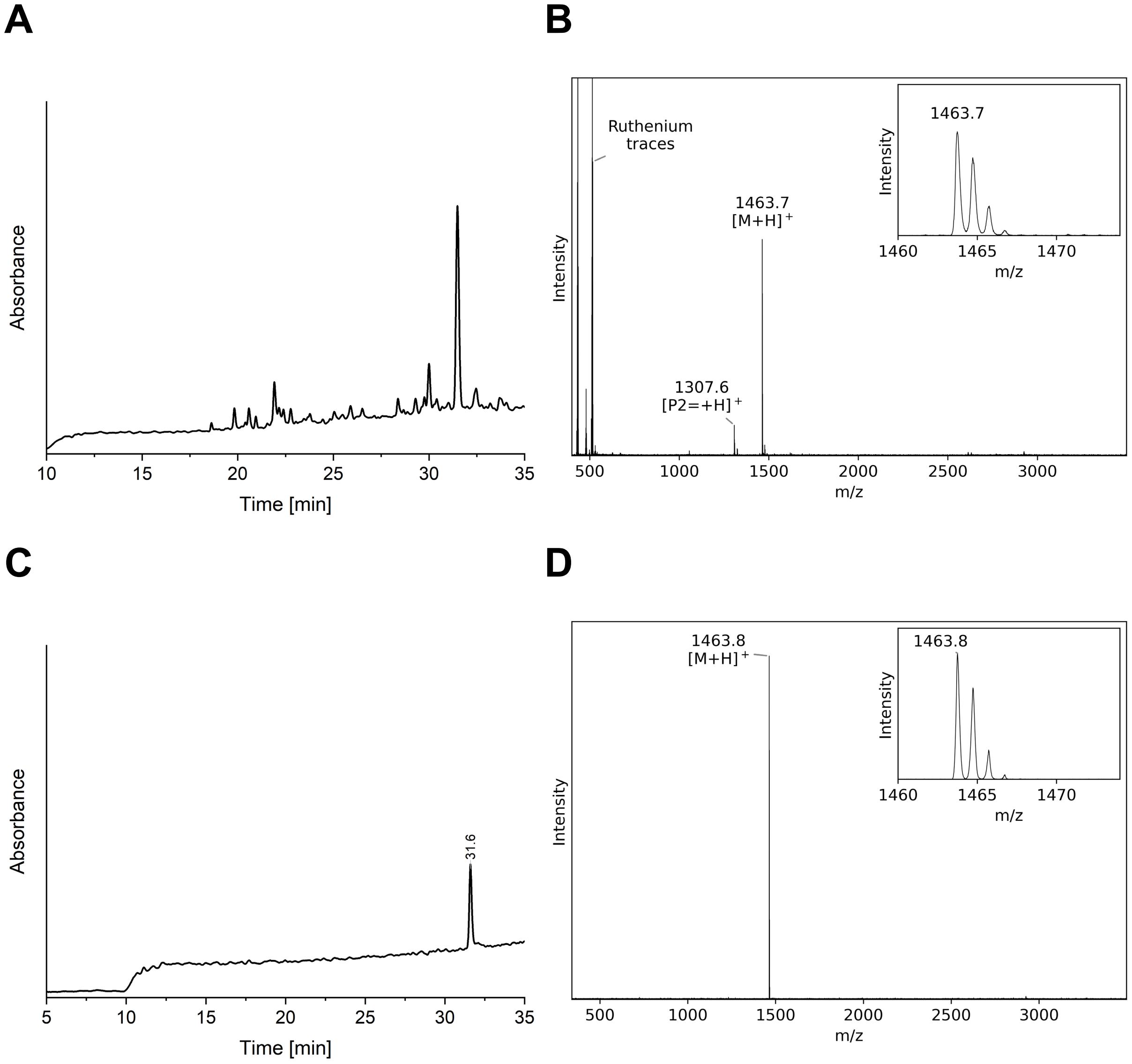

Figure S94: **P2-2** A) Analytical HPLC (220 nm, column B) of crude peptide. B) MALDI-TOF MS spectrum of crude peptide. C) Analytical HPLC (220 nm, column B) of purified peptide. D) MALDI-TOF MS spectrum of purified peptide. Calculated mass: [**P2=**+H]$^+$ 1307.7, [M+H]$^+$ 1463.8.

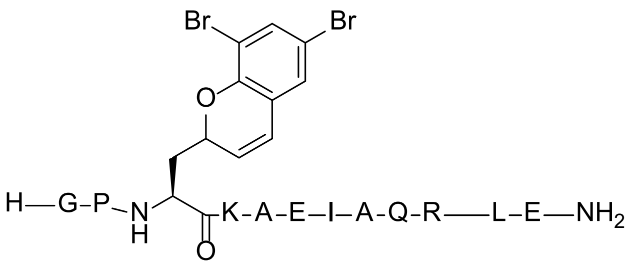

Chemical Formula: $C_{64}H_{100}Br_2N_{18}O_{18}$
Exact Mass: 1566,58
Molecular Weight: 1569,42

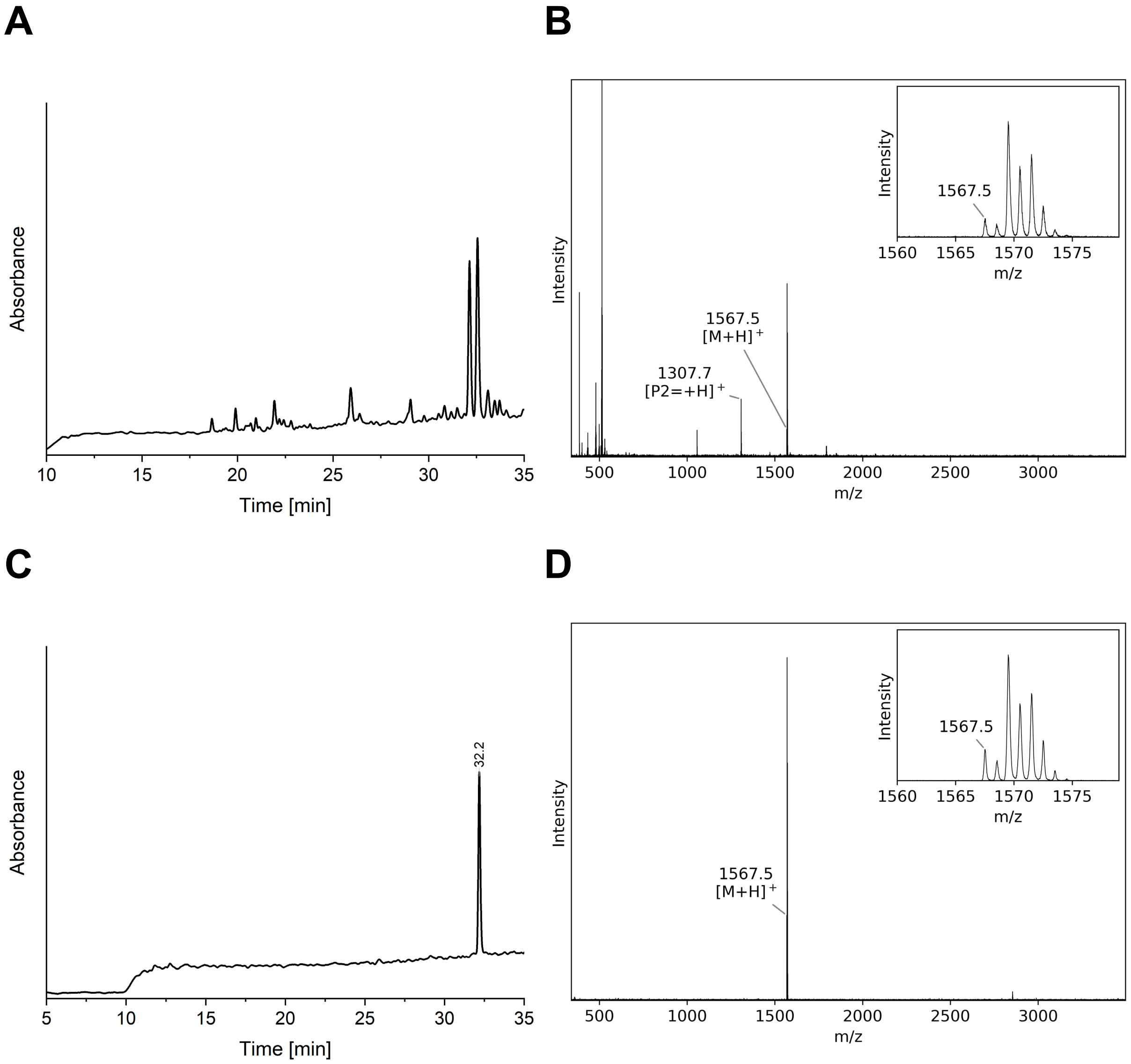

E

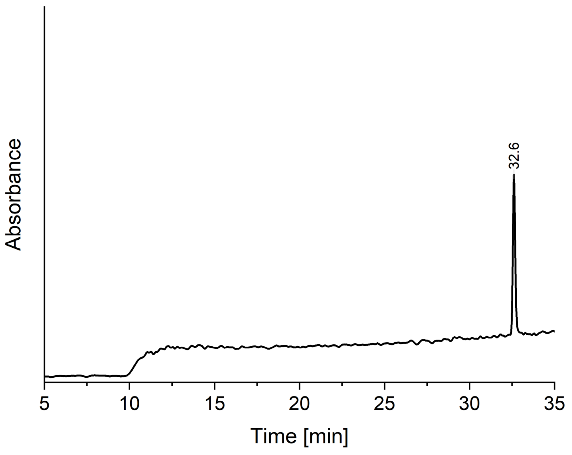

F

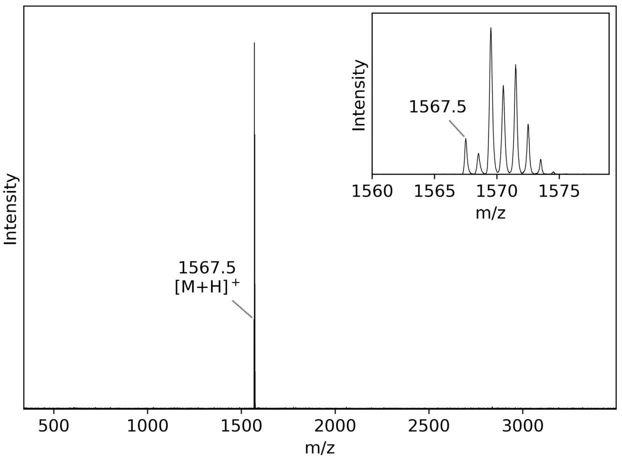

Figure S95: **P2-3** A) Analytical HPLC (220 nm, column B) of crude peptide. B) MALDI-TOF MS spectrum of crude peptide. C) Analytical HPLC (220 nm, column B) of purified peptide fraction 1. D) MALDI-TOF MS spectrum of purified peptide fraction 1. E) Analytical HPLC (220 nm, column B) of purified peptide fraction 2. F) MALDI-TOF MS spectrum of purified peptide fraction 2. Calculated mass: [**P2=**+H]$^+$ 1307.7, [M+H]$^+$ 1567.6.

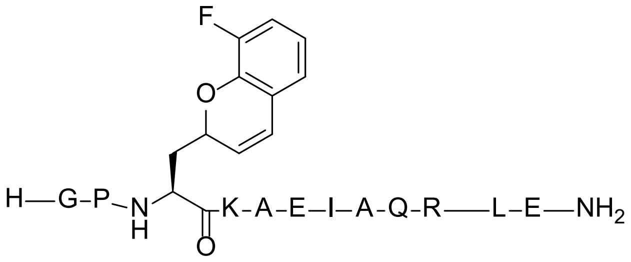

Chemical Formula: $C_{64}H_{101}FN_{18}O_{18}$
Exact Mass: 1428,75
Molecular Weight: 1429,62

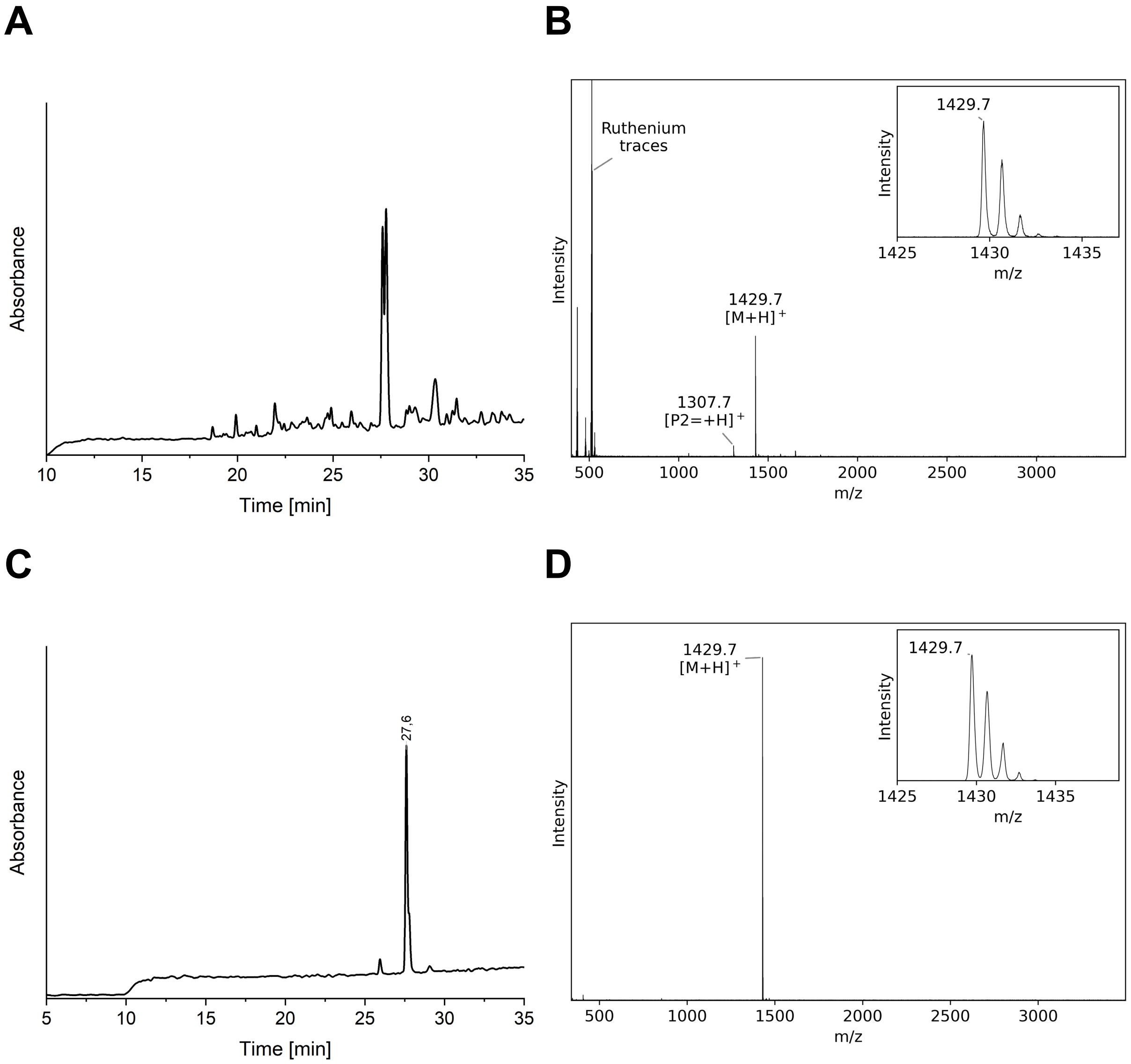

E

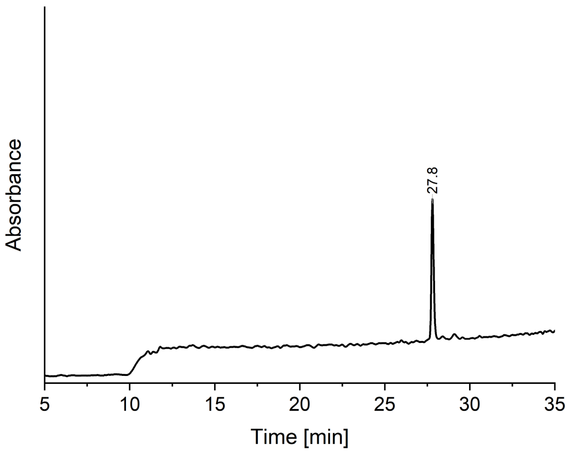

F

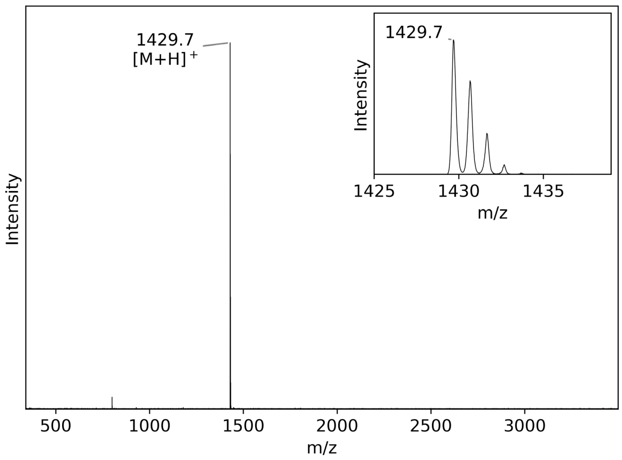

Figure S96: **P2-4** A) Analytical HPLC (220 nm, column B) of crude peptide. B) MALDI-TOF MS spectrum of crude peptide. C) Analytical HPLC (220 nm, column B) of purified peptide fraction 1. D) MALDI-TOF MS spectrum of purified peptide fraction 1. E) Analytical HPLC (220 nm, column B) of purified peptide fraction 2. F) MALDI-TOF MS spectrum of purified peptide fraction 2. Calculated mass: [**P2=**+H]$^+$ 1307.7, [M+H]$^+$ 1429.8.

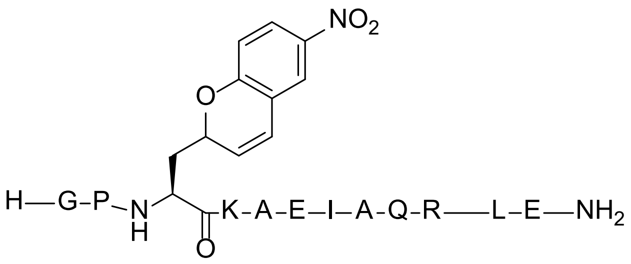

Chemical Formula: $C_{64}H_{101}N_{19}O_{20}$
Exact Mass: 1455,75
Molecular Weight: 1456,63

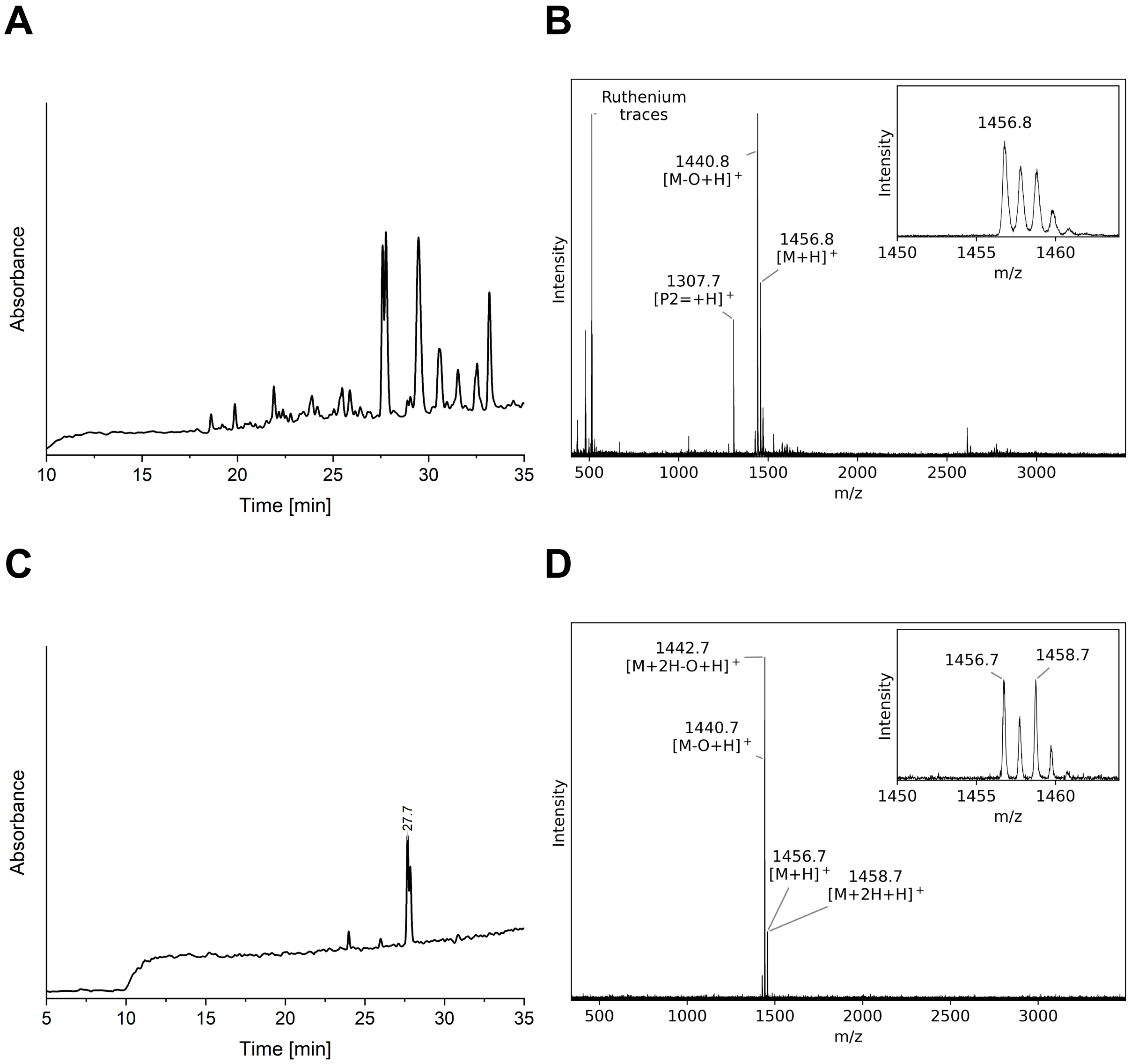

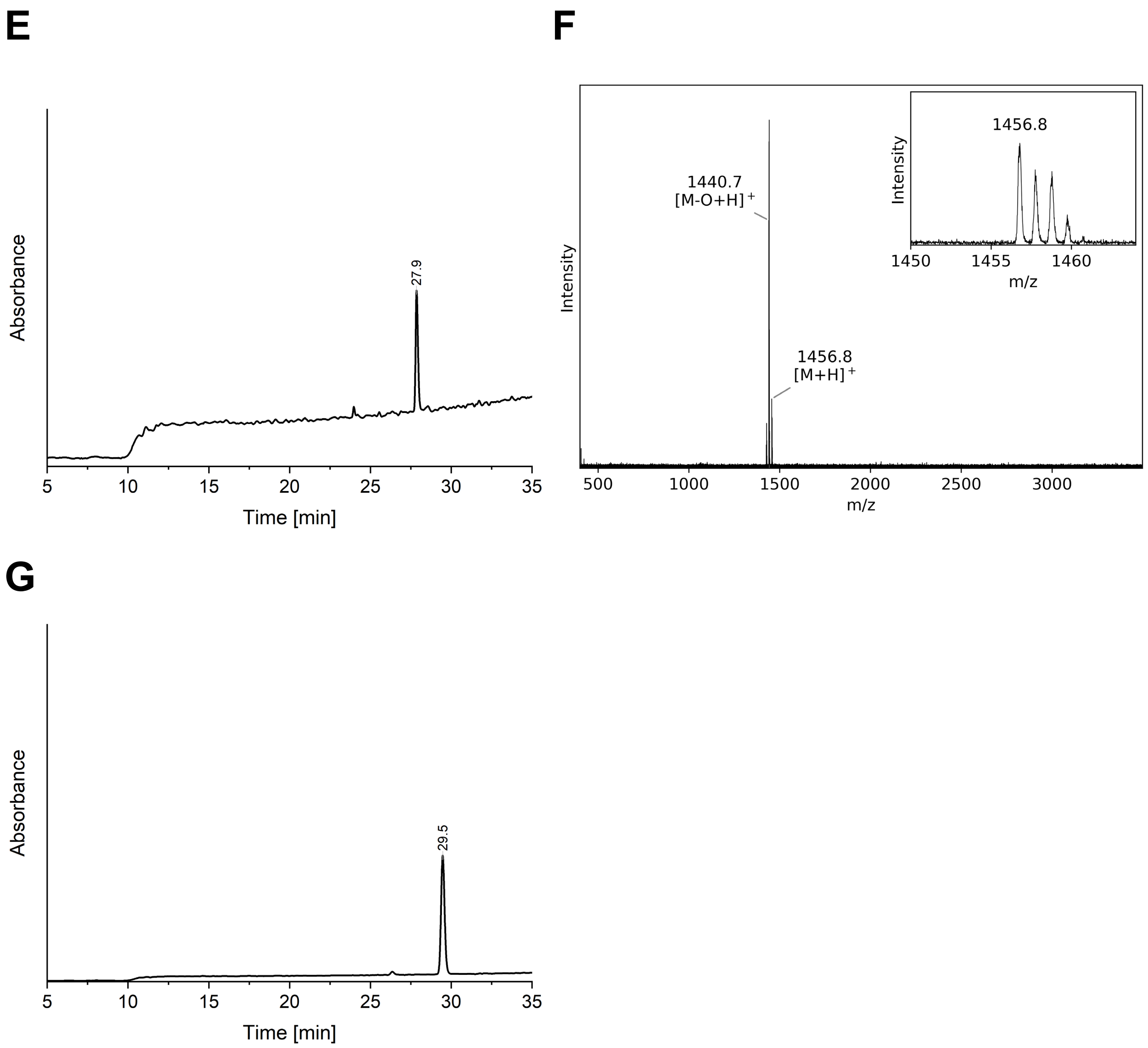

Figure S97: **P2-5** A) Analytical HPLC (220 nm, column B) of crude peptide. B) MALDI-TOF MS spectrum of crude peptide. C) Analytical HPLC (220 nm, column B) of purified peptide fraction 1. D) MALDI-TOF MS spectrum of purified peptide fraction 1. E) Analytical HPLC (220 nm, column B) of purified peptide fraction 2. F) MALDI-TOF MS spectrum of purified peptide fraction 2. G) Analytical HPLC (220 nm, column B) of 2-hydroxy-5-nitrobenzaldehyde. Calculated mass: [**P2=**+H]$^+$ 1307.7, [M-O+H]$^+$ 1440.8, [M+H]$^+$ 1456.8.

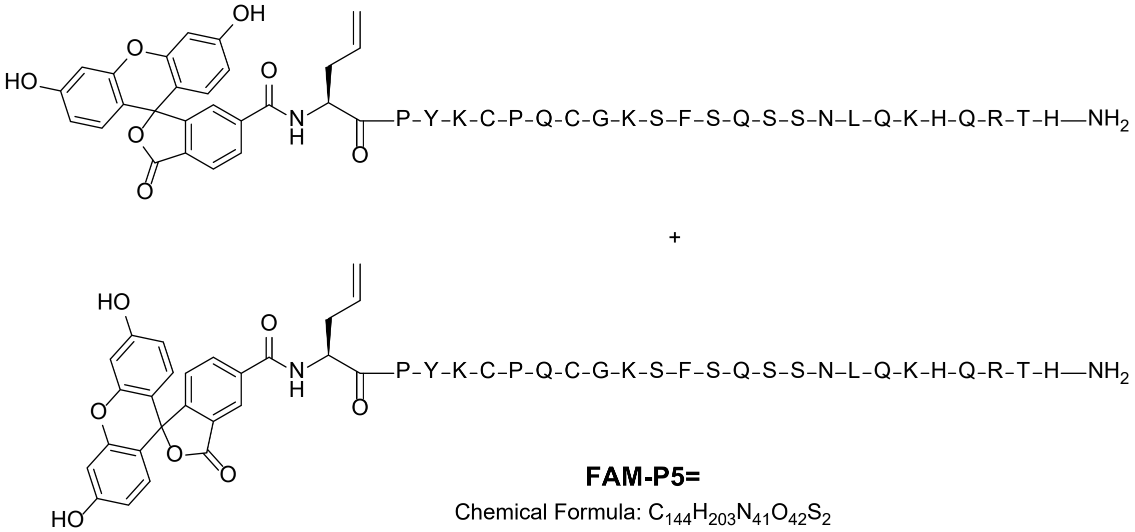

FAM-P5=

Chemical Formula: $C_{144}H_{203}N_{41}O_{42}S_2$

Exact Mass: 3242.45

Molecular Weight: 3244.57

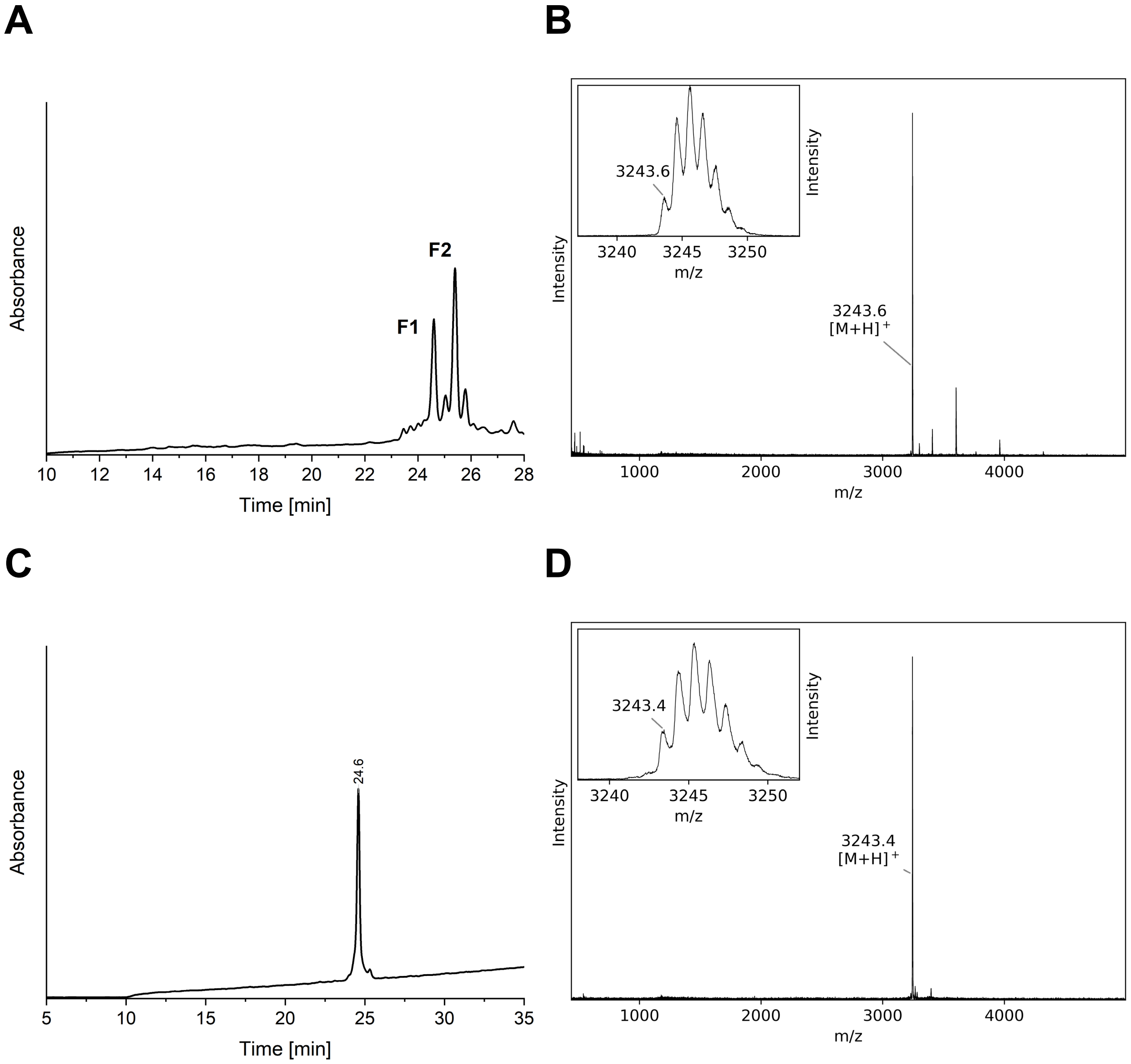

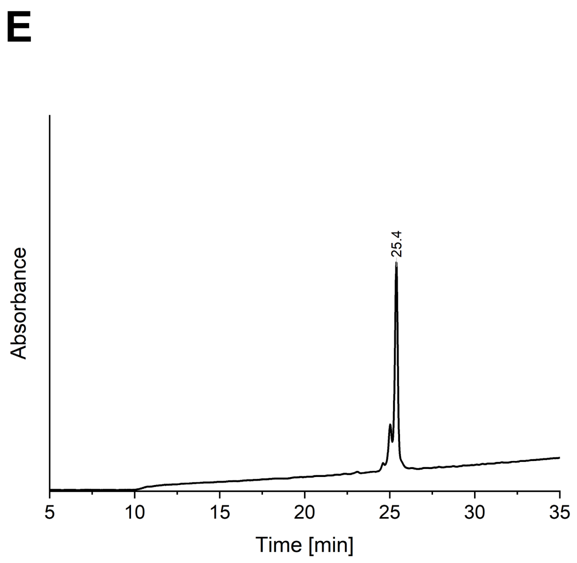

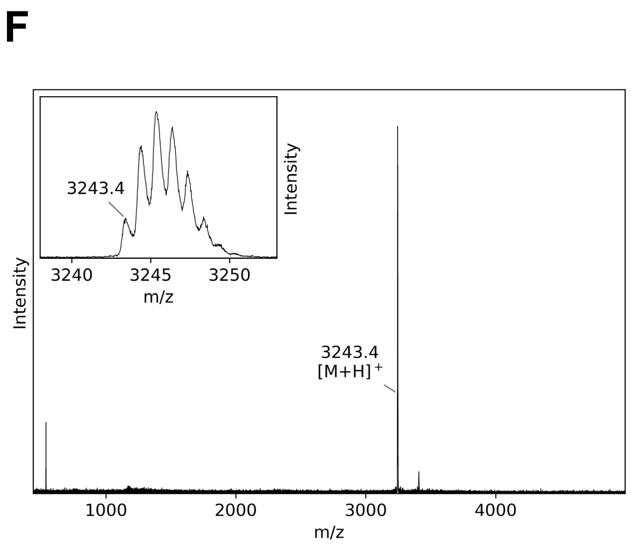

Figure S98: **FAM-P5=** A) Analytical HPLC (220 nm, column A) of crude peptide. B) MALDI-TOF MS spectrum of crude peptide. C) Analytical HPLC (220 nm, column A) of purified peptide fraction **F1**, which was used for the catch-release experiment. D) MALDI-TOF MS spectrum of purified peptide fraction **F1**, which was used for the catch-release experiment. E) Analytical HPLC (220 nm, column A) of purified peptide fraction **F2**. F) MALDI-TOF MS spectrum of purified peptide fraction **F2**. Fraction **F1** and **F2** are regioisomers. Calculated mass: $[M+H]^+$ 3243.5.

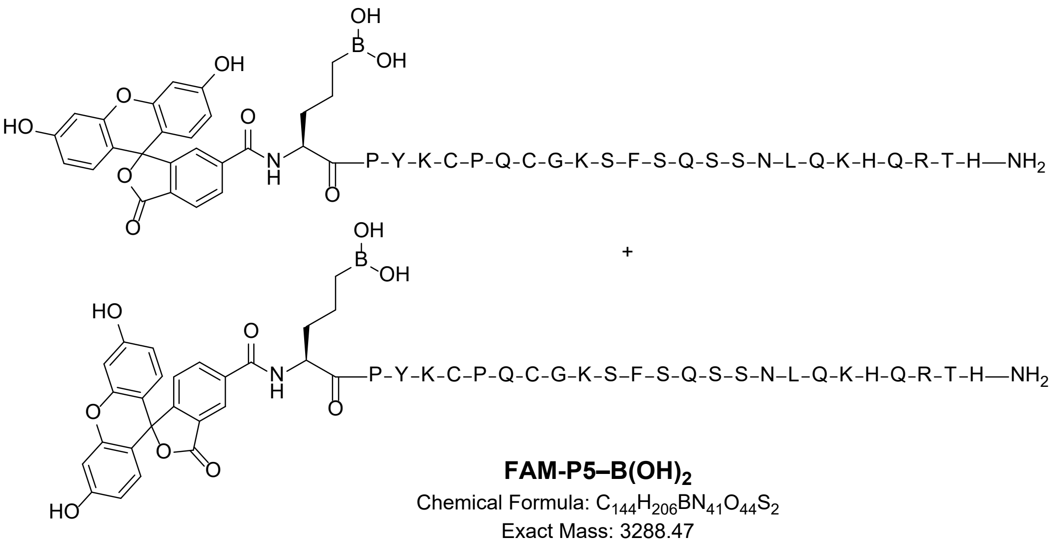

FAM-P5–B(OH)$_2$

Chemical Formula: $C_{144}H_{206}BN_{41}O_{44}S_2$

Exact Mass: 3288.47

Molecular Weight: 3290.41

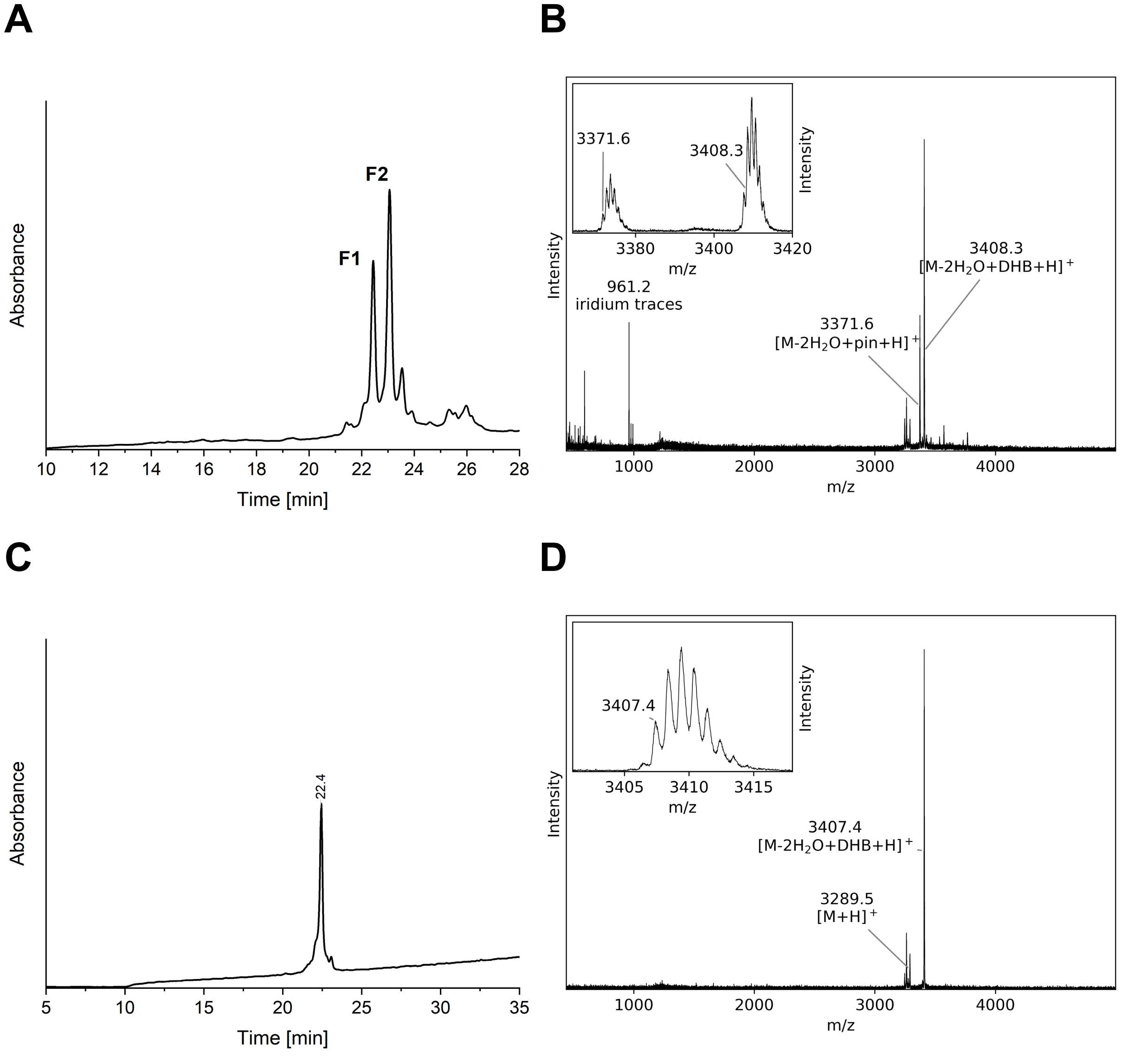

E

F

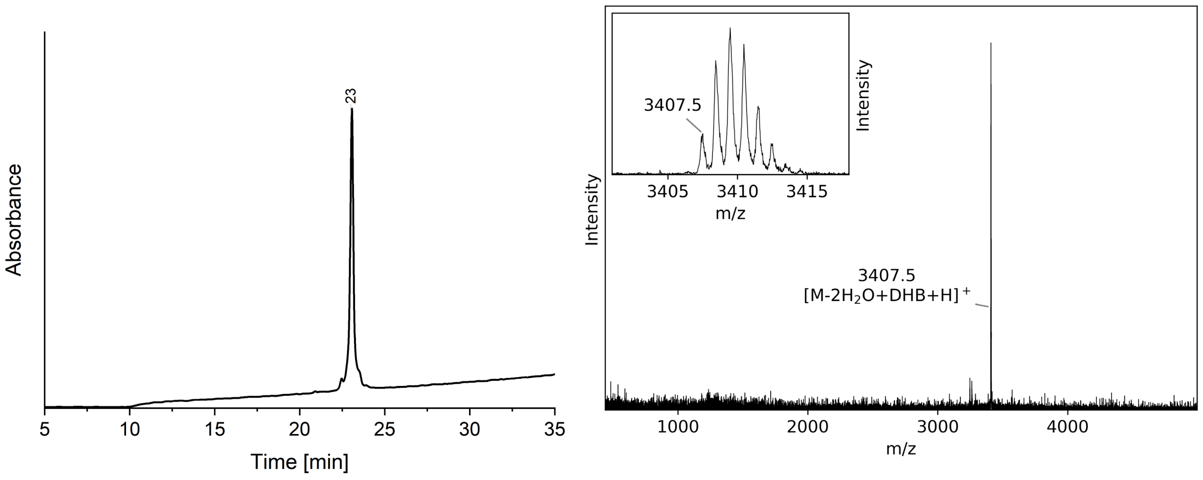

Figure S99: **FAM-P5–B(OH)$_2$** A) Analytical HPLC (220 nm, column A) of crude peptide. B) MALDI-TOF MS spectrum of crude peptide. C) Analytical HPLC (220 nm, column A) of purified peptide fraction **F1**, which was used for the catch-release experiment. D) MALDI-TOF MS spectrum of purified peptide fraction **F1**, which was used for the catch-release experiment. E) Analytical HPLC (220 nm, column A) of purified peptide fraction **F2**. F) MALDI-TOF MS spectrum of purified peptide fraction **F2**. Fraction **F1** and **F2** are regioisomers. Calculated mass: $[M+H]^+$ 3289.5, $[M-2H_2O+pin+H]^+$ 3371.6, $[M-2H_2O+DHB+H]^+$ 3407.5.

2 Experimental procedures

2.1 Reagents and solvents

Standard Fmoc-protected amino acids, Fmoc-Rink-Amide MBHA polystyrene resin and *N,N'*-diisopropylcarbodiimide (DIC) were acquired from *Iris Biotech GmbH* (Marktredwitz, Germany). Pinacolborane, Boc-Gly-OH and Fmoc-Nle-OH were purchased from *Carbolution Chemicals GmbH* (St. Ingebert, Germany). $[Ir(COD)Cl]_2$, $[Cp^*Ru(PPh_3)_2Cl]$, $[Ru(CO)(Cl)H(PPh_3)_3]$ and Fmoc-Ser(PO(OBzl)OH)-OH were purchased from *BLD Pharmatech GmbH.* (Reinbek, Germany). Oxyma®, *N,N'*-diisopropylethylamine (DIPEA), piperidine, MeCN (HPLC grade), trifluoroacetic acid (TFA, HPLC grade) and triisopropyl silane (TIPS) were acquired from *Merck KGaA* (Darmstadt, Germany). DMF (peptide synthesis grade) was purchased from *Fisher Scientific* (Loughborough, United Kingdom). All other solvents and reagents were at least *pro analysis* grade quality and were acquired from *Carbolution Chemicals GmbH* (St. Ingebert, Germany), *BLD Pharmatech GmbH.* (Reinbek, Germany), *Carl Roth GmbH + Co. KG* (Karlsruhe, Germany), *Fisher Scientific* (Loughborough, United Kingdom), *Grüssing GmbH* (Filsum, Germany), *Honeywell* (Seelze, Germany), *Merck KGaA* (Darmstadt, Germany), *neoFroxx GmbH* (Einhausen, Germany), *Th. Geyer GmbH & Co. KG* (Renningen, Germany) and *VWR International* (Fontenay-sous-Bois, France). Reagents and solvents were used as received. Dichloromethane (DCM) and toluene used as solvent for late-stage reactions were dried by a Solvent Purification System (SPS). Water was purified with a *Sartorius arium® mini* lab water system.

2.2 Software for data analysis and visualization

Data were analysed with *OriginPro® b9.5.1.195, Microsoft Excel Version 2311* and *Python 3.9* using the *matplotlib 3.8.0* and *numpy 1.24.3* packages if not stated otherwise. Chemical structures were drawn with *ChemDraw 19.0*.

2.3 Peptide synthesis, functionalization and purification

2.3.1 Automated solid-phase peptide synthesis

Peptides **P1**, **P2**, **P3**, **P4**, **P5**, **P6**, **P8**, **P9**, **P10** and **P11** were synthesized at 0.1 mmol scale and **P7** at 0.05 mmol scale on a microwave-assisted peptide synthesizer (*CEM Liberty Blue*, see section 2.3.12 for detailed settings). Fmoc-Rink-Amide MBHA polystyrene resin with a loading density of 0.67 mmol/g was used as solid support for **P1**, **P2**, **P4**, **P5**, **P6**, **P8**, **P9**, **P10** and **P11**. Fmoc-Thr-Wang polystyrene resin with a loading density of 0.38 mmol/g was used as solid support for **P3**. Fmoc-Rink-Amide Tentagel XV resin with a loading density of 0.24 mmol/g was used as solid support for **P7**. Prior to synthesis, the resin was swollen in DMF for at least 10 min. Fmoc protected amino acids were used as 0.2 M solution in DMF. *N,N'*-Diisopropylcarbodiimide (DIC) dissolved in DMF (0.5 M) was used as activator, and Oxyma® solution in DMF (1.0 M) plus 0.1 M *N,N*-diisopropylethylamine (DIPEA) was used as the activator base. A solution of piperidine/DMF 1:4 *v/v* was used to remove the Fmoc protecting group. To prevent aspartimide formation,[2] deprotection solution for **P3** and **P10** contained 5% (v/v) formic acid and deprotection solution for **P7** contained 1 M Oxyma®. After the synthesis, the peptide was transferred into a 10 mL syringe reactor with a PE frit and washed with DMF (5 x 5 mL), MeOH (5 x 5 mL), DCM (5 x 5 mL). For *N*-terminal acetylation, the peptide resin was further washed with DMF (3 x 5 mL) and treated with acetic anhydride (0.5 mL) in pyridine (4.5 mL) for 10 min, then washed with DMF (3 x 5 mL) and DCM (10 x 5 mL). The peptide resin was dried under reduced pressure in a desiccator. The mass of the resin was determined and it was assumed, that this corresponds to 0.1 mmol peptide or 0.05 mmol in case of **P7**.

2.3.2 Manual solid-phase peptide synthesis of P12

P12 was prepared by manual solid-phase peptide synthesis in a 0.1 mmol scale. Fmoc-Rink-Amide MBHA polystyrene resin with a loading density of 0.67 mmol/g was used as solid support. Prior to synthesis, the resin was swollen in DMF for at least 10 min. The solid-phase peptide synthesis was carried out in a 10 mL syringe reactor equipped with a PE frit. The standard cycle for peptide synthesis consists of 1) deprotection, 2) wash, 3) coupling and 4) wash.

1) **Deprotection**: To remove the Fmoc protecting group, the resin was treated with a solution of 20 % piperidine in DMF (5 mL) and the syringe reactor was shaken for 20 min.

2) **Wash:** The solution was discarded and the resin was washed with DMF (3×4 mL), DCM (3 × 4 mL) and DMF (3 × 4 mL).
3) **Coupling:** The protected amino acid (400 µmol, 4.00 eq.) was dissolved in DMF (4 mL), before DIPEA (140 µL, 800 µmol, 8.00 eq.) and PyBOP (187 mg, 360 µmol, 3.60 eq.) were added. After one minute of incubation, the resin was treated with the coupling reagent and the syringe reactor was shaken for one hour. Coupling was performed with the respective protected amino acids listed in Table S3.

Table S3: Synthesis of **P12** part 1.

	Amino acid
**1**	G
**2**	G
**3**	F

For the synthesis of **P12≡** and **P12=**, the H-FGG-Rink-Amide resin was splited in two parts, each containing 50 µmol peptide. The synthesis of the peptoid was performed as described in the literature.[3]

1) **Coupling of bromoacetic acid**: A solution of bromoacetic acid (2.0 M in DMF, 1 mL per coupling) and a solution of DIC (2.0 M in DMF, 1 mL per coupling) were added to the resin (50 µmol peptide) in a microwave-reaction vessel The reaction was performed in a *CEM Discovery SP* microwave oven at 35 °C and 300 W while stirring for 30 s. The mixture was transferred to a 10 mL syringe reactor with a PE frit and the resin was washed with DMF (5 x 5 mL) and anhydrous DMF (2 x 5 mL).
2) **Nucleophilic substitution with amines:** The resin was transferred into a new microwave reaction vessel with 2 mL anhydrous DMF in total. A solution of either propargylamine (1.0 M in DMF, 2 mL per coupling) for the synthesis of **P12≡** or allylamine (1.0 M in DMF, 2 mL per coupling) for the synthesis of **P12=** was added. The reaction was performed in a *CEM Discovery SP* microwave oven at 95 °C and 300 W while stirring for 90 s. The mixture was transferred to a 10 mL syringe reactor with a PE frit and subsequently washed with DMF (3 x 5 mL) and DCM (5 x 5 mL). The resin was dried under reduced pressure.

SPPS was continued with linearly scaled down reagents. Coupling was performed with the respective protected amino acids listed in Table S4.

Table S4: Synthesis of **P12** part 2.

	Amino acid
**5**	F
**6**	G

For *N*-terminal acetylation the peptide resin was further washed with DMF (3 x 2.5 mL) and treated with acetic anhydride (0.25 mL) in pyridine (2.25 mL) for 10 min, then washed with DMF (3 x 2.5 mL) and DCM (10 x 2.5 mL). The peptide resin was dried under reduced pressure in a desiccator.

2.3.3 Manual solid-phase peptide synthesis of FAM-P5= and FAM-P5-B(OH)$_2$

Resin referring to 5 µmol peptide of **P5=** and **P5-B(OH)$_2$**, prepared by the iridium-catalyzed hydroboration**,** was placed in a 2 mL syringe reactor with a PE frit. A solution of 5(6)-carboxyfluorescin (18.8 mg, 50 µmol, 10 eq.), HOBt x H_2O (7.6 mg, 50 µmol, 10 eq.) and DIC (9 µL, 50 µmol, 10 eq.) in 0.5 mL DMF was prepared and added to the resin. The mixture was shaken at room temperature for 3.5 h. The solution was discarded and the resin was washed with DMF (5 x 1 mL) and DCM (10 x 1 mL) and then dried under reduced pressure.

2.3.4 Cleavage and isolation of peptides

Cleavage of the peptide from the resin and final deprotection were carried out in trifluoroacetic acid (TFA) containing triisopropyl silane (TIPS) and water. Dry resin with 5 µmol peptide was placed into a 2 mL syringe reactor with a PE frit and incubated with one of the cleavage cocktails summarized in Table S5. The solution was collected and the resin was washed with TFA (2 x 1 mL). The TFA was removed in a stream of nitrogen and the peptide was precipitated from ice-cold Et_2O (10 mL). The peptide was isolated by centrifugation. The pellet was washed with Et_2O (2 x 10 mL) and dissolved in acetonitrile/water mixtures containing TFA, except for **P8**, which was dissolved in 0.4 mL HFIP (1,1,1,3,3,3-Hexafluoro-2-propanol) and, after 1 h incubation time,

diluted with 2.1 mL water (Table S5), frozen in liquid N_2 and lyophilized (*Christ Alpha 2-4-LDplus* connected to a *VACUUBRAND RZ 6* pump).

Table S5: Cleavage cocktails, incubation time and solvent mixtures for peptides **P1** to **P12**. A: water + 0.1% TFA, B: MeCN + 0.1% TFA.

Peptide	TFA [µL]	TIPS [µL]	water [µL]	incubation time [h]	solvent mixture/volume
**P1 and P12**	950	25	25	1	20% B in A / 2.5 mL
**P2, P3, P5, P6, P7, P9, P10 and P11**	950	25	25	3	20% B in A / 2.5 mL
**P4**	950	25	25	4	20% B in A / 2.5 mL
**P8**	950	25	25	3	0.4 mL HFIP + 2.1 mL water

2.3.5 Late-stage iridium-catalyzed hydroboration of alkene-containing peptides

Resin loaded with 5 µmol peptide **P1=** to **P12=** was placed in a 1.5 mL microcentrifuge tube. $[Ir(COD)Cl]_2$ and phosphine ligand were added to the resin. In a nitrogen-flushed desiccator, 0.5 mL dry DCM was added to the resin, followed by DIPEA and pinacolborane. The microcentrifuge tube was closed and sealed with parafilm. The reaction mixture was shaken for 16 h at room temperature.
The resin was transferred with DCM to a 2 mL syringe reactor equipped with a PE frit and washed with DCM (15 x 1 mL). The resin was dried under reduced pressure. The peptide was cleaved from the resin as described in section 2.3.4.

Table S6: Amount of catalyst, ligand, pinacolborane and DIPEA, and reaction time for iridium-catalyzed hydroboration of alkenes.

Peptide	Catalyst	Ligand	DIPEA	Pinacolborane	reaction time [h]
**P1, P2, P4, P5, P6 and P11**	$[Ir(COD)Cl]_2$ (6.7 mg, 10 µmol, 4 eq. referring to one [Ir])	dppm (7.7 mg, 20 µmol, 4 eq.)	9 µL, 50 µmol, 10 eq.	73 µL, 0.5 mmol, 100 eq.	16
**P3 and P7**	$[Ir(COD)Cl]_2$ (6.7 mg, 10 µmol, 4 eq. referring to one [Ir])	PCy_3 (11.2 mg, 40 µmol, 8 eq.)	9 µL, 50 µmol, 10 eq.	73 µL, 0.5 mmol, 100 eq.	16
**P8 and P10**	$[Ir(COD)Cl]_2$ (13.4 mg, 20 µmol, 8 eq. referring to one [Ir])	PCy_3 (22.4 mg, 80 µmol, 16 eq.)	18 µL, 100 µmol, 20 eq.	146 µL, 1 mmol, 200 eq.	88
**P9**	$[Ir(COD)Cl]_2$ (6.7 mg, 10 µmol, 4 eq. referring to one [Ir])	PCy_3 (11.2 mg, 40 µmol, 8 eq.)	9 µL, 50 µmol, 10 eq.	73 µL, 0.5 mmol, 100 eq.	44

<u>Protocol for entries from Table 1</u>
Resin loaded with 5 µmol peptide **P1=** was hydroborated in a similar way as stated above with 4 eq. (referring to one [Ir]) of iridium catalyst and 4 eq. of bis(phosphine) ligand listed in Table 1 without addition of DIPEA. In case of entry 6, 8 eq. of PCy_3 was added with 4 eq. (referring to one [Ir]) of iridium catalyst together with DIPEA (9 µL, 50 µmol, 10 eq.).

2.3.6 Late-stage iridium-catalyzed hydroboration of alkyne-containing peptides

Resin loaded with 5 µmol peptide **P1≡** to **P12≡**, except of **P7≡**, was placed in a 1.5 mL microcentrifuge tube. $[Ir(COD)Cl]_2$ (6.7 mg, 10 µmol, 4 eq. referring to one [Ir]) and dppm (7.7 mg, 20 µmol, 4 eq.) were added to the resin. In a nitrogen-flushed desiccator, 0.5 mL dry DCM was added to the resin, followed by pinacolborane (73 µL, 0.5 mmol, 100 eq.). The microcentrifuge tube was closed and sealed with parafilm. The reaction mixture was shaken for 16 h at room temperature.
The resin was transferred with DCM to a 2 mL syringe reactor equipped with a PE frit and washed with DCM (15 x 1 mL). The resin was dried under reduced pressure. The peptide was cleaved from the resin as described in section 2.3.4.

2.3.7 Late-stage [Ru(CO)(Cl)H(PPh_3)$_3$]-catalyzed hydroboration of alkyne-containing peptides (Reaction Conditions A)

Resin loaded with 5 µmol peptide was placed in a 1.5 mL microcentrifuge tube. [Ru(CO)(Cl)H(PPh_3)$_3$] (19 mg, 20 µmol, 4 eq.) was added to the resin. In a nitrogen-flushed desiccator, dry toluene was added to the resin, followed by pinacolborane. The microcentrifuge tube was closed and sealed with parafilm. The reaction mixture was shaken at 40 °C on a *VWR Incubating Microplate Shaker* for the time indicated in Table S7.
The resin was transferred with toluene to a 2 mL syringe reactor equipped with a PE frit and washed with toluene (5 x 1 mL) and DCM (10 x 1 mL). The resin was dried under reduced pressure. The peptide was cleaved from the resin as described in section 2.3.4.

Table S7: Amount of toluene and pinacolborane, and reaction time for [Ru(CO)(Cl)H(PPh_3)$_3$]-catalyzed hydroboration.

Peptide	Toluene [mL]	Pinacolborane	reaction time [h]	Temperature [°C]
**P1**	0.8	218 µL, 1.5 mmol, 300 eq.	48	40
**P2 to P10**	0.5	73 µL, 0.5 mmol, 100 eq.	64	40
**P11**	0.5	73 µL, 0.5 mmol, 100 eq.	16	25
**P12**	0.5	73 µL, 0.5 mmol, 100 eq.	16	40

2.3.8 Late-stage [Cp*Ru(PPh_3)$_2$Cl]-catalyzed hydroboration of alkyne-containing peptides (Reaction Conditions B)

Resin loaded with 5 µmol peptide **P1≡** to **P12≡** was placed in a 1.5 mL microcentrifuge tube. [Cp*Ru(PPh_3)$_2$Cl] (6 mg, 7.5 µmol, 1.5 eq.; for **P12≡**: 2 mg, 2.5 µmol, 0.5 eq.) was added to the resin. In a nitrogen-flushed desiccator, 0.5 mL dry DCM was added to the resin, followed by pinacolborane (73 µL, 0.5 mmol, 100 eq.). The microcentrifuge tube was closed and sealed with parafilm. The reaction mixture was shaken for 16 h at room temperature. The resin was transferred with DCM to a 2 mL syringe reactor equipped with a PE frit and washed with DCM (15 x 1 mL). The resin was dried under reduced pressure. The peptide was cleaved from the resin as described in section 2.3.4.

2.3.9 Attempted late-stage zirconium-catalyzed hydroboration of alkyne-containing peptides

Resin loaded with 5 µmol peptide **P1≡** was placed in a 2 mL syringe reactor with PE frit as reaction vessel. The catalyst [ZrCp$_2$(H)Cl] (5.16 mg, 20.0 µmol, 4.00 eq.) was added to the resin, followed by adding a solution of pinacolborane (72.5 µL, 500 µmol, 100 eq.) and Et_3N (7.00 µL, 50.0 µmol, 10.0 eq.) in dry DCM (1.00 mL) under a nitrogen atmosphere. The syringe reactor was shaken overnight at room temperature. The solution was discarded and the resin was washed with DCM (15 x 1 mL). The resin was dried under reduced pressure. The peptide was cleaved from the resin as described in section 2.3.4.

2.3.10 Attempted late-stage [Rh(COD)Cl]$_2$/2dppm-catalyzed hydroboration of alkyne-containing peptides

Resin loaded with 5 µmol peptide **P1≡** was placed in a 2 mL syringe reactor with PE frit. [Rh(COD)Cl]$_2$ (4.93 mg, 10 µmol, 4 eq. referring to one [Rh]) and dppm (7.7 mg, 20 µmol, 4 eq.) were added to the resin. In a nitrogen-flushed desiccator, 0.5 mL dry DCM was added to the resin, followed by pinacolborane (73 µL, 0.5 mmol, 100 eq.). The reaction mixture was shaken for 16 h at room temperature.
The resin was washed with DCM (15 x 1 mL) and dried under reduced pressure. The peptide was cleaved from the resin as described in section 2.3.4.

2.3.11 Attempted late-stage [Rh(CO)(PPh_3)$_2$Cl]-catalyzed hydroboration of alkyne-containing peptides

Resin loaded with 5 µmol peptide **P1≡** was placed in a 1.5 mL microcentrifuge tube. [Rh(CO)(PPh_3)$_2$Cl] (13.8 mg, 20 µmol, 4 eq. was added to the resin. In a nitrogen-flushed desiccator, 0.5 mL dry DCM was added to the resin, followed by pinacolborane (73 µL, 0.5 mmol, 100 eq.). The reaction mixture was shaken for 16 h at room temperature.

was transferred with DCM to a 2 mL syringe reactor equipped with a PE frit and washed with DCM (15 x 1 mL) and dried under reduced pressure. The peptide was cleaved from the resin as described in section 2.3.4.

2.3.12 Late-stage Petasis-Borono-Mannich reaction

Resin loaded with 5 µmol peptide **P2≡** was hydroborated as described in section 2.3.8 without the peptide being cleaved from the resin. Petasis-Borono-Mannich reaction was performed after the resin was dried under reduced pressure. The resin was transferred into a 1.5 mL microcentrifuge tube together with 1 mL dry 1,4-dioxane, 96 µL dibenzylamine (0.5 mmol, 100 eq.) and salicylaldehyde derivative (0.5 mmol, 100 eq.). The mixture was shaken for 16 h at 80 °C using a *Hettich Benelux MKR 13 Thermomixer*. The resin was transferred with DCM to a 2 mL syringe reactor equipped with a PE frit and washed with DCM (15 x 1 mL). The resin was dried under reduced pressure. The peptide was cleaved from the resin as described in section 2.3.4.

2.3.13 High-performance liquid chromatography (HPLC)

The peptides were purified by reversed-phase HPLC on a *VDS optilab VDSpher® PUR 100 C18-SE* (250 mm x 10 mm, 100 Å, 5 µm) column at a flow rate of 3 mL/min at 50 °C. A *Jasco* chromatography system with a *PU-4180* pump, a *CO-4060* column thermostat and a *UV-4070* detector was used. Analytical HPLC was performed using a *Hitachi Primaide* chromatography system containing a *1110 Pump*, a *1210 auto sampler*, *1310 column oven* and a *1430 diode array detector*. A *VDS optilab VDSpher® PUR 100 C18-SE* (250 mm x 4.6 mm, 100 Å, 5 µm) column and a flow rate of 1 mL/min at 50 °C were used. During the course of this study, the original column of the analytical HPLC (column A) was replaced with a new column of the same type (column B). Preparative chromatograms were monitored at 220 nm and 280 nm. Analytical chromatograms were monitored from 190 to 400 nm. Fractions containing pure peptide were identified by analytical HPLC and MALDI-TOF MS, pooled and lyophilized. As solvent system, water + 0.1% TFA (Buffer A) and MeCN + 0.1% TFA (Buffer B) was used. The gradients are displayed in Table S8. Prior to injection, peptide solutions were filtered with H-PTFE syringe filters (0.2 µm pore size) from *Macherey-Nagel*.
The preparation of **P8** for HPLC was performed similar to previously reported hydrophobic sequences.[4] Crude peptide **P8** was dissolved in 200 µL HFIP and incubated for at least 2 h. 100 µL of the peptide solution was mixed with 1380 µL purified water, 320 µL acetic acid and 200 µL Buffer B. The mixture was filtered with H-PTFE syringe filters (0.2 µm pore size) from *Macherey-Nagel* and immediately injected.

Table S8: Gradients used for the purification and characterization of peptides **P1** to **P12**. The percentages of Buffer B in Buffer A (*v*/*v*) are given.

preparative	Isocratic 1	linear gradient	Isocratic 2	Isocratic 3
**time / min**	0 → 8	8 → 38	39 → 44	45 → 50
**P1 and P12**	10%	10 – 80%	100%	10%
**P2, P4, P5, P8 and P5**	10%	10 – 40%	100%	10%
**P5**	20%	20 – 60%	100%	20%
**P6**	20%	20 – 45%	100%	20%
**P7 and P10**	20%	20 – 45%	100%	20%
**P9**	30%	30 – 80%	100%	30%
**P11**	5%	5 – 25%	100%	5%
**analytical**				
**time / min**	0 → 5	5 → 35	36 → 41	42 → 50
**P1 and P6**	10%	10 – 80%	95%	10%
**P2, P3 and P4**	10%	10 – 40%	95%	10%
**P5**	20%	20 – 60%	95%	20%
**P6**	20%	20 – 45%	95%	20%
**P7 and P10**	20%	20 – 45%	95%	20%
**P9**	30%	30 – 80%	95%	30%
**P11**	5%	5 – 25%	95%	5%
**P12**	20%	20 – 45%	95%	20%

2.3.14 Mass determination

The mass of lyophilized peptides was determined by weighing on a *VWR Sw.c13.01* balance.

2.3.15 Settings of the peptide synthesizer

The following Tables (S8-S14) contain the detailed settings for the microwave-assisted peptide syntheses performed in this study using the *CEM LibertyBlue* peptide synthesizer.

Table S9: Settings for **Microwave Methods**. Mixing of the reaction mixture was performed by bubbling nitrogen through the reaction vessel frit (bubble for 2 s, off for 3 s).

Microwave Method	Temperature / °C	Power / W	Hold Time / s	DeltaT / °C
Standard deprotection	75	155	15	2
	90	30	50	1
Conventional deprotection	25	0	300	2
Coupling	75	170	15	2
	90	30	110	1
Conventional coupling	25	0	3600	2
50 °C 10 min Coupling	25	0	120	2
	50	35	480	1

Table S10: Settings for **Resin Swelling**.

	Cycle Steps	Parameter values
1	Swell Resin	Main solvent volume: 15 mL, Time: 300 s

Table S11: Settings for **Single Coupling**.

	Cycle Steps	Parameter values
1	Deprotection	Reaction Method: Standard Deprotection, Deprotection Volume: 4 mL
2	Wash	Volume: 4 mL, Drain Time: 10 s
3	Wash	Volume: 4 mL, Drain Time: 5 s
4	Wash	Volume: 4 mL, Drain Time: 5 s
5	Wash	Volume: 4 mL, Drain Time: 5 s
6	Coupling	Reaction Method: Standard coupling, Amino Acid: from method, Amino Acid Volume: 2.5 mL, Activator Bottle Position: PositionACT, Activator Volume: 2 mL, Activator Base Position: PositionACTB, Activator Base Volume: 1 mL, Delayed Reagent Time: 0 s, Delayed Reagent Bottle Position: PositionACTB, Delayed Reagent Volume: 0 mL, Manifold Wash Volume: 2 mL
7	Wash	Volume: 4 mL, Drain Time: 10 s
8	Wash	Volume: 4 mL, Drain Time: 5 s

Table S12. Settings for **Single 50 °C Coupling**.

	Cycle Steps	Parameter values
1	Deprotection	Reaction Method: Standard Deprotection, Deprotection Volume: 4 mL
2	Wash	Volume: 4 mL, Drain Time: 10 s
3	Wash	Volume: 4 mL, Drain Time: 5 s
4	Wash	Volume: 4 mL, Drain Time: 5 s
5	Wash	Volume: 4 mL, Drain Time: 5 s
6	Coupling	Reaction Method: 50 °C 10 min coupling, Amino Acid: from method, Amino Acid Volume: 2.5 mL, Activator Bottle Position: PositionACT, Activator Volume: 2 mL, Activator Base Position: PositionACTB, Activator Base Volume: 1 mL, Delayed Reagent Time: 0 s, Delayed Reagent Bottle Position: PositionACTB, Delayed Reagent Volume: 0 mL, Manifold Wash Volume: 2 mL
7	Wash	Volume: 4 mL, Drain Time: 10 s
8	Wash	Volume: 4 mL, Drain Time: 5 s

Table S13: Settings for **Single rt Coupling**.

	Cycle Steps	Parameter values
1	Conventional deprotection	Reaction Method: Conventional Deprotection, Deprotection Volume: 4 mL
2	Conventional deprotection	Reaction Method: Conventional Deprotection, Deprotection Volume: 4 mL
3	Wash	Volume: 4 mL, Drain Time: 10 s
4	Wash	Volume: 4 mL, Drain Time: 5 s
5	Wash	Volume: 4 mL, Drain Time: 5 s
6	Wash	Volume: 4 mL, Drain Time: 5 s
7	Conventional coupling	Reaction Method: Standard coupling, Amino Acid: from method, Amino Acid Volume: 2.5 mL, Activator Bottle Position: PositionACT, Activator Volume: 2 mL, Activator Base Position: PositionACTB, Activator Base Volume: 1 mL, Delayed Reagent Time: 0 s, Delayed Reagent Bottle Position: PositionACTB, Delayed Reagent Volume: 0 mL, Manifold Wash Volume: 2 mL
8	Wash	Volume: 4 mL, Drain Time: 10 s
9	Wash	Volume: 4 mL, Drain Time: 5 s

Table S14: Settings for **Double Coupling**.

	Cycle Steps	Parameter values
1	Deprotection	Reaction Method: Standard Deprotection, Deprotection Volume: 4 mL
2	Wash	Volume: 4 mL, Drain Time: 10 s
3	Wash	Volume: 4 mL, Drain Time: 5 s
4	Wash	Volume: 4 mL, Drain Time: 5 s
5	Wash	Volume: 4 mL, Drain Time: 5 s
6	Coupling	Reaction Method: Standard coupling, Amino Acid: from method, Amino Acid Volume: 2.5 mL, Activator Bottle Position: Position ACT, Activator Volume: 2 mL, Activator Base Position: Position ACTB, Activator Base Volume: 1 mL, Delayed Reagent Time: 0 s, Delayed Reagent Bottle Position: PositionACTB, Delayed Reagent Volume: 0 mL, Manifold Wash Volume: 2 mL
7	Wash	Volume: 4 mL, Drain Time: 5 s
8	Coupling	see step 6
9	Wash	Volume: 4 mL, Drain Time: 10 s
10	Wash	Volume: 4 mL, Drain Time: 5 s

Table S15: Settings for **Double Coupling wash**.

	Cycle Steps	Parameter values
1	Deprotection	Reaction Method: Standard Deprotection, Deprotection Volume: 4 mL
2	Wash	Volume: 4 mL, Drain Time: 10 s
3	Wash	Volume: 4 mL, Drain Time: 5 s
4	Wash	Volume: 4 mL, Drain Time: 5 s
5	Wash	Volume: 4 mL, Drain Time: 5 s
6	Wash	Volume: 4 mL, Drain Time: 5 s
7	Wash	Volume: 4 mL, Drain Time: 10 s
8	Coupling	Reaction Method: Standard coupling, Amino Acid: from method, Amino Acid Volume: 2.5 mL, Activator Bottle Position: Position ACT, Activator Volume: 2 mL, Activator Base Position: Position ACTB, Activator Base Volume: 1 mL, Delayed Reagent Time: 0 s, Delayed Reagent Bottle Position: PositionACTB, Delayed Reagent Volume: 0 mL, Manifold Wash Volume: 2 mL
9	Wash	Volume: 4 mL, Drain Time: 10 s
10	Wash	Volume: 4 mL, Drain Time: 10 s
11	Coupling	see step 8
12	Wash	Volume: 4 mL, Drain Time: 10 s
13	Wash	Volume: 4 mL, Drain Time: 5 s
14	Wash	Volume: 4 mL, Drain Time: 5 s
15	Wash	Volume: 4 mL, Drain Time: 5 s

Table S16: Settings for **Final Deprotection**.

	Cycle Steps	Parameter values
1	Deprotection	Reaction Method: Standard Deprotection, Deprotection Volume: 4 mL
2	Wash	Volume: 4 mL, Drain Time: 5 s
3	Wash	Volume: 4 mL, Drain Time: 5 s
4	Wash	Volume: 4 mL, Drain Time: 5 s
5	Wash	Volume: 4 mL, Drain Time: 5 s

Manual Deprotection: The resin was treated with a solution of 20 % piperidine in DMF (5 mL) and the syringe reactor was shaken for 15 min. The solution was discarded and the resin was washed with DMF (5×5 mL), DCM (5×5 mL) and dried under reduced pressure.

Table S17: Settings for the synthesis of **P1**.

	Step	Used Cycles
**1**	Resin swelling	Resin swelling
**2**	G	Double Coupling wash
**3**	G	Double Coupling wash
**4**	F	Single Coupling
**5**	Pra/allylGly	Single Coupling
**6**	F	Single Coupling
**7**	G	Double Coupling wash
**8**	Final deprotection	Final Deprotection

Table S18: Settings for the synthesis of **P2**.

	Step	Used Cycles (optimized conditions)
**1**	Resin swelling	Resin swelling
**2**	E	Single Coupling
**3**	L	Single Coupling
**4**	R	Double Coupling wash
**5**	Q	Single Coupling
**6**	A	Single Coupling
**7**	I	Single Coupling
**8**	E	Single Coupling
**9**	A	Single Coupling
**10**	K	Single Coupling
**11**	Pra/allylGly	Single Coupling
**12**	P	Single Coupling
**13**	Boc-Gly	Double Coupling wash

Table S19: Settings for the synthesis of **P3**.

	Step	Used Cycles (optimized conditions)
**1**	Resin swelling	Resin swelling
**2**	A	Single Coupling
**3**	R	Double Coupling
**4**	Y	Single Coupling
**5**	Q	Single Coupling
**6**	D	Single Coupling
**7**	G	Double Coupling wash
**8**	Pra/allylGly	Single Coupling
**9**	A	Single Coupling
**10**	H	Single 50 °C Coupling
**11**	H	Single 50 °C Coupling
**12**	G	Double Coupling wash
**13**	I	Single Coupling
**14**	I	Single Coupling
**15**	I	Single Coupling
**16**	P	Single Coupling
**17**	K	Single Coupling
**18**	V	Single Coupling
**19**	W	Single Coupling
**20**	G	Double Coupling wash
**21**	Final deprotection	Final Deprotection

Table S20: Settings for the synthesis of **P4**.

	Step	Used Cycles (optimized conditions)
**1**	Resin swelling	Resin swelling
**2**	R	Double Coupling
**3**	H	Single 50 °C Coupling
**4**	P	Single Coupling
**5**	K	Single Coupling
**6**	K	Single Coupling
**7**	V	Single Coupling
**8**	G	Double Coupling
**9**	Pra/allylGly	Single Coupling
**10**	T	Single Coupling
**11**	S	Single Coupling
**12**	P	Single Coupling
**13**	A	Single Coupling
**14**	S	Single Coupling
**15**	M	Single Coupling
**16**	R	Double Coupling
**17**	A	Single Coupling
**18**	A	Single Coupling
**19**	K	Single Coupling
**20**	T	Double Coupling
**21**	A	Single Coupling
**22**	L	Single Coupling
**23**	Q	Single Coupling
**24**	K	Double Coupling
**25**	R	Double Coupling
**26**	P	Single Coupling
**27**	A	Double Coupling
**28**	K	Single Coupling
**29**	Manual deprotection	

Table S21: Settings for the synthesis of **P5**.

	Step	Used Cycles (optimized conditions)
**1**	Resin swelling	Resin swelling
**2**	H	Single 50 °C Coupling
**3**	T	Single Coupling
**4**	R	Double Coupling
**5**	Q	Single Coupling
**6**	H	Single 50 °C Coupling
**7**	K	Single Coupling
**8**	Q	Single Coupling
**9**	L	Single Coupling
**10**	N	Single Coupling
**11**	S	Single Coupling
**12**	S	Single Coupling
**13**	Q	Single Coupling
**14**	S	Single 50 °C Coupling
**15**	F	Single Coupling
**16**	S	Single Coupling
**17**	K	Single Coupling
**18**	G	Double Coupling
**19**	C	Single Coupling
**20**	Q	Single Coupling
**21**	P	Single Coupling
**22**	C	Single Coupling
**23**	K	Single Coupling
**24**	Y	Single Coupling
**25**	P	Single Coupling
**26**	Pra/allylGly	Single Coupling
**27**	Manual deprotection	

Table S22: Settings for the synthesis of **P6**.

	Step	Used Cycles (optimized conditions)
**1**	Resin swelling	Resin swelling
**2**	G	Double Coupling wash
**3**	S	Single Coupling
**4**	P	Single Coupling
**5**	R	Double Coupling
**6**	E	Single Coupling
**7**	Pra/allylGly	Single Coupling
**8**	Q	Single Coupling
**9**	S	Single Coupling
**10**	A	Single Coupling
**11**	N	Single Coupling
**12**	T	Single Coupling
**13**	I	Single Coupling
**14**	H	Single 50 °C Coupling
**15**	N	Single Coupling
**16**	F	Single Coupling
**17**	Y	Single Coupling
**18**	Y	Single Coupling
**19**	V	Single Coupling
**20**	R	Double Coupling
**21**	G	Double Coupling wash
**22**	S	Single Coupling
**23**	S	Single Coupling
**24**	R	Double Coupling
**25**	S	Single Coupling
**26**	Nle	Single Coupling
**27**	R	Double Coupling
**28**	K	Single Coupling
**29**	E	Single Coupling
**30**	W	Single Coupling
**31**	G	Double Coupling wash
**32**	P	Single Coupling
**33**	P	Single Coupling
**34**	L	Single Coupling
**35**	K	Single Coupling
**36**	Final deprotection	Final Deprotection

Table S23: Settings for the synthesis of **P7**.

	Step	Used Cycles (optimized conditions)
**1**	Resin swelling	Resin swelling
**2**	G	Double Coupling wash
**3**	Y	Single Coupling
**4**	K	Single Coupling
**5**	E	Single Coupling
**6**	V	Single Coupling
**7**	Y	Single Coupling
**8**	P	Single Coupling
**9**	V	Single Coupling
**10**	P	Single Coupling
**11**	I	Single Coupling
**12**	L	Single Coupling
**13**	G	Double Coupling wash
**14**	R	Double Coupling
**15**	K	Single Coupling
**16**	G	Double Coupling wash
**17**	E	Single Coupling
**18**	S	Single Coupling
**19**	N	Single Coupling
**20**	E	Single Coupling
**21**	A	Double Coupling wash
**22**	N	Single Coupling
**23**	W	Single Coupling
**24**	W	Single Coupling
**25**	Q	Single Coupling
**26**	E	Single Coupling
**27**	E	Single Coupling
**28**	P	Single Coupling
**29**	K	Single Coupling
**30**	D	Single Coupling
**31**	R	Double Coupling
**32**	I	Double Coupling
**33**	R	Double Coupling
**34**	L	Double Coupling
**35**	I	Double Coupling
**36**	E	Double Coupling
**37**	G	Double Coupling wash
**38**	K	Double Coupling
**39**	K	Double Coupling
**40**	F	Double Coupling
**41**	P	Double Coupling
**42**	L	Double Coupling
**43**	D	Double Coupling
**44**	E	Double Coupling
**45**	E	Double Coupling
**46**	Pra/allylGly	Double Coupling
**47**	N	Double Coupling
**48**	G	Double Coupling wash
**49**	N	Double Coupling
**50**	F	Double Coupling
**51**	D	Double Coupling
**52**	F	Double Coupling
**53**	L	Double Coupling
**54**	A	Double Coupling
**55**	R	Double Coupling
**56**	V	Double Coupling
**57**	Y	Double Coupling
**58**	E	Double Coupling
**59**	A	Double Coupling
**60**	Final deprotection	Final Deprotection

Table S24: Settings for the synthesis of **P8**.

	Step	Used Cycles (optimized conditions)
**1**	Resin swelling	Resin swelling
**2**	Pra/allylGly	Single Coupling
**3**	A	Double Coupling
**4**	A	Double Coupling
**5**	A	Double Coupling
**6**	A	Double Coupling
**7**	A	Double Coupling
**8**	A	Double Coupling
**9**	Final deprotection	Final Deprotection

Table S25: Settings for the synthesis of **P9**.

	Step	Used Cycles (optimized conditions)
**1**	Resin swelling	Resin swelling
**2**	Q	Single Coupling
**3**	Q	Single Coupling
**4**	R	Double Coupling
**5**	K	Single Coupling
**6**	R	Double Coupling
**7**	K	Single Coupling
**8**	I	Single Coupling
**9**	W	Single Coupling
**10**	S	Single Coupling
**11**	I	Single Coupling
**12**	L	Single Coupling
**13**	A	Single Coupling
**14**	P	Single Coupling
**15**	L	Single Coupling
**16**	G	Double Coupling wash
**17**	T	Single Coupling
**18**	T	Single Coupling
**19**	L	Single Coupling
**20**	V	Single Coupling
**21**	K	Single Coupling
**22**	L	Single Coupling
**23**	Pra/allylGly	Single Coupling
**24**	A	Single Coupling
**25**	G	Double Coupling wash
**26**	I	Single Coupling
**27**	G	Double Coupling wash
**28**	Final deprotection	Final Deprotection

Table S26: Settings for the synthesis of **P10**.

	Step	Used Cycles (optimized conditions)
**1**	Resin swelling	Resin swelling
**2**	P	Single Coupling
**3**	A	Single Coupling
**4**	G	Double Coupling wash
**5**	V	Single Coupling
**6**	G	Double Coupling wash
**7**	I	Single Coupling
**8**	A	Single Coupling
**9**	T	Single Coupling
**10**	Q	Single Coupling
**11**	P	Single Coupling
**12**	F	Single Coupling
**13**	T	Single Coupling
**14**	H	Single 50 °C Coupling
**15**	F	Single Coupling
**16**	K	Single Coupling
**17**	N	Single Coupling
**18**	F	Single Coupling
**19**	D	Single Coupling
**20**	Q	Single Coupling
**21**	T	Single Coupling
**22**	Y	Single Coupling
**23**	T	Single Coupling
**24**	G	Double Coupling wash
**25**	L	Single Coupling
**26**	Pra/allylGly	Single Coupling
**27**	C	Double Coupling
**28**	T	Single Coupling
**29**	S	Single Coupling
**30**	L	Single Coupling
**31**	N	Single Coupling
**32**	G	Double Coupling wash
**33**	C	Single Coupling
**34**	Final deprotection	Final Deprotection

Table S27: Settings for the synthesis of **P11**.

	Step	Used Cycles (optimized conditions)
**1**	Resin swelling	Resin swelling
**2**	S	Single Coupling
**3**	P	Single Coupling
**4**	Pra/allylGly	Single Coupling
**5**	T	Single Coupling
**6**	P	Single Coupling
**7**	pS	Single rt Coupling
**8**	Y	Single rt Coupling
**9**	Final deprotection	Final Deprotection

2.4 Catch-release experiment and fluorescence microscopy

A 5 mL syringe reactor equipped with a PE frit was charged with 20 mg *Sephadex* G-100 powder. FAM labelled peptide (**FAM-P5=** and **FAM-P5–B(OH)$_2$**) was prepared as a 1 mL solution in dry DMSO in presence of 250 µM DIPEA. The peptide concentration was 25 µM. In parallel, a sample without peptide was prepared. The mixture was added to the Sephadex powder and the syringe reactor was shaken for 20 h. The solution was discarded, and the dextran beads were washed with dry DMSO (5 x 1 mL), whereby the plunger was removed and DMSO was eluted through the beads. The picture shown in Figure 6B was taken under a UV lamp (λ_{ex} = 365 nm). The beads were suspended in 1 mL dry DMSO for analysis by fluorescence microscopy (Figure S100, S101). For release of the peptide boronic acid, the solution was discarded and 1 mL purified water was added. The mixture was shaken for 1 h. The solution was discarded and the beads were photographed under a UV lamp (λ_{ex} = 365 nm) as shown in Figure 6C. The beads were washed with 1 mL of buffer A and then suspended in 1 mL dry DMSO for analysis by fluorescence microscopy (Figure S102).

Glass slides (*epredia* Microscope Slides, ground 45° frosted) and cover slides (VWR 20x20 mm cover borosilicate glass) were washed with isopropanol and dried in a stream of nitrogen. 10 µL of each dextran bead/peptide suspension was dropcasted onto the glass slide and covered immediately with a cover slide to obtain a homogenously thin layer in DMSO.

The samples were immediately analysed using a *Zeiss Axio Imager Z2m* equipped with the Axiocam 503 color camera using the *Zen 3.1 blue edition* software. Images were captured using a *Zeiss Epiplan-NEOFLUAR 10x/0.25 HD DIC* objective. Bright-field images were captured in transmission light modus with automatic exposure time. A Colibri 7 LED light used was used for fluorescence illumination. Fluorescence images were captured in reflective light modus using an excitation wavelength of λ= 475 nm for FAM excitation and an exposure time of 200 ms for all images to guarantee comparability of fluorescence intensities. All images have a size of 1146 x 864 pixel and a pixelsize of 0.82 µm/pixel.

All images were post-processed using *ImageJ 1.53t* to adjust contrast for better visibility. The option *Image --> Adjust --> Brightness/Contrast* was used. The pixel values for all bright-field images (RGB) were adjusted in a range of 90 to 180. Deviating from this, the pixel values of the bright-field image of pure dextran beads (Figure S100 A) were adjusted in a range of 70 to 170. Equally, all fluorescence images (RGB image) were adjusted to a range of pixel values between 8 and 110.

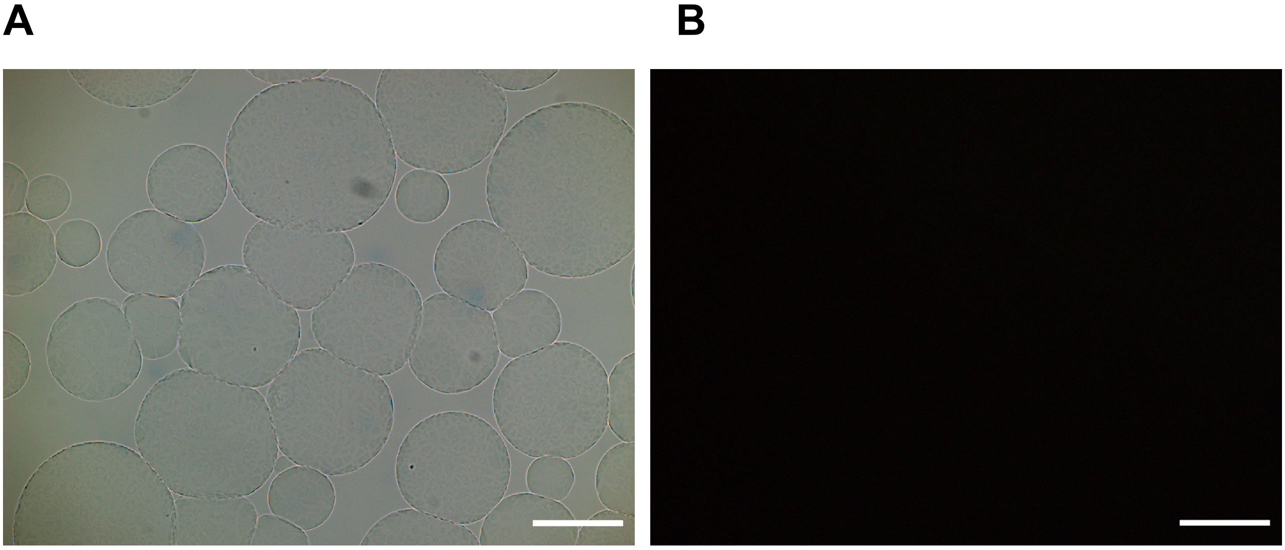

Figure S100: Bright-field image (A) and fluorescence image (B) of pure Sephadex beads in DMSO. Excitation wavelength λ = 475 nm. Scale bar = 200 µm.

A **B**

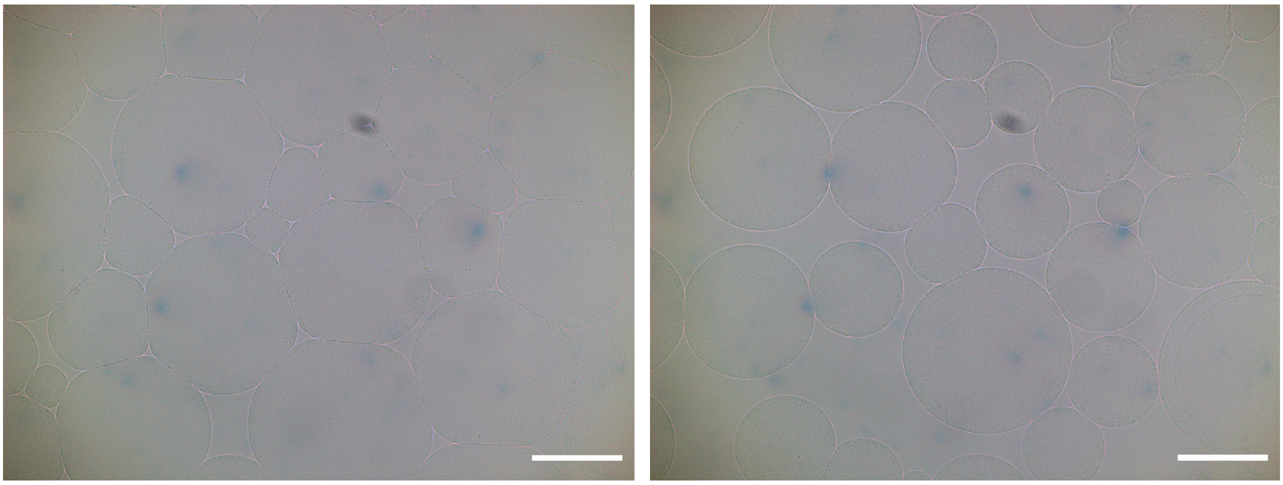

Figure S101: Bright-field images of Sephadex beads in DMSO after incubation with **FAM-P5= (A)** and incubation of **FAM-P5–B(OH)$_2$** (B) in DMSO. Scale bar = 200 µm.

A **B**

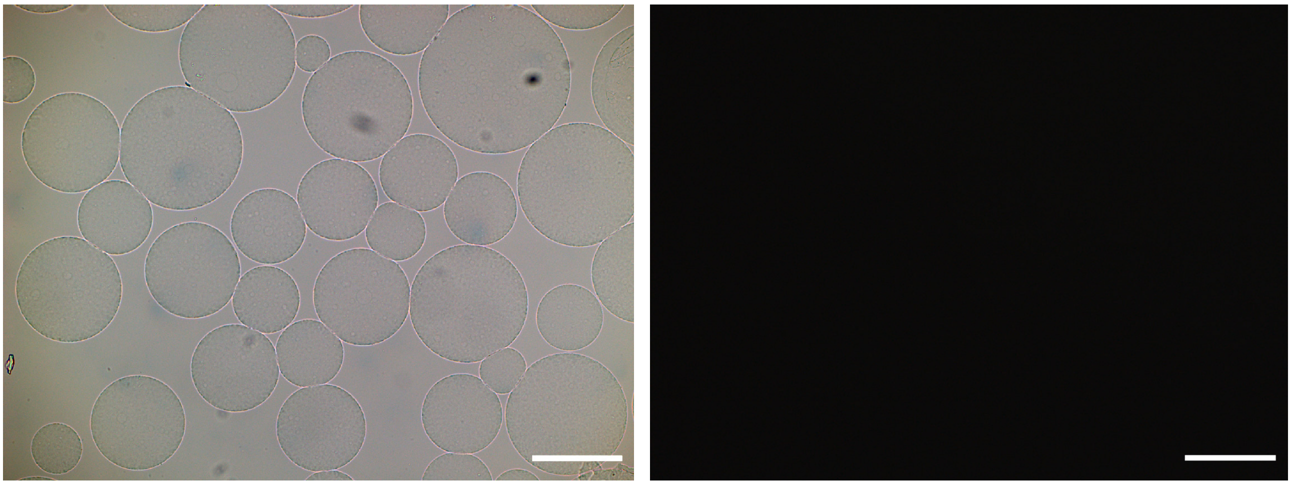

Figure S102: Bright-field image (A) and fluorescence image (B) of Sephadex beads in DMSO after release of **FAM-P5–B(OH)$_2$**. Excitation wavelength λ = 475 nm. Scale bar = 200 µm.

2.5 NMR spectroscopy

Nuclear magnetic resonance (NMR) spectroscopy was performed on a Bruker Avance III 400 spectrometer at 400 MHz. Samples were dissolved in deuterated DMSO and transferred to 5 mm diameter tubes. In preparation for the NMR measurement of a purified peptide, the sample was dissolved in a mixture of deuterated MeCN (200 µL) and deuterated water (D_2O) (800 µL). After incubation for one hour, the solution was lyophilised. This step was repeated once to ensure backbone hydrogen-deuterium exchange. The measured ^{1}H-NMR spectra were referenced to the residual protons of the deuterated solvent (δ = 2.50 ppm for DMSO-d_6).

Spectra processing and analysis was performed using *MestReNova 14.3.3*. Signal multiplicities were abbreviated with s (singlet), d (doublet), t (triplet), dt (doublet of triplets) and m (multiplet).

2.6 Mass spectrometry

Electrospray ionisation (ESI) was performed on an ion cyclotron resonance Bruker microTOF-Q II.

Matrix-assisted laser desorption ionisation time-of-flight mass spectrometry (MALDI-TOF MS) was performed on a Bruker Autoflex Speed. 1 µl of the matrix 2,5-dihydroxybenzoic acid (20 mg/mL in 7:3 MeCN/water + 0.1% TFA) was mixed with 1 µl of the sample, pipetted onto the target plate and air dried.

3 ESI-MS

Pure **P4–B(OH)$_2$**

Calculated mass: [M-2H$_2$O] 2920.7, [M-H$_2$O] 2938.7, [M] 2956.7.

Display Report

Analysis Info

Analysis Name	E:\Data4\HEIKO\11_2023\Marius_Werner\JB_052_21.0.d	Acquisition Date	11/24/2023 12:43:57 PM
Method	2_2_HR_hohe_Massen_300-3000_pos_20-07-2023.m	Operator	Heiko Rudy
Sample Name	C126H226BN45O34S = 2956,715 u	Instrument	micrOTOF-Q 228888.10254
Comment			

Acquisition Parameter

Source Type	ESI	Ion Polarity	Positive	Set Nebulizer	0.3 Bar
Focus	Not active	Set Capillary	4500 V	Set Dry Heater	200 °C
Scan Begin	300 m/z	Set End Plate Offset	-500 V	Set Dry Gas	4.0 l/min
Scan End	3000 m/z	Set Collision Cell RF	700.0 Vpp	Set Divert Valve	Source

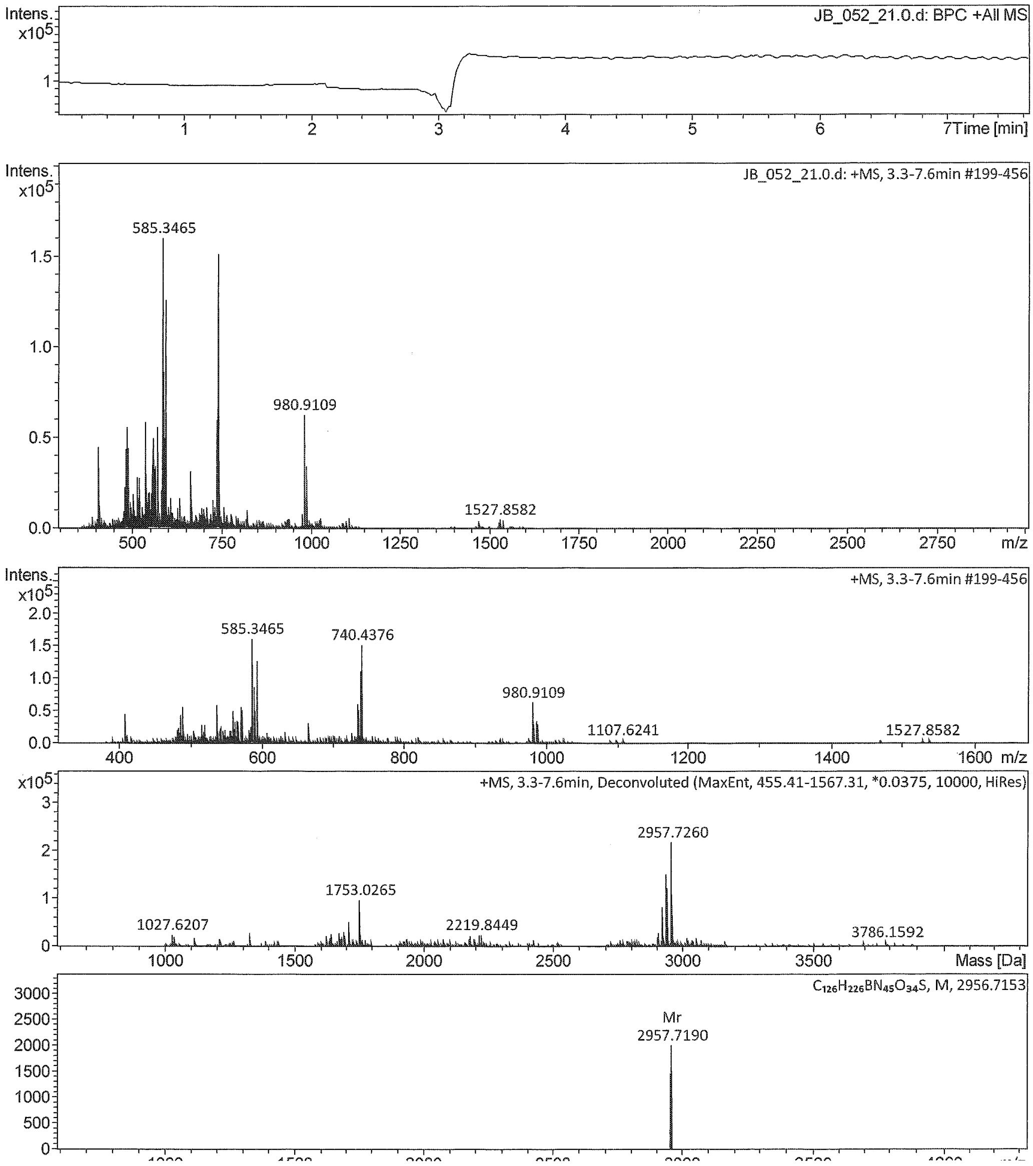

Display Report

Analysis Info

Analysis Name	E:\Data4\HEIKO\11_2023\Marius_Werner\JB_052_21.0.d	Acquisition Date	11/24/2023 12:43:57 PM
Method	2_2_HR_hohe_Massen_300-3000_pos_20-07-2023.m	Operator	Heiko Rudy
Sample Name	C126H226BN45O34S = 2956,715 u	Instrument	micrOTOF-Q 228888.10254
Comment			

Acquisition Parameter

Source Type	ESI	Ion Polarity	Positive	Set Nebulizer	0.3 Bar
Focus	Not active	Set Capillary	4500 V	Set Dry Heater	200 °C
Scan Begin	300 m/z	Set End Plate Offset	-500 V	Set Dry Gas	4.0 l/min
Scan End	3000 m/z	Set Collision Cell RF	700.0 Vpp	Set Divert Valve	Source

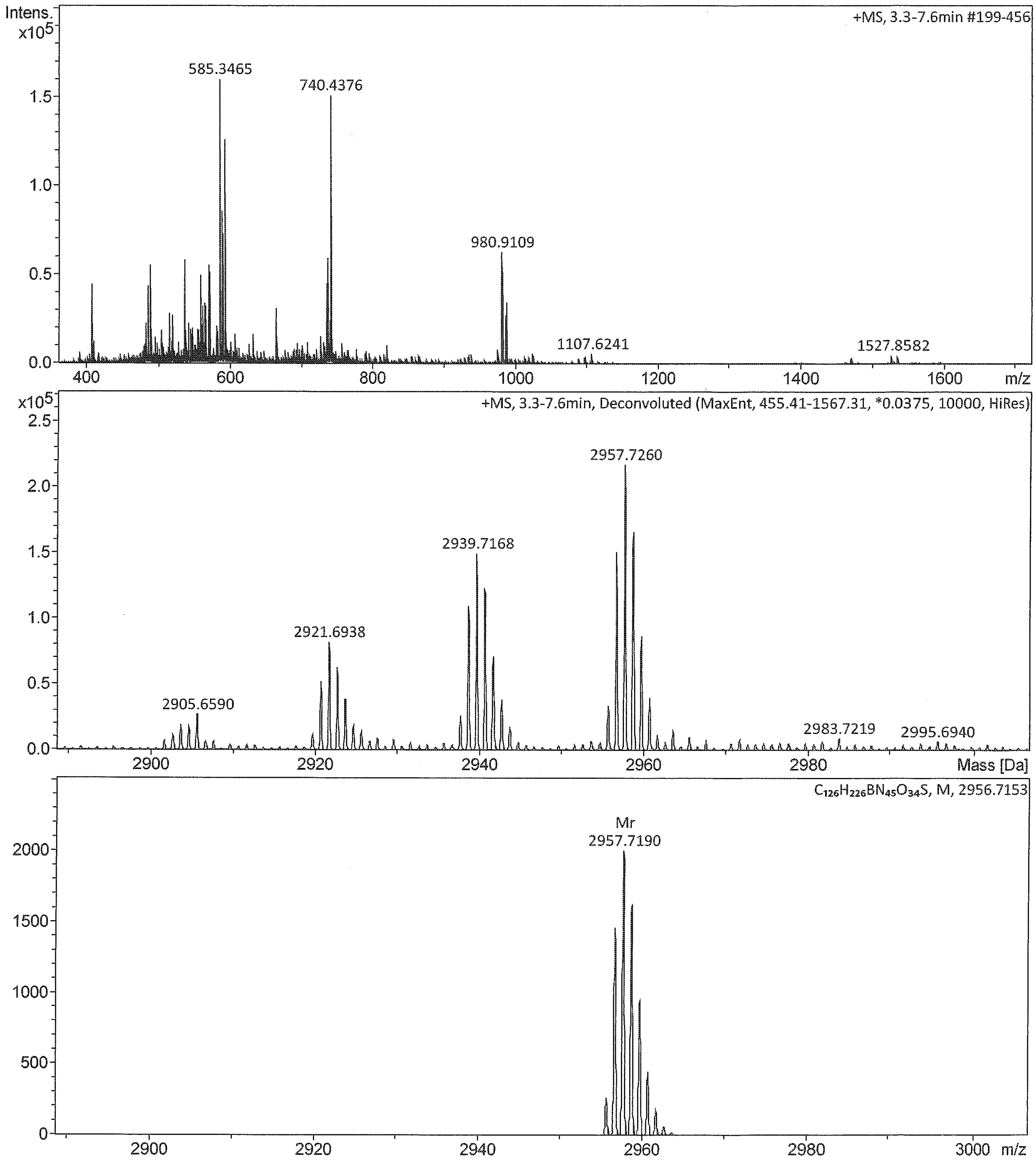

Bruker Compass DataAnalysis 4.2 printed: 11/24/2023 12:59:02 PM by: Heiko Rudy Page 1 of 1

Display Report

Analysis Info

Analysis Name	E:\Data4\HEIKO\11_2023\Marius_Werner\JB_052_21.0.d	Acquisition Date	11/24/2023 12:43:57 PM
Method	2_2_HR_hohe_Massen_300-3000_pos_20-07-2023.m	Operator	Heiko Rudy
Sample Name	C126H226BN45O34S = 2956,715 u	Instrument	micrOTOF-Q 228888.10254
Comment			

Acquisition Parameter

Source Type	ESI	Ion Polarity	Positive	Set Nebulizer	0.3 Bar
Focus	Not active	Set Capillary	4500 V	Set Dry Heater	200 °C
Scan Begin	300 m/z	Set End Plate Offset	-500 V	Set Dry Gas	4.0 l/min
Scan End	3000 m/z	Set Collision Cell RF	700.0 Vpp	Set Divert Valve	Source

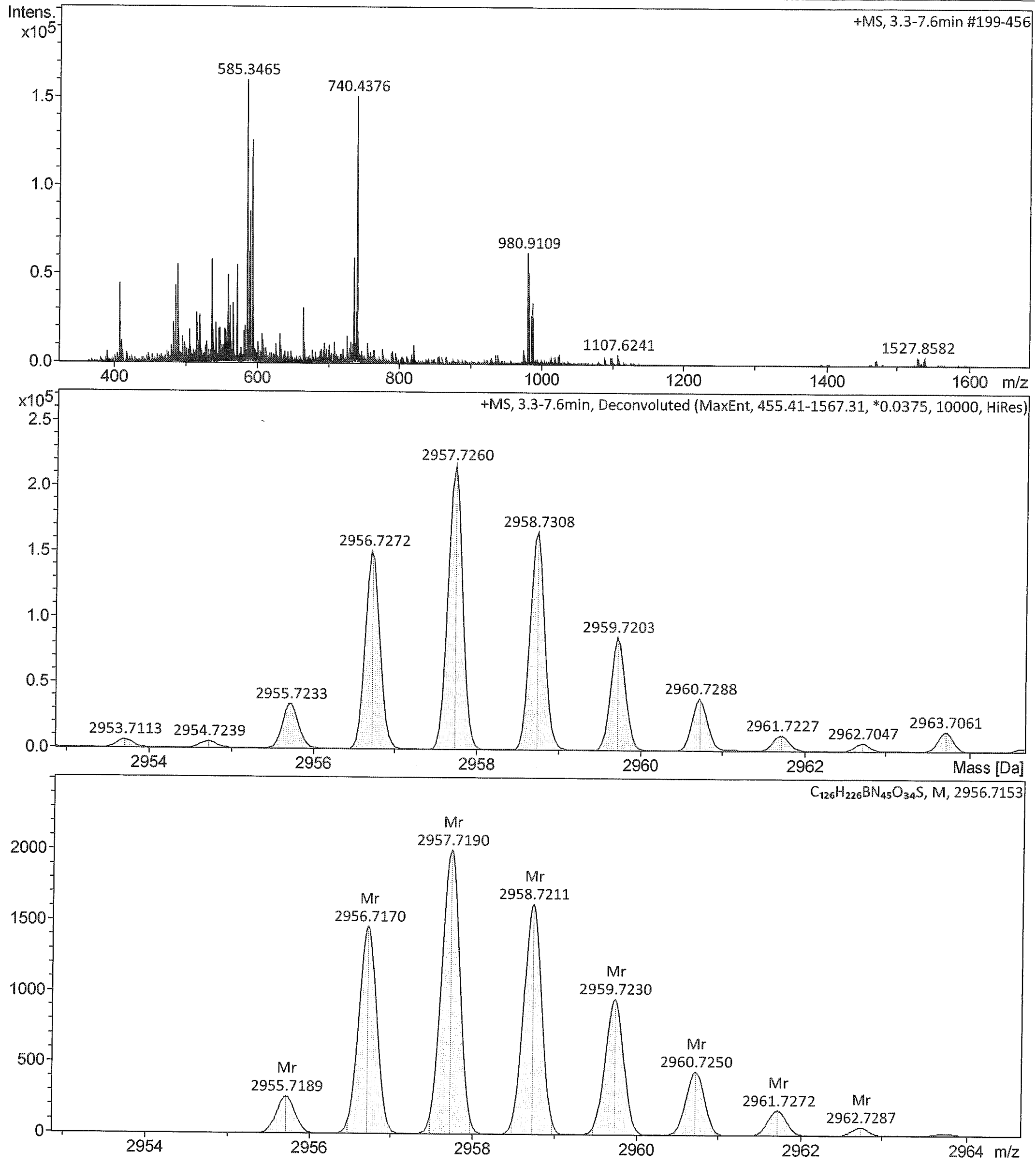

Pure **P6–B(OH)$_2$**

Calculated mass: [M-2H_2O] 3965.0, [M-H_2O] 3983.1.

Display Report

Analysis Info

Analysis Name E:\Data4\HEIKO\10_2023\Marius_Werner\JB_111_F_2.d
Method 2_2_HR_hohe_Massen_300-3000_pos_20-07-2023.m
Sample Name [redacted]
Comment

Acquisition Date 10/26/2023 12:10:25 PM

Operator Heiko Rudy
Instrument micrOTOF-Q 228888.10254

Acquisition Parameter

Source Type	ESI	Ion Polarity	Positive	Set Nebulizer	0.3 Bar
Focus	Not active	Set Capillary	4500 V	Set Dry Heater	200 °C
Scan Begin	300 m/z	Set End Plate Offset	-500 V	Set Dry Gas	4.0 l/min
Scan End	3000 m/z	Set Collision Cell RF	700.0 Vpp	Set Divert Valve	Source

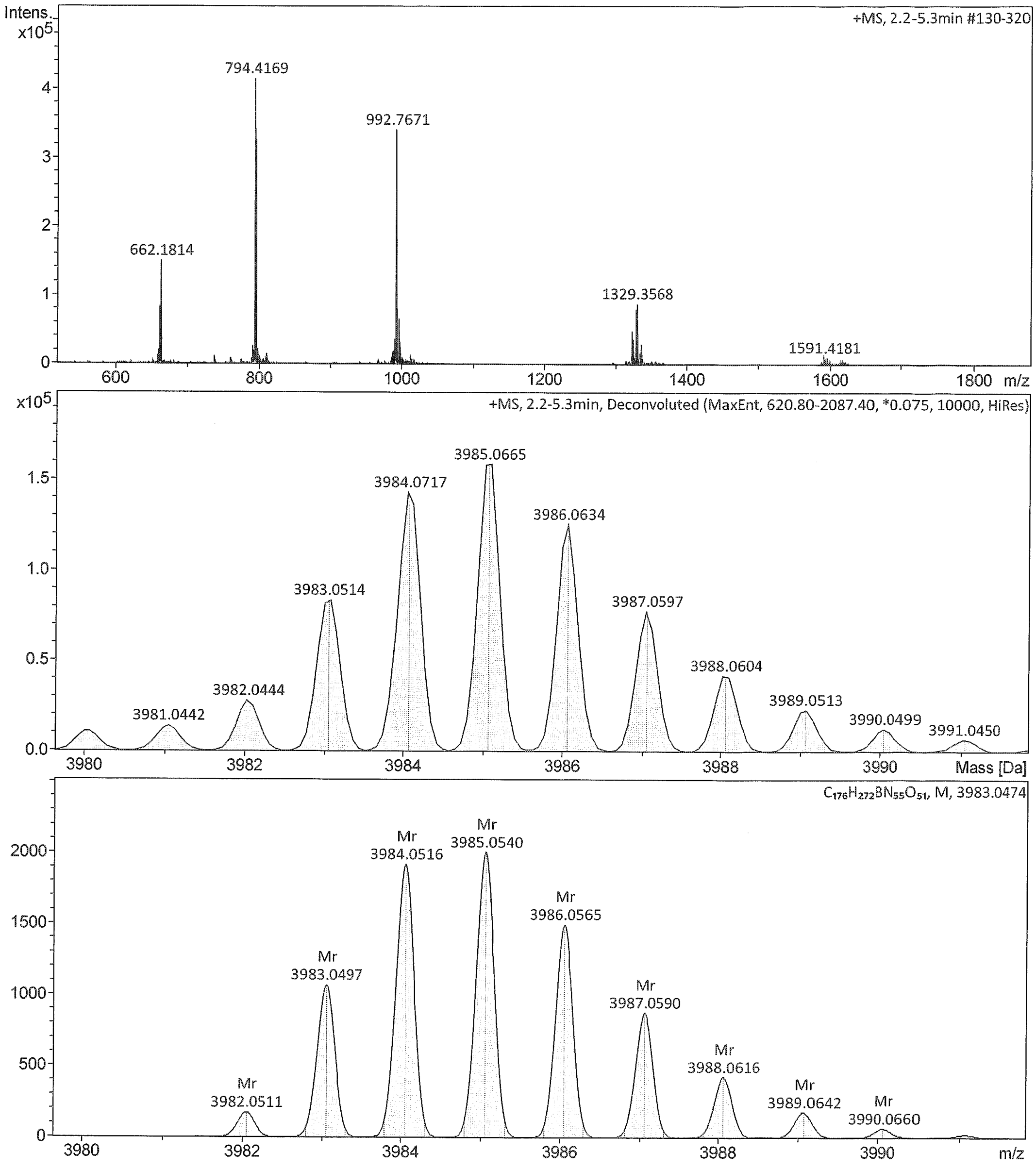

Bruker Compass DataAnalysis 4.2 printed: 10/26/2023 12:26:15 PM by: Heiko Rudy Page 1 of 1

Display Report

Analysis Info

Analysis Name	E:\Data4\HEIKO\10_2023\Marius_Werner\JB_111_F_2.d	Acquisition Date	10/26/2023 12:10:25 PM
Method	2_2_HR_hohe_Massen_300-3000_pos_20-07-2023.m	Operator	Heiko Rudy
Sample Name	[redacted]	Instrument	micrOTOF-Q 228888.10254
Comment			

Acquisition Parameter

Source Type	ESI	Ion Polarity	Positive	Set Nebulizer	0.3 Bar
Focus	Not active	Set Capillary	4500 V	Set Dry Heater	200 °C
Scan Begin	300 m/z	Set End Plate Offset	-500 V	Set Dry Gas	4.0 l/min
Scan End	3000 m/z	Set Collision Cell RF	700.0 Vpp	Set Divert Valve	Source

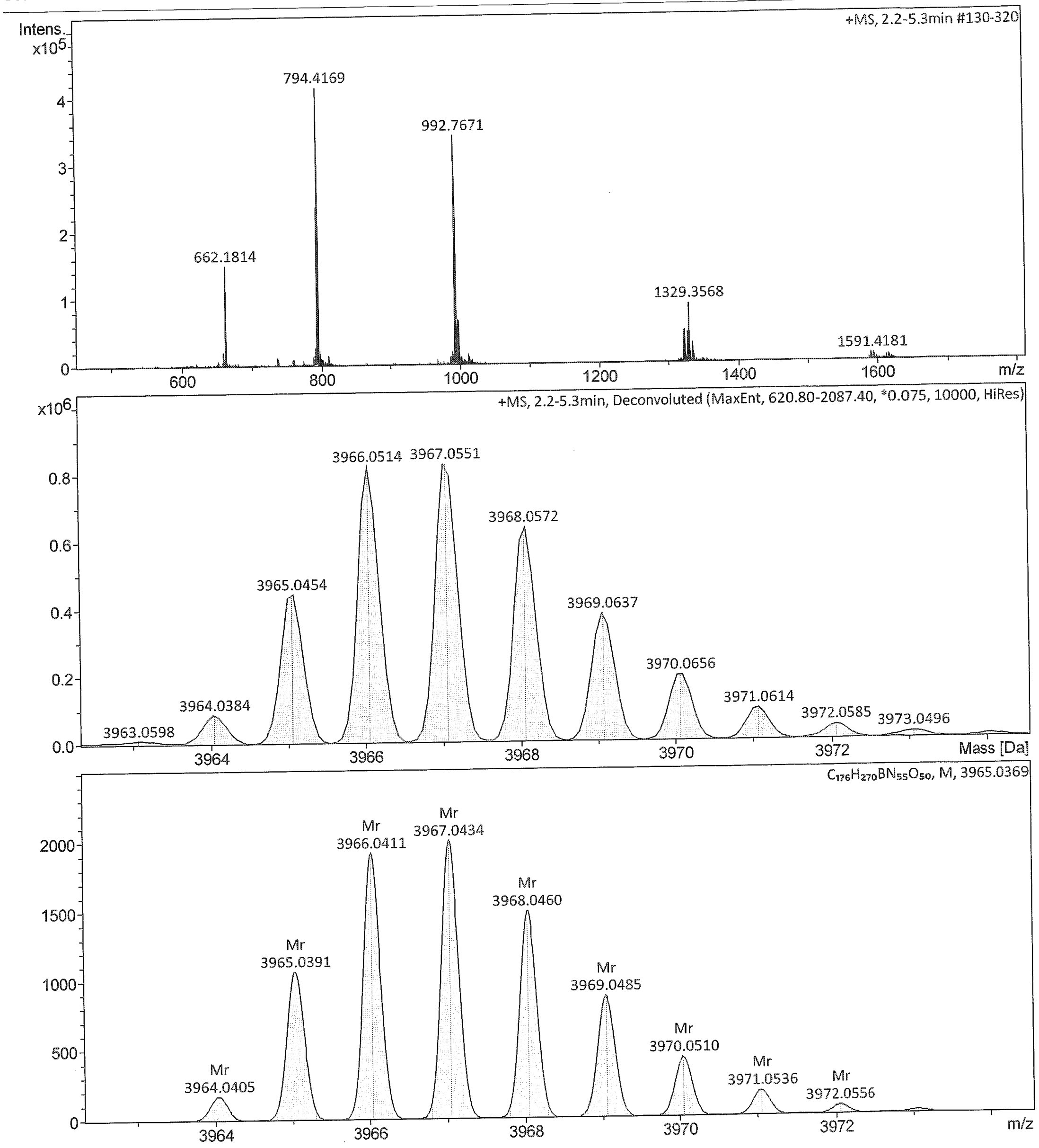

Bruker Compass DataAnalysis 4.2 printed: 10/26/2023 12:28:35 PM by: Heiko Rudy Page 1 of 1

Pure **P7–B(OH)₂**

Calculated mass: [M-2H_2O] 6844.5.

Display Report

Analysis Info

Analysis Name	E:\Data4\HEIKO\02_2024\Marius_Werner\SH3_02_pure.d	Acquisition Date	2/9/2024 10:53:38 AM
Method	2_2_HR_hohe_Massen_300-3000_pos_20-07-2023.m	Operator	Heiko Rudy
Sample Name	C312H472BN83O93 = 6880,48 u	Instrument	micrOTOF-Q 228888.10254
Comment			

Acquisition Parameter

Source Type	ESI	Ion Polarity	Positive	Set Nebulizer	0.3 Bar
Focus	Not active	Set Capillary	4500 V	Set Dry Heater	200 °C
Scan Begin	300 m/z	Set End Plate Offset	-500 V	Set Dry Gas	4.0 l/min
Scan End	3000 m/z	Set Collision Cell RF	700.0 Vpp	Set Divert Valve	Source

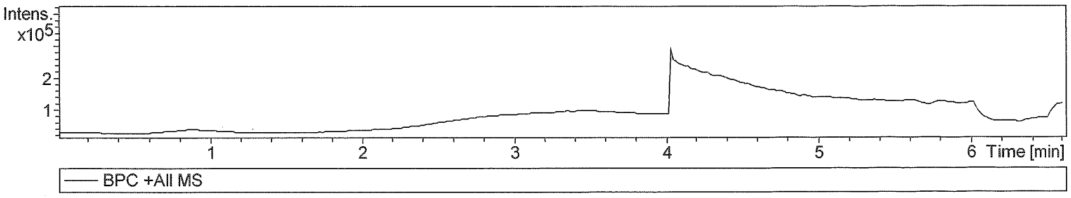

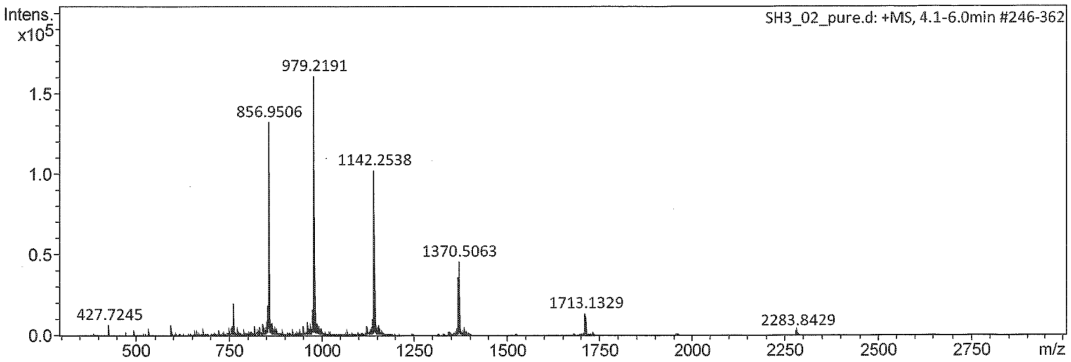

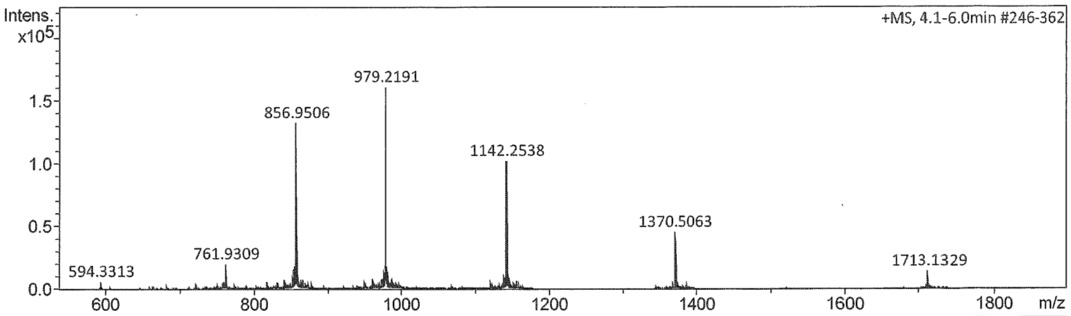

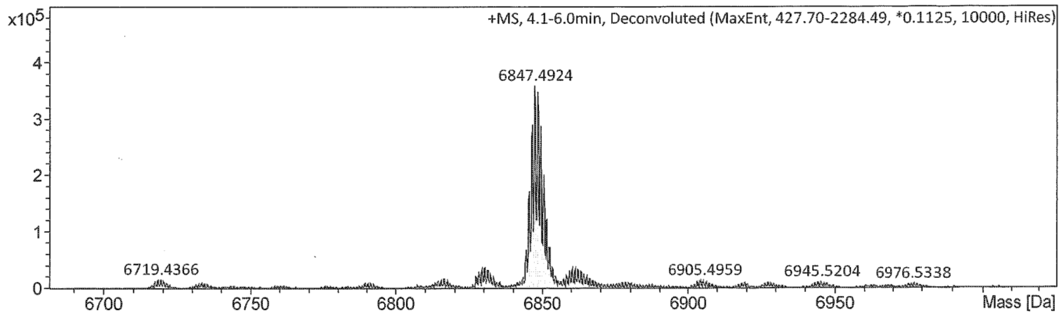

Bruker Compass DataAnalysis 4.2 printed: 2/9/2024 11:06:09 AM by: Heiko Rudy Page 1 of 1

Display Report

Analysis Info

Analysis Name	E:\Data4\HEIKO\02_2024\Marius_Werner\SH3_02_pure.d	Acquisition Date	2/9/2024 10:53:38 AM
Method	2_2_HR_hohe_Massen_300-3000_pos_20-07-2023.m	Operator	Heiko Rudy
Sample Name	C312H472BN83O93 = 6880,48 u	Instrument	micrOTOF-Q 228888.10254
Comment			

Acquisition Parameter

Source Type	ESI	Ion Polarity	Positive	Set Nebulizer	0.3 Bar
Focus	Not active	Set Capillary	4500 V	Set Dry Heater	200 °C
Scan Begin	300 m/z	Set End Plate Offset	-500 V	Set Dry Gas	4.0 l/min
Scan End	3000 m/z	Set Collision Cell RF	700.0 Vpp	Set Divert Valve	Source

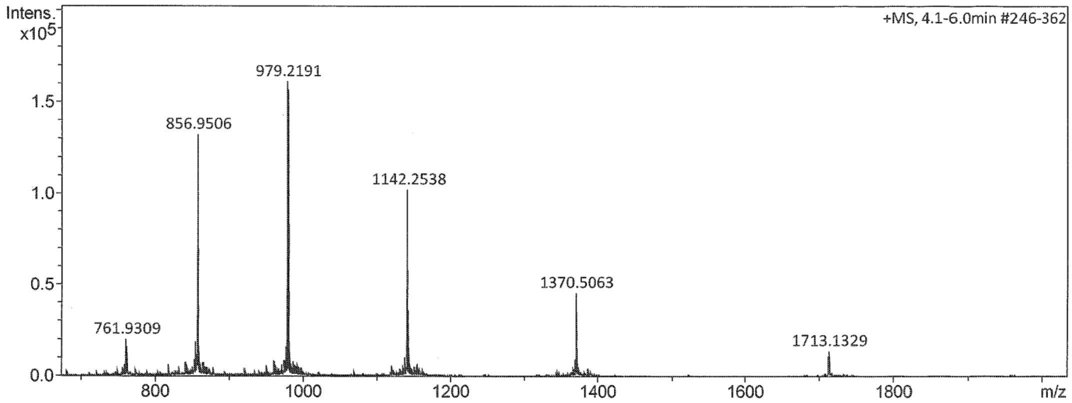

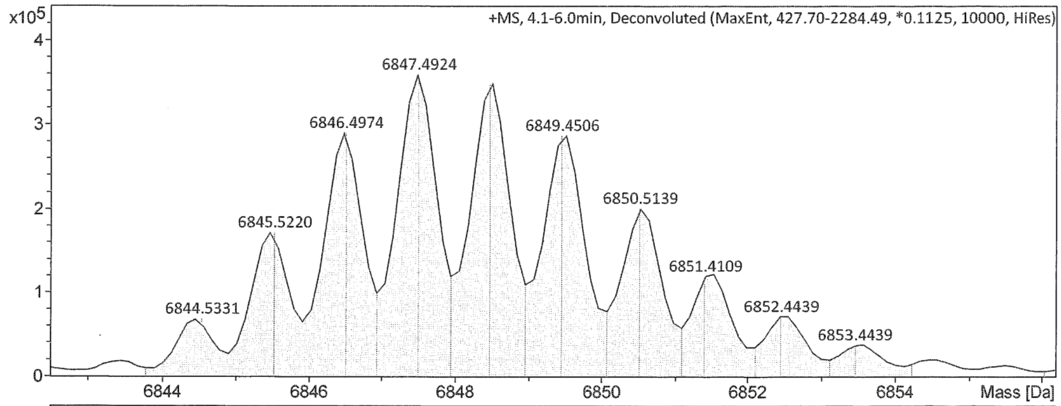

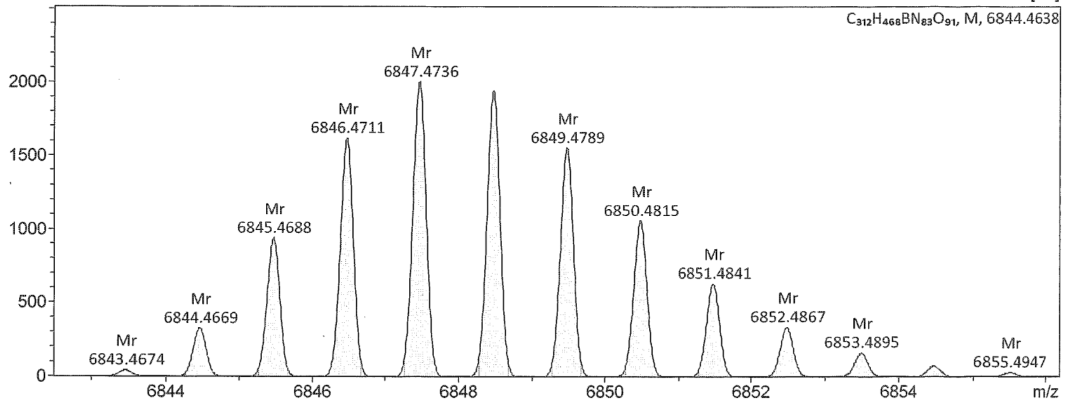

Bruker Compass DataAnalysis 4.2 printed: 2/9/2024 11:07:53 AM by: Heiko Rudy Page 1 of 1

Pure **P4=B(OH)₂** prepared by $[Ru(CO)(Cl)H(PPh_3)_3]$-catalyzed hydroboration (Reaction Conditions A).

Calculated mass: $[M-2H_2O]$ 2918.7, $[M-H_2O]$ 2936.7, [M] 2954.7.

Display Report

Analysis Info

Analysis Name	E:\Data4\HEIKO\12_2023\Marius_Werner\JB_044_F_1.d	Acquisition Date	12/18/2023 9:37:01 AM
Method	2_2_HR_hohe_Massen_300-3000_pos_20-07-2023.m	Operator	Heiko Rudy
Sample Name	C126H224BN45O34S = 2954,700 u	Instrument	micrOTOF-Q 228888.10254
Comment			

Acquisition Parameter

Source Type	ESI	Ion Polarity	Positive	Set Nebulizer	0.3 Bar
Focus	Not active	Set Capillary	4500 V	Set Dry Heater	200 °C
Scan Begin	300 m/z	Set End Plate Offset	-500 V	Set Dry Gas	4.0 l/min
Scan End	3000 m/z	Set Collision Cell RF	700.0 Vpp	Set Divert Valve	Source

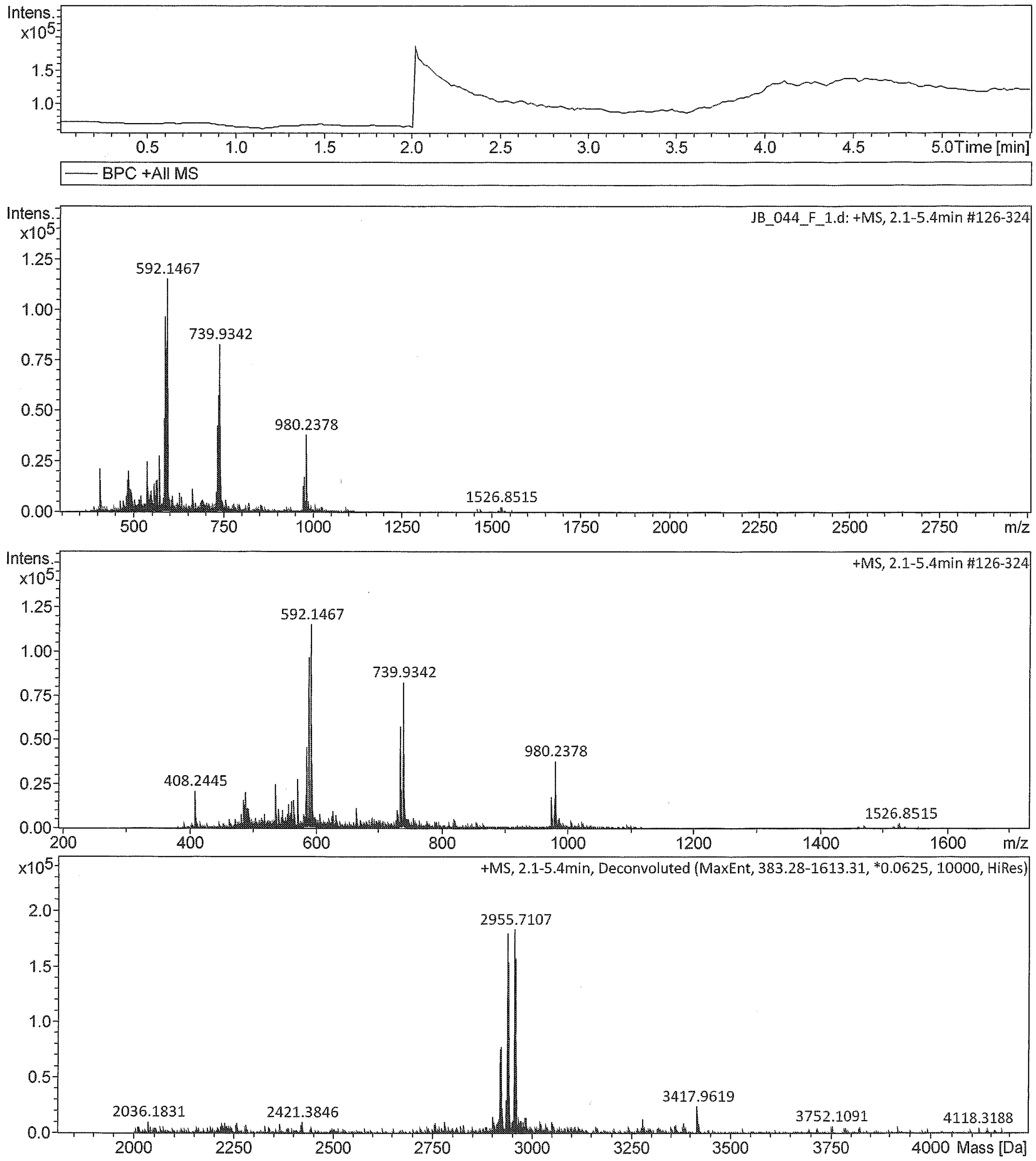

Display Report

Analysis Info

Analysis Name	E:\Data4\HEIKO\12_2023\Marius_Werner\JB_044_F_1.d	Acquisition Date	12/18/2023 9:37:01 AM
Method	2_2_HR_hohe_Massen_300-3000_pos_20-07-2023.m	Operator	Heiko Rudy
Sample Name	C126H224BN45O34S = 2954,700 u	Instrument	micrOTOF-Q 228888.10254
Comment			

Acquisition Parameter

Source Type	ESI	Ion Polarity	Positive	Set Nebulizer	0.3 Bar
Focus	Not active	Set Capillary	4500 V	Set Dry Heater	200 °C
Scan Begin	300 m/z	Set End Plate Offset	-500 V	Set Dry Gas	4.0 l/min
Scan End	3000 m/z	Set Collision Cell RF	700.0 Vpp	Set Divert Valve	Source

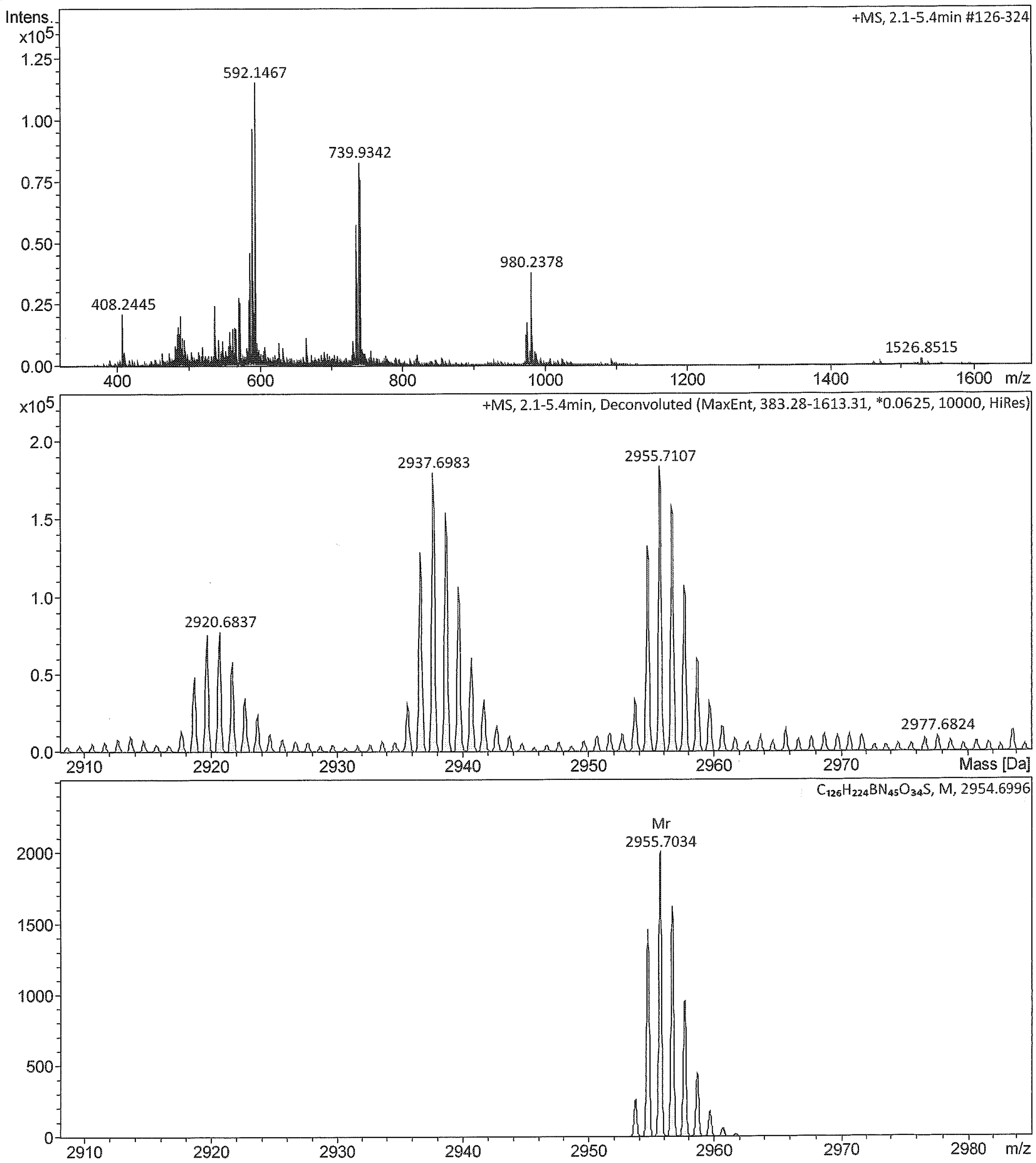

Display Report

Analysis Info

Analysis Name	E:\Data4\HEIKO\12_2023\Marius_Werner\JB_044_F_1.d	Acquisition Date	12/18/2023 9:37:01 AM
Method	2_2_HR_hohe_Massen_300-3000_pos_20-07-2023.m	Operator	Heiko Rudy
Sample Name	C126H224BN45O34S = 2954,700 u	Instrument	micrOTOF-Q 228888.10254
Comment			

Acquisition Parameter

Source Type	ESI	Ion Polarity	Positive	Set Nebulizer	0.3 Bar
Focus	Not active	Set Capillary	4500 V	Set Dry Heater	200 °C
Scan Begin	300 m/z	Set End Plate Offset	-500 V	Set Dry Gas	4.0 l/min
Scan End	3000 m/z	Set Collision Cell RF	700.0 Vpp	Set Divert Valve	Source

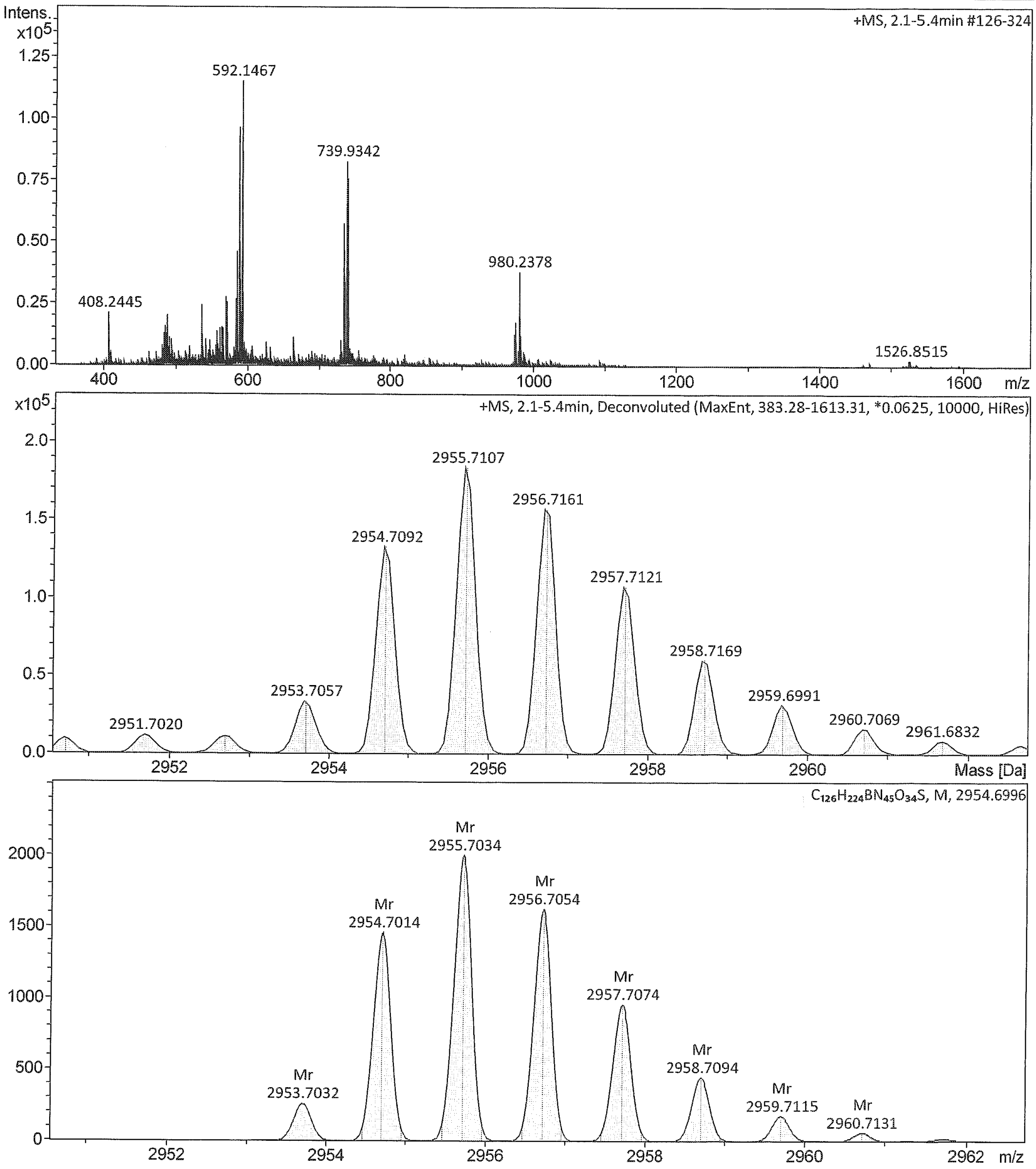

Display Report

Analysis Info

Analysis Name	E:\Data4\HEIKO\12_2023\Marius_Werner\JB_044_F_1.d	Acquisition Date	12/18/2023 9:37:01 AM
Method	2_2_HR_hohe_Massen_300-3000_pos_20-07-2023.m	Operator	Heiko Rudy
Sample Name	C126H224BN45O34S = 2954,700 u	Instrument	micrOTOF-Q 228888.10254
Comment			

Acquisition Parameter

Source Type	ESI	Ion Polarity	Positive	Set Nebulizer	0.3 Bar
Focus	Not active	Set Capillary	4500 V	Set Dry Heater	200 °C
Scan Begin	300 m/z	Set End Plate Offset	-500 V	Set Dry Gas	4.0 l/min
Scan End	3000 m/z	Set Collision Cell RF	700.0 Vpp	Set Divert Valve	Source

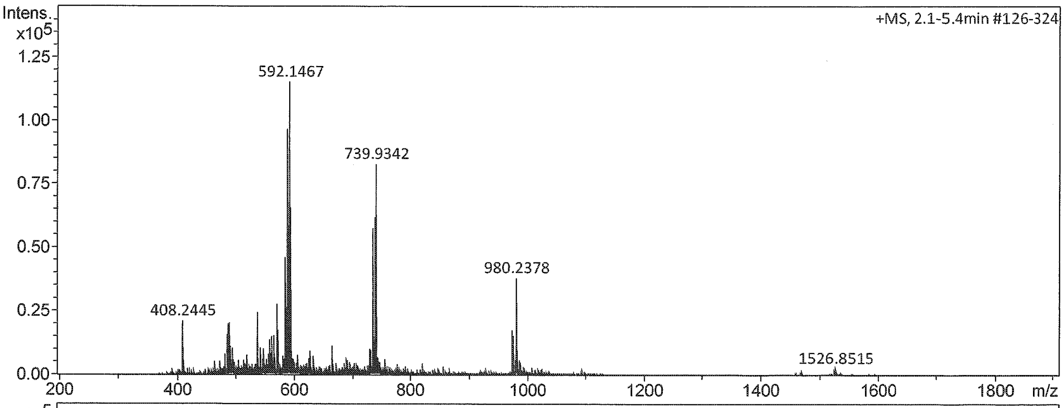

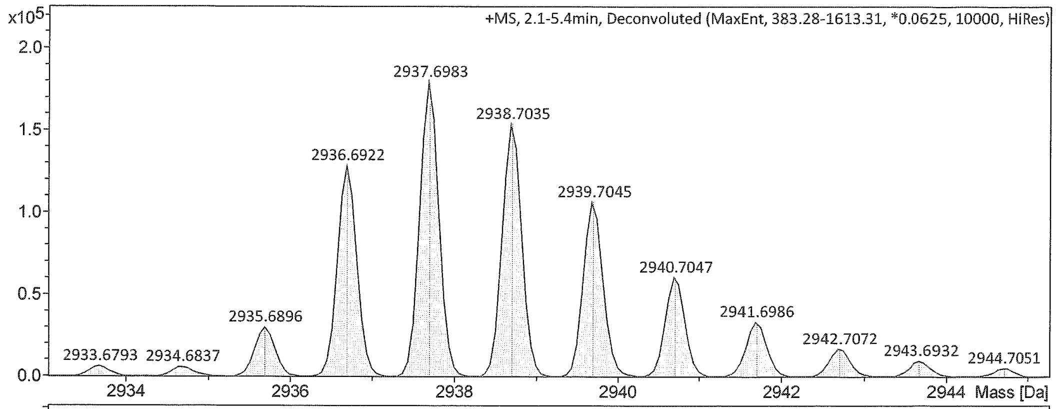

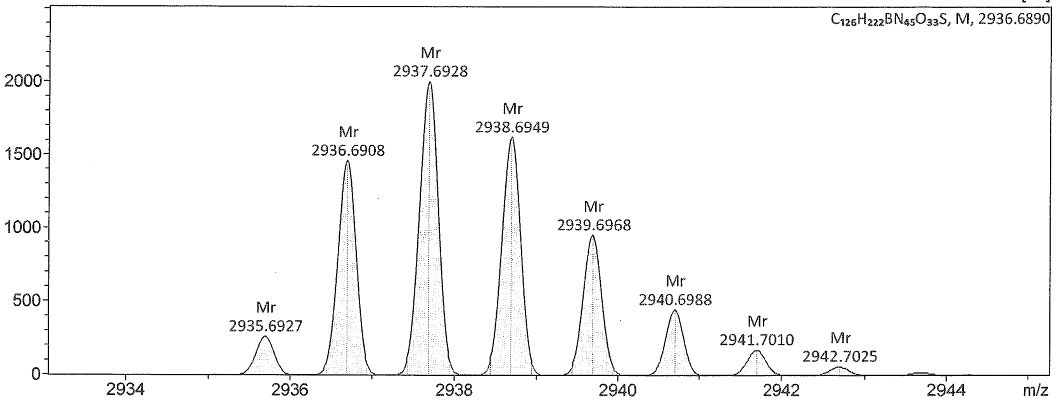

Display Report

Analysis Info

Analysis Name	E:\Data4\HEIKO\12_2023\Marius_Werner\JB_044_F_1.d	Acquisition Date	12/18/2023 9:37:01 AM
Method	2_2_HR_hohe_Massen_300-3000_pos_20-07-2023.m	Operator	Heiko Rudy
Sample Name	C126H224BN45O34S = 2954,700 u	Instrument	micrOTOF-Q 228888.10254
Comment			

Acquisition Parameter

Source Type	ESI	Ion Polarity	Positive	Set Nebulizer	0.3 Bar
Focus	Not active	Set Capillary	4500 V	Set Dry Heater	200 °C
Scan Begin	300 m/z	Set End Plate Offset	-500 V	Set Dry Gas	4.0 l/min
Scan End	3000 m/z	Set Collision Cell RF	700.0 Vpp	Set Divert Valve	Source

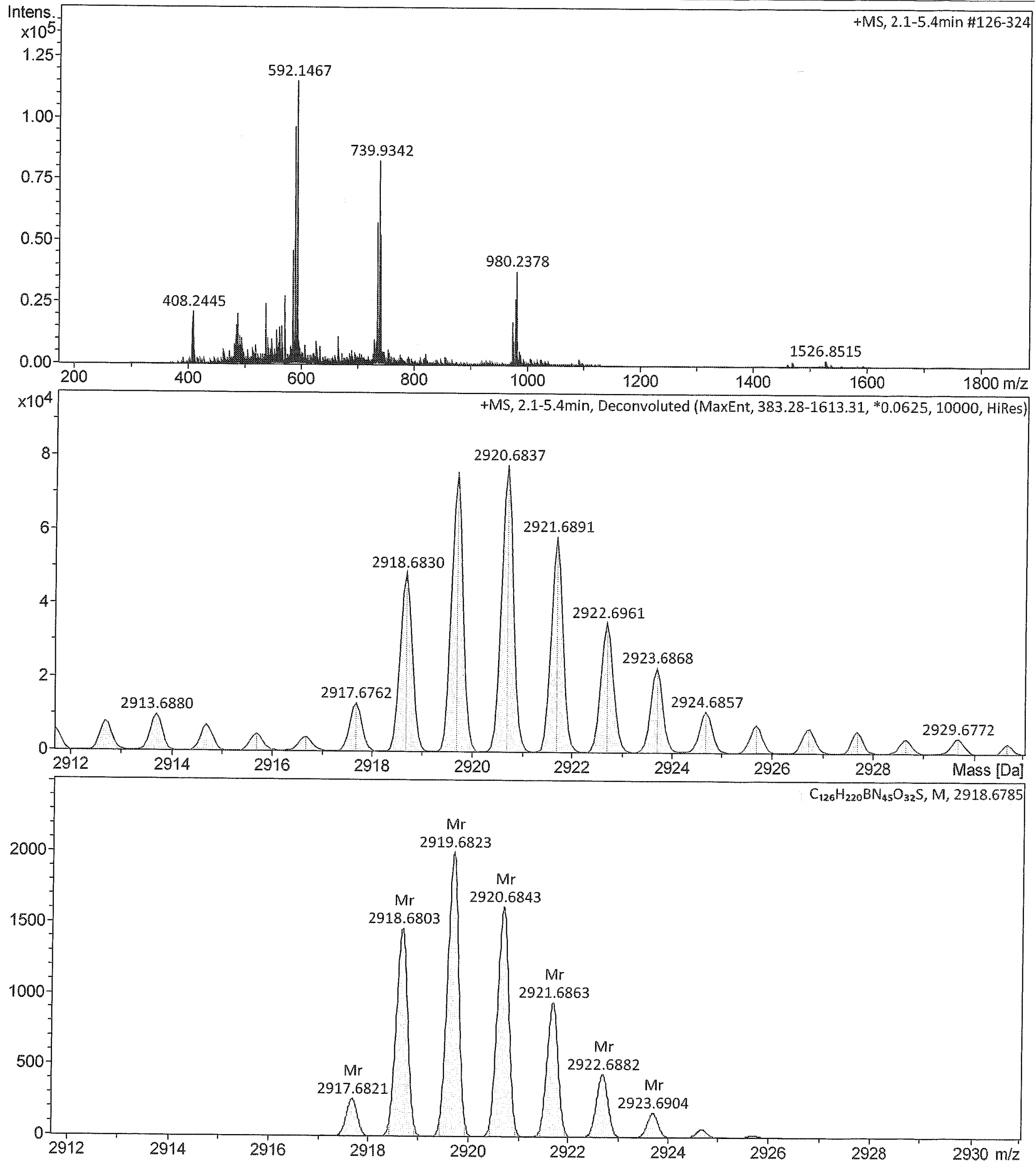

Pure **P4=B(OH)₂** prepared by [Cp*Ru(PPh₃)₂Cl]-catalyzed hydroboration (Reaction Conditions B).

Calculated mass: [M-2H_2O] 2918.7, [M-H_2O] 2936.7, [M] 2954.7.

Display Report

Analysis Info

Analysis Name	E:\Data4\HEIKO\03_2024\Marius Werner\K27M_07_20.6.d	Acquisition Date	3/28/2024 11:57:29 AM
Method	2_2_HR_hohe_Massen_300-3000_pos_20-07-2023.m	Operator	Heiko Rudy
Sample Name	C126H224BN45O34S = 2954,70 u	Instrument	micrOTOF-Q 228888.10254
Comment			

Acquisition Parameter

Source Type	ESI	Ion Polarity	Positive	Set Nebulizer	0.3 Bar
Focus	Not active	Set Capillary	4500 V	Set Dry Heater	200 °C
Scan Begin	300 m/z	Set End Plate Offset	-500 V	Set Dry Gas	4.0 l/min
Scan End	3000 m/z	Set Collision Cell RF	700.0 Vpp	Set Divert Valve	Source

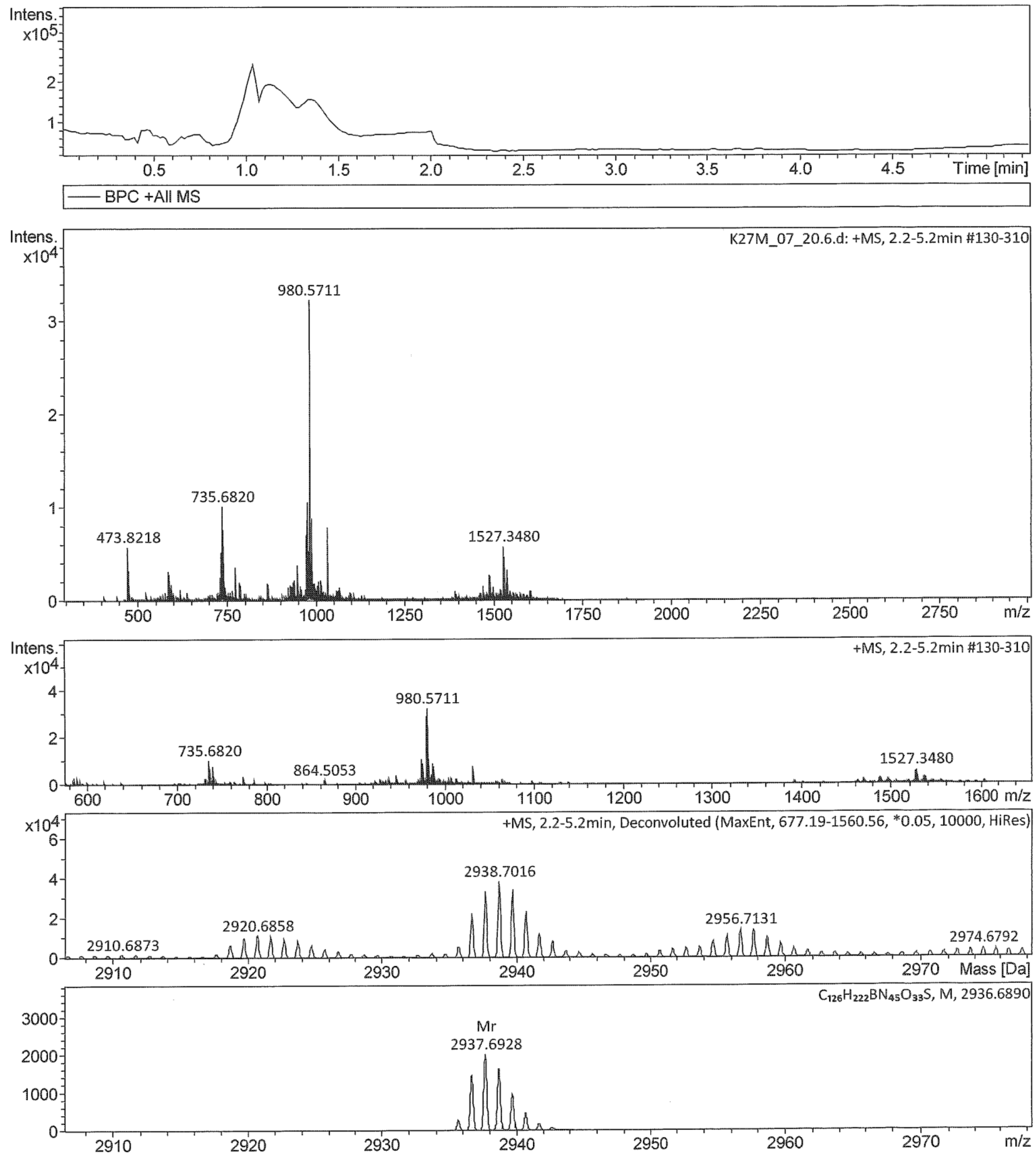

Bruker Compass DataAnalysis 4.2 printed: 3/28/2024 12:08:44 PM by: Heiko Rudy Page 1 of 1

Display Report

Analysis Info

Analysis Name	E:\Data4\HEIKO\03_2024\MariusWerner\K27M_07_20.6.d	Acquisition Date	3/28/2024 11:57:29 AM
Method	2_2_HR_hohe_Massen_300-3000_pos_20-07-2023.m	Operator	Heiko Rudy
Sample Name	C126H224BN45O34S = 2954,70 u	Instrument	micrOTOF-Q 228888.10254
Comment			

Acquisition Parameter

Source Type	ESI	Ion Polarity	Positive	Set Nebulizer	0.3 Bar
Focus	Not active	Set Capillary	4500 V	Set Dry Heater	200 °C
Scan Begin	300 m/z	Set End Plate Offset	-500 V	Set Dry Gas	4.0 l/min
Scan End	3000 m/z	Set Collision Cell RF	700.0 Vpp	Set Divert Valve	Source

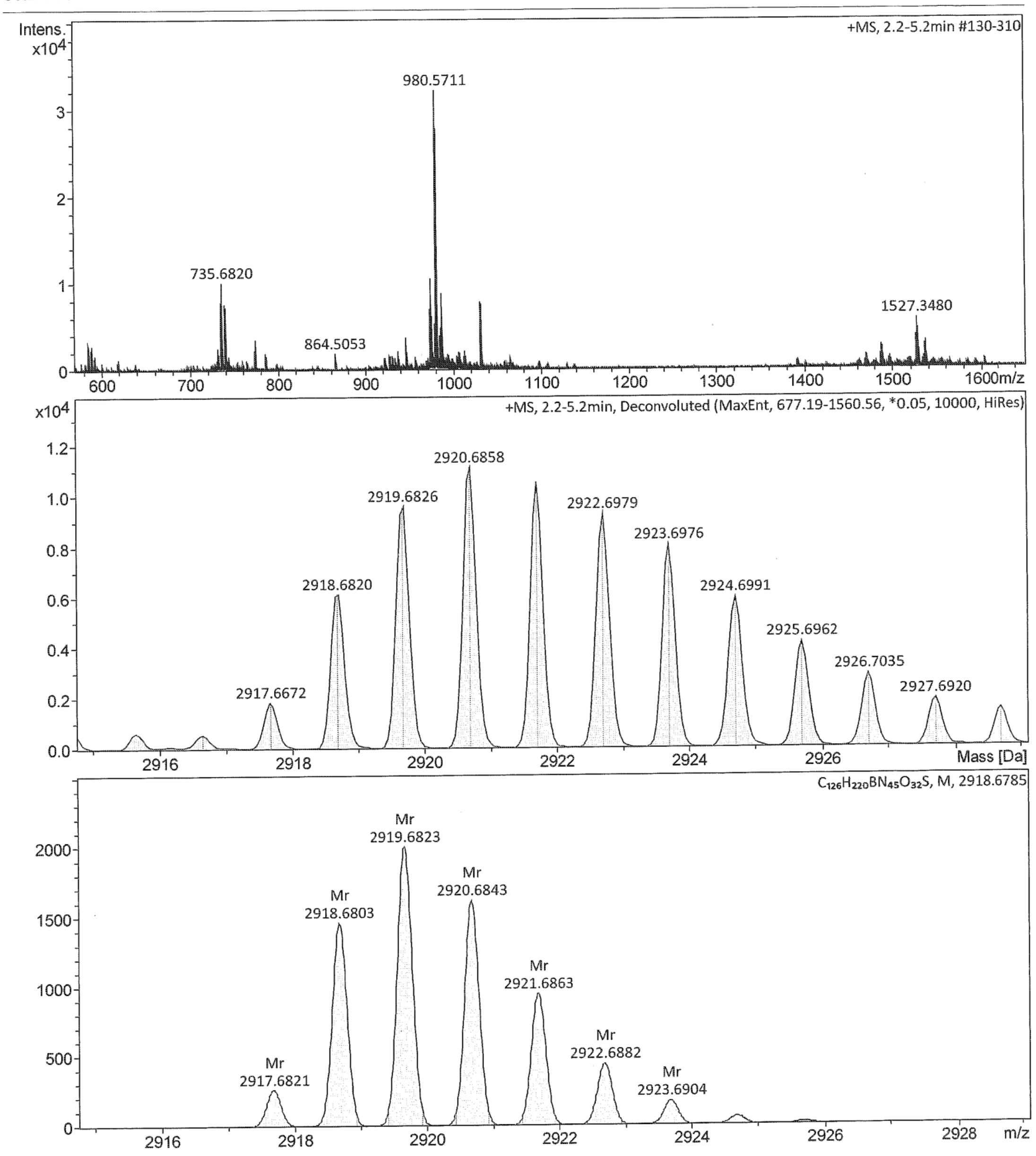

Display Report

Analysis Info

Analysis Name	E:\Data4\HEIKO\03_2024\Marius Werner\K27M_07_20.6.d	Acquisition Date	3/28/2024 11:57:29 AM
Method	2_2_HR_hohe_Massen_300-3000_pos_20-07-2023.m	Operator	Heiko Rudy
Sample Name	C126H224BN45O34S = 2954,70 u	Instrument	micrOTOF-Q 228888.10254
Comment			

Acquisition Parameter

Source Type	ESI	Ion Polarity	Positive	Set Nebulizer	0.3 Bar
Focus	Not active	Set Capillary	4500 V	Set Dry Heater	200 °C
Scan Begin	300 m/z	Set End Plate Offset	-500 V	Set Dry Gas	4.0 l/min
Scan End	3000 m/z	Set Collision Cell RF	700.0 Vpp	Set Divert Valve	Source

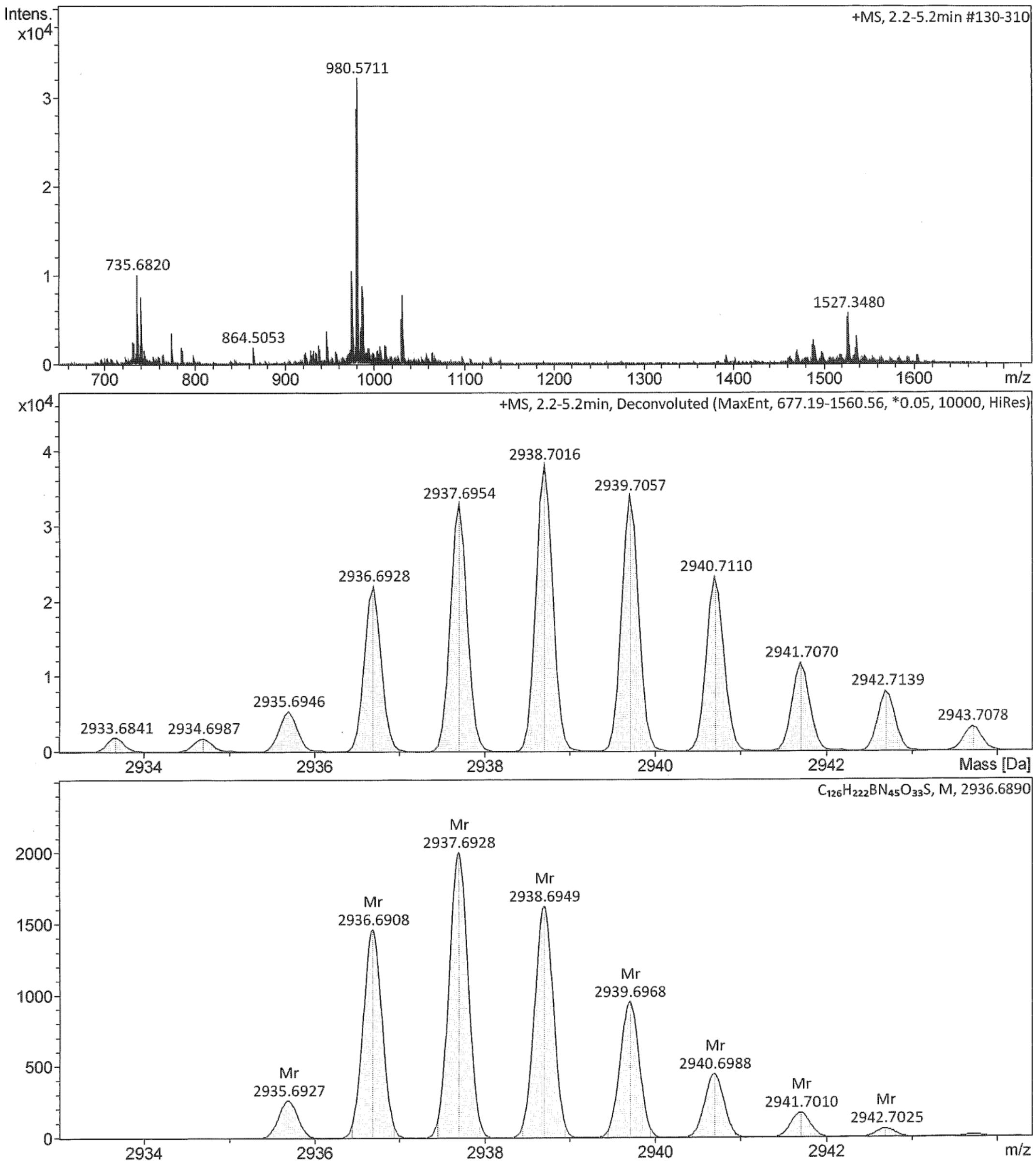

Display Report

Analysis Info

Analysis Name	E:\Data4\HEIKO\03_2024\Marius Werner\K27M_07_20.6.d	Acquisition Date	3/28/2024 11:57:29 AM
Method	2_2_HR_hohe_Massen_300-3000_pos_20-07-2023.m	Operator	Heiko Rudy
Sample Name	C126H224BN45O34S = 2954,70 u	Instrument	micrOTOF-Q 228888.10254
Comment			

Acquisition Parameter

Source Type	ESI	Ion Polarity	Positive	Set Nebulizer	0.3 Bar
Focus	Not active	Set Capillary	4500 V	Set Dry Heater	200 °C
Scan Begin	300 m/z	Set End Plate Offset	-500 V	Set Dry Gas	4.0 l/min
Scan End	3000 m/z	Set Collision Cell RF	700.0 Vpp	Set Divert Valve	Source

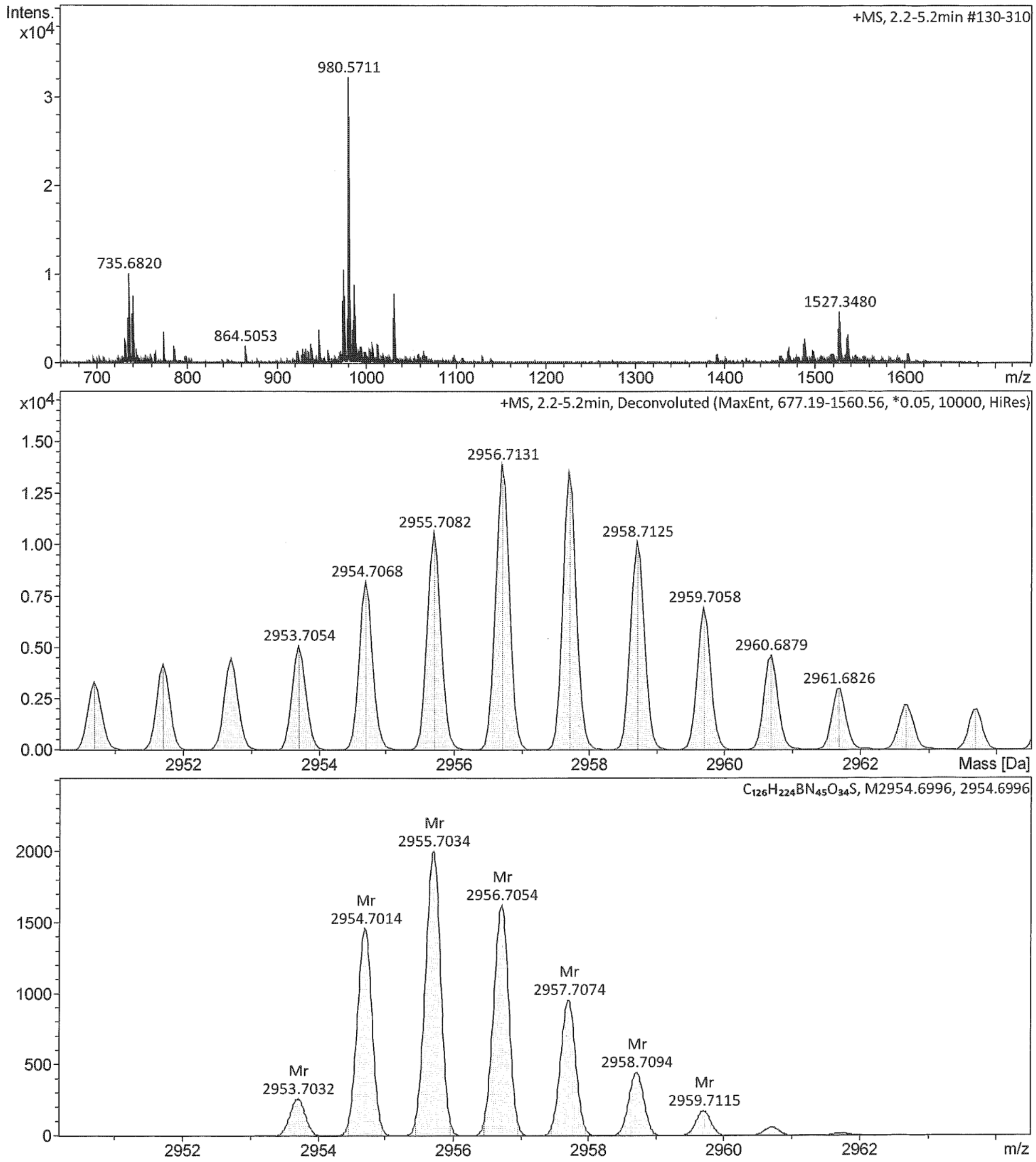

Pure **P6=B(OH)₂** prepared by [Ru(CO)(Cl)H(PPh₃)₃]-catalyzed hydroboration (Reaction Conditions A).

Calculated mass: [M-2H_2O] 3963.0, [M-H_2O] 3981.0.

Display Report

Analysis Info

Analysis Name	E:\Data4\HEIKO\09_2023\Marius_Werner\LH_HB_19.d	Acquisition Date	9/11/2023 7:35:44 AM
Method	2_2_HR_hohe_Massen_300-3000_pos_20-07-2023.m	Operator	Heiko Rudy
Sample Name	C176H272BN55O52 = 3999,042 u	Instrument	micrOTOF-Q 228888.10254
Comment			

Acquisition Parameter

Source Type	ESI	Ion Polarity	Positive	Set Nebulizer	0.3 Bar
Focus	Not active	Set Capillary	4500 V	Set Dry Heater	200 °C
Scan Begin	300 m/z	Set End Plate Offset	-500 V	Set Dry Gas	4.0 l/min
Scan End	3000 m/z	Set Collision Cell RF	700.0 Vpp	Set Divert Valve	Source

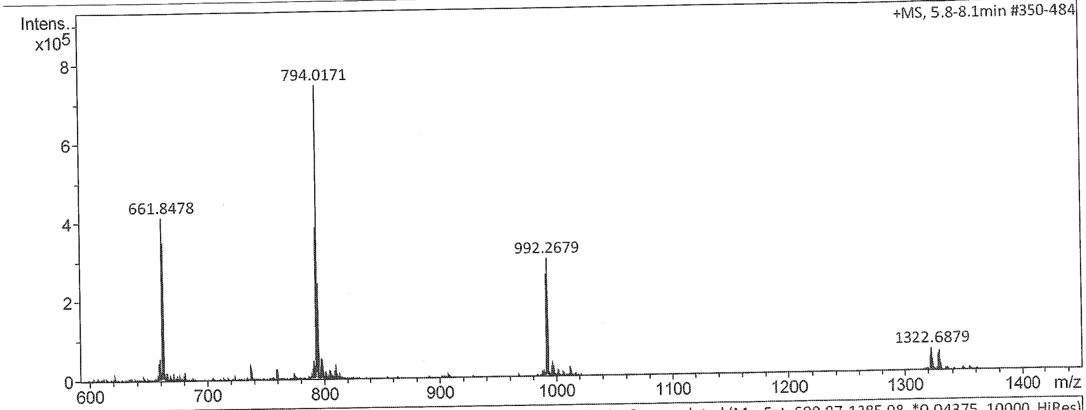

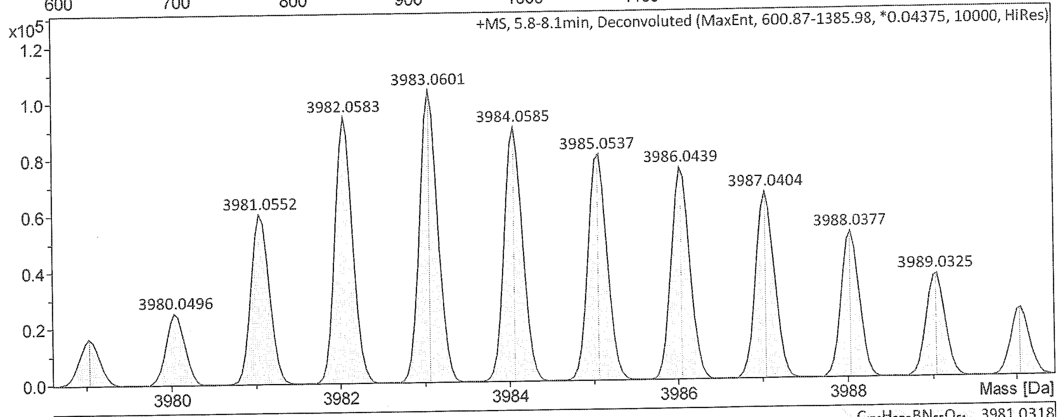

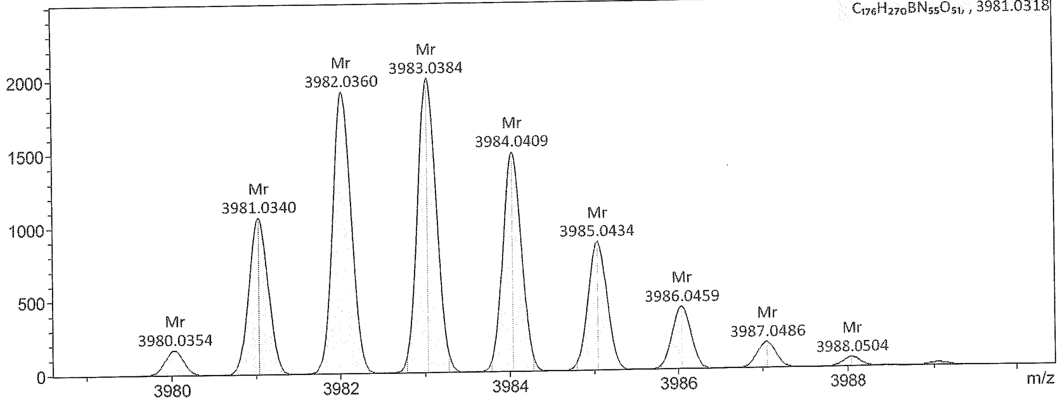

Bruker Compass DataAnalysis 4.2 printed: 9/11/2023 7:53:26 AM by: Heiko Rudy Page 1 of 1

Display Report

Analysis Info

Analysis Name	E:\Data4\HEIKO\09_2023\Marius_Werner\LH_HB_19.d	Acquisition Date	9/11/2023 7:35:44 AM
Method	2_2_HR_hohe_Massen_300-3000_pos_20-07-2023.m	Operator	Heiko Rudy
Sample Name	C176H272BN55O52 = 3999,042 u	Instrument	micrOTOF-Q 228888.10254
Comment			

Acquisition Parameter

Source Type	ESI	Ion Polarity	Positive	Set Nebulizer	0.3 Bar
Focus	Not active	Set Capillary	4500 V	Set Dry Heater	200 °C
Scan Begin	300 m/z	Set End Plate Offset	-500 V	Set Dry Gas	4.0 l/min
Scan End	3000 m/z	Set Collision Cell RF	700.0 Vpp	Set Divert Valve	Source

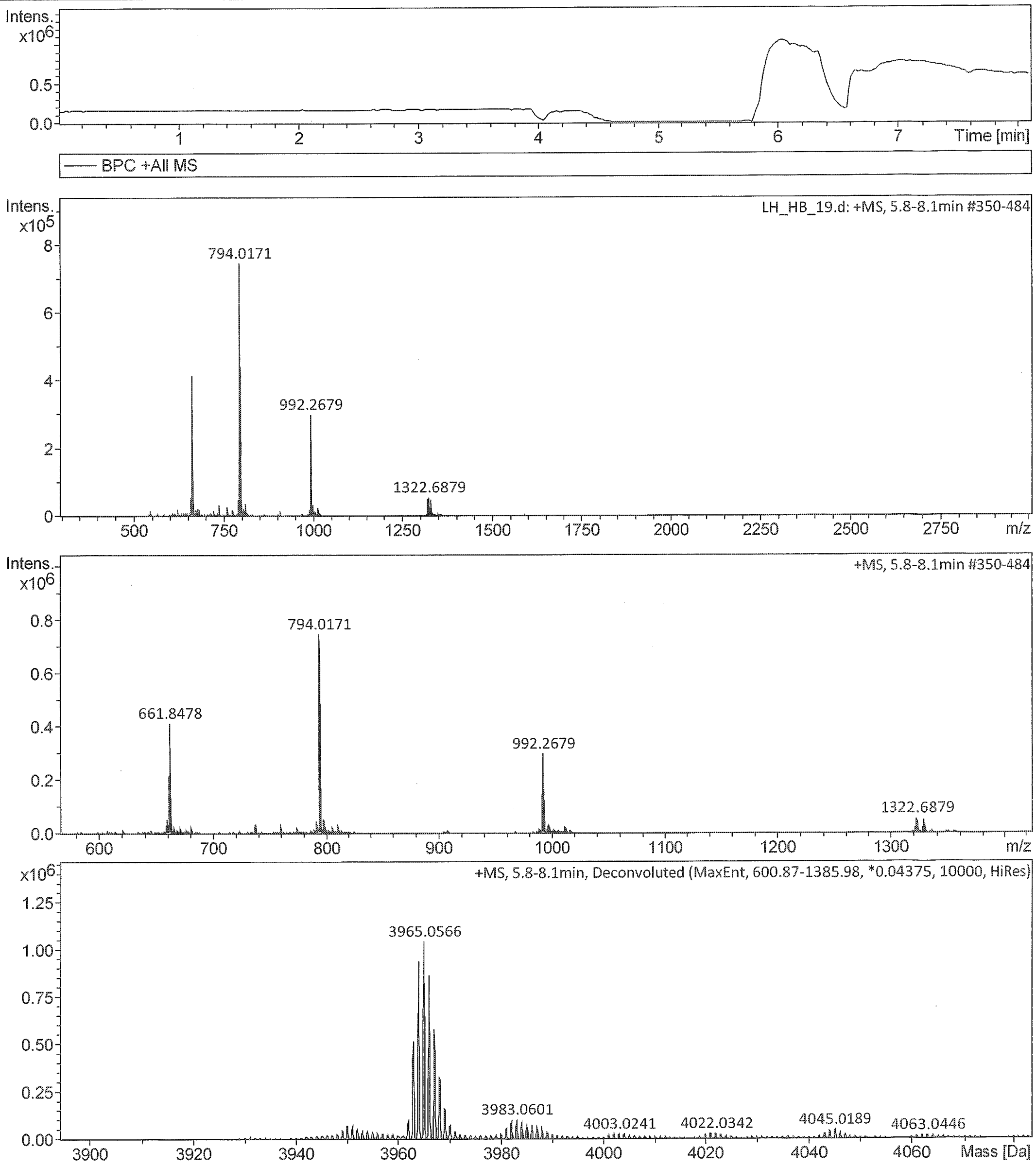

Display Report

Analysis Info

Analysis Name	E:\Data4\HEIKO\09_2023\Marius_Werner\LH_HB_19.d	Acquisition Date	9/11/2023 7:35:44 AM
Method	2_2_HR_hohe_Massen_300-3000_pos_20-07-2023.m	Operator	Heiko Rudy
Sample Name	C176H272BN55O52 = 3999,042 u	Instrument	micrOTOF-Q 228888.10254
Comment			

Acquisition Parameter

Source Type	ESI	Ion Polarity	Positive	Set Nebulizer	0.3 Bar
Focus	Not active	Set Capillary	4500 V	Set Dry Heater	200 °C
Scan Begin	300 m/z	Set End Plate Offset	-500 V	Set Dry Gas	4.0 l/min
Scan End	3000 m/z	Set Collision Cell RF	700.0 Vpp	Set Divert Valve	Source

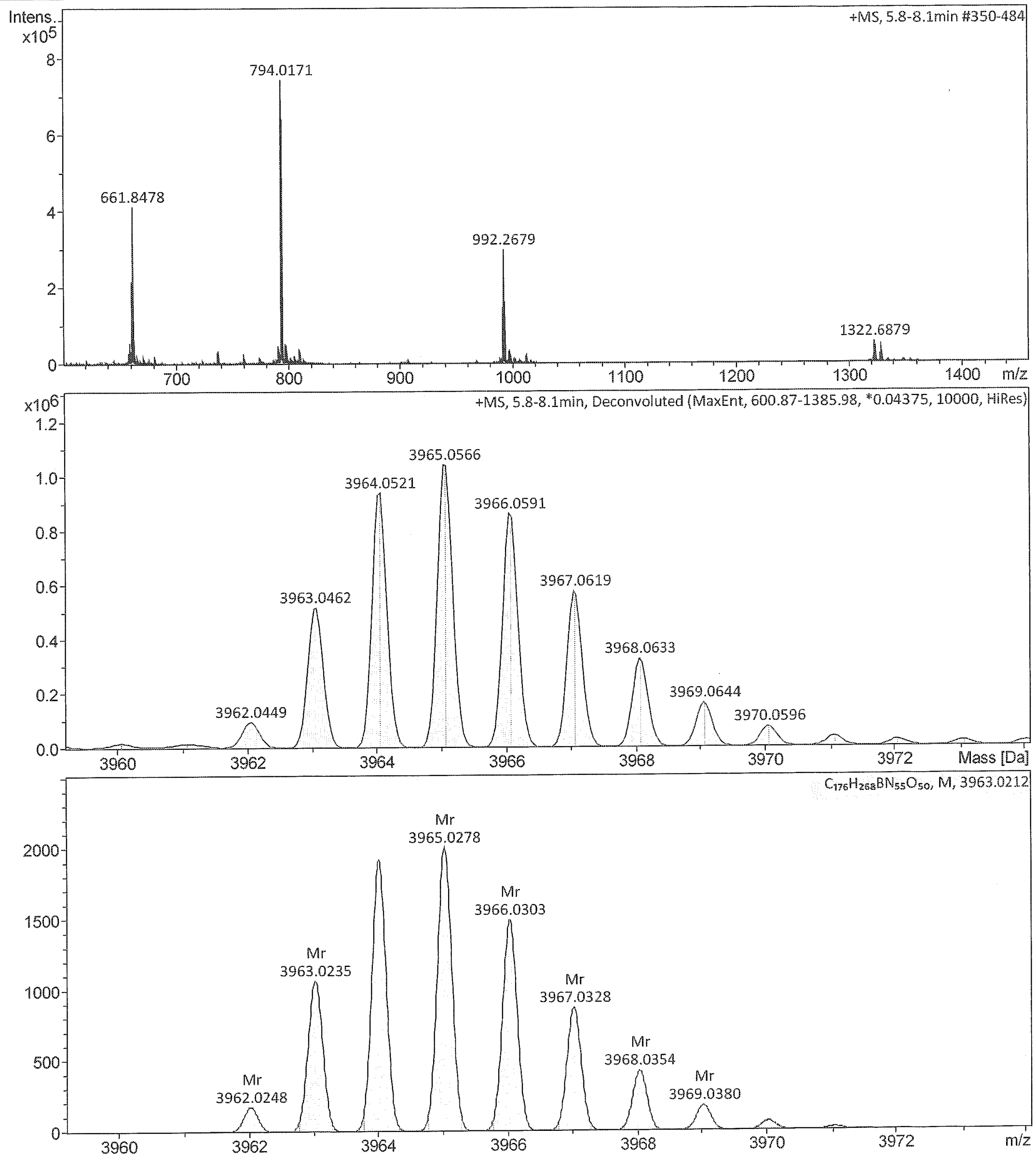

Bruker Compass DataAnalysis 4.2 printed: 9/11/2023 7:51:22 AM by: Heiko Rudy Page 1 of 1

Pure **P6=B(OH)₂** prepared by [Cp*Ru(PPh₃)₂Cl]-catalyzed hydroboration (Reaction Conditions B).

Calculated mass: [M-2H₂O] 3963.0, [M-H₂O] 3981.0.

Display Report

Analysis Info

Analysis Name	E:\Data4\HEIKO\03_2024\MariusWerner\WW-02_pure_pos.d	Acquisition Date	3/28/2024 9:46:11 AM
Method	2_2_HR_hohe_Massen_300-3000_pos_20-07-2023.m	Operator	Heiko Rudy
Sample Name	C176H272BN55O52 = 3999,04 u	Instrument	micrOTOF-Q 228888.10254
Comment			

Acquisition Parameter

Source Type	ESI	Ion Polarity	Positive	Set Nebulizer	0.3 Bar
Focus	Not active	Set Capillary	4500 V	Set Dry Heater	200 °C
Scan Begin	300 m/z	Set End Plate Offset	-500 V	Set Dry Gas	4.0 l/min
Scan End	3000 m/z	Set Collision Cell RF	700.0 Vpp	Set Divert Valve	Source

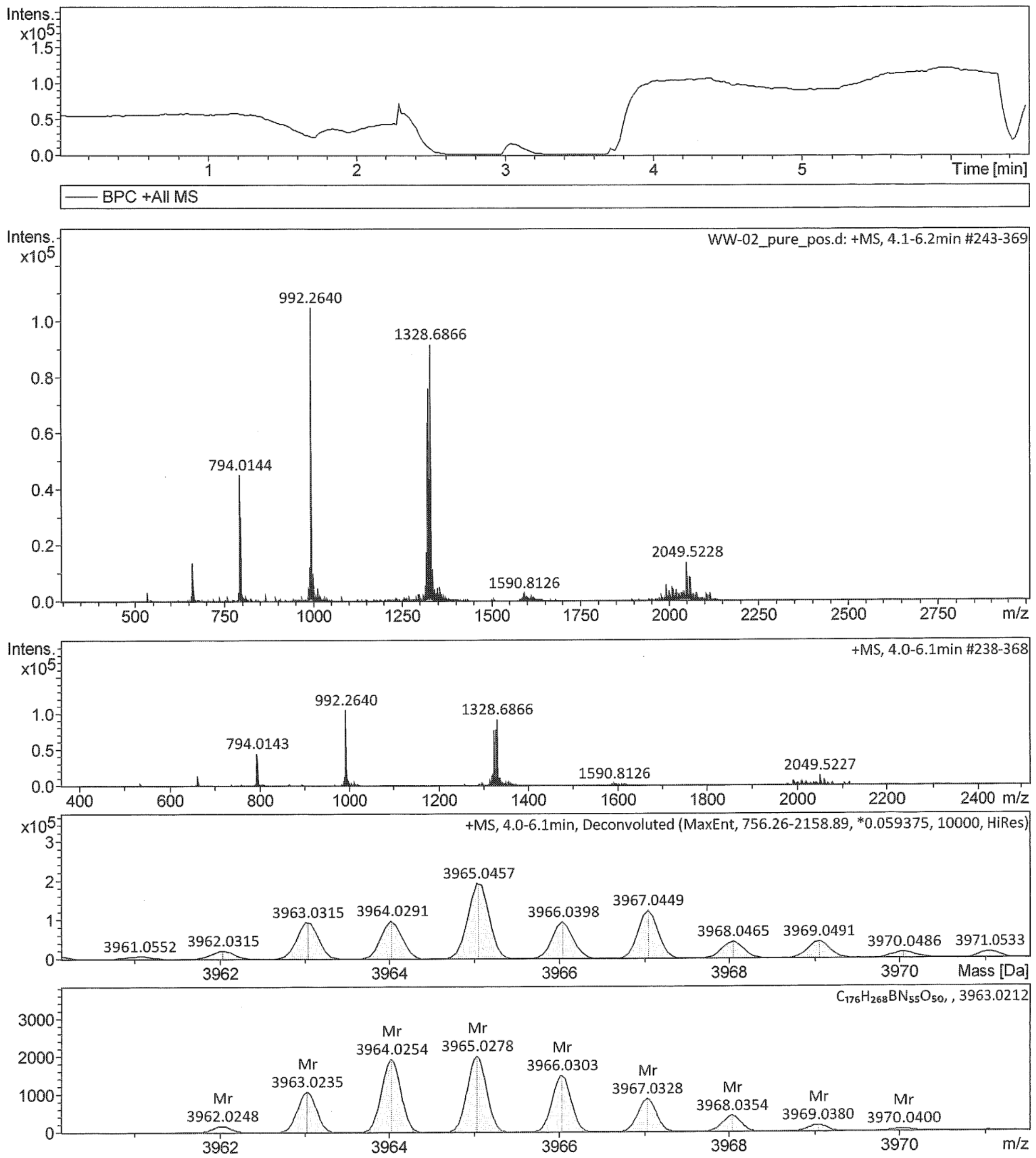

Bruker Compass DataAnalysis 4.2 printed: 3/28/2024 10:21:44 AM by: Heiko Rudy Page 1 of 1

Display Report

Analysis Info

Analysis Name	E:\Data4\HEIKO\03_2024\MariusWerner\WW-02_pure_pos.d	Acquisition Date	3/28/2024 9:46:11 AM
Method	2_2_HR_hohe_Massen_300-3000_pos_20-07-2023.m	Operator	Heiko Rudy
Sample Name	C176H272BN55O52 = 3999,04 u	Instrument	micrOTOF-Q 228888.10254
Comment			

Acquisition Parameter

Source Type	ESI	Ion Polarity	Positive	Set Nebulizer	0.3 Bar
Focus	Not active	Set Capillary	4500 V	Set Dry Heater	200 °C
Scan Begin	300 m/z	Set End Plate Offset	-500 V	Set Dry Gas	4.0 l/min
Scan End	3000 m/z	Set Collision Cell RF	700.0 Vpp	Set Divert Valve	Source

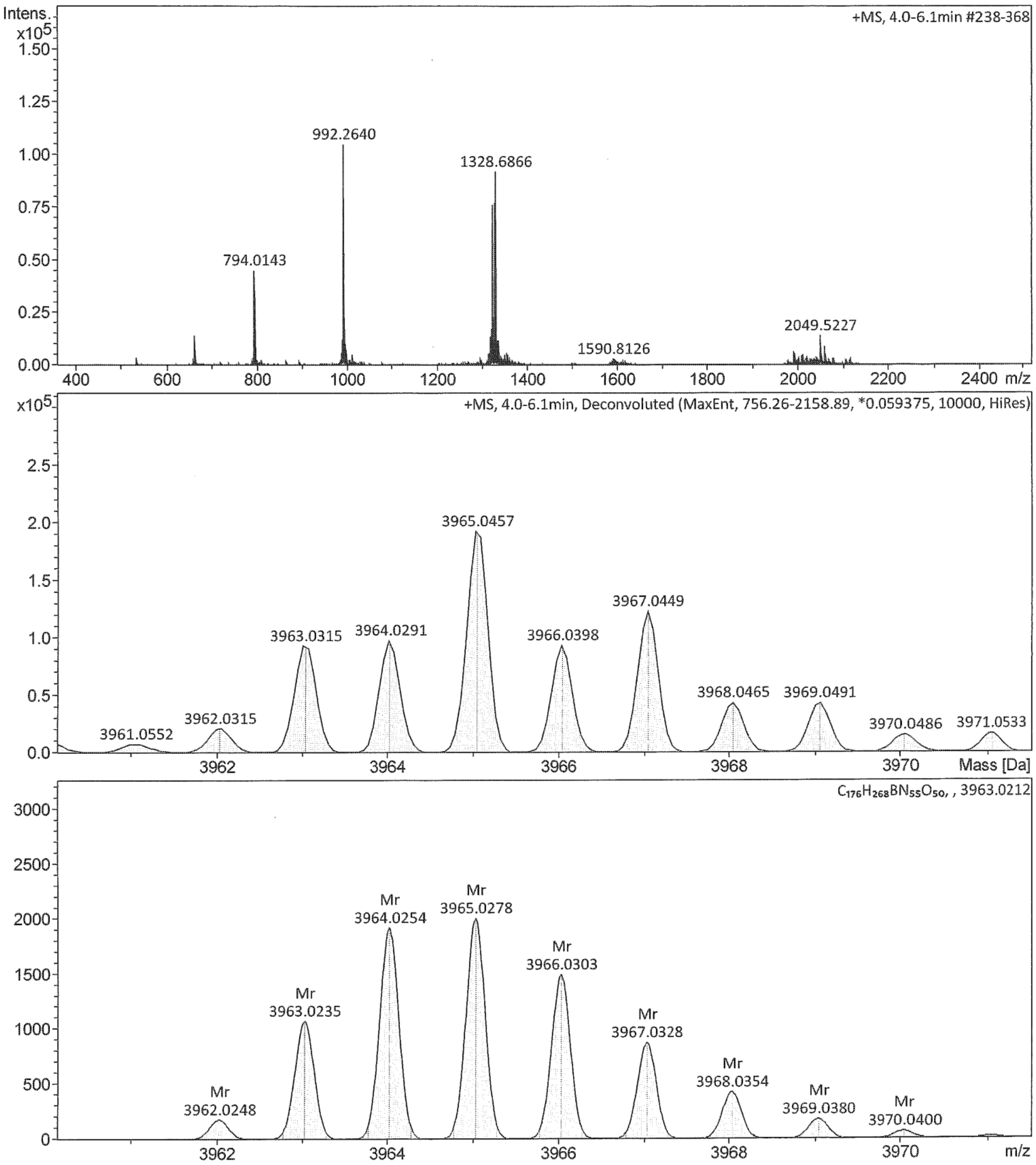

Crude **P6=B(OH)$_2$** prepared by iridium-catalyzed hydroboration.

Calculated mass: [M-2H$_2$O] 3963.0.

Display Report

Analysis Info

Analysis Name	E:\Data4\HEIKO\12_2023\Marius_Werner\LH_HB_06_crude.d	Acquisition Date	12/18/2023 10:06:07 AM
Method	2_2_HR_hohe_Massen_300-3000_pos_20-07-2023.m	Operator	Heiko Rudy
Sample Name	C176H272BN55O52 = 3999,042 u	Instrument	micrOTOF-Q 228888.10254
Comment			

Acquisition Parameter

Source Type	ESI	Ion Polarity	Positive	Set Nebulizer	0.3 Bar
Focus	Not active	Set Capillary	4500 V	Set Dry Heater	200 °C
Scan Begin	300 m/z	Set End Plate Offset	-500 V	Set Dry Gas	4.0 l/min
Scan End	3000 m/z	Set Collision Cell RF	700.0 Vpp	Set Divert Valve	Source

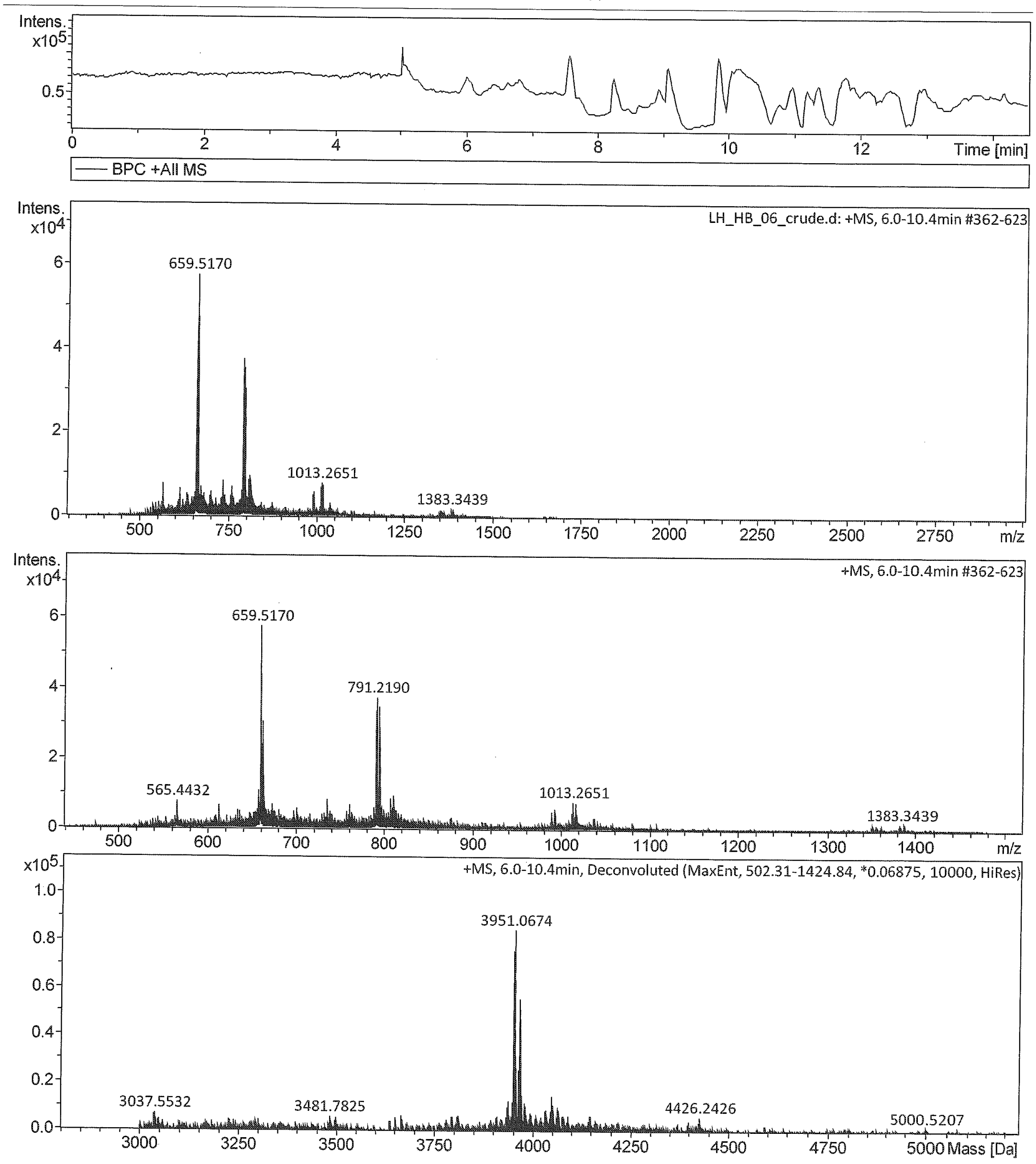

Bruker Compass DataAnalysis 4.2 printed: 12/18/2023 10:26:53 AM by: Heiko Rudy Page 1 of 1

Display Report

Analysis Info

Analysis Name	E:\Data4\HEIKO\12_2023\Marius_Werner\LH_HB_06_crude.d	Acquisition Date	12/18/2023 10:06:07 AM
Method	2_2_HR_hohe_Massen_300-3000_pos_20-07-2023.m	Operator	Heiko Rudy
Sample Name	C176H272BN55O52 = 3999,042 u	Instrument	micrOTOF-Q 228888.10254
Comment			

Acquisition Parameter

Source Type	ESI	Ion Polarity	Positive	Set Nebulizer	0.3 Bar
Focus	Not active	Set Capillary	4500 V	Set Dry Heater	200 °C
Scan Begin	300 m/z	Set End Plate Offset	-500 V	Set Dry Gas	4.0 l/min
Scan End	3000 m/z	Set Collision Cell RF	700.0 Vpp	Set Divert Valve	Source

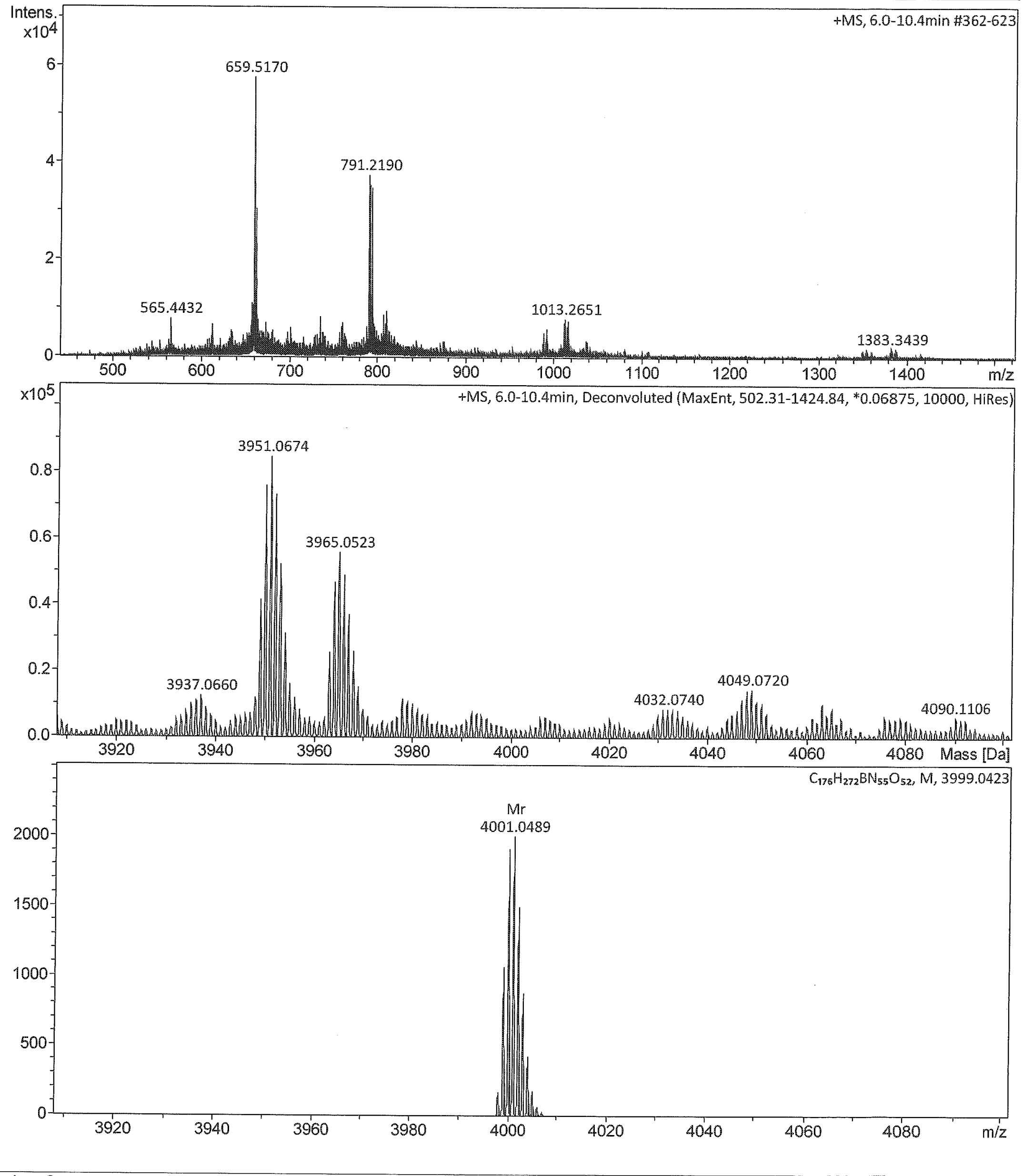

Display Report

Analysis Info

Analysis Name	E:\Data4\HEIKO\12_2023\Marius_Werner\LH_HB_06_crude.d	Acquisition Date	12/18/2023 10:06:07 AM
Method	2_2_HR_hohe_Massen_300-3000_pos_20-07-2023.m	Operator	Heiko Rudy
Sample Name	C176H272BN55O52 = 3999,042 u	Instrument	micrOTOF-Q 228888.10254
Comment			

Acquisition Parameter

Source Type	ESI	Ion Polarity	Positive	Set Nebulizer	0.3 Bar
Focus	Not active	Set Capillary	4500 V	Set Dry Heater	200 °C
Scan Begin	300 m/z	Set End Plate Offset	-500 V	Set Dry Gas	4.0 l/min
Scan End	3000 m/z	Set Collision Cell RF	700.0 Vpp	Set Divert Valve	Source

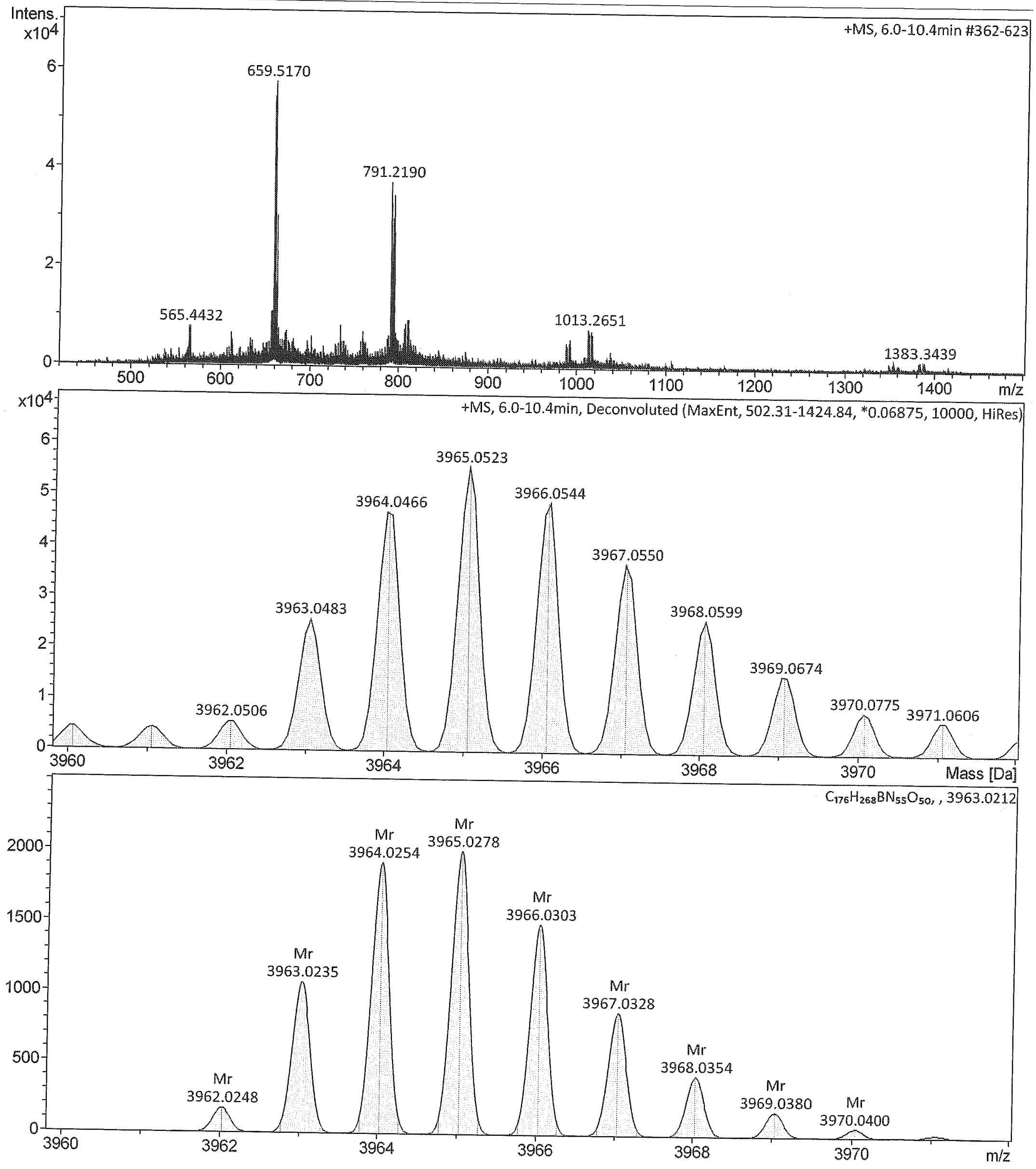

Bruker Compass DataAnalysis 4.2 printed: 12/18/2023 10:30:57 AM by: Heiko Rudy Page 1 of 1

Pure **P7=B(OH)$_2$** prepared by [Cp*Ru(PPh$_3$)$_2$Cl]-catalyzed hydroboration (Reaction Conditions B).

Calculated mass: [M-2H_2O] 6842.5.

Display Report

Analysis Info

Analysis Name	E:\Data4\HEIKO\03_2024\MariusWerner\SH3-04_pure_pos.d	Acquisition Date	3/28/2024 10:24:19 AM
Method	2_2_HR_hohe_Massen_300-3000_pos_20-07-2023.m	Operator	Heiko Rudy
Sample Name	C312H470BN83O93 = 6878,47 u	Instrument	micrOTOF-Q 228888.10254
Comment			

Acquisition Parameter

Source Type	ESI	Ion Polarity	Positive	Set Nebulizer	0.3 Bar
Focus	Not active	Set Capillary	4500 V	Set Dry Heater	200 °C
Scan Begin	300 m/z	Set End Plate Offset	-500 V	Set Dry Gas	4.0 l/min
Scan End	3000 m/z	Set Collision Cell RF	700.0 Vpp	Set Divert Valve	Source

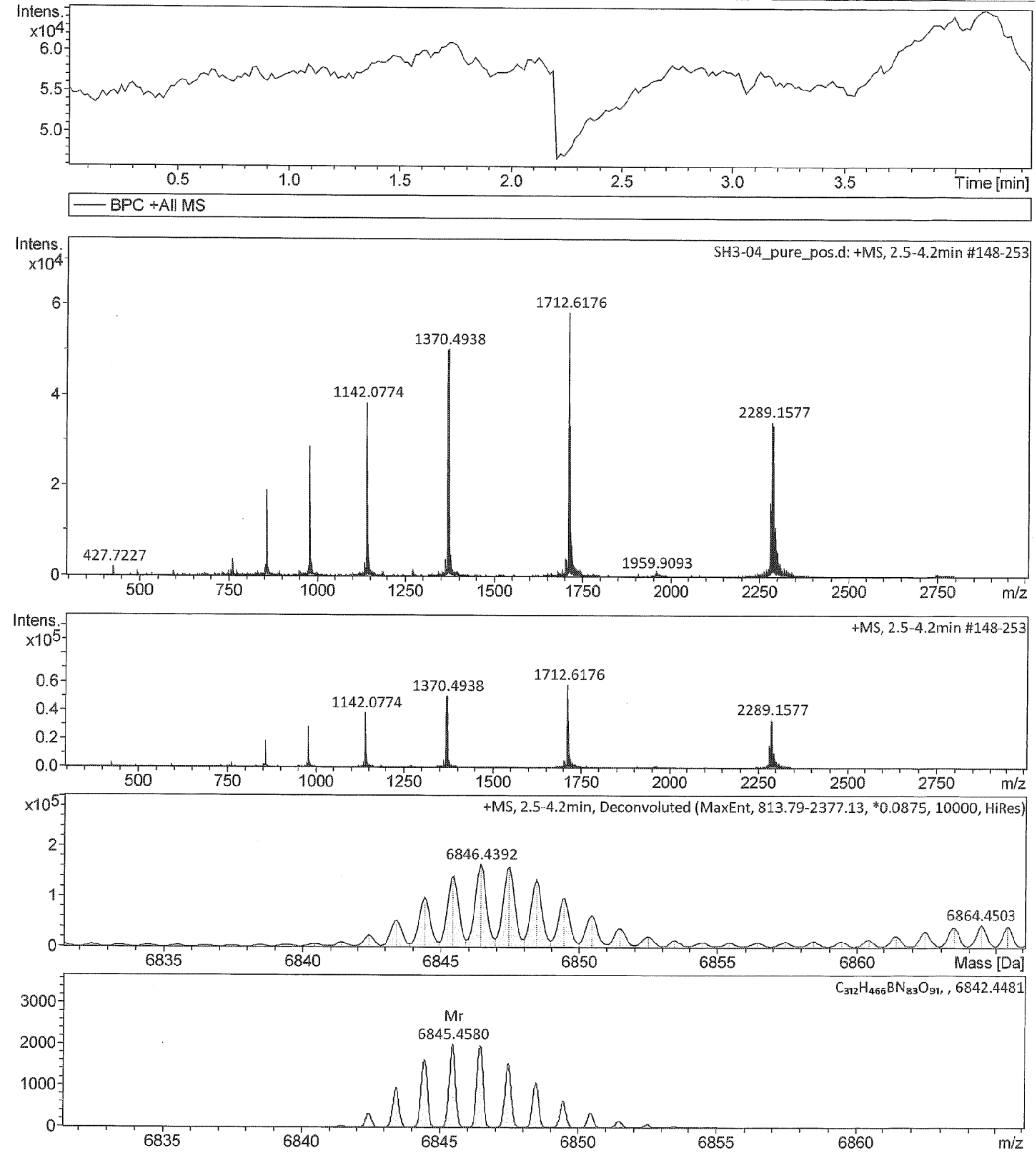

Bruker Compass DataAnalysis 4.2 printed: 3/28/2024 10:40:45 AM by: Heiko Rudy Page 1 of 1

Display Report

Analysis Info

Analysis Name	E:\Data4\HEIKO\03_2024\MariusWerner\SH3-04_pure_pos.d	Acquisition Date	3/28/2024 10:24:19 AM
Method	2_2_HR_hohe_Massen_300-3000_pos_20-07-2023.m	Operator	Heiko Rudy
Sample Name	C312H470BN83O93 = 6878,47 u	Instrument	micrOTOF-Q 228888.10254
Comment			

Acquisition Parameter

Source Type	ESI	Ion Polarity	Positive	Set Nebulizer	0.3 Bar
Focus	Not active	Set Capillary	4500 V	Set Dry Heater	200 °C
Scan Begin	300 m/z	Set End Plate Offset	-500 V	Set Dry Gas	4.0 l/min
Scan End	3000 m/z	Set Collision Cell RF	700.0 Vpp	Set Divert Valve	Source

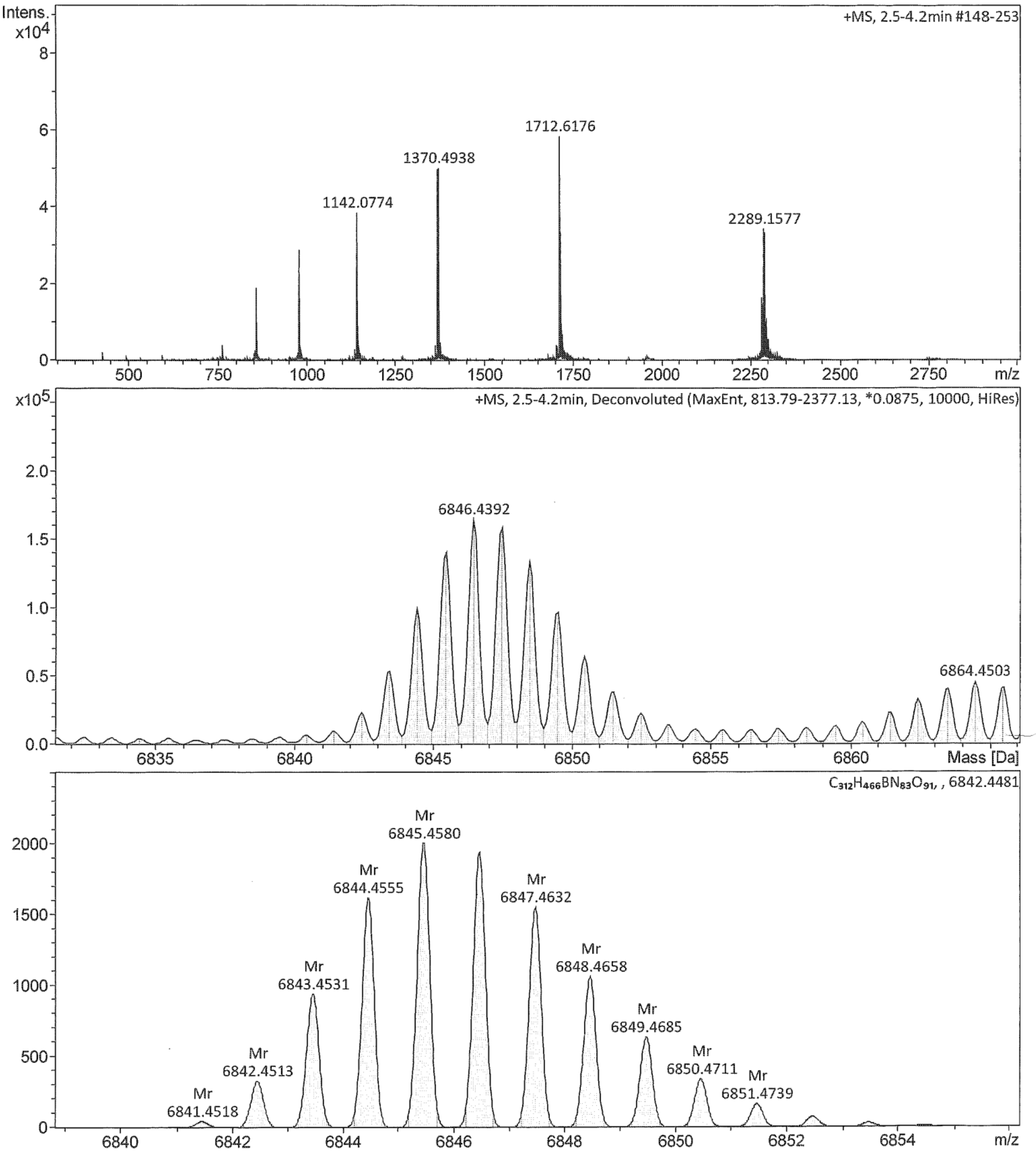

Pure **P11=B(OH)$_2$** prepared by [Cp*Ru(PPh$_3$)$_2$Cl]-catalyzed hydroboration (Reaction Conditions B).

Calculated mass: [M-2H$_2$O] 6842.5.

Display Report

Analysis Info

Analysis Name E:\Data4\HEIKO\03_2024\Marius Werner\CTD_06_pure.d
Method 2_1_b_HR_kleine_Massen_100-1550_pos_25-07-2023.m
Sample Name C34H52BN8O16P = 870,33 u
Comment

Acquisition Date 3/28/2024 12:25:29 PM
Operator Heiko Rudy
Instrument micrOTOF-Q 228888.10254

Acquisition Parameter

Source Type	ESI	Ion Polarity	Positive	Set Nebulizer	0.3 Bar
Focus	Not active	Set Capillary	4500 V	Set Dry Heater	200 °C
Scan Begin	100 m/z	Set End Plate Offset	-500 V	Set Dry Gas	4.0 l/min
Scan End	1550 m/z	Set Collision Cell RF	240.0 Vpp	Set Divert Valve	Source

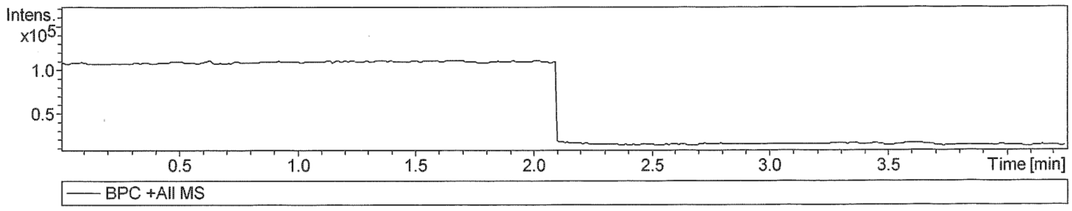

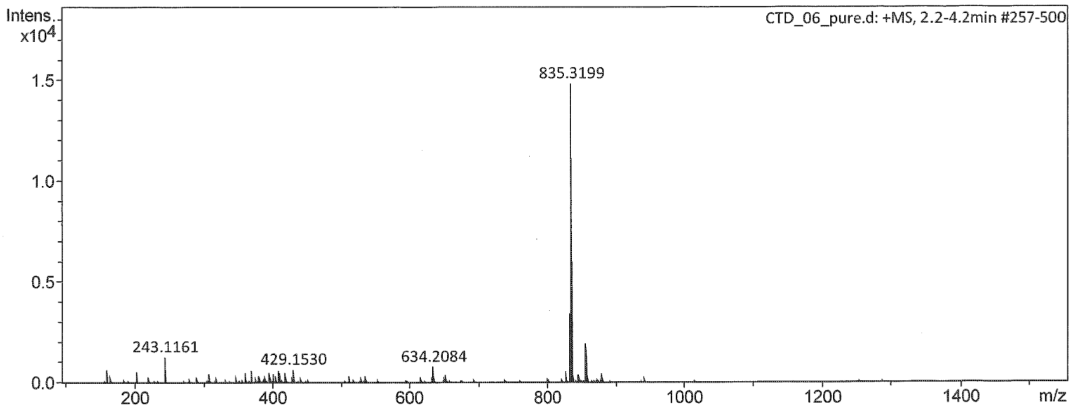

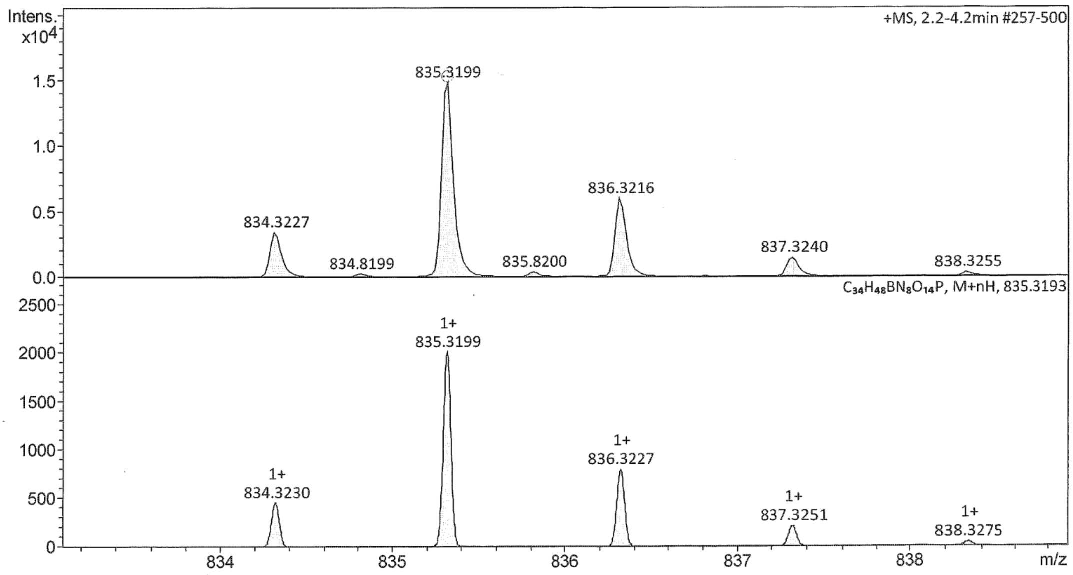

Bruker Compass DataAnalysis 4.2 printed: 3/28/2024 12:35:06 PM by: Heiko Rudy Page 1 of 1

Mass Spectrum SmartFormula Report

Analysis Info

Analysis Name	E:\Data4\HEIKO\03_2024\Marius Werner\CTD_06_pure.d	Acquisition Date	3/28/2024 12:25:29 PM
Method	2_1_b_HR_kleine_Massen_100-1550_pos_25-07-2023.m	Operator	Heiko Rudy
Sample Name	C34H52BN8O16P = 870,33 u	Instrument	micrOTOF-Q 228888.10254
Comment			

Acquisition Parameter

Source Type	ESI	Ion Polarity	Positive	Set Nebulizer	0.3 Bar
Focus	Not active	Set Capillary	4500 V	Set Dry Heater	200 °C
Scan Begin	100 m/z	Set End Plate Offset	-500 V	Set Dry Gas	4.0 l/min
Scan End	1550 m/z	Set Collision Cell RF	240.0 Vpp	Set Divert Valve	Source

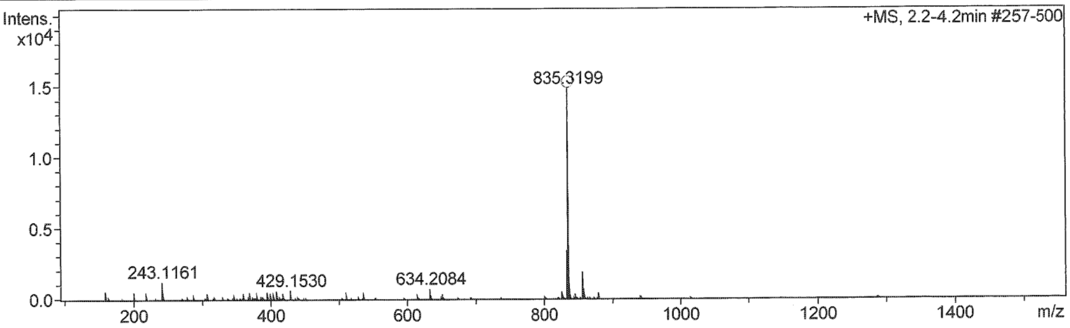

Meas. m/z	#	Ion Formula	m/z	err [ppm]	mSigma	# mSigma	Score	rdb	e⁻ Conf	N-Rule
835.3199	1	C34H49BN8O14P	835.3193	0.0	5.2	1	100.00	15.5	even	ok
	2	C38H53BN2O16P	835.3220	3.3	10.8	2	15.10	14.5	even	ok
	3	C33H53BN4O18P	835.3180	-1.6	12.8	3	44.48	10.5	even	ok
	4	C32H57BO22P	835.3167	-3.2	21.4	4	13.30	5.5	even	ok
	5	C29H49BN10O16P	835.3153	-4.9	22.2	5	3.00	11.5	even	ok
	6	C43H53BO14P	835.3260	8.2	28.7	6	0.04	18.5	even	ok
	7	C31H57BN2O21P	835.3279	10.2	668.8	7	0.00	5.5	even	ok
	1	C36H54BN2NaO16P	835.3196	0.4	6.1	1	100.00	11.5	even	ok
	2	C32H50BN8NaO14P	835.3169	-2.9	12.6	2	23.30	12.5	even	ok
	3	C41H54BNaO14P	835.3236	5.3	19.3	3	2.38	15.5	even	ok
	4	C31H54BN4NaO18P	835.3156	-4.5	21.3	4	5.13	7.5	even	ok
	5	C30H54BN6NaO17P	835.3268	8.9	23.1	5	0.02	7.5	even	ok
	6	C30H58BNaO22P	835.3143	-6.1	30.0	6	0.74	2.5	even	ok
	7	C29H58BN2NaO21P	835.3255	7.3	31.9	7	0.15	2.5	even	ok

Mass Spectrum List Report

Analysis Info

Analysis Name	E:\Data4\HEIKO\03_2024\Marius Werner\CTD_06_pure.d	Acquisition Date	3/28/2024 12:25:29 PM
Method	2_1_b_HR_kleine_Massen_100-1550_pos_25-07-2023.m	Operator	Heiko Rudy
Sample Name	C34H52BN8O16P = 870,33 u	Instrument	micrOTOF-Q 228888.10254
Comment			

Acquisition Parameter

Source Type	ESI	Ion Polarity	Positive	Set Nebulizer	0.3 Bar
Focus	Not active	Set Capillary	4500 V	Set Dry Heater	200 °C
Scan Begin	100 m/z	Set End Plate Offset	-500 V	Set Dry Gas	4.0 l/min
Scan End	1550 m/z	Set Collision Cell RF	240.0 Vpp	Set Divert Valve	Source

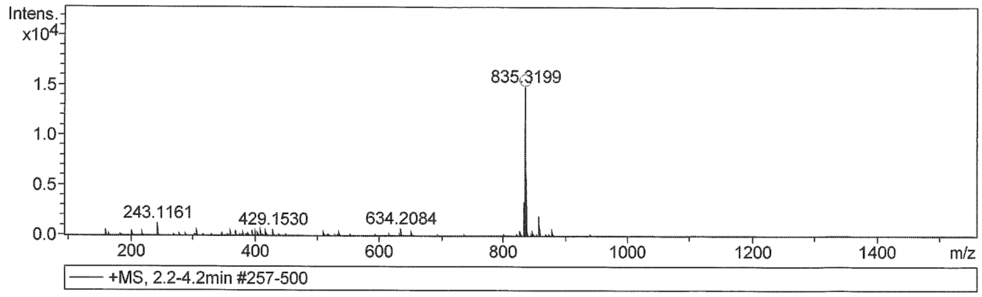

#	m/z	Res.	S/N	I	I %	FWHM
1	160.0757	5920	40.3	631	4.3	0.0270
2	202.1180	6961	33.3	511	3.4	0.0290
3	243.1161	7473	81.9	1261	8.5	0.0325
4	307.1561	8283	27.8	443	3.0	0.0371
5	360.1683	8300	28.3	455	3.1	0.0434
6	360.6647	8085	19.1	307	2.1	0.0446
7	369.1733	8659	34.7	559	3.8	0.0426
8	380.1647	8591	19.7	317	2.1	0.0443
9	395.6519	8426	31.4	501	3.4	0.0470
10	400.6432	9040	26.7	425	2.9	0.0443
11	404.6576	9052	20.2	321	2.2	0.0447
12	409.1568	8427	36.2	573	3.9	0.0486
13	409.6537	8833	29.4	465	3.1	0.0464
14	418.1629	8770	30.0	472	3.2	0.0477
15	429.1530	9326	41.6	646	4.4	0.0460
16	511.1584	9930	21.4	310	2.1	0.0515
17	536.2303	9966	21.9	305	2.1	0.0538
18	634.2084	10882	62.0	766	5.2	0.0583
19	652.2219	10476	29.8	368	2.5	0.0623
20	827.3318	12177	43.2	529	3.6	0.0679
21	834.3227	11693	277.1	3421	23.1	0.0714
22	835.3199	11327	1200.4	14822	100.0	0.0737
23	835.8200	11060	31.2	385	2.6	0.0756
24	836.3216	11665	484.2	5979	40.3	0.0717
25	837.3240	11674	118.4	1462	9.9	0.0717
26	846.3169	10237	29.1	360	2.4	0.0827
27	856.3039	11758	35.8	443	3.0	0.0728
28	857.3009	11586	156.8	1940	13.1	0.0740
29	858.3034	11672	60.9	753	5.1	0.0735
30	879.2840	11860	36.2	442	3.0	0.0741

4 Abbreviation

Ac	Acetyl
allylGly	Allylglycine
Boc	*tert*-Butoxycarbonyl
Bzl	Benzyl
COD	1,5-Cyclooctadiene
COSY	Correlation spectroscopy
Cp	Cyclopentadiene
Cp*	Pentamethylcyclopentadiene
DCM	Dichloromethane
DHB	2,5-Dihydroxybenzoic acid
DIC	*N*,*N'*-Diisopropylcarbodiimide
DIPEA	*N*,*N*-Diisopropylethylamine
DMF	*N*,*N*-Dimethylformamide
DMSO	Dimethyl sulfoxide
dppe	1,2-Bis(diphenylphosphino)ethane
dppf	1,1'-Bis(diphenylphosphino)ferrocene
dppm	1,1-Bis(diphenylphosphino)methane
ESI	Electrospray ionisation
Et_2O	Diethyl ether
FAM	5(6)-Carboxyfluorescein
Fmoc	Fluorenylmethyloxycarbonyl
HBpin	4,4,5,5-Tetramethyl-1,3,2-dioxaborolane (Pinacolborane)
HFIP	1,1,1,3,3,3-Hexafluoro-2-propanol
HOBt	1-Hydroxybenzotriazole
HPLC	High performance liquid chromatography
LSF	Late-stage-functionalization
MALDI	Matrix assisted laser desorption ionisation
MBHA	4-Methylbenzhydrylamine
MeCN	Acetonitrile
MeOH	Methanol
MHC	Major histocompatibility complex
MS	Mass spectrometry
Nle	Norleucine
Oxyma	Ethyl cyano(hydroxyimino)acetate
PCy_3	Tricyclohexylphosphine
Pin	2,3-Dimethyl-2,3-butandiol Pinacol
PPh_3	Triphenylphosphine
Pra	Propargylglycine
PyBOP	Benzotriazol-1-yloxytripyrrolidinophosphonium hexafluorophosphate
RP	Reversed phase
rt	Room temperature
SPPS	Solid-phase peptide synthesis
SPS	Solvent purification system
TFA	Trifluoroacetic acid
TIPS	Triisopropyl silane
TOF	Time-of-flight

5 Literature

[1] J. B. Crumpton, W. Zhang, W. L. Santos, *Anal. Chem.* **2011**, *83*, 3548-3554.
[2] T. Michels, R. Dölling, U. Haberkorn, W. Mier, *Org. Lett.* **2012**, *14*, 5218-5221.
[3] B. C. Gorske, S. A. Jewell, E. J. Guerard, H. E. Blackwell, *Org. Lett.* **2005**, *7*, 1521-1524.
[4] V. Reusche, F. Thomas, *ChemBioChem* **2021**, *22*, 1779-1783.
